# Supplementary material for: Palladium-catalyzed regio- and enantioselective migratory allylic C(sp3)-H functionalization
Source: Nat Commun. 2021 Sep 24;12:5626. doi: 10.1038/s41467-021-25978-6 (PMC8463607; doi:10.1038/s41467-021-25978-6)
Supplement: Supplementary file 1 — Supplementary Information [file 41467_2021_25978_MOESM1_ESM.pdf]

Supplementary Information for

Palladium-Catalyzed Regio- and Enantioselective Migratory Allylic C(sp^3)-H Functionalization

Ye-Wei Chen,[1,2,3] Yang Liu,[1,3] Han-Yu Lu,[1,2] Guo-Qiang Lin[1,2,*] and Zhi-Tao He[1,*]

[1] Key Laboratory of Synthetic Chemistry of Natural Substances, Center for Excellence in Molecular Synthesis, Shanghai Institute of Organic Chemistry, University of Chinese Academy of Sciences, Shanghai, 200032, China.

[2] School of Physical Science and Technology, ShanghaiTech University, Shanghai, 201210, China.

[3] These authors contributed equally to this work.

*Correspondence to: lingq@sioc.ac.cn (G.-Q.L.); hezt@sioc.ac.cn (Z.-T.H.)

Supplementary Methods…………………………………………………………S2

1. General information ……………………………………………………S2

2. Synthesis of substrates …………………………………………………S2

3. Development of reaction conditions……………………………………S13

4. General procedure for Pd-catalyzed migratory allylic functionalization……S14

5. Chain walking test of different nucleophiles……………………………S38

6. Substrates ineffective for the migratory allylation………………………S40

7. Mechanistic studies………………………………………………………S41

8. Regioconvergent synthesis, gram-scale test and transformations of allylation products………S57

9. X-ray crystal structure of compound **11**……………………………………S61

10. Copies of ^{1}H NMR, ^{13}C NMR, and ^{19}F NMR spectra…………………S71

Supplementary References……………………………………………S155

Supplementary Methods

1. General information

All air-sensitive procedures were conducted by Schlenk techniques under argon. Unless otherwise indicated, all commercially available starting materials and dry solvents were purchased and used directly without further purification. ^{1}H, ^{13}C and ^{19}F NMR spectra were acquired on 400 MHz Bruker or 500 MHz Agilent instruments at Shanghai Institute of Organic Chemistry. For High-resolution mass spectra: ESI mass spectra were recorded on Thermo Scientific Q Exactive HF Orbitrap-FTMS; MALDI was measured on Voyager-DE STR; EI mass spectra were recorded on Waters Premier GC-TOF MS; FI mass spectra were recorded on JEOL-AccuTOF-GCv4G-GCT MS. Optical rotation was measured using a 1 mL cell with 1.0 dm path length on a JASCO P-1030 polarimeter. HPLC analysis was conducted on a Shimadzu HPLC system equipped with Daicel or Chiralpak chiral-stationary-phase columns (ϕ 4.6 mm×250 mm). Chemical shifts are reported in δ (ppm) referenced to an internal TMS standard or $CHCl_3$ in $CDCl_3$ (7.26 ppm) for ^{1}H NMR, $CDCl_3$ (δ = 77.10 ppm) for ^{13}C NMR, and $CFCl_3$ (0 ppm) for ^{19}F NMR. Coupling constants (*J*) are reported in Hz. Multiplicities are reported using the following abbreviations: br = broad, s = singlet, d = doublet, t = triplet, q = quartet, m = multiplet. Column chromatography was performed with 300-400 mesh silica gel using flash column chromatography technique.

2. Synthesis of substrates

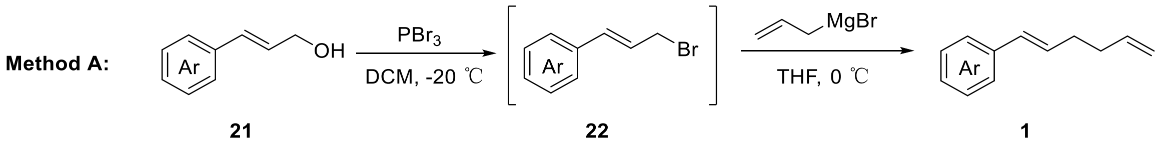

Method A: To a 25 mL flask were added the allyl alcohol **21** (1 mmol) and DCM (10 mL, 0.1 M) under nitrogen. Then PBr_3 (0.3 mmol) was added to the solution dropwise at -20 ℃. The resulting mixture was stirred at -20 ℃ and monitored by TLC until the complete consumption of substrate **21**. Then the reaction was condensed directly to afford the crude allyl bromide **22** without further purification. Next, the allyl bromide **22** was dissolved in dry THF (2 mL) and the reaction mixture was cooled down to 0 ℃, followed by the addition of allyl magnesium bromide (2 mmol, 1 mL, 2 M in THF) dropwise over 5 min. After the fully conversion of intermediate **22**, the reaction was condensed and purified by flash column chromatography to give the distant diene compound **1**.

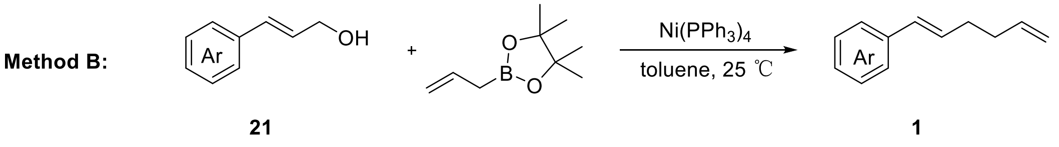

Method B was conducted according to the reported literature [1]: To a 25 mL Schlenk tube were added tertrakis(triphenylphosphine)nickel (0.15 mmol, 10 mol%), allyl alcohol (1.5 mmol, 1.0 equiv) and toluene (15 mL). The Allylboronic acid pinacol ester (1.8 mmol) was then added to the mixture dropwise over 5 min at room temperature. After this time, the resulting mixture continued to stir at room temperature and was monitored by TLC. The reaction was diluted with diethyl ether (6 mL), filtered through a pad of

silica gel, condensed and purified by flash column chromatography to give the distant diene compound **1**.

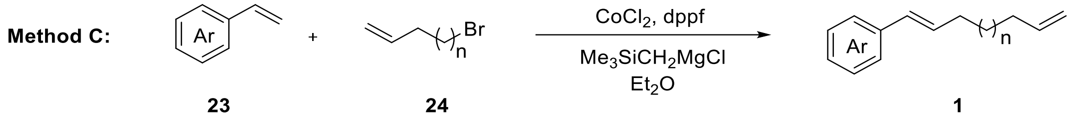

Method C was conducted according to the reported literature[2]: To a 50 mL flask were added $CoCl_2$ (0.15 mmol, 5.0 mol%) 1,1'-Bis(diphenylphosphino)ferrocene (dppf, 0.18 mmol, 6.0 mol%) and Et_2O (10 mL) under nitrogen. The mixture was stirred at room temperature for 30 min. Then alkyl bromide **24** (4.5 mmol, 1.5 equiv), alkene **23** (3.0 mmol, 1.0 equiv) and (trimethylsilyl)methylmagnesium chloride (7.5 mmol, 2.5 equiv) were added to the reaction sequentially at 0 °C. The resulting mixture was stirred at room temperature for 6 h. After this time, the reaction was quenched by saturated aqueous NH_4Cl solution (10 mL), exacted by ethyl acetate (10 mL × 3), condensed and purified by flash column chromatography to give the pure desired products **1**.

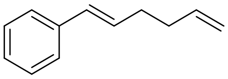

(*E*)-Hexa-1,5-dien-1-ylbenzene (1a)

1a was prepared according to method A. Known compound[1]. Colorless oil, 65% yield. ^{1}H NMR (600 MHz, chloroform-*d*) δ 7.33 – 7.32 (m, 2H), 7.29 – 7.23 (m, 2H), 7.21 – 7.13 (m, 1H), 6.39 (d, *J* = 15.8 Hz, 1H), 6.26 – 6.15 (m, 1H), 5.88 – 5.82 (m, 1H), 5.11 – 4.94 (m, 2H), 2.33 – 2.14 (m, 4H). HRMS (EI): $[M]^{\oplus}$calcd for $C_{12}H_{14}^{\oplus}$ 158.1090, found 158.1093.

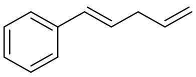

(*E*)-Penta-1,4-dien-1-ylbenzene (1b)

1b was prepared according to the reported literature[3]. Colorless oil, 65% yield. ^{1}H NMR (400 MHz, chloroform-*d*) δ 7.36 (d, *J* = 6.7 Hz, 2H), 7.33 – 7.27 (m, 2H), 7.25 – 7.17 (m, 1H), 6.42 (d, *J* = 15.8 Hz, 1H), 6.23 (dt, *J* = 15.7, 6.6 Hz, 1H), 5.99 – 5.84 (m, 1H), 5.17 – 5.07 (m, 1H), 5.11 – 5.04 (m, 1H), 2.97 (t, *J* = 6.6 Hz, 2H). HRMS (EI): $[M]^{\oplus}$calcd for $C_{11}H_{12}^{\oplus}$ 144.0934, found 144.0935.

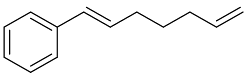

(*E*)-Hepta-1,6-dien-1-ylbenzene (1c)

1c was prepared according to method C. Known compound[4]. Colorless oil, 58% yield. ^{1}H NMR (400 MHz, chloroform-*d*) δ 7.35 – 7.26 (m, 4H), 7.22 – 7.14 (m, 1H), 6.39 (d, *J* = 15.8 Hz, 1H), 6.21 (dt, *J* = 15.8, 6.9 Hz, 1H), 5.89 – 5.77 (m, 1H), 5.07 – 4.97 (m, 2H), 2.25 – 2.20 (m, 2H), 2.14 – 2.09 (m, 2H), 1.61 – 1.54 (m, 2H). HRMS (EI): $[M]^{\oplus}$calcd for $C_{13}H_{16}^{\oplus}$ 172.1247, found 172.1250.

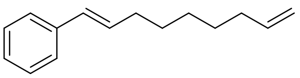

(*E*)-Nona-1,8-dien-1-ylbenzene (1d)

1d was prepared according to method C. Colorless oil, 68% yield. ^{1}H NMR (500 MHz, chloroform-*d*) δ 7.33 (d, *J* = 8.3 Hz, 2H), 7.27 (t, *J* = 7.6 Hz, 2H), 7.17 (t, *J* = 7.3 Hz, 1H), 6.37 (d, *J* = 15.8 Hz, 1H), 6.21 (dt, *J* = 15.6, 6.9 Hz, 1H), 5.85 – 5.77 (m, 1H), 5.04 – 4.92 (m, 2H), 2.22 – 2.17 (m, 2H), 2.07 – 2.03 (m, 2H), 1.50 – 1.44 (m, 2H), 1.42 – 1.32 (m, 4H). ^{13}C NMR (126 MHz, chloroform-*d*) δ 139.1, 138.0, 131.1, 129.8, 128.5, 126.8, 126.0, 114.3, 33.8, 33.1, 29.3, 28.9, 28.8. HRMS (EI): [M]$^{\oplus}$calcd for $C_{15}H_{20}{}^{\oplus}$ 200.1560, found 200.1564.

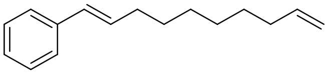

(*E*)-Deca-1,9-dien-1-ylbenzene (1e)

1e was prepared according to method C. Colorless oil, 71% yield. ^{1}H NMR (500 MHz, chloroform-*d*) δ 7.37 – 7.26 (m, 4H), 7.22 – 7.14 (m, 1H), 6.40 – 6.36 (m, 1H), 6.23 (dt, *J* = 15.8, 6.9 Hz, 1H), 5.86 – 5.78 (m, 1H), 5.02 – 4.91 (m, 2H), 2.23 – 2.18 (m, 2H), 2.09 – 2.01 (m, 2H), 1.52 – 1.29 (m, 8H). ^{13}C NMR (126 MHz, chloroform-*d*) δ 139.3, 138.0, 131.3, 129.8, 128.6, 126.8, 126.0, 114.3, 33.9, 33.1, 29.4, 29.1, 29.1, 29.0. HRMS (EI): [M]$^{\oplus}$calcd for $C_{16}H_{22}{}^{\oplus}$ 214.1716, found 214.1720.

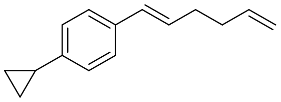

(*E*)-1-Cyclopropyl-4-(hexa-1,5-dien-1-yl)benzene (1f)

1f was prepared according to method B. Colorless oil, 56% yield. ^{1}H NMR (400 MHz, chloroform-*d*) δ 7.22 (d, *J* = 8.1 Hz, 2H), 6.98 (d, *J* = 8.1 Hz, 2H), 6.35 (d, *J* = 15.8 Hz, 1H), 6.15 (dt, *J* = 15.8, 6.6 Hz, 1H), 5.92 – 5.78 (m, 1H), 5.11 – 4.91 (m, 2H), 2.34 – 2.15 (m, 4H), 1.88 – 1.81 (m, 1H), 0.96 – 0.89 (m, 2H), 0.69 – 0.64 (m, 2H). ^{13}C NMR (126 MHz, chloroform-*d*) δ 142.7, 138.2, 135.1, 130.1, 128.9, 125.9, 125.8, 114.9, 33.7, 32.5, 15.3, 9.2. HRMS (EI): [M]$^{\oplus}$calcd for $C_{15}H_{18}{}^{\oplus}$ 198.1403, found 198.1402.

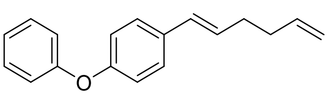

(*E*)-1-(Hexa-1,5-dien-1-yl)-4-phenoxybenzene (1g)

1g was prepared according to method B. Colorless oil, 47% yield. ^{1}H NMR (500 MHz, chloroform-*d*) δ 7.34 – 7.30 (m, 4H), 7.09 (t, *J* = 7.3 Hz, 1H), 7.00 (d, *J* = 8.0 Hz, 2H), 6.94 (d, *J* = 8.2 Hz, 2H), 6.37 (d, *J* = 15.7 Hz, 1H), 6.18 – 6.12 (m, 1H), 5.91 – 5.83 (m, 1H), 5.09 – 5.08 (m, 1H), 5.01 – 5.99 (m, 1H), 2.33 – 2.29 (m, 2H), 2.25 – 2.21 (m, 2H). ^{13}C NMR (126 MHz, chloroform-*d*) δ 169.1, 168.0, 160.1, 138.2, 129.8, 129.4, 129.4, 127.3, 123.2, 119.1, 118.8, 115.0, 33.7, 32.5. HRMS (ESI): [M+H]$^{\oplus}$calcd for $C_{18}H_{19}O^{\oplus}$ 251.1430, found 251.1429.

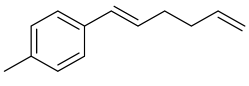

(*E*)-1-(Hexa-1,5-dien-1-yl)-4-methylbenzene (1h)

1h was prepared according to method B. Known compound[5]. Colorless oil, 71% yield. ^{1}H NMR (400 MHz, chloroform-*d*) δ 7.23 (d, *J* = 8.0 Hz, 2H), 7.09 (d, *J* = 7.9 Hz, 2H), 6.36 (d, *J* = 15.8 Hz, 1H), 6.16 (dt, *J* = 15.8, 6.6 Hz, 1H), 5.93 – 5.78 (m, 1H), 5.10 – 5.00 (m, 1H), 5.02 – 4.94 (m, 1H), 2.33 – 2.24 (m, 5H), 2.28 – 2.16 (m, 2H). HRMS (EI): $[M]^{\oplus}$ calcd for $C_{13}H_{16}^{\oplus}$ 172.1247, found 172.1247.

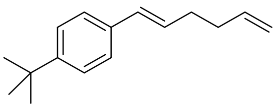

(*E*)-1-(*tert*-Butyl)-4-(hexa-1,5-dien-1-yl)benzene (1i)

1i was prepared according to method B. Known compound[6]. Colorless oil, 36% yield. ^{1}H NMR (500 MHz, chloroform-*d*) δ 7.47 – 7.38 (m, 4H), 6.52 (d, *J* = 15.8 Hz, 1H), 6.31 (dt, *J* = 15.9, 6.8 Hz, 1H), 6.07 – 5.91 (m, 1H), 5.23 – 5.15 (m, 1H), 5.15 – 5.09 (m, 1H), 2.45 – 2.41 (m, 2H), 2.39 – 2.31 (m, 2H), 1.44 (s, 9H). HRMS (EI): $[M]^{\oplus}$ calcd for $C_{16}H_{22}^{\oplus}$ 214.1716, found 214.1720.

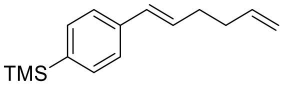

(*E*)-(4-(Hexa-1,5-dien-1-yl)phenyl)trimethylsilane (1j)

1j was prepared according to method B. Colorless oil, 41% yield. ^{1}H NMR (500 MHz, chloroform-*d*) δ 7.62 – 7.54 (m, 2H), 7.47 – 7.42 (m, 2H), 6.51 (d, *J* = 15.9 Hz, 1H), 6.37 (dt, *J* = 15.9, 6.7 Hz, 1H), 6.01 – 5.93 (m, 1H), 5.27 – 5.04 (m, 2H), 2.47 – 2.39 (m, 2H), 2.38 – 2.30 (m, 2H), 0.38 (s, 9H). ^{13}C NMR (126 MHz, chloroform-*d*) δ 139.0, 138.3, 138.1, 133.6, 130.5, 130.3, 125.4, 115.0, 33.6, 32.6, -1.01. HRMS (EI): $[M]^{\oplus}$ calcd for $C_{15}H_{22}Si^{\oplus}$ 230.1485, found 230.1489.

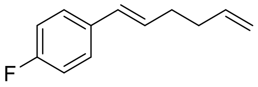

(*E*)-1-Fluoro-4-(hexa-1,5-dien-1-yl)benzene (1k)

1k was prepared according to method B. Known compounds[7]. Colorless oil, 68% yield. ^{1}H NMR (400 MHz, chloroform-*d*) δ 7.38 – 7.24 (m, 2H), 7.03 – 6.92 (m, 2H), 6.36 (d, *J* = 15.8 Hz, 1H), 6.13 (dt, *J* = 15.9, 6.6 Hz, 1H), 5.96 – 5.78 (m, 1H), 5.14 – 4.95 (m, 2H), 2.35 – 2.17 (m, 4H). HRMS (EI): $[M]^{\oplus}$ calcd for $C_{12}H_{13}F^{\oplus}$ 176.1003, found 176.1000.

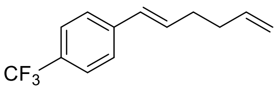

(*E*)-1-(Hexa-1,5-dien-1-yl)-4-(trifluoromethyl)benzene (1l)

1l was prepared according to method B. Known compounds[7]. Colorless oil, 70% yield. ^{1}H NMR (500 MHz, chloroform-*d*) δ 7.56 (d, *J* = 7.9 Hz, 2H), 7.44 (d, *J* = 8.0 Hz, 2H), 6.49 – 6.33 (m, 1H), 6.41 – 6.32 (m, 1H), 5.99 – 5.81 (m, 1H), 5.20 – 4.97 (m, 2H), 2.41 – 2.33 (m, 2H), 2.32 – 2.24 (m, 2H). HRMS (EI): $[M]^{\oplus}$calcd for $C_{13}H_{13}F_{3}^{\oplus}$ 226.0694, found 226.0697.

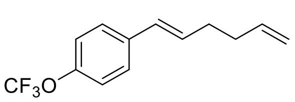

(*E*)-1-(Hexa-1,5-dien-1-yl)-4-(trifluoromethoxy)benzene (**1m**)

1m was prepared according to method A. Colorless oil, 51% yield. ^{1}H NMR (500 MHz, chloroform-*d*) δ 7.39 – 7.33 (m, 2H), 7.16 (d, *J* = 8.2 Hz, 2H), 6.41 (d, *J* = 15.8 Hz, 1H), 6.23 (dt, *J* = 15.8, 6.7 Hz, 1H), 5.93 – 5.85 (m, 1H), 5.15 – 4.97 (m, 2H), 2.38 – 2.31 (m, 2H), 2.28 – 2.24 (m, 2H). ^{13}C NMR (126 MHz, chloroform-*d*) δ 148.1, 138.0, 136.7, 131.3, 128.9, 127.2, 121.1, 120.6 (q, *J* = 257.0 Hz), 115.1, 33.5, 32.4. HRMS (EI): [M]$^{\oplus}$calcd for $C_{13}H_{13}F_3O^{\oplus}$ 242.0913, found 242.0910.

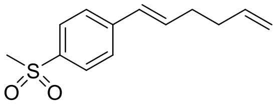

(*E*)-1-(Hexa-1,5-dien-1-yl)-4-(methylsulfonyl)benzene (**1n**)

1n was prepared according to method A. Yellow oil, 62% yield. ^{1}H NMR (600 MHz, chloroform-*d*) δ 7.88 – 7.83 (m, 2H), 7.52 – 7.47 (m, 2H), 6.48 – 6.37 (m, 2H), 5.88 – 5.82 (m, 1H), 5.14 – 4.95 (m, 2H), 3.04 (s, 3H), 2.39 – 2.33 (m, 2H), 2.29 – 2.22 (m, 2H). ^{13}C NMR (151 MHz, chloroform-*d*) δ 143.2, 138.3, 137.6, 134.7, 128.7, 127.7, 126.6, 115.3, 44.6, 33.1, 32.4. HRMS (ESI): [M+H] $^{\oplus}$calcd for $C_{13}H_{17}O_2S^{\oplus}$ 237.0944, found 237.0944.

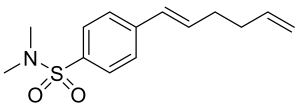

(*E*)-4-(Hexa-1,5-dien-1-yl)-*N*,*N*-dimethylbenzenesulfonamide (**1o**)

1o was prepared according to method A. Colorless oil, 42% yield. ^{1}H NMR (500 MHz, chloroform-*d*) δ 7.69 (d, *J* = 8.1 Hz, 2H), 7.48 (d, *J* = 8.1 Hz, 2H), 6.45 (d, *J* = 16.0 Hz, 1H), 6.44 – 6.34 (m, 1H), 5.90 – 5.82 (m, 1H), 5.12 – 4.99 (m, 2H), 2.69 (s, 6H), 2.40 – 2.32 (m, 2H), 2.30 – 2.21 (m, 2H). ^{13}C NMR (126 MHz, chloroform-*d*) δ 142.2, 137.7, 134.1, 133.2, 128.8, 128.1, 126.3, 115.3, 38.0, 33.2, 32.4. HRMS (EI): [M]$^{\oplus}$calcd for $C_{14}H_{19}O_2NS^{\oplus}$ 265.1131, found 265.1134.

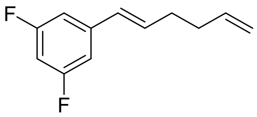

(*E*)-1,3-Difluoro-5-(hexa-1,5-dien-1-yl)benzene (**1p**)

1p was prepared according to method B. Colorless oil, 51% yield. ^{1}H NMR (500 MHz, chloroform-*d*) δ 6.84 (d, *J* = 7.2 Hz, 2H), 6.64 (t, *J* = 8.9 Hz, 1H), 6.37 – 6.20 (m, 2H), 5.92 – 5.78 (m, 1H), 5.05 (dd, *J* = 27.0, 13.7 Hz, 2H), 2.38 – 2.29 (m, 2H), 2.29 – 2.20 (m, 2H). ^{13}C NMR (126 MHz, chloroform-*d*) δ 163.3 (dd, *J* = 247.2, 13.2 Hz), 141.3 (t, *J* = 9.5 Hz), 137.8, 133.1, 128.6 (t, *J* = 2.8 Hz), 115.3, 108.7 (dd, *J* =

19.4, 5.7 Hz), 102.1 (t, J = 25.7 Hz), 33.3, 32.3. ^{19}F NMR (376 MHz, Methylene Chloride-d_2) δ -111.82 (t, J = 7.9 Hz). HRMS (EI): $[M]^{\oplus}$calcd for $C_{12}H_{12}F_2^{\oplus}$ 194.0902, found 194.0905.

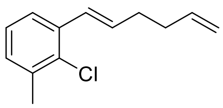

(*E*)-2-Chloro-1-(hexa-1,5-dien-1-yl)-3-methylbenzene (**1q**)

1q was prepared according to method B. Colorless oil, 58% yield. ^{1}H NMR (500 MHz, chloroform-d) δ 7.35 – 7.33 (m, 1H), 7.10 (d, J = 4.8 Hz, 2H), 6.85 – 6.82 (m, 1H), 6.20 – 6.14 (m, 1H), 5.94 – 5.81 (m, 1H), 5.12 – 4.97 (m, 2H), 2.38 – 2.34 (m, 5H), 2.29 – 2.24 (m, 2H). ^{13}C NMR (126 MHz, chloroform-d) δ 138.1, 136.6, 136.3, 133.0, 129.3, 127.3, 126.2, 124.4, 115.1, 33.5, 32.6, 20.9 (one aromatic carbon signal was not observed because of overlapping). HRMS (EI): $[M]^{\oplus}$calcd for $C_{13}H_{15}Cl^{\oplus}$ 206.0857, found 206.0858.

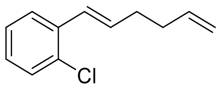

(*E*)-1-Chloro-2-(hexa-1,5-dien-1-yl)benzene (**1r**)

1r was prepared according to method A. Colorless oil, 24% yield. ^{1}H NMR (500 MHz, chloroform-d) δ 7.46 (dd, J = 7.7, 1.7 Hz, 1H), 7.30 (d, J = 7.9 Hz, 1H), 7.15 (t, J = 7.5 Hz, 1H), 7.12 – 7.06 (m, 1H), 6.78 (d, J = 15.8 Hz, 1H), 6.18 (dt, J = 15.8, 6.8 Hz, 1H), 5.89 – 5.81 (m, 1H), 5.08 – 5.04 (m, 1H), 5.00 (d, J = 9.9 Hz, 1H), 2.38 – 2.30 (m, 2H), 2.26 – 2.21 (m, 2H). ^{13}C NMR (126 MHz, chloroform-d) δ 137.9, 135.8, 133.0, 132.6, 129.6, 128.0, 126.8, 126.7, 126.5, 115.1, 100.0, 33.4, 32.6. HRMS (EI): $[M]^{\oplus}$calcd for $C_{12}H_{13}Cl^{\oplus}$ 192.0700, found 192.0702.

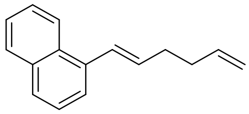

(*E*)-1-(Hexa-1,5-dien-1-yl)naphthalene (**1s**)

1s was prepared according to method B. Known compound[8]. Colorless oil, 50% yield. ^{1}H NMR (500 MHz, chloroform-d) δ 8.09 (d, J = 8.4 Hz, 1H), 7.79 (dd, J = 7.6, 1.9 Hz, 1H), 7.70 (d, J = 8.2 Hz, 1H), 7.52 – 7.36 (m, 4H), 7.13 – 7.08 (m, 1H), 6.24 – 6.15 (m, 1H), 5.97 – 5.80 (m, 1H), 5.14 – 4.96 (m, 2H), 2.43 – 2.35 (m, 2H), 2.33 – 2.22 (m, 2H). HRMS (EI): $[M]^{\oplus}$calcd for $C_{16}H_{16}^{\oplus}$ 208.1247, found 208.1244.

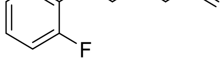

(*E*)-1-Fluoro-2-(hexa-1,5-dien-1-yl)benzene (**1t**)

1t was prepared according to method B. Known compound[9]. Colorless oil, 37% yield. ^{1}H NMR (500 MHz, chloroform-d) δ 7.42 (td, J = 7.7, 1.9 Hz, 1H), 7.20 – 7.12 (m, 1H), 7.10 – 7.04 (m, 1H), 7.01 (ddd, J = 10.8, 8.1, 1.3 Hz, 1H), 6.58 – 6.54 (m, 1H), 6.30 (dt, J = 16.0, 6.8 Hz, 1H), 5.92 – 5.81 (m, 1H), 5.11

– 5.03 (m, 1H), 5.03 – 4.97 (m, 1H), 2.39 – 2.30 (m, 2H), 2.29 – 2.20 (m, 2H). HRMS (EI): [M]$^{\oplus}$calcd for $C_{12}H_{13}F^{\oplus}$ 176.0996, found 176.0995.

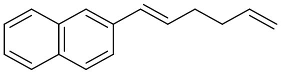

(*E*)-2-(Hexa-1,5-dien-1-yl)naphthalene (**1u**)

1u was prepared according to method B. Known compound[10]. Yellow solid, 80% yield. ^{1}H NMR (400 MHz, chloroform-*d*) δ 7.80 – 7.74 (m, 3H), 7.67 (s, 1H), 7.60 – 7.55 (m, 1H), 7.46 – 7.38 (m, 2H), 6.56 (d, *J* = 15.9 Hz, 1H), 6.36 (dt, *J* = 15.9, 6.6 Hz, 1H), 5.97 – 5.82 (m, 1H), 5.14 – 5.05 (m, 1H), 5.05 – 4.98 (m, 1H), 2.42 – 2.32 (m, 2H), 2.32 – 2.22 (m, 2H). HRMS (EI): [M]$^{\oplus}$calcd for $C_{16}H_{16}^{\oplus}$ 208.1247, found 208.1251.

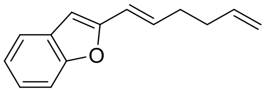

(*E*)-2-(Hexa-1,5-dien-1-yl)benzofuran (**1v**)

1v was prepared according to method B. Colorless oil, 47% yield. ^{1}H NMR (600 MHz, chloroform-*d*) δ 7.49 – 7.47 (m, 1H), 7.41 (d, *J* = 8.1 Hz, 1H), 7.26 – 7.14 (m, 2H), 6.50 – 6.45 (m, 2H), 6.35 – 6.32 (m, 1H), 5.90 – 5.83 (m, 1H), 5.13 – 5.05 (m, 1H), 5.04 – 4.99 (m, 1H), 2.39 – 2.32 (m, 2H), 2.31 – 2.23 (m, 2H). ^{13}C NMR (151 MHz, chloroform-*d*) δ 155.1, 154.7, 137.9, 132.9, 129.2, 124.1, 122.8, 120.7, 119.1, 115.3, 110.9, 103.0, 33.2, 32.4. HRMS (EI): [M]$^{\oplus}$calcd for $C_{14}H_{14}O^{\oplus}$ 198.1039, found 198.1041.

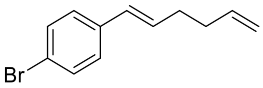

(*E*)-1-Bromo-4-(hexa-1,5-dien-1-yl)benzene (**1w**)

1w was prepared according to method A. Known compound[11]. Colorless oil, 80% yield. ^{1}H NMR (500 MHz, chloroform-*d*) δ 7.39 (d, *J* = 8.4 Hz, 2H), 7.19 (d, *J* = 8.5 Hz, 2H), 6.33 (d, *J* = 15.8 Hz, 1H), 6.25 – 6.16 (m, 1H), 5.90 – 5.79 (m, 1H), 5.08 – 5.03 (m, 1H), 5.01 – 4.99 (m, 1H), 2.33 – 2.26 (m, 2H), 2.26 – 2.18 (m, 2H). ^{13}C NMR (126 MHz, chloroform-*d*) δ 137.99, 136.74, 131.60, 131.06, 129.15, 127.58, 120.58, 115.14, 33.45, 32.44. HRMS (EI): [M]$^{\oplus}$calcd for $C_{12}H_{13}Br^{\oplus}$ 236.0195, found 236.0197.

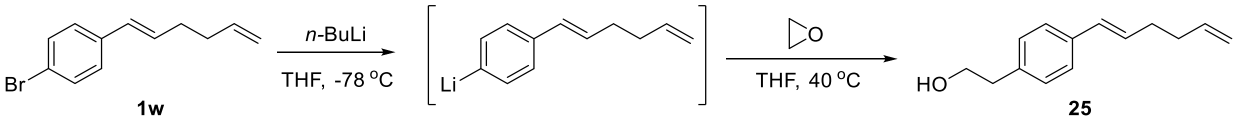

(*E*)-2-(4-(Hexa-1,5-dien-1-yl)phenyl)ethan-1-ol (**25**): To a 100 mL Schlenk tube with a magnetic stirring bar was added **1w** (2.4 g, 10 mmol) and THF (50 mL) under nitrogen. Then *n*-BuLi (1 M in THF, 15 mL) was added dropwise at -78 °C and the mixture continued to stir at room temperature for 2 h. Next, epoxyethane (3 M in THF, 12 mmol) was added to the reaction. The resulting mixture stirred at 40 ^{0}C for 5 h. After this time, the reaction was quenched by ethyl acetate (15 mL), concentrated and purified by

flash column chromatography (hexane/ethyl acetate = 3/1) to give compound **25** as white solid in 58% yield. ^{1}H NMR (500 MHz, chloroform-*d*) δ 7.29 (d, *J* = 7.8 Hz, 2H), 7.16 (d, *J* = 7.8 Hz, 2H), 6.38 (d, *J* = 15.8 Hz, 1H), 6.20 (dt, *J* = 15.7, 6.7 Hz, 1H), 5.90 – 5.82 (m, 1H), 5.06 (dd, *J* = 17.2, 2.0 Hz, 1H), 4.99 (dd, *J* = 10.1, 2.0 Hz, 1H), 3.84 (t, *J* = 6.5 Hz, 2H), 2.84 (t, *J* = 6.6 Hz, 2H), 2.33 – 2.29 (m, 2H), 2.25 – 2.21 (m, 2H), 1.42 (s, 1H). ^{13}C NMR (126 MHz, chloroform-*d*) δ 138.2, 137.2, 136.2, 129.9, 129.9, 129.3, 126.3, 115.0, 63.7, 38.9, 33.6, 32.5. HRMS (EI): $[M]^{\oplus}$calcd for $C_{14}H_{18}O^{\oplus}$ 202.1352, found 202.1354.

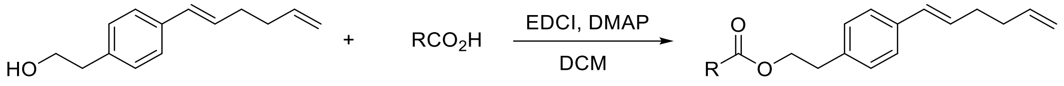

25 **9**, **26-33**

General procedure for esterification: To a 50 mL dry flask with a magnetic stirring bar were added **25** (0.4 g, 2 mmol), EDCI (0.42 g, 2.2 mmol), DMAP (0.6 g, 0.5 mmol), carboxylic acid (2 mmol) and DCM (10 mL). The reaction was stirred at room temperature for 12 h and monitored by TLC. The reaction was quenched by water (10 mL), exacted by DCM (10 mL × 3), condensed and purified by flash column chromatography to give the desired ester.

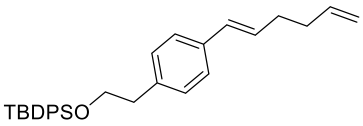

(*E*)-*tert*-Butyl(4-(hexa-1,5-dien-1-yl)phenethoxy)diphenylsilane (26)

Colorless oil, 95% yield. ^{1}H NMR (500 MHz, chloroform-*d*) δ 7.61 – 7.56 (m, 4H), 7.40 (t, *J* = 7.3 Hz, 2H), 7.34 (t, *J* = 7.4 Hz, 4H), 7.23 (d, *J* = 7.8 Hz, 2H), 7.06 (d, *J* = 7.8 Hz, 2H), 6.37 (d, *J* = 15.8 Hz, 1H), 6.23 – 6.12 (m, 1H), 5.92 – 5.81 (m, 1H), 5.09 – 4.96 (m, 2H), 3.82 (t, *J* = 6.9 Hz, 2H), 2.82 (t, *J* = 6.9 Hz, 2H), 2.34 – 2.26 (m, 2H), 2.22 (q, *J* = 7.1 Hz, 2H), 1.02 (s, 9H). ^{13}C NMR (126 MHz, chloroform-*d*) δ 138.4, 137.9, 135.8, 135.7, 133.9, 130.1, 129.6, 129.4, 129.4, 127.67, 125.9, 115.0, 65.3, 39.1, 33.7, 32.5, 26.9, 19.2. HRMS (ESI): $[M+NH_4]^{\oplus}$calcd for $C_{30}H_{40}ONSi$ 458.2874, found 458.2876.

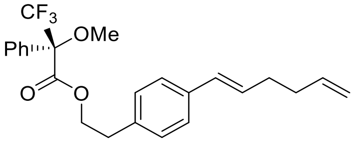

(*E*) 4-(Hex-1-en-1-yl)phenethyl (*S*)-3,3,3-trifluoro-2-methoxy-2-phenylpropanoate (27)

Colorless oil, 30% yield. ^{1}H NMR (500 MHz, chloroform-*d*) δ 7.44 – 7.36 (m, 3H), 7.33 (dd, *J* = 8.2, 6.6 Hz, 2H), 7.25 (d, *J* = 7.9 Hz, 2H), 7.09 (d, *J* = 7.8 Hz, 2H), 6.37 (d, *J* = 15.8 Hz, 1H), 6.20 (dt, *J* = 15.7, 6.7 Hz, 1H), 5.91 – 5.83 (m, 1H), 5.14 – 4.96 (m, 2H), 4.51 (t, *J* = 6.9 Hz, 2H), 3.47 (s, 3H), 3.02 – 2.93 (m, 2H), 2.38 – 2.28 (m, 2H), 2.28 – 2.18 (m, 2H). ^{13}C NMR (126 MHz, chloroform-*d*) δ 166.6, 138.2, 136.5, 135.7, 132.3, 130.1, 129.9, 129.6, 129.1, 128.5, 127.3, 126.2, 115.0, 66.9, 55.5, 51.0 (q, *J* = 2.52 Hz), 34.5, 33.6, 32.5 (the carbon signal of CF_3 was not observed). ^{19}F NMR (376 MHz, Chloroform-*d*) δ -71.69. HRMS (ESI): $[M+Na]^{\oplus}$calcd for $C_{24}H_{25}O_3F_3Na^{\oplus}$ 441.1648, found 441.1647.

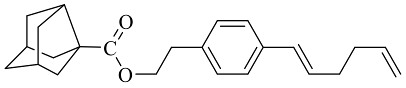

4-((*E*)-Hexa-1,5-dien-1-yl)phenethyl-hexahydro-2,5-methanopentalene-3a(1*H*)-carboxylate (**28**)

Yellow oil, 61% yield. ^{1}H NMR (500 MHz, chloroform-*d*) δ 7.27 (d, *J* = 7.9 Hz, 2H), 7.15 (d, *J* = 7.7 Hz, 2H), 6.38 (d, *J* = 15.8 Hz, 1H), 6.20 (dt, *J* = 15.7, 6.7 Hz, 1H), 5.93 – 5.80 (m, 1H), 5.11 – 4.96 (m, 2H), 4.27 (t, *J* = 6.8 Hz, 2H), 2.91 (t, *J* = 6.9 Hz, 2H), 2.59 (t, *J* = 6.8 Hz, 1H), 2.35 – 2.19 (m, 6H), 2.07 – 1.97 (m, 2H), 1.81 – 1.71 (m, 4H), 1.65 – 1.52 (m, 4H). ^{13}C NMR (126 MHz, chloroform-*d*) δ 177.6, 138.2, 136.9, 136.1, 129.9, 129.7, 129.2, 126.0, 115.0, 64.8, 53.8, 46.9, 44.1, 43.7, 37.5, 35.0, 34.8, 33.6, 32.5. HRMS (ESI): [M+Na]$^{\oplus}$calcd for $C_{24}H_{30}O_2Na^{\oplus}$ 373.2138, found 373.2137.

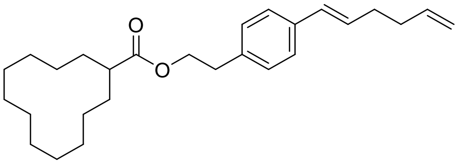

(*E*)-4-(Hexa-1,5-dien-1-yl)phenethyl cyclododecanecarboxylate (**29**)

Colorless oil, 83% yield. ^{1}H NMR (500 MHz, chloroform-*d*) δ 7.25 (d, *J* = 7.8 Hz, 2H), 7.12 (d, *J* = 7.8 Hz, 2H), 6.18 (dt, *J* = 15.7, 6.7 Hz, 1H), 5.01 (dd, *J* = 32.7, 13.6 Hz, 2H), 4.25 (t, *J* = 7.0 Hz, 2H), 2.88 (t, *J* = 7.0 Hz, 2H), 2.47 – 2.42 (m, 1H), 2.31 – 2.27 (m, 2H), 2.23 – 2.19 (m, 2H), 1.58 – 1.54 (m, 4H), 1.35 – 1.26 (m, 18H). ^{13}C NMR (126 MHz, chloroform-*d*) δ 176.5, 138.0, 136.6, 136.1, 129.9, 129.6, 129.0, 126.0, 114.9, 64.5, 40.3, 34.9, 33.6, 32.4, 26.6, 23.7, 23.5, 23.5, 23.4, 22.3. HRMS (ESI): [M+Na]$^{\oplus}$calcd for $C_{27}H_{40}O_2Na^{\oplus}$ 419.2921, found 419.2920.

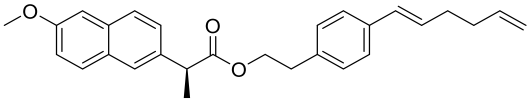

(*E*)-4-(Hexa-1,5-dien-1-yl)phenethyl (*S*)-2-(6-methoxynaphthalen-2-yl)propanoate (**9**)

White solid, 68% yield. ^{1}H NMR (500 MHz, chloroform-*d*) δ 7.67 (d, *J* = 8.6 Hz, 2H), 7.61 (s, 1H), 7.35 (dd, *J* = 8.5, 1.8 Hz, 1H), 7.16 – 7.07 (m, 4H), 6.94 (d, *J* = 7.8 Hz, 2H), 6.31 (d, *J* = 15.8 Hz, 1H), 6.13 (dt, *J* = 15.8, 6.7 Hz, 1H), 5.92 – 5.80 (m, 1H), 5.10 – 4.94 (m, 2H), 4.32 – 4.19 (m, 2H), 3.92 (s, 3H), 3.86 – 3.78 (m, 1H), 2.88 – 2.75 (m, 2H), 2.34 – 2.26 (m, 2H), 2.26 – 2.19 (m, 2H), 1.55 (d, *J* = 7.2 Hz, 3H). ^{13}C NMR (126 MHz, chloroform-*d*) δ 174.6, 157.7, 138.2, 136.5, 136.0, 135.7, 133.8, 129.9, 129.7, 129.4, 129.1, 129.0, 127.2, 126.3, 126.1, 126.0, 119.0, 115.0, 105.6, 65.3, 55.4, 45.6, 34.7, 33.7, 32.5, 18.4. HRMS (ESI): [M+Na]$^{\oplus}$calcd for $C_{28}H_{30}O_3Na^{\oplus}$ 437.2087, found 437.2087.

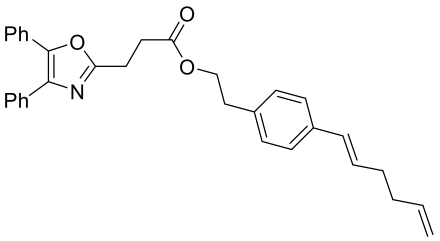

(*E*)-4-(Hexa-1,5-dien-1-yl)phenethyl (*S*)-2-(6-methoxynaphthalen-2-yl)propanoate (**30**)

Colorless oil, 60% yield. ^{1}H NMR (500 MHz, chloroform-*d*) δ 7.66 – 7.60 (m, 2H), 7.59 – 7.54 (m, 2H), 7.39 – 7.28 (m, 6H), 7.25 (d, *J* = 7.9 Hz, 2H), 7.12 (d, *J* = 7.8 Hz, 2H), 6.35 (d, *J* = 15.8 Hz, 1H), 6.17 (dt, *J* = 15.8, 6.7 Hz, 1H), 5.89 – 5.81 (m, 1H), 5.05 (dd, *J* = 17.3, 1.9 Hz, 1H), 4.99 (d, *J* = 10.1 Hz, 1H), 4.32 (t, *J* = 7.0 Hz, 2H), 3.16 (t, *J* = 7.5 Hz, 2H), 2.94 – 2.86 (m, 4H), 2.31 – 2.27 (m, 2H), 2.24 – 2.19 (m, 2H). ^{13}C NMR (126 MHz, chloroform-*d*) δ 172.0, 161.8, 154.4, 145.5, 138.2, 136.4, 136.2, 135.2, 132.5, 129.9, 129.1, 129.0, 128.7, 128.6, 128.5, 128.1, 127.9, 126.5, 126.1, 115.0, 65.3, 34.8, 33.6, 32.5, 31.2, 23.6. HRMS (ESI): [M+H]$^\oplus$calcd for $C_{32}H_{32}NO_3{}^\oplus$ 478.2377, found 478.2377.

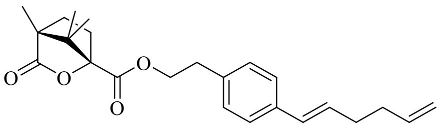

4-((*E*)-Hexa-1,5-dien-1-yl)phenethyl(1*S*,4*R*)-4,7,7-trimethyl-3-oxo-2-oxabicyclo[2.2.1]heptane-1-carboxylate (**31**)

Yellow solid, 63% yield. ^{1}H NMR (500 MHz, chloroform-*d*) δ 7.28 (d, *J* = 8.1 Hz, 2H), 7.15 (d, *J* = 7.8 Hz, 2H), 6.37 (d, *J* = 15.8 Hz, 1H), 6.20 (dt, *J* = 15.5, 6.7 Hz, 1H), 5.91 – 5.80 (m, 1H), 5.10 – 5.02 (m, 1H), 5.02 – 4.96 (m, 1H), 4.44 (t, *J* = 7.0 Hz, 2H), 2.98 (t, *J* = 7.0 Hz, 2H), 2.40 – 2.19 (m, 5H), 2.03 – 1.94 (m, 1H), 1.94 – 1.84 (m, 1H), 1.71 – 1.62 (m, 1H), 1.09 (s, 3H), 0.96 (s, 3H), 0.86 (s, 3H). ^{13}C NMR (126 MHz, chloroform-*d*) δ 178.2, 167.5, 138.1, 136.4, 135.7, 130.0, 129.8, 129.1, 126.2, 115.0, 91.2, 65.9, 54.8, 54.2, 34.7, 33.6, 32.5, 30.7, 29.0, 16.71, 16.66, 9.7. HRMS (ESI): [M+Na]$^\oplus$calcd for $C_{24}H_{30}O_4Na^\oplus$ 405.2036, found 405.2036.

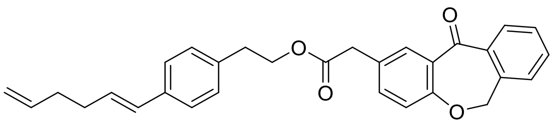

(*E*)-4-(Hexa-1,5-dien-1-yl)phenethyl 2-(11-oxo-6,11-dihydrodibenzo[*b*,*e*]oxepin-2-yl)acetate (**32**)

Colorless oil, 63% yield. ^{1}H NMR (500 MHz, chloroform-*d*) δ 8.10 (d, *J* = 2.4 Hz, 1H), 7.89 (d, *J* = 7.6 Hz, 1H), 7.56 – 7.49 (m, 1H), 7.48 – 7.41 (m, 1H), 7.36 – 7.31 (m, 2H), 7.23 (d, *J* = 7.9 Hz, 2H), 7.07 (d, *J* = 7.8 Hz, 2H), 6.98 (d, *J* = 8.4 Hz, 1H), 6.34 (d, *J* = 15.8 Hz, 1H), 6.17 (dt, *J* = 15.7, 6.7 Hz, 1H), 5.90 – 5.78 (m, 1H), 5.15 (s, 2H), 5.09 – 5.02 (m, 1H), 5.02 – 4.96 (m, 1H), 4.28 (t, *J* = 6.9 Hz, 2H), 3.60 (s, 2H), 2.88 (t, *J* = 6.9 Hz, 2H), 2.32 – 2.25 (m, 2H), 2.25 – 2.17 (m, 2H). ^{13}C NMR (126 MHz, chloroform-*d*) δ 190.7, 171.3, 160.4, 140.4, 138.1, 136.3, 136.3, 136.1, 135.6, 132.7, 132.5, 129.8, 129.7, 129.5, 129.2,

129.0, 127.8, 127.8, 126.1, 125.1, 121.0, 114.9, 73.6, 65.4, 40.2, 34.7, 33.6, 32.4. HRMS (ESI): [M+H]$^{\oplus}$ calcd for $C_{30}H_{29}O_4{}^{\oplus}$ 453.2060, found 453.2061.

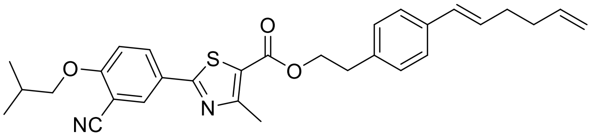

(*E*)-4-(Hexa-1,5-dien-1-yl)phenethyl2-(3-cyano-4-isobutoxyphenyl)-4-methylthiazole-5-carboxylate (**33**)

White solid, 43% yield. ^{1}H NMR (500 MHz, chloroform-*d*) δ 8.17 – 8.13 (m, 1H), 8.10 – 8.04 (m, 1H), 7.30 (d, *J* = 7.7 Hz, 2H), 7.19 (d, *J* = 7.7 Hz, 2H), 7.00 (d, *J* = 8.9 Hz, 1H), 6.38 (d, *J* = 15.8 Hz, 1H), 6.21 (dt, *J* = 15.4, 6.8 Hz, 1H), 5.92 – 5.79 (m, 1H), 5.13 – 4.94 (m, 2H), 4.48 (t, *J* = 6.8 Hz, 2H), 3.89 (d, *J* = 6.5 Hz, 2H), 3.02 (t, *J* = 6.8 Hz, 2H), 2.71 (s, 3H), 2.34 – 2.26 (m, 2H), 2.26 – 2.14 (m, 3H), 1.09 (d, *J* = 6.7 Hz, 6H). ^{13}C NMR (126 MHz, chloroform-*d*) δ 167.3, 162.5, 161.9, 161.3, 138.1, 136.3, 136.2, 132.6, 132.1, 129.9, 129.8, 129.1, 126.2, 126.0, 121.7, 115.4, 115.0, 112.6, 102.9, 75.7, 65.8, 34.8, 34.8, 33.6, 32.4, 28.2, 19.1, 17.5. HRMS (ESI): [M+H]$^{\oplus}$calcd for $C_{30}H33O_3N_2S^{\oplus}$ 501.2206, found 501.2208.

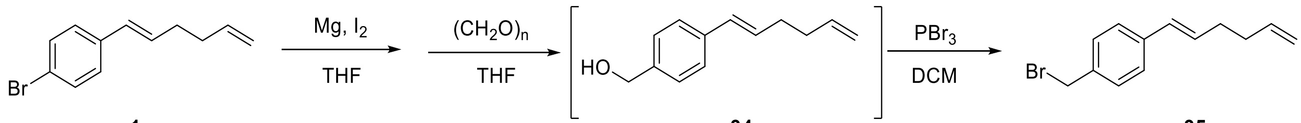

1 w 34 35

(*E*)-1-(Bromomethyl)-4-(hexa-1,5-dien-1-yl)benzene (**35**): To a 100 mL flask with a magnetic stirring bar were added Mg (0.48 g) activated by HCl and I_2 (25 mg). The reaction was stirred at 60 ^{o}C for 5 min. Then **1w** (2.4 g, 10 mmol) in dry THF (20 mL) was added to the solution and the resulting mixture was stirred at 60 ^{o}C for 1 h before $(CH_2O)_n$ (4.5 g, 15 mmol) was added to the reaction under nitrogen. After stirring at 60 ^{o}C for 5 h, the reaction was quenched by saturated aqueous NaCl solution (20 mL), exacted by ethyl acetate (20 mL × 3), condensed and purified by flash column chromatography to give the phenol product **34**. To a 100 mL flask with all the isolated **34** above and DCM (20 mL) was added PBr_3 (0.8 g, 3 mmol) dropwise at -20 ^{o}C. After stirring at -20 ^{o}C for 5 h, the reaction was condensed and purified by flash column chromatography to give pure compound **35** as a colorless oil in 27% yield. ^{1}H NMR (500 MHz, chloroform-*d*) δ 7.36 – 7.28 (m, 4H), 6.39 (d, *J* = 16.0 Hz, 1H), 6.25 (dt, *J* = 15.7, 6.7 Hz, 1H), 5.90 – 5.82 (m, 1H), 5.08 – 4.99 (m, 2H), 4.51 – 4.49 (m, 2H), 2.34 – 2.21 (m, 4H). ^{13}C NMR (126 MHz, chloroform-*d*) δ 131.2, 129.7, 129.4, 129.4, 128.9, 126.4, 120.1, 115.1, 33.8, 33.5, 32.5. HRMS (EI): [M]$^{\oplus}$calcd for $C_{13}H_{15}Br^{\oplus}$ 250.0352, found 250.0346.

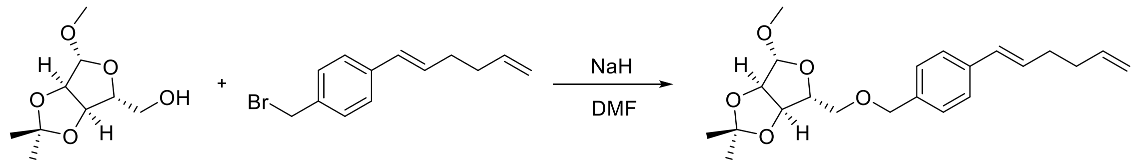

36 35 37

(3a*R*,4*R*,6*R*,6a*R*)-4-(((4-((*E*)-Hexa-1,5-dien-1-yl)benzyl)oxy)methyl)-6-methoxy-2,2-dimethyltetrahydrofuro[3,4-*d*][1,3]dioxole (**37**): To a 25 mL Schlenk tube with **36** (0.41 g, 2.0 mmol) and DMF (5 mL) was added NaH (48 mg, 2.0 mmol) under nitrogen. The reaction was stirred at room temperature for 1 h. Then **35** (0.50 g, 2.0 mmol) was added to the solution. The resulting mixture continued

to stir at room temperature for 6 h. After this time, the reaction was quenched by saturated NaCl solution (5 mL), exacted by ethyl acetate (20 mL × 3), condensed and purified by flash column chromatography to give diene compound **37** as a white solid in 61% yield. ^{1}H NMR (500 MHz, chloroform-*d*) δ 7.32 (d, *J* = 7.9 Hz, 2H), 7.26 (d, *J* = 7.9 Hz, 2H), 6.39 (d, *J* = 15.8 Hz, 1H), 6.23 (dt, *J* = 15.9, 6.7 Hz, 1H), 5.92 – 5.81 (m, 1H), 5.10 – 5.03 (m, 1H), 5.03 – 4.97 (m, 1H), 4.96 (s, 1H), 4.67 (d, *J* = 6.0 Hz, 1H), 4.56 (d, *J* = 6.1 Hz, 1H), 4.52 (d, *J* = 1.6 Hz, 2H), 4.39 – 4.33 (m, 1H), 3.53 – 3.47 (m, 1H), 3.47 – 3.40 (m, 1H), 3.29 (s, 3H), 2.35 – 2.27 (m, 2H), 2.27 – 2.19 (m, 2H), 1.48 (s, 3H), 1.31 (s, 3H). ^{13}C NMR (126 MHz, chloroform-*d*) δ138.2, 137.4, 136.7, 130.3, 129.9, 128.0, 126.1, 115.0, 112.5, 109.3, 85.2, 85.2, 82.2, 73.1, 71.0, 54.9, 33.6, 32.5, 26.5, 25.1. HRMS (EI): $[M]^{\oplus}$calcd for $C_{22}H_{30}O_5^{\oplus}$ 374.2097, found 374.2094.

3. Development of reaction conditions

3.1 Evaluation of chiral ligands

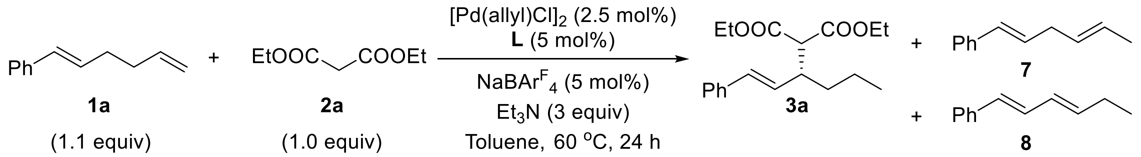

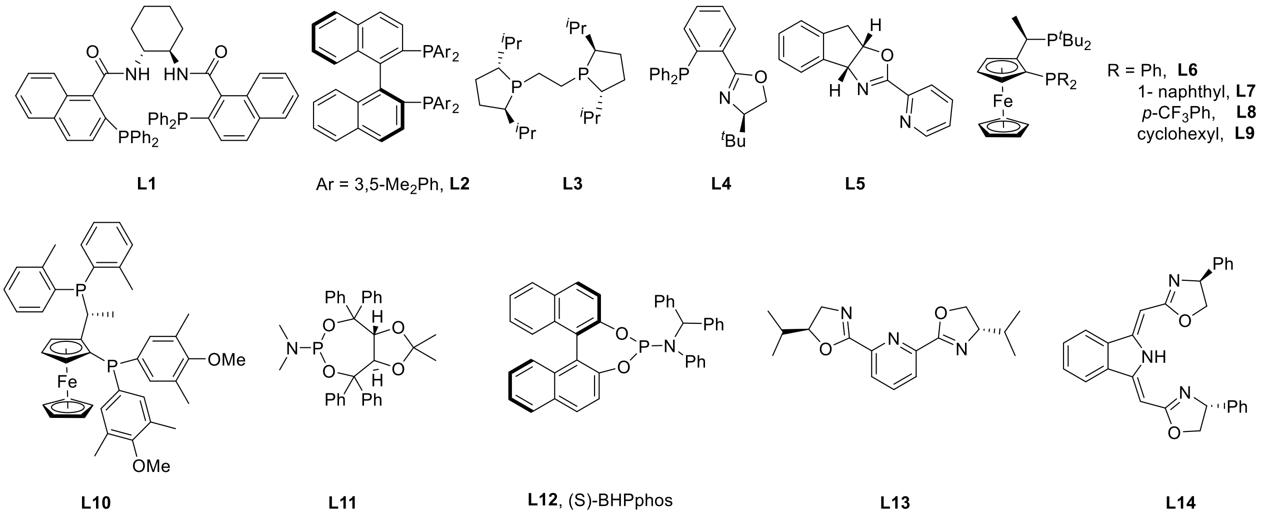

Entry	L	**7** (%)[a]	**8** (%)[a]	Yield (%)[a]	er[b]
1	**L1**	18	0	0	
2	**L2**	0	0	8	62:38
3	**L3**	22	2	0	
4	**L4**	22	2	0	
5	**L5**	0	2	0	

6	**L6**	14	14	23	95:5
7	**L7**	11	13	21	90:10
8	**L8**	7	3	21	97:3
9	**L9**	9	12	29	97:3
10	**L10**	7	18	10	44:56
11	**L11**	12	4	0	
12	**L12**	31	6	0	
13	**L13**	18	0	0	
14	**L14**	20	0	0	

Supplementary Fig. 1. [a]Determined by crude ^{1}H NMR. [b]Determined by chiral HPLC.

3.2 Evaluation of solvents and temperatures

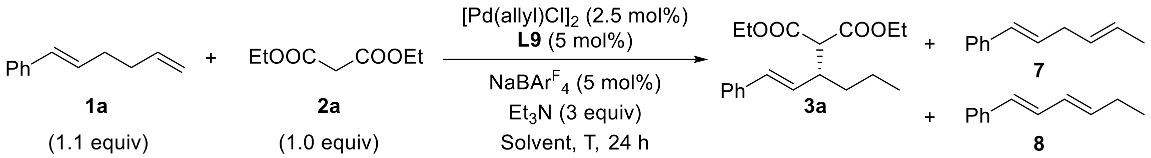

Entry	Solvent	T /°C	**7** (%)[a]	**8** (%)[a]	Yield (%)[a]	er[c]
1	DCM	60	4	9	Trace	74:26
2	THF	60	11	12	Trace	89:11
3	CH_3CN	60	0	0	0	
4	DMF	60	0	0	0	
5	Hexane	60	5	8	52	96.5:3.5
6	CPME	60	6	10	34	97:3
7	Et_3N	60	0	8	91 (82)[b]	97:3
8	Et_3N	40	4	2	76	96:4
9	Et_3N	80	7	24	60	94:6

Supplementary Fig. 2. [a]Determined by crude ^{1}H NMR. [b]Isolated yield. [c]Determined by chiral HPLC.

4. General procedure for Pd-catalyzed migratory allylic functionalization

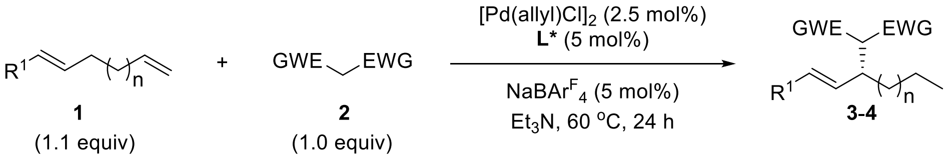

General procedure: To a 4 mL vial in the glovebox under nitrogen were added $[Pd(allyl)Cl]_2$ (1.8 mg, 0.0050 mmol), **L** (5.6 mg, 0.010 mmol), sodium tetrakis[3,5-bis(trifluoromethyl)phenyl]borate ($NaBAr^F_4$, 8.8 mg, 0.010 mmol) and dry Et_3N (0.2 mL). The mixture was stirred at room temperature for 5 min. Then the remote diene **1** (0.22 mmol) was added to the solution and the reaction continued to stir for 1 min. Finally, the nucleophile **2** (0.20 mmol) was added to the reaction and the resulting mixture was stirred at 60 ^oC for 24 h. After this time, the crude mixture was cooled to room temperature, condensed and crude ^1H NMR was obtained with dibromomethane (7 µL, 0.1 mmol) as internal standard to help determine the regioselectivity and conversion. The reaction was further purified by flash column chromatography to afford the pure allylation product **3-4**.

Notice: all the racemic products were prepared by using the racemic ligand **L6** under the standard catalytic conditions.

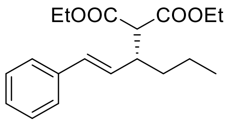

Diethyl (*R*,*E*)-2-(1-phenylhex-1-en-3-yl)malonate (**3a**)

Colorless oil, 82% yield, $[\alpha]_D^{25}$ +41.4 (*c* 2.1, $CHCl_3$) for 97:3 er; ^1H NMR (500 MHz, chloroform-*d*) δ 7.33 (d, *J* = 7.1 Hz, 2H), 7.28 (t, *J* = 7.7 Hz, 2H), 7.23 – 7.18 (m, 1H), 6.43 (d, *J* = 15.8 Hz, 1H), 6.04 (dd, *J* = 15.8, 9.7 Hz, 1H), 4.20 (q, *J* = 7.2 Hz, 2H), 4.16 – 4.08 (m, 2H), 3.41 (d, *J* = 8.8 Hz, 1H), 2.98 – 2.92 (m, 1H), 1.54 – 1.48 (m, 1H), 1.46 – 1.36 (m, 2H), 1.30 – 1.25 (m, 4H), 1.17 (t, *J* = 7.1 Hz, 3H), 0.89 (t, *J* = 7.1 Hz, 3H). ^{13}C NMR (126 MHz, chloroform-*d*) δ 168.4, 168.3, 137.1, 132.4, 129.9, 128.5, 127.3, 126.3, 61.4, 61.2, 57.3, 43.4, 35.1, 20.4, 14.2, 13.9 (one alkyl carbon signal was not observed because of overlapping). HRMS (EI): $[M]^{\oplus}$ calcd for $C_{19}H_{26}O_4^{\oplus}$ 318.1826, found 318.1830. HPLC analysis: Chiracel AD-H column; detected at 254 nm, 40 ^oC; 1% ^{i}PrOH in *n*-Hexane; flow = 0.7 mL/min; Retention time: 20.7 min (major), 25.2 min (minor).

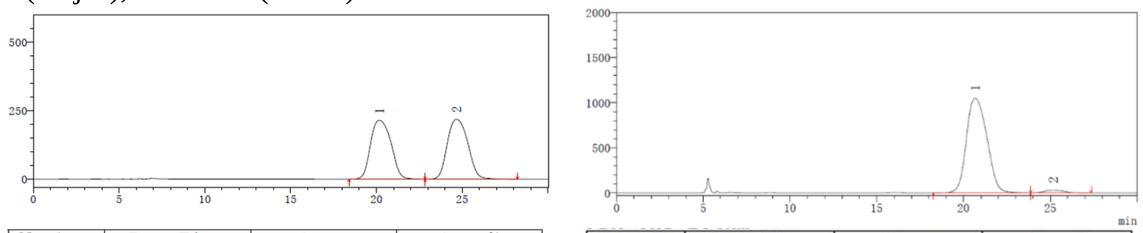

Number	Ret. Time	Area	Area%
1	20.172	17862056	49.35
2	24.672	18336084	50.65

Number	Ret. Time	Area	Area%
1	20.667	86643515	97.26
2	25.163	2438739	2.74

Supplementary Fig. 3. HPLC of **3a**.

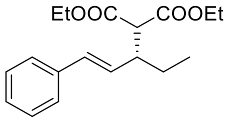

Diethyl (*R*,*E*)-2-(1-phenylpent-1-en-3-yl)malonate (**3b**)

Colorless oil, 76% yield, $[\alpha]_D^{25}$ +49.8 (*c* 1.6, $CHCl_3$) for 96:4 er; ^1H NMR (500 MHz, chloroform-*d*) δ 7.36 – 7.26 (m, 4H), 7.25 – 7.18 (m, 1H), 6.45 (d, *J* = 15.7 Hz, 1H), 6.03 (dd, *J* = 15.7, 9.6 Hz, 1H), 4.23

– 4.17 (m, 2H), 4.15 – 4.07 (m, 2H), 3.43 (d, J = 8.8 Hz, 1H), 2.89 – 2.82 (m, 1H), 1.68 – 1.58 (m, 1H), 1.48 – 1.37 (m, 1H), 1.26 (t, J = 7.1 Hz, 3H), 1.17 (t, J = 7.1 Hz, 3H), 0.92 (t, J = 7.4 Hz, 3H). ^{13}C NMR (126 MHz, chloroform-*d*) δ 168.5, 168.3, 137.1, 132.7, 129.6, 128.5, 127.4, 126.3, 61.4, 61.2, 57.1, 45.3, 25.9, 14.2, 11.8 (one alkyl carbon signal was not observed because of overlapping). HRMS (ESI): [M+Na]$^{\oplus}$ calcd for $C_{18}H_{24}O_4Na^{\oplus}$ 327.1567, found 327.1567. HPLC analysis: Chiracel (AD-H)+(AD-H) column; detected at 254 nm, 20 °C; 1% ^{i}PrOH in *n*-Hexane; flow = 0.7 mL/min; Retention time: 15.6 min (major), 19.1 min (minor).

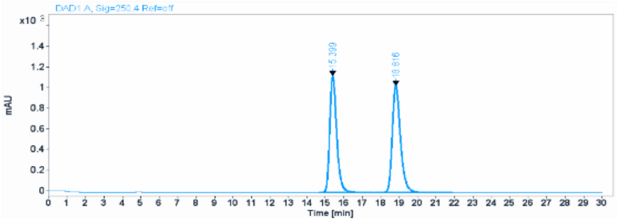

Signal: DAD1 A, Sig=250,4 Ref=off

RT [min]	Width [min]	Area [mAU*S]	Height [mAU]	Area%	ee value%
15.399	0.400	29476.553	1130.344	48.1392	RAC
18.816	0.470	31755.301	1035.523	51.8608	

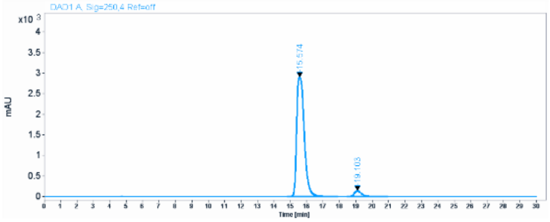

Signal: DAD1 A, Sig=250,4 Ref=off

RT [min]	Width [min]	Area [mAU*S]	Height [mAU]	Area%	ee value%
15.574	0.500	92522.523	2901.923	95.6985	91.397
19.103	0.466	4158.726	136.144	4.3015	

Supplementary Fig. 4. HPLC of **3b**.

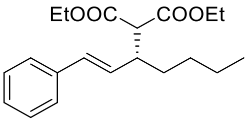

Diethyl (*R*,*E*)-2-(1-phenylhept-1-en-3-yl)malonate (**3c**)

Colorless oil, 72% yield, $[\alpha]_D^{25}$ +36.8 (*c* 1.7, $CHCl_3$) for 97:3 er; ^{1}H NMR (500 MHz, chloroform-*d*) δ 7.36 – 7.27 (m, 4H), 7.23 – 7.18 (m, 1H), 6.43 (d, J = 15.8 Hz, 1H), 6.04 (dd, J = 15.7, 9.6 Hz, 1H), 4.24 – 4.17 (m, 2H), 4.15 – 4.08 (m, 2H), 3.41 (d, J = 8.9 Hz, 1H), 2.96 – 2.89 (m, 1H), 1.59 – 1.50 (m, 1H), 1.44 – 1.37 (m, 1H), 1.36 – 1.30 (m, 2H), 1.29 – 1.23 (m, 5H), 1.17 (t, J = 7.1 Hz, 3H), 0.86 (t, J = 6.4 Hz, 3H). ^{13}C NMR (126 MHz, chloroform-*d*) δ 168.5, 168.3, 137.2, 132.4, 130.0, 128.5, 127.4, 126.3, 61.4, 61.3, 57.4, 43.6, 32.7, 29.4, 22.5, 14.2, 14.0 (one alkyl carbon signal was not observed because of overlapping). HRMS (EI): [M]$^{\oplus}$ calcd for $C_{20}H_{28}O_4^{\oplus}$ 332.1982, found 332.1984. HPLC analysis: Chiralpak IE column; detected at 254 nm, 30 °C; 10% ^{i}PrOH in *n*-Hexane; flow = 0.7 mL/min; Retention time: 7.6 min (minor), 8.0 min (major).

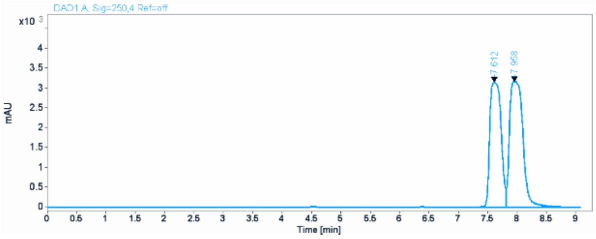

Signal: DAD1 A, Sig=250,4 Ref=off

RT [min]	Width [min]	Area [mAU*S]	Height [mAU]	Area%	ee value%
7.612	0.214	41761.078	3150.719	45.3169	RAC
7.958	0.255	50392.266	3169.843	54.6831	

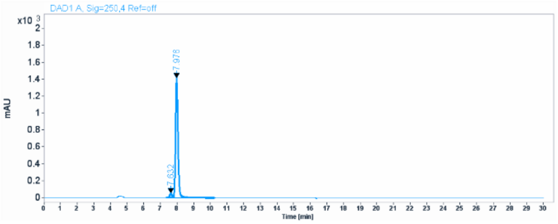

Signal: DAD1 A, Sig=250,4 Ref=off

RT [min]	Width [min]	Area [mAU*S]	Height [mAU]	Area%	ee value%
7.632	0.143	457.090	49.850	3.0410	93.918
7.976	0.157	14573.830	1407.607	96.9590	

Supplementary Fig. 5. HPLC of **3c**.

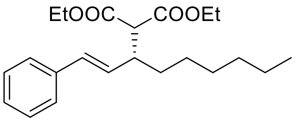

Diethyl (*R*,*E*)-2-(1-phenylnon-1-en-3-yl)malonate (**3d**)

Colorless oil, 72% yield, $[\alpha]_D^{25}$ +28.1 (*c* 1.6, $CHCl_3$) for 97:3 er; ^{1}H NMR (500 MHz, chloroform-*d*) δ 7.39 – 7.34 (m, 2H), 7.34 – 7.29 (m, 2H), 7.27 – 7.21 (m, 1H), 6.46 (d, *J* = 15.8 Hz, 1H), 6.06 (dd, *J* = 15.8, 9.7 Hz, 1H), 4.26 – 4.21 (m, 2H), 4.18 – 4.10 (m, 2H), 3.45 (s, 1H), 2.99 – 2.93 (m, 1H), 1.61 – 1.51 (m, 1H), 1.48 – 1.25 (m, 12H), 1.20 (t, *J* = 7.1 Hz, 3H), 0.89 (t, *J* = 6.9 Hz, 3H). ^{13}C NMR (126 MHz, chloroform-*d*) δ 168.5, 168.3, 137.2, 132.4, 129.9, 128.5, 127.3, 126.3, 61.4, 61.2, 57.4, 43.7, 32.9, 31.8, 29.1, 27.2, 22.7, 14.2, 14.1 (one alkyl carbon signal was not observed because of overlapping). HRMS (EI): $[M]^{\oplus}$ calcd for $C_{22}H_{32}O_4^{\oplus}$ 360.2295, found 360.2298. HPLC analysis: Chiracel AD-H column; detected at 254 nm, 40 ^{o}C; 1% ^{i}PrOH in *n*-Hexane; flow = 0.7 mL/min; Retention time: 28.7 min (major), 30.0 min (minor).

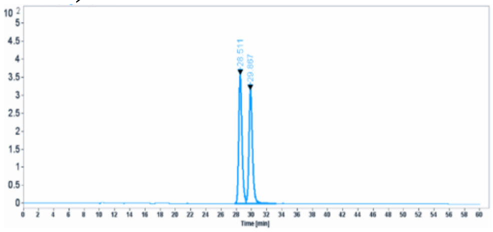

RT [min]	Width [min]	Area [mAU*S]	Height [mAU]	Area%	ee value%
28.511	0.450	10522.397	356.638	51.2824	2.565
29.867	0.480	9996.132	314.837	48.7176	

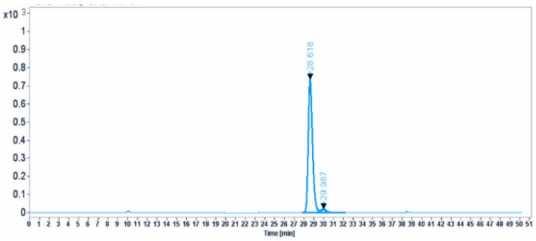

RT [min]	Width [min]	Area [mAU*S]	Height [mAU]	Area%	ee value%
28.618	0.451	22038.631	735.772	96.7059	93.412
29.987	0.552	750.710	19.822	3.2941	

Supplementary Fig. 6. HPLC of **3d**.

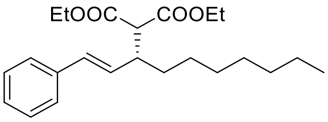

Diethyl (*R*,*E*)-2-(1-phenyldec-1-en-3-yl)malonate (**3e**)

Colorless oil, 62% yield, $[\alpha]_D^{25}$ +26.3 (*c* 1.7, $CHCl_3$) for 98:2 er; ^1H NMR (500 MHz, chloroform-*d*) δ 7.35 – 7.32 (m, 2H), 7.31 – 7.26 (m, 2H), 7.24 – 7.16 (m, 1H), 6.43 (d, *J* = 15.8 Hz, 1H), 6.03 (dd, 1H), 4.23 – 4.17 (m, 2H), 4.15 – 4.07 (m, 2H), 3.41 (d, *J* = 8.8 Hz, 1H), 2.96 – 2.90 (m, 1H), 1.59 – 1.49 (m, 1H), 1.45 – 1.20 (m, 14H), 1.17 (t, *J* = 7.1 Hz, 3H), 0.86 (t, *J* = 6.9 Hz, 3H). ^{13}C NMR (126 MHz, $CDCl_3$) δ 168.5, 168.3, 137.2, 132.4, 130.0, 128.5, 127.3, 126.3, 61.4, 61.2, 57.4, 43.7, 32.9, 31.9, 29.4, 29.2, 27.2, 22.7, 14.2, 14.1 (one alkyl carbon signal was not observed because of overlapping). HRMS (EI): $[M]^{\oplus}$ calcd for $C_{23}H_{34}O_4^{\oplus}$ 374.2452, found 374.2458. HPLC analysis: Chiralpak IE column; detected at 254 nm, 40 °C; 10% ^{i}PrOH in *n*-Hexane; flow = 0.7 mL/min; Retention time: 6.8 min (minor), 7.1 min (major).

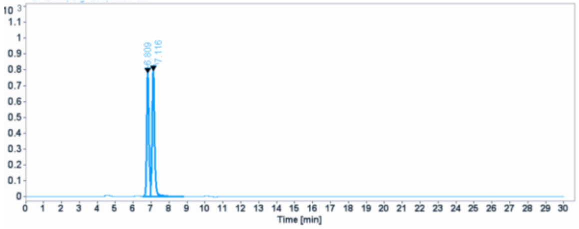

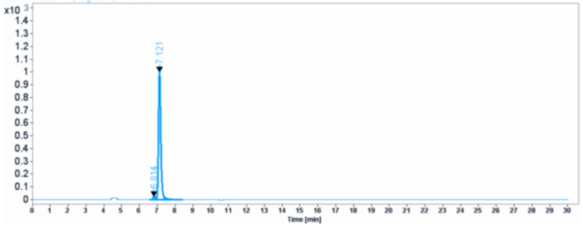

RT [min]	Width [min]	Area [mAU*S]	Height [mAU]	Area%	ee value%
6.809	0.131	6575.290	778.776	47.1274	RAC
7.116	0.140	7376.858	794.327	52.8726	

RT [min]	Width [min]	Area [mAU*S]	Height [mAU]	Area%	ee value%
6.814	0.127	196.704	23.780	2.0865	95.827
7.121	0.141	9230.714	999.971	97.9135	

Supplementary Fig. 7. HPLC of **3e**.

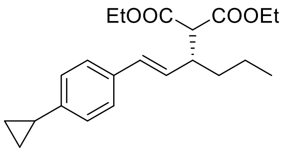

Diethyl (*R*,*E*)-2-(1-(4-cyclopropylphenyl)hex-1-en-3-yl)malonate (3f)

Colorless oil, 91% yield, $[\alpha]_D^{25}$ +40.6 (*c* 2.5, $CHCl_3$) for 97:3 er; ^1H NMR (500 MHz, chloroform-*d*) δ 7.25 – 7.19 (m, 2H), 7.01 – 6.95 (m, 2H), 6.39 (d, *J* = 15.7 Hz, 1H), 5.95 (dd, *J* = 15.7, 9.7 Hz, 1H), 4.23 – 4.17 (m, 2H), 4.14 – 4.07 (m, 2H), 3.39 (d, *J* = 9.0 Hz, 1H), 2.95 – 2.89 (m, 1H), 1.89 – 1.83 (m, 1H), 1.54 – 1.45 (m, 1H), 1.45 – 1.33 (m, 2H), 1.26 (t, *J* = 7.1 Hz, 4H), 1.17 (t, *J* = 7.1 Hz, 3H), 0.98 – 0.90 (m, 2H), 0.88 (t, *J* = 7.2 Hz, 3H), 0.69 – 0.64 (m, 2H). ^{13}C NMR (126 MHz, $CDCl_3$) δ 168.5, 168.3, 143.3, 134.4, 132.2, 128.7, 126.2, 125.8, 61.4, 61.2, 57.5, 43.5, 35.1, 20.4, 15.3, 14.21, 14.20, 13.9, 9.3 (one alkyl carbon signal was not observed because of overlapping). HRMS (EI): $[M]^{\oplus}$ calcd for $C_{22}H_{30}O_4^{\oplus}$ 358.2139, found 358.2142. HPLC analysis: Chiracel AD-H column; detected at 254 nm, 40 °C; 2% ^{i}PrOH in *n*-Hexane; flow = 0.7 mL/min; Retention time: 14.9 min (major), 16.1 min (minor).

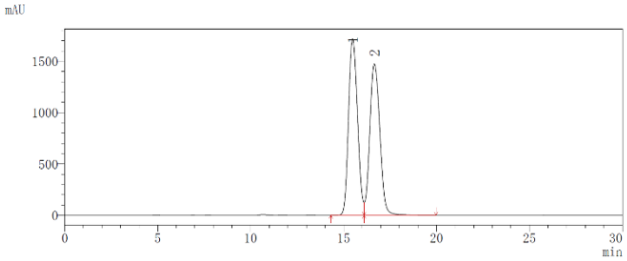

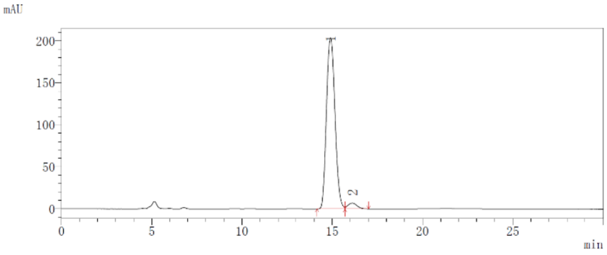

Number	Ret. Time	Area	Area%
1	15.487	57324029	50.80
2	16.657	55523754	49.20

Number	Ret. Time	Area	Area%
1	14.904	6690942	96.49
2	16.110	243590	3.51

Supplementary Fig. 8. HPLC of **3f**.

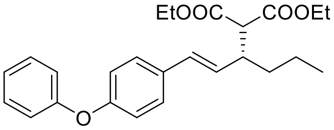

Diethyl (*R*,*E*)-2-(1-(4-phenoxyphenyl)hex-1-en-3-yl)malonate (**3g**)

Colorless oil, 74% yield, $[\alpha]_D^{25}$ +31.9 (*c* 2.4, $CHCl_3$) for 96:4 er; ^1H NMR (500 MHz, chloroform-*d*) δ 7.37 – 7.27 (m, 4H), 7.13 – 7.05 (m, 1H), 7.03 – 6.90 (m, 4H), 6.40 (d, *J* = 15.6 Hz, 1H), 6.03 – 5.86 (m, 1H), 4.26 – 4.01 (m, 4H), 3.48 – 3.33 (m, 1H), 3.00 – 2.84 (m, 1H), 1.56 – 1.46 (m, 1H), 1.45 – 1.34 (m, 2H), 1.30 – 1.23 (m, 4H), 1.18 (t, *J* = 7.3 Hz, 3H), 0.93 – 0.85 (m, 3H). ^{13}C NMR (126 MHz, $CDCl_3$) δ 168.5, 168.3, 157.3, 156.6, 132.5, 131.6, 129.8, 129.0, 127.6, 123.3, 119.0, 118.8, 61.4, 61.2, 57.4, 43.4, 35.1, 20.4, 14.2, 13.9 (one alkyl carbon signal was not observed because of overlapping). HRMS (ESI): $[M+Na]^{\oplus}$ calcd for $C_{25}H_{30}O_5Na^{\oplus}$ 433.1985, found 433.1984. HPLC analysis: Chiralpak IE column; detected at 254 nm, 40 °C; 1% ^{i}PrOH in *n*-Hexane; flow = 0.7 mL/min; Retention time: 19.4 min (minor), 21.3 min (major).

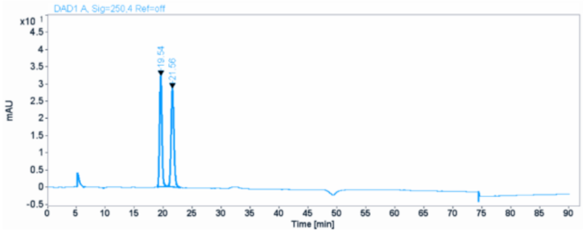

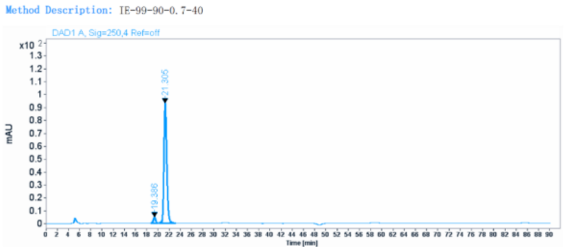

Signal: DAD1 A, Sig=250,4 Ref=off

RT [min]	Width [min]	Area [mAU*S]	Height [mAU]	Area%	ee value%
19.540	0.450	916.633	32.247	48.6269	RAC
21.560	0.524	968.399	28.563	51.3731	

Signal: DAD1 A, Sig=250,4 Ref=off

RT [min]	Width [min]	Area [mAU*S]	Height [mAU]	Area%	ee value%
19.386	0.448	122.460	4.272	3.6348	92.730
21.305	0.537	3246.673	92.548	96.3652	

Supplementary Fig. 9. HPLC of **3g**.

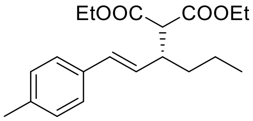

Diethyl (*R*,*E*)-2-(1-(*p*-tolyl)hex-1-en-3-yl)malonate (**3h**)

Colorless oil, 76% yield, $[\alpha]_D^{25}$ +41.5 (*c* 1.9, $CHCl_3$) for 96:4 er; ^1H NMR (500 MHz, chloroform-*d*) δ 7.23 (d, *J* = 7.9 Hz, 2H), 7.09 (d, *J* = 7.8 Hz, 2H), 6.40 (d, *J* = 15.8 Hz, 1H), 5.97 (dd, *J* = 15.7, 9.7 Hz, 1H), 4.23 – 4.17 (m, 2H), 4.14 – 4.06 (m, 2H), 3.40 (d, *J* = 9.0 Hz, 1H), 2.96 – 2.90 (m, 1H), 2.31 (s, 3H), 1.55 – 1.45 (m, 1H), 1.45 – 1.34 (m, 2H), 1.29 – 1.25 (m, 4H), 1.17 (t, *J* = 7.1 Hz, 3H), 0.89 (t, *J* = 7.2 Hz, 3H). ^{13}C NMR (126 MHz, chloroform-*d*) δ168.5, 168.3, 137.1, 134.4, 132.3, 129.2, 128.8, 126.2, 61.4, 61.2, 57.4, 43.4, 35.1, 21.2, 20.4, 14.2, 13.9 (one alkyl carbon signal was not observed because of

overlapping). HRMS (EI): [M]$^{\oplus}$ calcd for $C_{20}H_{28}O_4^{\oplus}$ 332.1982, found 332.1990. HPLC analysis: Chiracel AD-H column; detected at 254 nm, 40 °C; 2% ^{i}PrOH in *n*-Hexane; flow = 0.7 mL/min; Retention time: 11.6 min (major), 13.7 min (minor).

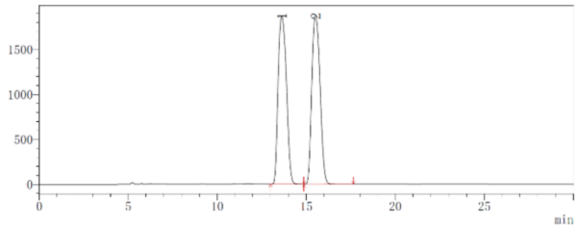

Number	Ret. Time	Area	Area%
1	13.621	60815591	49.96
2	15.520	60921090	50.04

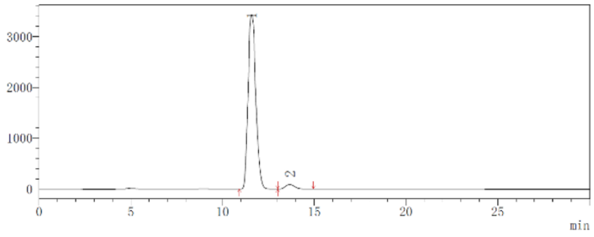

Number	Ret. Time	Area	Area%
1	11.581	100333675	97.09
2	13.665	3002535	2.91

Supplementary Fig. 10. HPLC of **3h**.

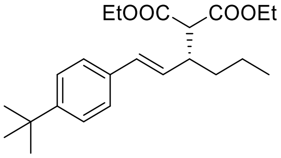

Diethyl (*R*,*E*)-2-(1-(4-(*tert*-butyl)phenyl)hex-1-en-3-yl)malonate (**3i**)

Colorless oil, 79% yield, $[\alpha]_D^{25}$ +37.0 (*c* 2.6, $CHCl_3$) for 97:3 er; ^{1}H NMR (500 MHz, chloroform-*d*) δ 7.35 (d, *J* = 8.5 Hz, 2H), 7.31 (d, *J* = 8.3 Hz, 2H), 6.45 (d, *J* = 15.7 Hz, 1H), 6.02 (dd, *J* = 15.7, 9.7 Hz, 1H), 4.26 – 4.20 (m, 2H), 4.19 – 4.11 (m, 2H), 3.44 (d, *J* = 8.9 Hz, 1H), 3.00 – 2.94 (m, 1H), 1.57 – 1.48 (m, 1H), 1.48 – 1.41 (m, 2H), 1.34 (s, 9H), 1.33 – 1.27 (m, 4H), 1.22 (t, *J* = 7.1 Hz, 3H), 0.91 (t, *J* = 7.1 Hz, 3H). ^{13}C NMR (126 MHz, $CDCl_3$) δ 168.5, 168.3, 150.4, 134.4, 132.2, 129.1, 126.0, 125.4, 61.4, 61.2, 57.4, 43.4, 35.1, 34.6, 31.3, 20.4, 14.21, 14.19, 13.9. HRMS (ESI): [M+Na]$^{\oplus}$ calcd for $C_{23}H_{34}O_4Na^{\oplus}$ 397.2349, found 397.2347. HPLC analysis: Chiracel (AD-H)+(AD-H) column; detected at 254 nm, 20 °C; 1% ^{i}PrOH in *n*-Hexane; flow = 0.7 mL/min; Retention time: 22.8 min (major), 24.3 min (minor).

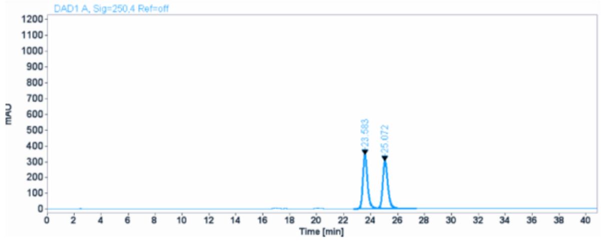

Signal: DAD1 A, Sig=250,4 Ref=off

RT [min]	Width [min]	Area [mAU*S]	Height [mAU]	Area%	ee value%
23.583	0.360	8058.676	340.258	50.6061	RAC
25.072	0.399	7865.625	300.618	49.3939	

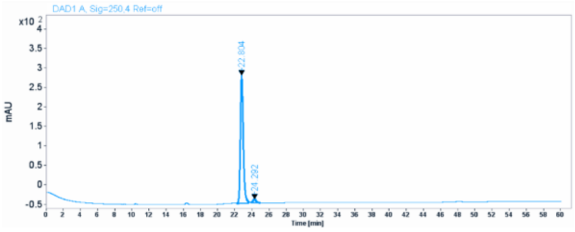

Signal: DAD1 A, Sig=250,4 Ref=off

RT [min]	Width [min]	Area [mAU*S]	Height [mAU]	Area%	ee value%
22.804	0.391	7659.245	326.761	97.4356	94.871
24.292	0.353	201.581	8.738	2.5644	

Supplementary Fig. 11. HPLC of **3i**.

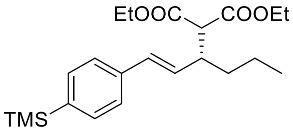

Diethyl (*R*,*E*)-2-(1-(4-(trimethylsilyl)phenyl)hex-1-en-3-yl)malonate (3j)

Colorless oil, 82% yield, $[\alpha]_D^{25}$ +28.0 (*c* 3.1, $CHCl_3$) for 96:4 er; ^{1}H NMR (500 MHz, chloroform-*d*) δ 7.48 – 7.42 (m, 2H), 7.32 (d, *J* = 8.0 Hz, 2H), 6.43 (d, *J* = 15.7 Hz, 1H), 6.06 (dd, *J* = 15.8, 9.7 Hz, 1H), 4.24 – 4.18 (m, 2H), 4.15 – 4.07 (m, 2H), 3.41 (d, *J* = 8.8 Hz, 1H), 2.98 – 2.92 (m, 1H), 1.56 – 1.46 (m, 1H), 1.46 – 1.34 (m, 2H), 1.27 (t, *J* = 7.1 Hz, 4H), 1.19 (t, *J* = 7.1 Hz, 3H), 0.89 (t, *J* = 7.2 Hz, 3H), 0.25 (s, 9H). ^{13}C NMR (126 MHz, $CDCl_3$) δ 168.4, 168.3, 139.6, 137.6, 133.6, 132.5, 130.2, 125.6, 61.4, 61.2, 57.4, 43.4, 35.1, 20.4, 14.2, 14.2, 13.9, -1.1. HRMS (EI): $[M]^{\oplus}$ calcd for $C_{22}H_{34}O_4Si^{\oplus}$ 390.2221, found 390.2219. HPLC analysis: Chiralpak IE column; detected at 254 nm, 40 °C; 2% ^{i}PrOH in *n*-Hexane; flow = 0.7 mL/min; Retention time: 9.8 min (major), 11.5 min (minor).

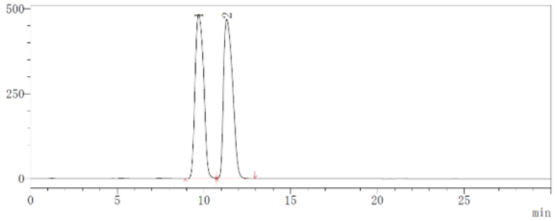

Number	Ret. Time	Area	Area%
1	9.695	16517173	49.38
2	11.311	16932502	50.62

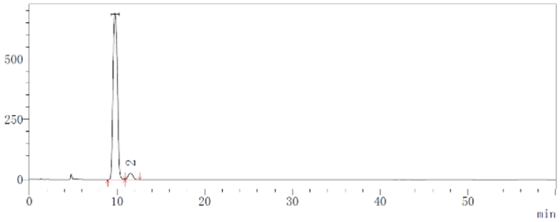

Number	Ret. Time	Area	Area%
1	9.756	24062260	96.07
2	11.517	983613	3.93

Supplementary Fig. 12. HPLC of **3j**.

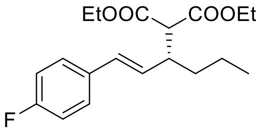

Diethyl (*R*,*E*)-2-(1-(4-fluorophenyl)hex-1-en-3-yl)malonate (3k)

Colorless oil, 89% yield, $[\alpha]_D^{25}$ +37.3 (*c* 1.3, $CHCl_3$) for 95:5 er; ^{1}H NMR (500 MHz, chloroform-*d*) δ 7.32 – 7.28 (m, 2H), 6.99 – 6.95 (m, 2H), 6.44 – 6.36 (m, 1H), 5.96 (dd, *J* = 15.9, 9.5 Hz, 1H), 4.24 – 4.18 (m, 2H), 4.16 – 4.07 (m, 2H), 3.41 (d, *J* = 8.6 Hz, 1H), 2.96 – 2.90 (m, 1H), 1.56 – 1.47 (m, 1H), 1.46 – 1.36 (m, 2H), 1.29 – 1.24 (m, 4H), 1.17 (t, *J* = 7.1 Hz, 3H), 0.90 (t, *J* = 7.1 Hz, 3H). ^{113}C NMR (126 MHz, $CDCl_3$) δ 168.4, 168.3, 162.2 (d, *J* = 245.6 Hz), 133.3 (d, *J* = 4.1 Hz), 131.2, 129.6 (d, *J* = 2.7 Hz), 127.7 (d, *J* = 8.4 Hz), 115.4 (d, *J* = 21.5 Hz), 61.4, 61.2, 57.3, 43.4, 35.0, 20.4, 14.2, 13.9. ^{19}F NMR (376 MHz, Chloroform-*d*) δ -115.03 (s). HRMS (ESI): $[M+H]^{\oplus}$ calcd for $C_{19}H_{26}O_4F^{\oplus}$ 337.1810, found 337.1808. HPLC analysis: Chiracel AD-H column; detected at 254 nm, 40 °C; 5% ^{i}PrOH in *n*-Hexane; flow = 0.7 mL/min; Retention time: 9.4 min (major), 10.5 min (minor).

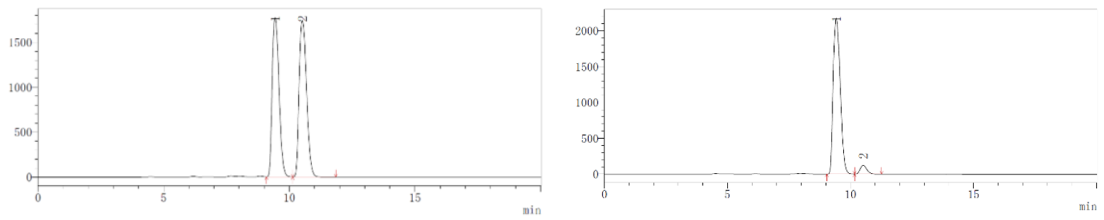

Number	Ret. Time	Area	Area%
1	9.436	33541207	48.49
2	10.518	35627030	51.51

Number	Ret. Time	Area	Area%
1	9.419	43513232	94.97
2	10.516	2306420	5.03

Supplementary Fig. 13. HPLC of **3k**.

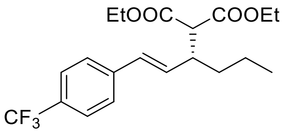

Diethyl (*R*,*E*)-2-(1-(4-(trifluoromethyl)phenyl)hex-1-en-3-yl)malonate (3l)

Colorless oil, 83% yield, $[\alpha]_D^{25}$ +29.5 (*c* 2.4, $CHCl_3$) for 93:7 er; ^{1}H NMR (500 MHz, chloroform-*d*) δ 7.54 (d, *J* = 8.0 Hz, 2H), 7.43 (d, *J* = 8.0 Hz, 2H), 6.47 (d, *J* = 15.8 Hz, 1H), 6.18 (dd, *J* = 15.8, 9.6 Hz, 1H), 4.24 – 4.18 (m, 2H), 4.16 – 4.09 (m, 2H), 3.44 (d, *J* = 8.6 Hz, 1H), 3.01 – 2.94 (m, 1H), 1.59 – 1.38 (m, 3H), 1.29 – 1.25 (m, 4H), 1.18 (t, *J* = 7.1 Hz, 3H), 0.90 (t, *J* = 7.2 Hz, 3H). ^{13}C NMR (126 MHz, $CDCl_3$) δ 168.31, 168.21, 140.61, 132.84, 131.15, 129.22 (q, *J* = 32.6 Hz), 126.42, 125.52 (q, *J* = 4.1 Hz), 124.3 (q, *J* = 270.9 Hz), 61.50, 61.32, 57.08, 43.38, 34.94, 20.47, 14.19, 14.17, 13.88. ^{19}F NMR (376 MHz, Chloroform-*d*) δ -62.55. HRMS (ESI): $[M+H]^{\oplus}$ calcd for $C_{20}H_{26}O_4F_3^{\oplus}$ 387.1778, found 387.1776. HPLC analysis: Chiracel AD-H column; detected at 254 nm, 40 °C; 10% ^{i}PrOH in *n*-Hexane; flow = 0.7 mL/min; Retention time: 8.1 min (minor), 8.7 min (major).

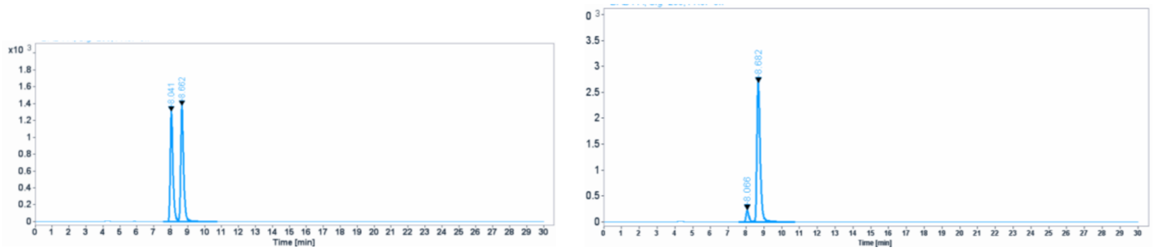

RT [min]	Width [min]	Area [mAU*S]	Height [mAU]	Area%
8.041	0.164	14353.434	1305.799	47.3429
8.662	0.175	15964.592	1374.682	52.6571

RT [min]	Width [min]	Area [mAU*S]	Height [mAU]	Area%
8.066	0.166	2444.357	219.890	6.6257
8.682	0.196	34447.648	2678.006	93.3743

Supplementary Fig. 14. HPLC of **3l**.

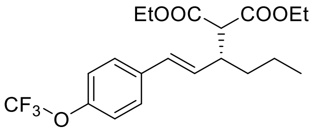

Diethyl (*R*,*E*)-2-(1-(4-(trifluoromethoxy)phenyl)hex-1-en-3-yl)malonate (**3m**)

Colorless oil, 63% yield, $[\alpha]_D^{25}$ +32.0 (*c* 2.5, $CHCl_3$) for 96:4 er; ^1H NMR (500 MHz, chloroform-*d*) δ 7.39 – 7.31 (m, 2H), 7.13 (d, *J* = 8.3 Hz, 2H), 6.42 (d, *J* = 15.7 Hz, 1H), 6.05 (dd, *J* = 15.7, 9.6 Hz, 1H), 4.24 – 4.18 (m, 2H), 4.17 – 4.09 (m, 2H), 3.42 (d, *J* = 8.6 Hz, 1H), 2.98 – 2.92 (m, 1H), 1.56 – 1.47 (m, 1H), 1.46 – 1.34 (m, 2H), 1.27 (t, *J* = 7.1 Hz, 4H), 1.18 (t, *J* = 7.1 Hz, 3H), 0.90 (t, *J* = 7.2 Hz, 3H). ^{13}C NMR (126 MHz, $CDCl_3$) δ 168.4, 168.3, 148.4, 135.9, 131.1, 131.0, 127.4, 121.1, 61.5, 61.3, 57.2, 43.3, 35.0, 20.5, 14.2, 13.9 (one alkyl carbon signal was not observed because of overlapping. The carbon signal of CF_3 was also not observed). ^{19}F NMR (376 MHz, $CDCl_3$) δ -57.97. HRMS (EI): $[M]^{\oplus}$ calcd for $C_{20}H_{25}O_5F_3^{\oplus}$ 402.1649, found 402.1651. HPLC analysis: Chiralpak IG column; detected at 254 nm, 40 ^oC; 5% ^{i}PrOH in *n*-Hexane; flow = 0.7 mL/min; Retention time: 8.4 min (major), 9.3 min (minor).

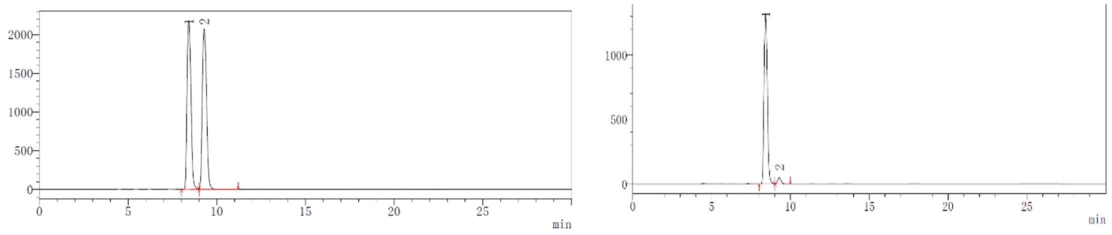

Number	Ret. Time	Area	Area%
1	8.428	33732142	49.58
2	9.293	34299246	50.42

Number	Ret. Time	Area	Area%
1	8.419	19712236	96.31
2	9.291	754589	3.69

Supplementary Fig. 15. HPLC of **3m**.

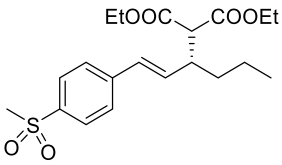

Diethyl (*R*,*E*)-2-(1-(4-(methylsulfonyl)phenyl)hex-1-en-3-yl)malonate (**3n**)

Colorless oil, 48% yield, $[\alpha]_D^{25}$ +33.9 (*c* 1.2, $CHCl_3$) for 97:3 er; ^1H NMR (400 MHz, chloroform-*d*) δ 7.90 – 7.82 (m, 2H), 7.55 – 7.47 (m, 2H), 6.50 (d, *J* = 15.8 Hz, 1H), 6.28 (dd, *J* = 15.8, 9.6 Hz, 1H), 4.21 (q, *J* = 7.2 Hz, 2H), 4.13 (qd, *J* = 7.1, 3.1 Hz, 2H), 3.45 (d, *J* = 8.4 Hz, 1H), 3.05 (s, 3H), 3.02 – 2.95 (m, 1H), 1.60 – 1.31 (m, 4H), 1.27 (t, *J* = 7.1 Hz, 3H), 1.19 (t, *J* = 7.1 Hz, 3H), 0.91 (t, *J* = 7.2 Hz, 3H). ^{13}C NMR (101 MHz, chloroform-*d*) δ 168.2, 168.1, 142.5, 138.8, 134.5, 130.7, 127.7, 126.9, 61.5, 61.3, 56.9, 44.6, 43.3, 34.8, 20.4, 14.2, 14.1, 13.8. HRMS (EI): $[M]^{\oplus}$ calcd for $C_{20}H_{28}O_6S^{\oplus}$ 396.1601, found 396.1606. HPLC analysis: Chiralpak IA column; detected at 254 nm, 40 ^oC; 15% ^{i}PrOH in *n*-Hexane; flow = 0.7 mL/min; Retention time:17.3 min (minor), 19.6 min (major).

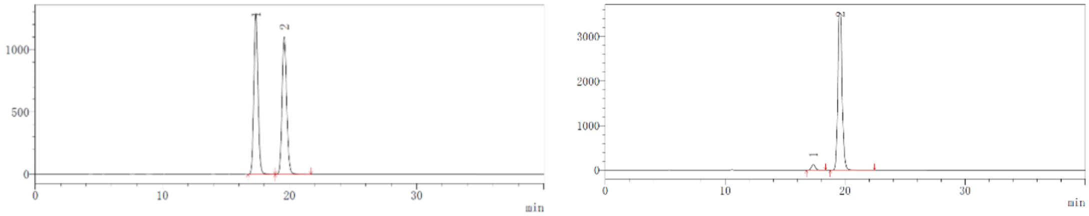

Number	Ret. Time	Area	Area%
1	17.352	29174655	51.16
2	19.581	27846616	48.84

Number	Ret. Time	Area	Area%
1	17.345	2920700	3.18
2	19.579	88979602	96.82

Supplementary Fig. 16. HPLC of **3n**.

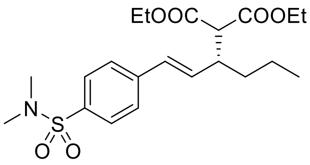

Diethyl (*R*,*E*)-2-(1-(4-(*N*,*N*-dimethylsulfamoyl)phenyl)hex-1-en-3-yl)malonate (**3o**)

Colorless oil, 51% yield, $[\alpha]_D^{25}$ +26.1 (*c* 2.2, $CHCl_3$) for 97:3 er; ^1H NMR (500 MHz, chloroform-*d*) δ 7.70 (d, *J* = 8.1 Hz, 2H), 7.49 (d, *J* = 8.1 Hz, 2H), 6.49 (d, *J* = 15.8 Hz, 1H), 6.25 (dd, *J* = 15.8, 9.6 Hz, 1H), 4.25 – 4.19 (m, 2H), 4.18 – 4.10 (m, 2H), 3.46 (d, *J* = 8.4 Hz, 1H), 3.02 – 2.96 (m, 1H), 2.71 (s, 6H), 1.59 – 1.37 (m, 3H), 1.28 (t, *J* = 7.1 Hz, 4H), 1.20 (t, *J* = 7.1 Hz, 3H), 0.92 (t, *J* = 7.2 Hz, 3H). ^{13}C NMR (126 MHz, $CDCl_3$) δ 168.3, 168.2, 141.5, 133.9, 133.8, 130.8, 128.1, 126.6, 61.5, 61.3, 61.3, 57.0, 43.3, 38.0, 34.9, 20.5, 14.2, 13.9. HRMS (EI): $[M]^{\oplus}$ calcd for $C_{21}H_{31}O_6NS^{\oplus}$ 425.1867, found 425.1870. HPLC analysis: Chiralpak IG column; detected at 254 nm, 40 °C; 5% ^{i}PrOH in *n*-Hexane; flow = 0.7 mL/min; Retention time: 21.5 min (minor), 25.4 min (major).

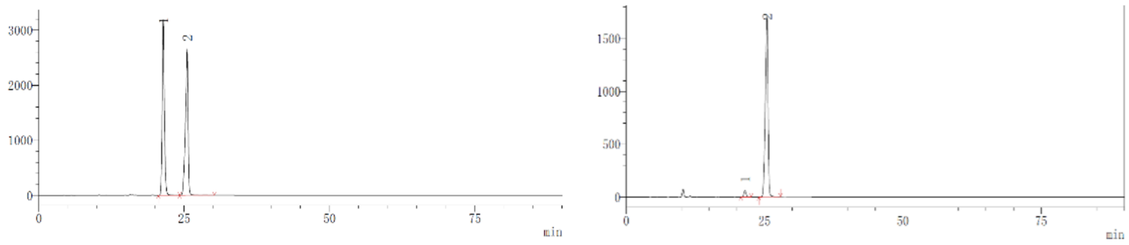

Number	Ret. Time	Area	Area%
1	21.383	81689009	49.61
2	25.503	82958198	50.39

Number	Ret. Time	Area	Area%
1	21.460	1718770	2.86
2	25.448	58361865	97.14

Supplementary Fig. 17. HPLC of **3o**.

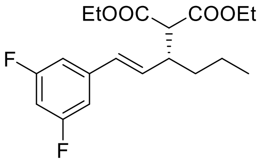

Diethyl (*R*,*E*)-2-(1-(3,5-difluorophenyl)hex-1-en-3-yl)malonate (**3p**)

Colorless oil, 73% yield, $[\alpha]_D^{25}$ +34.9 (*c* 0.78, $CHCl_3$) for 96:4 er; ^1H NMR (500 MHz, chloroform-*d*) δ 6.88 – 6.79 (m, 2H), 6.70 – 6.60 (m, 1H), 6.36 (d, *J* = 15.7 Hz, 1H), 6.10 (dd, *J* = 15.7, 9.7 Hz, 1H), 4.23 – 4.19 (m, 2H), 4.17 – 4.09 (m, 2H), 3.42 (d, *J* = 8.5 Hz, 1H), 2.98 – 2.91 (m, 1H), 1.57 – 1.47 (m, 1H), 1.47 – 1.35 (m, 2H), 1.29 – 1.24 (m, 4H), 1.19 (t, *J* = 7.1 Hz, 3H), 0.90 (t, *J* = 7.2 Hz, 3H). ^{13}C NMR (126 MHz, $CDCl_3$) δ 168.3, 168.2, 163.2 (dd, 13.9, 248.2 Hz), 132.9, 130.5 (t, *J* = 3.0 Hz), 109.0 (d, *J* = 6.3 Hz), 108.8 (d, *J* = 6.0 Hz), 102.6 (t, *J* = 26.5 Hz), 61.5, 61.3, 57.0, 43.2, 34.9, 20.4, 14.2, 13.9 (one alkyl

carbon signal was not observed because of overlapping). ^{19}F NMR (376 MHz, Chloroform-*d*) δ, -110.55. HRMS (EI): $[M]^{\oplus}$ calcd for $C_{19}H_{24}O_4F_2^{\oplus}$ 354.1637, found 354.1639. HPLC analysis: Chiracel AD-H column; detected at 254 nm, 40 °C; 5% ^{i}PrOH in *n*-Hexane; flow = 0.7 mL/min; Retention time: 7.7 min (major), 10.1 min (minor).

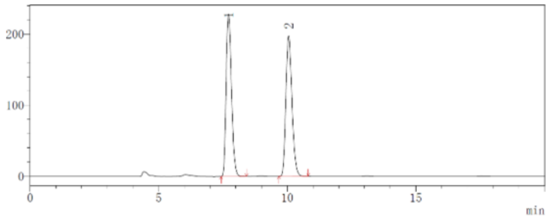

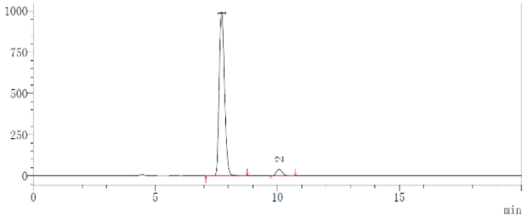

Number	Ret. Time	Area	Area%
1	7.715	3319281	50.51
2	10.053	3252899	49.49

Number	Ret. Time	Area	Area%
1	7.718	15174707	95.59
2	10.071	700501	4.41

Supplementary Fig. 18. HPLC of **3p**.

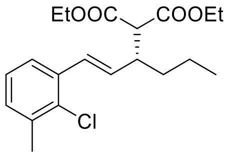

Diethyl (*R*,*E*)-2-(1-(2-chloro-3-methylphenyl)hex-1-en-3-yl)malonate (3q)

Colorless oil, 75% yield, $[\alpha]_D^{25}$ +24.6 (*c* 1.5, $CHCl_3$) for 94:6 er; ^{1}H NMR (400 MHz, chloroform-*d*) δ 7.32 (dd, *J* = 6.9, 2.6 Hz, 1H), 7.14 – 7.05 (m, 2H), 6.86 (d, *J* = 15.7 Hz, 1H), 6.00 (dd, *J* = 15.7, 9.6 Hz, 1H), 4.25 – 4.11 (m, 4H), 3.44 (d, *J* = 8.6 Hz, 1H), 3.05 – 2.97 (m, 1H), 2.36 (s, 3H), 1.60 – 1.49 (m, 1H), 1.48 – 1.37 (m, 2H), 1.33 – 1.25 (m, 4H), 1.21 (t, *J* = 7.1 Hz, 3H), 0.91 (t, *J* = 7.1 Hz, 3H). ^{13}C NMR (101 MHz, $cdcl_3$) δ 168.4, 168.2, 136.6, 135.7, 133.0, 132.7, 129.7, 129.5, 126.2, 124.7, 61.4, 61.3, 57.3, 43.4, 34.9, 20.7, 20.4, 14.1, 14.1, 13.9. HRMS (EI): $[M]^{\oplus}$ calcd for $C_{20}H_{27}O_4Cl^{\oplus}$ 366.1592, found 366.1596. HPLC analysis: Chiralpak ID column; detected at 254 nm, 40 °C; 5% ^{i}PrOH in *n*-Hexane; flow = 0.7 mL/min; Retention time: 16.7 min (minor), 18.8 min (major).

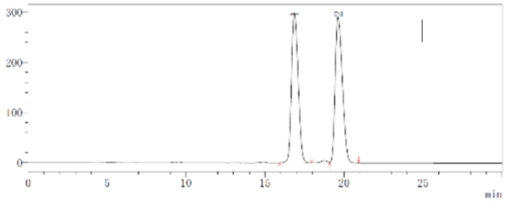

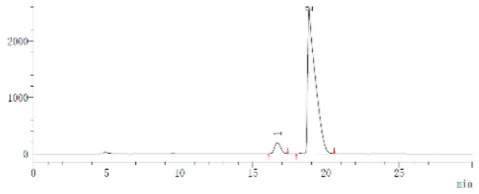

Number	Ret. Time	Area	Area%
1	16.856	8858159	48.89
2	19.618	9260280	51.11

Number	Ret. Time	Area	Area%
1	16.668	5620440	5.68
2	18.842	93284488	94.32

Supplementary Fig. 19. HPLC of **3q**.

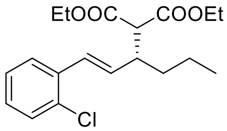

Diethyl (*R*,*E*)-2-(1-(2-chlorophenyl)hex-1-en-3-yl)malonate (3r)

Colorless oil, 41% yield, $[\alpha]_D^{25}$ +24.7 (*c* 1.1, $CHCl_3$) for 95:5 er; ^{1}H NMR (500 MHz, Chloroform-*d*) δ 7.48 (dd, *J* = 7.7, 1.8 Hz, 1H), 7.32 (dd, *J* = 7.8, 1.5 Hz, 1H), 7.21 – 7.13 (m, 2H), 6.81 (d, *J* = 15.8 Hz, 1H), 6.06 (dd, *J* = 15.8, 9.6 Hz, 1H), 4.25 – 4.17 (m, 3H), 4.19 – 4.11 (m, 2H), 3.44 (d, *J* = 8.6 Hz, 1H), 3.06 – 2.96 (m, 1H), 1.59 – 1.48 (m, 1H), 1.48 – 1.37 (m, 2H), 1.37 – 1.28 (m, 2H), 1.27 (t, *J* = 7.1 Hz, 3H), 1.21 (t, *J* = 7.1 Hz, 3H), 0.91 (t, *J* = 7.2 Hz, 3H). ^{13}C NMR (126 MHz, $CDCl_3$) δ 168.4, 168.2, 135.4, 132.9, 132.8, 129.6, 128.7, 128.4, 127.0, 126.8, 61.5, 61.4, 57.3, 43.4, 35.0, 20.4, 14.18, 14.16, 13.9. HRMS (ESI): $[M+Na]^{\oplus}$ calcd for $C_{19}H_{25}O_4ClNa^{\oplus}$ 375.1334, found 375.1333. HPLC analysis: Chiracel AD-H column; detected at 254 nm, 40 °C; 5% ^{i}PrOH in *n*-Hexane; flow = 0.7 mL/min; Retention time: 7.1min (minor), 7.8 min (major).

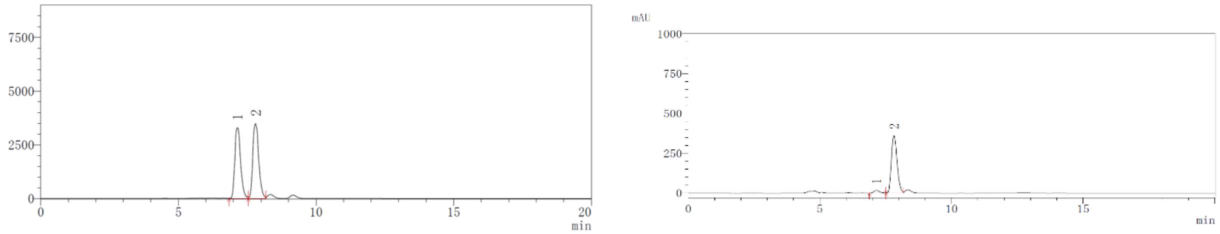

Number	Ret. Time	Area	Area%
1	7.145	48198465	47.90
2	7.805	52421412	52.10

PDA Ch1 254nm

Number	Ret. Time	Area	Area%
1	7.147	260651	4.55
2	7.813	5464834	95.45

Supplementary Fig. 20. HPLC of **3r**.

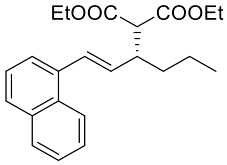

Diethyl (*R*,*E*)-2-(1-(naphthalen-1-yl)hex-1-en-3-yl)malonate (3s)

Colorless oil, 58% yield, $[\alpha]_D^{25}$ +19.8 (*c* 2.3, $CHCl_3$) for 91:9 er; ^{1}H NMR (500 MHz, chloroform-*d*) δ 8.07 (dd, *J* = 8.2, 1.4 Hz, 1H), 7.85 – 7.80 (m, 1H), 7.75 (d, *J* = 8.1 Hz, 1H), 7.55 – 7.37 (m, 4H), 7.19 (d, *J* = 15.5 Hz, 1H), 6.04 (dd, *J* = 15.5, 9.7 Hz, 1H), 4.28 – 4.19 (m, 2H), 4.17 – 4.08 (m, 2H), 3.48 (d, *J* = 9.0 Hz, 1H), 3.15 – 3.07 (m, 1H), 1.63 – 1.32 (m, 4H), 1.28 (t, *J* = 7.1 Hz, 3H), 1.17 (t, *J* = 7.1 Hz, 3H), 0.94 (t, *J* = 7.1 Hz, 3H). ^{13}C NMR (126 MHz, $CDCl_3$) δ 168.5, 168.4, 135.1, 133.6, 133.2, 131.2, 130.0, 128.5, 127.8, 126.0, 125.8, 125.7, 123.9, 123.9, 61.5, 61.3, 57.5, 43.7, 35.1, 20.6, 14.2, 14.2, 14.0. HRMS (EI): $[M]^{\oplus}$ calcd for $C_{23}H_{28}O_4^{\oplus}$ 368.1982, found 368.1977. HPLC analysis: Chiralpak IE column; detected at 254 nm, 40 °C; 1% ^{i}PrOH in *n*-Hexane; flow = 0.7 mL/min; Retention time:18.1 min (major), 20.0 min (minor).

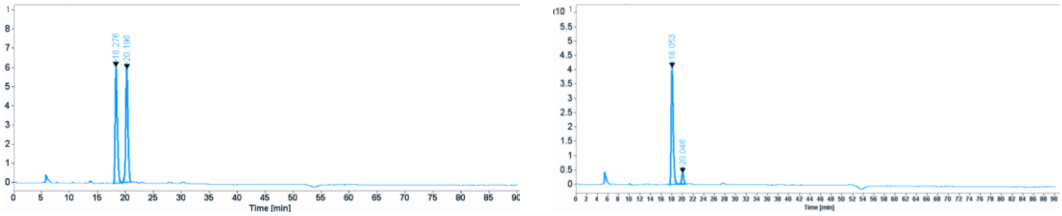

RT [min]	Width [min]	Area [mAU*S]	Height [mAU]	Area%
18.276	0.395	1598.604	60.441	49.5174
20.196	0.429	1629.764	59.216	50.4826

RT [min]	Width [min]	Area [mAU*S]	Height [mAU]	Area%
18.053	0.393	1042.382	40.697	90.7054
20.046	0.424	106.813	3.938	9.2946

Supplementary Fig. 21. HPLC of **3s**.

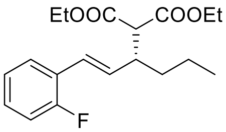

Diethyl (*R*,*E*)-2-(1-(2-fluorophenyl)hex-1-en-3-yl)malonate (3t)

Colorless oil, 68% yield, $[\alpha]_D^{25}$ +32.7 (*c* 1.9, $CHCl_3$) for 96:4 er; ^{1}H NMR (500 MHz, chloroform-*d*) δ 7.45 – 7.38 (m, 1H), 7.21 – 7.13 (m, 1H), 7.10 – 7.03 (m, 1H), 7.03 – 6.95 (m, 1H), 6.59 (d, *J* = 16.0 Hz, 1H), 6.14 (dd, *J* = 16.0, 9.7 Hz, 1H), 4.24 – 4.19 (m, 2H), 4.16 – 4.10 (m, 2H), 3.43 (d, *J* = 8.7 Hz, 1H), 3.00 – 2.94 (m, 1H), 1.58 – 1.48 (m, 1H), 1.48 – 1.36 (m, 2H), 1.32 – 1.25 (m, 4H), 1.20 (t, *J* = 7.1 Hz, 3H), 0.90 (t, *J* = 7.2 Hz, 3H). ^{13}C NMR (126 MHz, $CDCl_3$) δ 168.4, 168.2, 160.1 (d, *J* = 248.8 Hz), 132.6 (d, *J* = 5.0 Hz), 128.6 (d, *J* = 8.5 Hz), 127.4 (d, *J* = 4.2 Hz), 124.9 (d, *J* = 12.3 Hz), 124.8 (d, *J* = 3.9 Hz), 124.1 (d, *J* = 3.8 Hz), 115.7 (d, *J* = 22.1 Hz), 61.4, 61.3, 57.3, 43.7, 35.0, 20.4, 14.2, 14.1, 13.9. ^{19}F NMR (376 MHz, $CDCl_3$) δ -118.52. HRMS (EI): $[M]^{\oplus}$ calcd for $C_{19}H_{25}O_4F^{\oplus}$ 336.1731, found 336.1730. HPLC analysis: Chiralpak ID column; detected at 254 nm, 40 °C; 5% ^{i}PrOH in *n*-Hexane; flow = 0.7 mL/min; Retention time: 9.3 min (minor), 9.7 min (major).

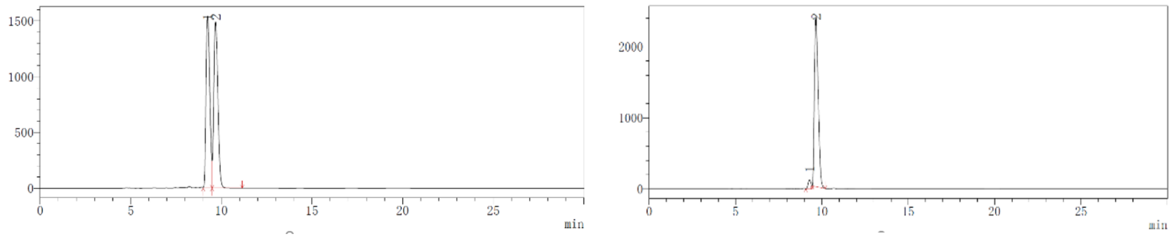

Number	Ret. Time	Area	Area%
1	9.239	22895586	49.34
2	9.679	23511501	50.66

Number	Ret. Time	Area	Area%
1	9.288	1538467	4.07
2	9.660	36243375	95.93

Supplementary Fig. 22. HPLC of **3t**.

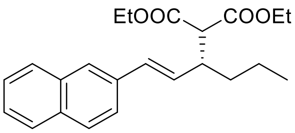

Diethyl (*R*,*E*)-2-(1-(naphthalen-2-yl)hex-1-en-3-yl)malonate (3u)

Colorless oil, 72% yield, $[\alpha]_D^{25}$ +36.4 (c 0.62, $CHCl_3$) for 95:5 er; ^{1}H NMR (500 MHz, chloroform-*d*) δ 7.81 – 7.73 (m, 3H), 7.68 (s, 1H), 7.61 – 7.53 (m, 1H), 7.48 – 7.38 (m, 2H), 6.60 (d, *J* = 15.7 Hz, 1H), 6.17 (dd, *J* = 15.8, 9.6 Hz, 1H), 4.26 – 4.17 (m, 2H), 4.17 – 4.06 (m, 2H), 3.45 (d, *J* = 8.8 Hz, 1H), 3.04 – 2.98 (m, 1H), 1.60 – 1.52 (m, 1H), 1.50 – 1.38 (m, 2H), 1.36 – 1.30 (m, 1H), 1.27 (t, *J* = 7.2 Hz, 3H), 1.16 (t, *J* = 7.1 Hz, 3H), 0.91 (t, *J* = 7.2 Hz, 3H). ^{13}C NMR (126 MHz, $CDCl_3$) δ 168.5, 168.4, 134.6, 133.7, 132.9, 132.6, 130.3, 128.2, 128.0, 127.7, 126.3, 126.0, 125.8, 123.7, 61.5, 61.3, 57.4, 43.6, 35.2, 20.5, 14.2, 13.9. HRMS (ESI): $[M+Na]^{\oplus}$ calcd for $C_{23}H_{28}O_4Na^{\oplus}$ 391.1880, found 391.1879. HPLC analysis: Chiralpak IE column; detected at 254 nm, 40 °C; 1% ^{i}PrOH in *n*-Hexane; flow = 0.7 mL/min; Retention time: 25.7 min (major), 28.4 min (minor).

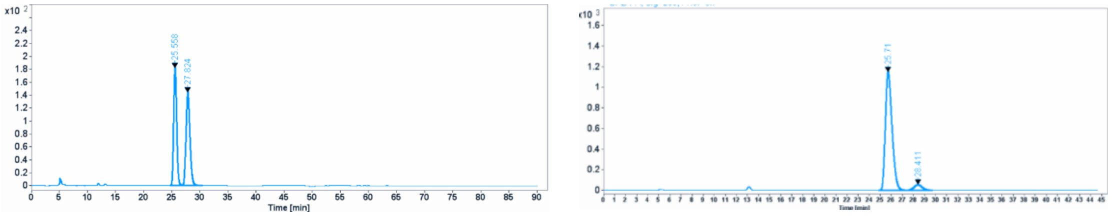

RT [min]	Width [min]	Area [mAU*S]	Height [mAU]	Area%
25.558	0.589	6820.565	182.097	49.4971
27.824	0.730	6959.159	144.015	50.5029

RT [min]	Width [min]	Area [mAU*S]	Height [mAU]	Area%
25.710	0.621	47376.164	1148.395	94.7918
28.411	0.772	2602.993	51.844	5.2082

Supplementary Fig. 23. HPLC of **3u**.

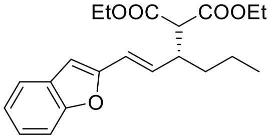

Diethyl (*R*,*E*)-2-(1-(benzofuran-2-yl)hex-1-en-3-yl)malonate (3v)

Colorless oil, 93% yield, $[\alpha]_D^{25}$ +37.1 (*c* 2.7, $CHCl_3$) for 96:4 er; ^{1}H NMR (500 MHz, chloroform-*d*) δ 7.51 (dd, *J* = 7.7, 1.4 Hz, 1H), 7.44 (d, *J* = 8.2 Hz, 1H), 7.28 – 7.23 (m, 1H), 7.23 – 7.17 (m, 1H), 6.53 (s, 1H), 6.42 (d, *J* = 15.7 Hz, 1H), 6.31 (dd, *J* = 15.7, 9.6 Hz, 1H), 4.27 – 4.23 (m, 2H), 4.18 – 4.14 (m, 2H), 3.47 (d, *J* = 9.0 Hz, 1H), 3.04 – 2.97 (m, 1H), 1.58 – 1.53 (m, 1H), 1.52 – 1.41 (m, 2H), 1.35 – 1.29 (m, 4H), 1.21 (t, *J* = 7.1 Hz, 3H), 0.94 (t, *J* = 7.2 Hz, 3H). ^{1} ^{13}C NMR (126 MHz, $CDCl_3$) δ 168.3, 168.2, 154.7, 154.3, 132.2, 129.0, 124.4, 122.8, 121.1, 120.8, 110.9, 104.0, 61.5, 61.4, 57.1, 43.3, 35.0, 20.4, 14.2, 14.1, 13.9. HRMS (ESI): $[M+Na]^{\oplus}$ calcd for $C_{21}H_{26}O_5Na^{\oplus}$ 381.1672, found 381.1670. HPLC analysis: Chiracel AD-H column; detected at 254 nm, 40 °C; 5% ^{i}PrOH in *n*-Hexane; flow = 0.7 mL/min; Retention time: 9.6 min (major), 12.4 min (minor).

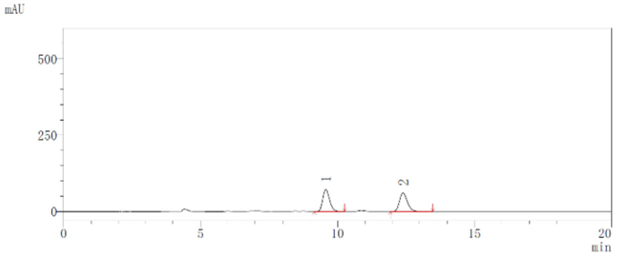

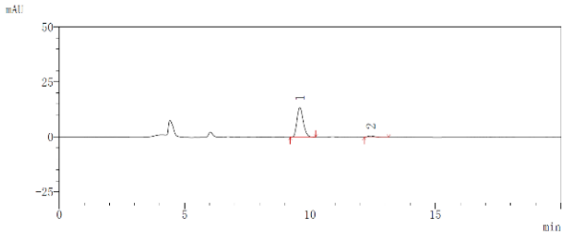

PDA Ch1 254nm

Number	Ret. Time	Area	Area%
1	9.578	1254988	50.70
2	12.395	1220576	49.30

PDA Ch1 254nm

Number	Ret. Time	Area	Area%
1	9.595	236089	95.78
2	12.416	10402	4.22

Supplementary Fig. 24. HPLC of **3v**.

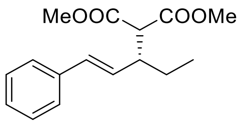

Dimethyl (*R*,*E*)-2-(1-phenylpent-1-en-3-yl)malonate (**3w**)

Colorless oil, 61% yield, $[\alpha]_D^{25}$ +38.0 (*c* 0.48, $CHCl_3$) for 96:4 er; ^{1}H NMR (500 MHz, chloroform-*d*) δ 7.34 (d, *J* = 8.4 Hz, 2H), 7.29 (t, *J* = 7.8 Hz, 2H), 7.25 – 7.18 (m, 1H), 6.45 (d, *J* = 15.7 Hz, 1H), 6.01 (dd, *J* = 15.7, 9.6 Hz, 1H), 3.74 (s, 3H), 3.65 (s, 3H), 3.48 (d, *J* = 9.0 Hz, 1H), 2.89 – 2.82 (m, 1H), 1.65 – 1.56 (m, 1H), 1.47 – 1.35 (m, 1H), 0.92 (t, *J* = 7.3 Hz, 3H). ^{13}C NMR (126 MHz, $CDCl_3$) δ 168.9, 168.7, 137.2, 132.8, 129.4, 128.6, 127.5, 126.4, 56.9, 52.5, 52.4, 45.3, 25.9, 11.9. HRMS (ESI): $[M+Na]^{\oplus}$ calcd for $C_{16}H_{20}O_4Na^{\oplus}$ 299.1254, found 299.1255. HPLC analysis: Chiracel AD-H column; detected at 254 nm, 40 ^{o}C; 5% ^{i}PrOH in *n*-Hexane; flow = 0.7 mL/min; Retention time: 9.8 min (major), 11.3 min (minor).

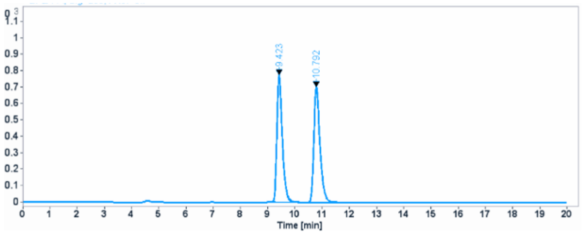

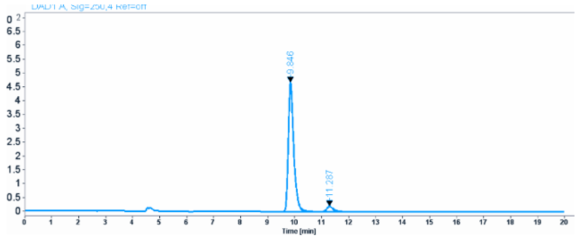

RT [min]	Width [min]	Area [mAU*S]	Height [mAU]	Area%
9.423	0.207	10745.709	776.276	49.6722
10.792	0.233	10887.542	702.109	50.3278

RT [min]	Width [min]	Area [mAU*S]	Height [mAU]	Area%
9.846	0.208	6478.008	467.711	95.5120
11.287	0.231	304.397	19.624	4.4880

Supplementary Fig. 25. HPLC of **3w**.

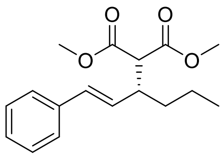

Dimethyl (*R*,*E*)-2-(1-phenylhex-1-en-3-yl)malonate (**3x**)

Colorless oil, 74% yield, $[\alpha]_D^{25}$ +41.2 (*c* 1.4, $CHCl_3$) for 95:5 er; ^1H NMR (400 MHz, chloroform-*d*) δ 7.37 – 7.31 (m, 2H), 7.32 – 7.23 (m, 2H), 7.25 – 7.17 (m, 1H), 6.44 (d, *J* = 15.8 Hz, 1H), 6.01 (dd, *J* = 15.8, 9.6 Hz, 1H), 3.74 (s, 3H), 3.64 (s, 3H), 3.46 (d, *J* = 8.8 Hz, 1H), 2.99 – 2.91 (m, 1H), 1.55 – 1.34 (m, 3H), 1.32 – 1.21 (m, 1H), 0.89 (t, *J* = 7.0 Hz, 3H). ^{13}C NMR (101 MHz, cdcl3) δ 168.8, 168.6, 137.1, 132.5, 129.7, 128.5, 127.4, 126.3, 57.1, 52.5, 52.3, 43.5, 35.0, 20.4, 13.9. HRMS (EI): $[M]^{\oplus}$ calcd for $C_{17}H_{22}O_4^{\oplus}$ 290.1506, found 290.1509. HPLC analysis: Chiracel AD-H column; detected at 254 nm, 40 °C; 1% ^{i}PrOH in *n*-Hexane; flow = 0.7 mL/min; Retention time:12.1min (major), 14.9 min (minor).

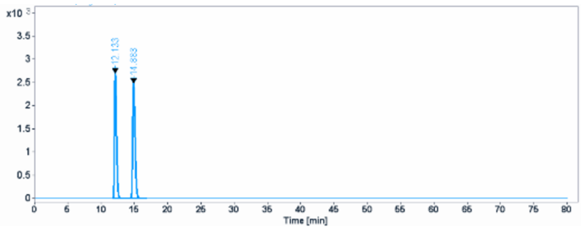

Signal: DAD1 A, Sig=250,4 Ref=off

RT [min]	Width [min]	Area [mAU*S]	Height [mAU]	Area%	ee value%
12.133	0.342	58620.332	2691.310	49.0080	RAC
14.883	0.385	60993.449	2477.860	50.9920	

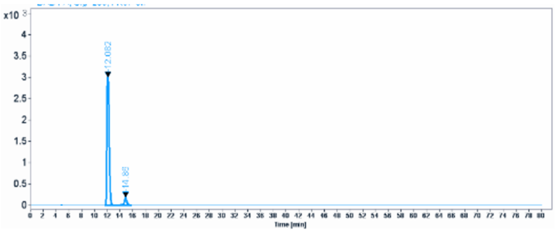

Signal: DAD1 A, Sig=250,4 Ref=off

RT [min]	Width [min]	Area [mAU*S]	Height [mAU]	Area%	ee value%
12.082	0.399	75360.727	3000.156	94.5966	89.193
14.860	0.353	4304.618	178.467	5.4034	

Supplementary Fig. 26. HPLC of **3x**.

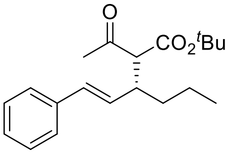

***tert*-Butyl (2*S*)-2-acetyl-3-((*E*)-styryl)hexanoate** (**3y**)

Colorless oil, 68% yield, $[\alpha]_D^{25}$ +49.7 (*c* 2.1, $CHCl_3$) for 97:3 er, dr = 3:2; The data of spectra were given as a mixture of two diastereoisomers. ^1H NMR (400 MHz, Chloroform-*d*) δ 7.36 – 7.26 (m, 4H), 7.25 – 7.14 (m, 1H), 6.47 – 6.37 (m, 1H), 6.04 – 5.87 (m, 1H), 3.45 – 3.34 (m, 1H), 2.99 – 2.87 (m, 1H), 2.29 – 2.10 (m, 3H), 1.53 – 1.27 (m, 13H), 0.92 – 0.85 (m, 3H). ^{13}C NMR (101 MHz, chloroform-*d*) δ 202.9, 168.0, 167.8, 137.1, 137.0, 132.3, 132.3, 130.01, 129.97, 128.6, 128.5, 127.4, 127.3, 126.3, 126.2, 82.1, 81.9, 66.44, 66.37, 43.12, 43.08, 35.2, 35.1, 29.4, 29.3, 27.99, 27.95, 20.4, 20.2, 13.94, 13.91. HRMS (EI): $[M]^{\oplus}$ calcd for $C_{20}H_{28}O_3^{\oplus}$ 316.2033, found 316.2028. HPLC analysis: Chiracel AD-H column; detected at 254 nm, 40 °C; 1% ^{i}PrOH in *n*-Hexane; flow = 0.7 mL/min; Retention time: 8.4 min (major), 10.4 min (minor).

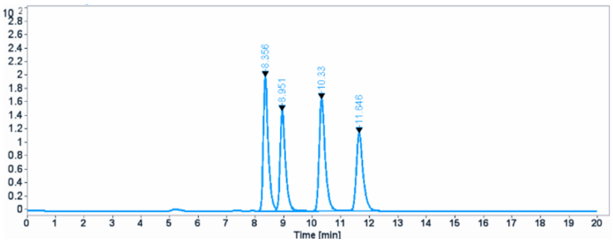

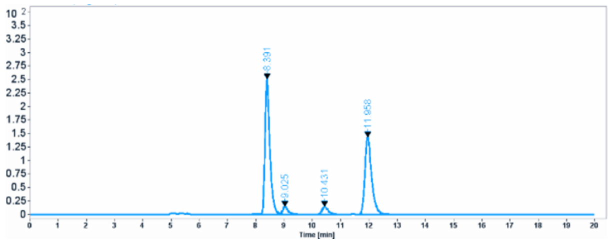

RT [min]	Width [min]	Area [mAU*S]	Height [mAU]	Area%
8.356	0.178	2391.716	199.227	27.6124
8.951	0.195	1956.320	148.869	22.5858
10.330	0.220	2422.573	166.686	27.9687
11.646	0.245	1891.128	115.502	21.8331

RT [min]	Width [min]	Area [mAU*S]	Height [mAU]	Area%
8.391	0.178	3015.917	250.813	52.1604
9.025	0.204	188.753	13.625	3.2645
10.431	0.223	217.655	14.545	3.7643
11.958	0.246	2359.683	143.019	40.8108

Supplementary Fig. 27. HPLC of **3y**.

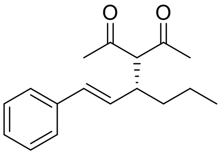

(*R*,*E*)-3-(1-phenylhex-1-en-3-yl)pentane-2,4-dione (**3z**)

Colorless oil, 45% yield, $[\alpha]_D^{25}$ +72.0 (*c* 0.68, $CHCl_3$) for 84:16 er; ^{1}H NMR (400 MHz, chloroform-*d*) δ 7.35 – 7.27 (m, 4H), 7.24 – 7.16 (m, 1H), 6.43 (d, *J* = 15.8 Hz, 1H), 5.85 (dd, *J* = 15.8, 9.6 Hz, 1H), 3.76 (d, *J* = 10.5 Hz, 1H), 3.09 – 3.01 (m, 1H), 2.23 (s, 3H), 2.10 (s, 3H), 1.47 – 1.26 (m, 4H), 0.88 (t, *J* = 6.9 Hz, 3H). ^{13}C NMR (101 MHz, cdcl$_3$) δ 203.7, 203.6, 136.8, 132.7, 129.4, 128.6, 127.6, 126.3, 74.9, 43.6, 35.3, 30.2, 29.8, 20.2, 13.9. HRMS (EI): $[M]^{\oplus}$ calcd for $C_{17}H_{22}O_2^{\oplus}$ 258.1614, found 258.1613. HPLC analysis: Chiracel AD-H column; detected at 254 nm, 40 °C; 2% ^{i}PrOH in *n*-Hexane; flow = 0.7 mL/min; Retention time: 12. 9 min (major), 13.7 min (minor).

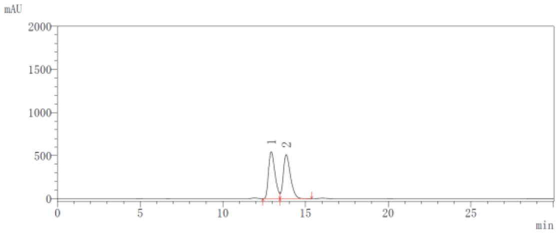

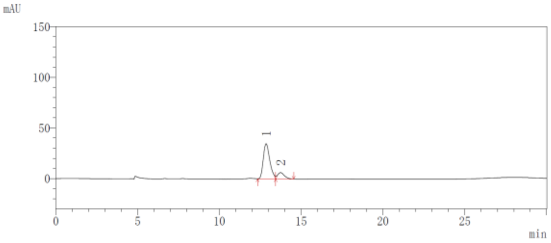

PDA Ch1 254nm

Number	Ret. Time	Area	Area%
1	12.927	14888425	49.16
2	13.834	15399510	50.84

PDA Ch1 254nm

Number	Ret. Time	Area	Area%
1	12.853	971374	83.77
2	13.735	188151	16.23

Supplementary Fig. 28. HPLC of **3z**.

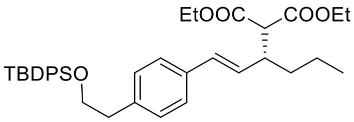

Diethyl (*R*,*E*)-2-(1-(4-(2-((*tert*-butyldiphenylsilyl)oxy)ethyl)phenyl)hex-1-en-3-yl)malonate (**4a**)

Colorless oil, 78% yield, $[\alpha]_D^{25}$ +23.8 (c 0.57, $CHCl_3$) for 97:3 er; ^{1}H NMR (500 MHz, chloroform-*d*) δ 7.60 – 7.55 (m, 4H), 7.43 – 7.37 (m, 2H), 7.37 – 7.30 (m, 4H), 7.23 – 7.19 (m, 2H), 7.06 (d, *J* = 7.9 Hz, 2H), 6.40 (d, *J* = 15.8 Hz, 1H), 5.97 (dd, *J* = 15.7, 9.6 Hz, 1H), 4.24 – 4.16 (m, 2H), 4.14 – 4.05 (m, 2H), 3.81 (t, *J* = 6.9 Hz, 2H), 3.43 – 3.35 (m, 1H), 2.95 – 2.87 (m, 1H), 2.81 (t, *J* = 6.8 Hz, 2H), 1.48 (d, *J* = 11.6 Hz, 1H), 1.39 (d, *J* = 10.6 Hz, 2H), 1.29 – 1.23 (m, 4H), 1.19 – 1.11 (m, 3H), 1.02 (s, 9H), 0.89 (t, *J*

= 7.2 Hz, 3H). ^{13}C NMR (126 MHz, $CDCl_3$) δ 168.5, 168.4, 138.5, 135.6, 135.1, 133.8, 132.3, 129.6, 129.4, 129.1, 127.7, 126.1, 65.2, 61.4, 61.3, 57.5, 43.5, 39.1, 35.2, 26.9, 20.4, 19.2, 14.22, 13.9 (one alkyl carbon signal was not observed because of overlapping). HRMS (ESI): $[M+Na]^{\oplus}$ calcd for $C_{37}H_{48}O_5NaSi^{\oplus}$ 623.3163, found 623.3166. The er value was determined by the HPLC analysis of the desilyl derivative of **4a** as shown below.

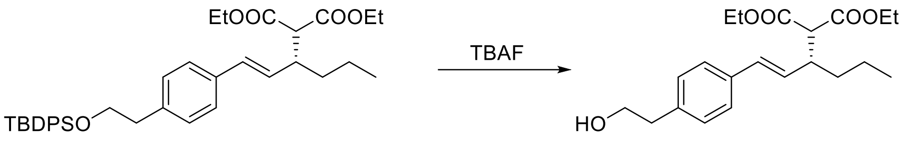

4a **4a***

Diethyl (*R*,*E*)-2-(1-(4-(2-hydroxyethyl)phenyl)hex-1-en-3-yl)malonate (**4a***): To a 25mL flask were added **4a** (43 mg, 0.070 mmol) and TBAF (1M in THF, 0.7 mL). The mixture was stirred at room temperature for 12 h and monitored by TLC. The reaction was quenched by water (3 mL), exacted by ethyl acetate (3 mL × 3), condensed and purified by flash column chromatography (hexane / ethyl acetate = 3/1) to give **4a*** as a colorless oil in 67% yield. $[\alpha]_D^{25}$ +35.1 (*c* 0.90, $CHCl_3$) for 97:3 er; ^{1}H NMR (500 MHz, chloroform-*d*) δ 7.29 (d, *J* = 7.9 Hz, 2H), 7.16 (d, *J* = 7.8 Hz, 2H), 6.41 (d, *J* = 15.7 Hz, 1H), 6.00 (dd, *J* = 15.7, 9.6 Hz, 1H), 4.23 – 4.18 (m, 2H), 4.15 – 4.08 (m, 2H), 3.84 (t, *J* = 6.6 Hz, 2H), 3.41 (d, *J* = 8.8 Hz, 1H), 2.97 – 2.90 (m, 1H), 2.84 (t, *J* = 6.5 Hz, 2H), 1.54 – 1.47 (m, 1H), 1.45 – 1.35 (m, 3H), 1.32 – 1.23 (m, 4H), 1.18 (t, *J* = 7.1 Hz, 3H), 0.89 (t, *J* = 7.1 Hz, 3H). ^{13}C NMR (126 MHz, $CDCl_3$) δ 168.5, 168.3, 137.7, 135.6, 132.1, 129.5, 129.2, 126.5, 63.7, 61.4, 61.3, 57.4, 43.4, 38.9, 35.1, 20.4, 14.2, 14.2, 13.9. HRMS (ESI): $[M+Na]^{\oplus}$ calcd for $C_{21}H_{30}O_5Na^{\oplus}$ 385.1985, found 385.1987. HPLC analysis: Chiracel AD-H column; detected at 254 nm, 40 °C; 10 % ^{i}PrOH in *n*-Hexane; flow = 0.7 mL/min; Retention time: 20.9 min (minor), 22.6 min (major).

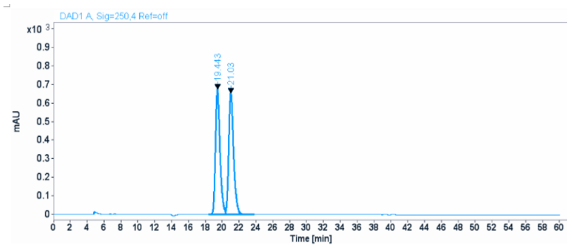

Signal: DAD1 A, Sig=250,4 Ref=off

RT [min]	Width [min]	Area [mAU*S]	Height [mAU]	Area%	ee value%
19.443	0.564	24971.553	671.975	49.0951	RAC
21.030	0.597	25892.043	652.505	50.9049	

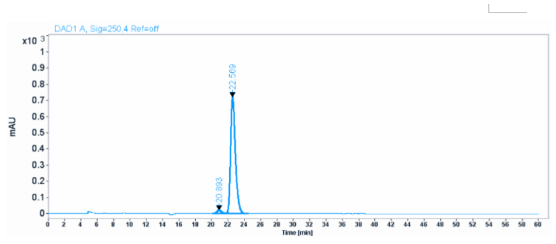

Signal: DAD1 A, Sig=250,4 Ref=off

RT [min]	Width [min]	Area [mAU*S]	Height [mAU]	Area%	ee value%
20.893	0.579	801.211	20.909	2.6853	94.629
22.569	0.606	29035.691	718.347	97.3147	

Supplementary Fig. 29. HPLC of **4a’**.

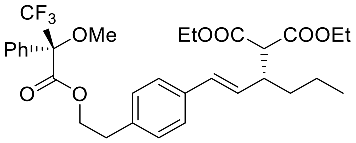

Diethyl 2-((*R*,*E*)-1-(4-(2-(((*S*)-3,3,3-trifluoro-2-methoxy-2-phenylpropanoyl)oxy)ethyl)phenyl)hex-1-en-3-yl)malonate (**4b**)

Colorless oil, 81% yield, $[\alpha]_D^{25}$ +21.5 (*c* 3.3, $CHCl_3$) for 97:3 dr; ^{1}H NMR (500 MHz, chloroform-*d*) δ 7.41 – 7.35 (m, 3H), 7.35 – 7.28 (m, 2H), 7.24 (d, *J* = 7.8 Hz, 2H), 7.09 (d, *J* = 7.8 Hz, 2H), 6.41 (d, *J* = 15.7 Hz, 1H), 6.02 (dd, *J* = 15.7, 9.7 Hz, 1H), 4.56 – 4.47 (m, 2H), 4.24 – 4.18 (m, 2H), 4.15 – 4.05 (m, 2H), 3.47 (s, 3H), 3.42 (d, *J* = 8.8 Hz, 1H), 3.04 – 2.91 (m, 3H), 1.53 – 1.49 (m, 1H), 1.46 – 1.35 (m, 2H), 1.29 – 1.22 (m, 4H), 1.16 (t, *J* = 7.1 Hz, 3H), 0.90 (t, *J* = 7.1 Hz, 3H). ^{13}C NMR (126 MHz, $CDCl_3$) δ 168.4, 168.3, 166.6, 136.2, 135.8, 132.0, 129.7, 129.6, 129.1, 128.4, 127.3 (q, *J* = 1.7 Hz), 126.5, 66.8, 61.4, 61.2, 57.3, 55.4 (q, *J* = 1.9 Hz), 43.4, 35.01, 34.4, 20.4, 14.2, 14.2, 13.9 (the carbon signal of CF_3 was not observed; one more alkyl carbon signal was also not observed because of overlapping). ^{19}F NMR (376 MHz, chloroform-*d*) δ -91.68. HRMS (ESI): $[M+Na]^{\oplus}$ calcd for $C_{31}H_{37}O_7F_3Na^{\oplus}$ 601.2384, found 601.2391. HPLC analysis: Chiracel AD-H column; detected at 254 nm, 30 °C; 5% ^{i}PrOH in *n*-Hexane; flow = 0.7 mL/min; Retention time: 24.6 min (major), 26.3 min (minor).

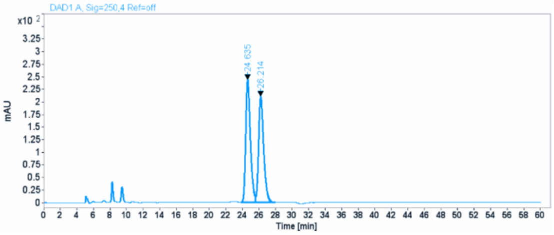

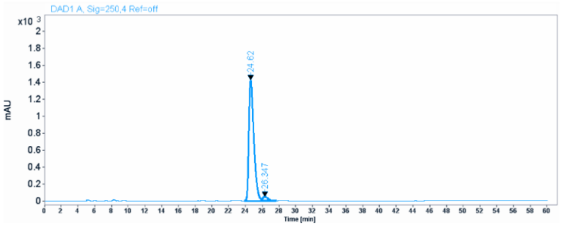

Signal: DAD1 A, Sig=250,4 Ref=off

RT [min]	Width [min]	Area [mAU*S]	Height [mAU]	Area%	ee value%
24.635	0.584	9459.979	243.295	51.6061	RAC
26.214	0.631	8871.154	210.138	48.3939	

Signal: DAD1 A, Sig=250,4 Ref=off

RT [min]	Width [min]	Area [mAU*S]	Height [mAU]	Area%	ee value%
24.620	0.622	58689.242	1427.052	96.7699	93.540
26.347	0.486	1959.005	47.770	3.2301	

Supplementary Fig. 30. HPLC of **4b**.

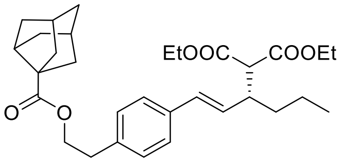

Diethyl 2-((*R*,*E*)-1-(4-octahydro-2,5-methanopentalene-2-carbonyl)oxy)ethyl)phenyl)hex-1-en-3-yl)malonate (**4c**)

Colorless oil, 58% yield, $[\alpha]_D^{25}$ +25.2 (*c* 1.2, $CHCl_3$) for 98:2 er; ^{1}H NMR (500 MHz, chloroform-*d*) δ 7.29 – 7.26 (m, 2H), 7.15 (d, *J* = 7.8 Hz, 2H), 6.41 (d, *J* = 15.8 Hz, 1H), 6.00 (dd, *J* = 15.7, 9.6 Hz, 1H), 4.27 (t, *J* = 6.8 Hz, 2H), 4.20 (q, *J* = 7.1 Hz, 2H), 4.13 – 4.08 (m, 2H), 3.40 (d, *J* = 8.8 Hz, 1H), 2.98 – 2.84 (m, 3H), 2.58 (t, *J* = 6.8 Hz, 1H), 2.27 (s, 2H), 2.01 (d, *J* = 11.0 Hz, 2H), 1.81 – 1.72 (m, 4H), 1.63 – 1.55 (m, 4H), 1.53 – 1.46 (m, 1H), 1.43 – 1.36 (m, 2H), 1.29 – 1.23 (m, 4H), 1.17 (t, *J* = 7.1 Hz, 3H), 0.89 (t, *J* = 7.1 Hz, 3H). ^{13}C NMR (126 MHz, $CDCl_3$) δ 177.6, 168.5, 168.3, 137.4, 135.5, 132.1, 129.5, 129.2, 126.3, 64.8, 61.4, 61.2, 57.4, 53.8, 46.9, 44.1, 44.1, 43.7, 43.45, 37.5, 35.1, 35.0, 34.8, 20.4, 14.2, 13.9. HRMS (EI): $[M]^{\oplus}$ calcd for $C_{31}H_{42}O_6^{\oplus}$ 510.2976, found 510.2982. HPLC analysis: Chiracel AD-H column; detected at 254 nm, 40 °C; 5% ^{i}PrOH in *n*-Hexane; flow = 0.7 mL/min; Retention time:19.9 min (major), 21.3 min (minor).

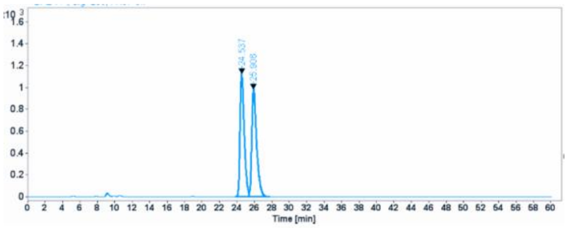

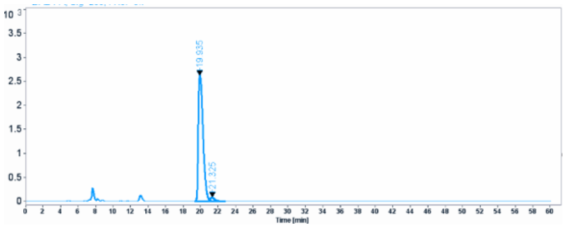

Signal: DAD1 A, Sig=250,4 Ref=off

RT [min]	Width [min]	Area [mAU*S]	Height [mAU]	Area%	ee value%
24.537	0.506	37459.688	1125.084	49.8765	RAC
25.906	0.576	37645.152	984.713	50.1235	

Signal: DAD1 A, Sig=250,4 Ref=off

RT [min]	Width [min]	Area [mAU*S]	Height [mAU]	Area%	ee value%
19.935	0.591	97831.539	2621.152	97.5813	95.163
21.325	0.499	2424.886	73.138	2.4187	

Supplementary Fig. 31. HPLC of **4c**.

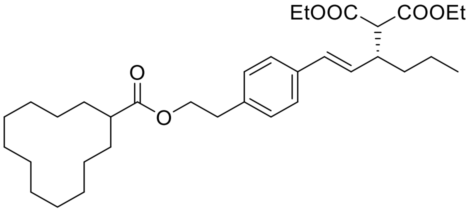

Diethyl (*R*,*E*)-2-(1-(4-(2-((cyclododecanecarbonyl)oxy)ethyl)phenyl)hex-1-en-3-yl)malonate (4d)

Colorless oil, 54% yield, $[\alpha]_D^{25}$ +32.2 (*c* 1.8, $CHCl_3$) for 98:2 er; ^{1}H NMR (500 MHz, chloroform-*d*) δ 7.26 (d, *J* = 5.8 Hz, 2H), 7.14 (d, *J* = 7.8 Hz, 2H), 6.41 (d, *J* = 15.8 Hz, 1H), 6.00 (dd, *J* = 15.8, 9.6 Hz, 1H), 4.29 – 4.16 (m, 4H), 4.16 – 4.06 (m, 2H), 3.40 (d, *J* = 8.9 Hz, 1H), 2.97 – 2.87 (m, 3H), 2.48 – 2.43 (m, 1H), 1.60 – 1.48 (m, 5H), 1.41 – 1.25 (m, 24H), 1.17 (t, *J* = 7.1 Hz, 3H), 0.89 (t, *J* = 7.1 Hz, 3H). ^{13}C NMR (126 MHz, $CDCl_3$) δ 176.7, 168.4, 168.3, 137.2, 135.5, 132.1, 129.5, 129.1, 126.3, 64.6, 61.4, 61.2, 57.4, 43.4, 40.4, 35.1, 35.0, 34.9, 26.6, 23.8, 23.6, 23.5, 22.3, 20.4, 14.2, 13.9 (one alkyl carbon signal was not observed because of overlapping). HRMS (ESI): $[M+Na]^{\oplus}$ calcd for $C_{34}H_{52}O_6Na^{\oplus}$ 579.3656, found 579.3659. HPLC analysis: Chiralpak AY3 column; detected at 214 nm, 40 ^{o}C; 5% ^{i}PrOH in *n*-Hexane; flow = 0.7 mL/min; Retention time: 13.1 min (major), 25.2 min (minor).

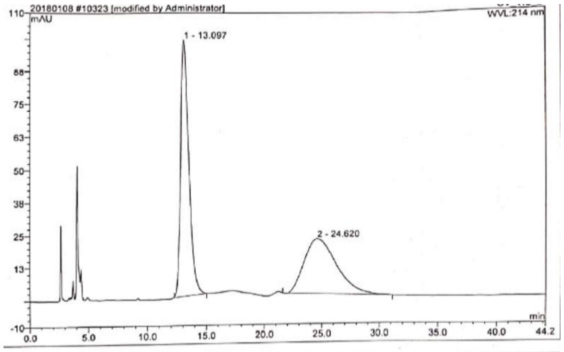

No.	Ret.Time min	Peak Name	Height mAU	Area mAU*min	Rel.Area %	Amount	Type
1	13.10	n.a.	98.438	79.442	53.39	n.a.	BMB*
2	24.62	n.a.	21.328	69.352	46.61	n.a.	BMB*
Total:			119.766	148.793	100.00	0.000	

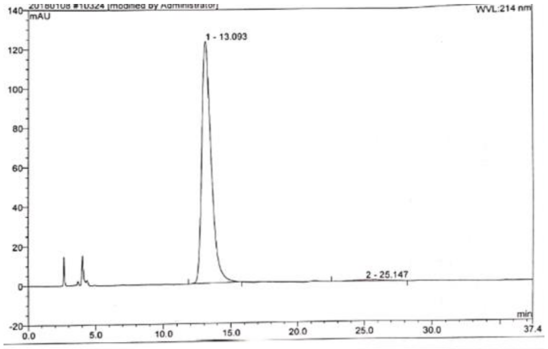

No.	Ret.Time min	Peak Name	Height mAU	Area mAU*min	Rel.Area %	Amount	Type
1	13.09	n.a.	123.418	99.903	98.38	n.a.	BMB*
2	25.15	n.a.	0.642	1.648	1.62	n.a.	BMB*
Total:			124.061	101.551	100.00	0.000	

Supplementary Fig. 32. HPLC of **4d**.

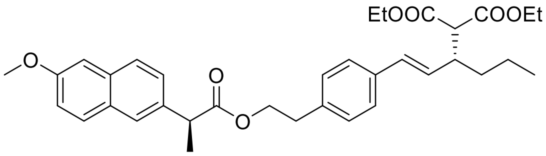

Diethyl 2-((*R*,*E*)-1-(4-(2-(((*S*)-2-(6-methoxynaphthalen-2-yl)propanoyl)oxy)ethyl)phenyl)hex-1-en-3-yl)malonate (4e)

Colorless oil, 88% yield, $[\alpha]_D^{25}$ +30.0 (*c* 1.8, $CHCl_3$) for 97:3 dr; ^1H NMR (500 MHz, chloroform-*d*) δ 7.71 – 7.65 (m, 2H), 7.65 – 7.61 (m, 1H), 7.35 (dd, *J* = 8.5, 1.9 Hz, 1H), 7.18 – 7.11 (m, 2H), 7.11 – 7.07 (m, 2H), 6.97 – 6.92 (m, 2H), 6.36 (d, *J* = 15.7 Hz, 1H), 5.94 (dd, *J* = 15.7, 9.6 Hz, 1H), 4.33 – 4.18 (m, 4H), 4.17 – 4.03 (m, 2H), 3.93 (s, 3H), 3.82 (q, *J* = 7.1 Hz, 1H), 3.40 (d, *J* = 8.9 Hz, 1H), 2.98 – 2.88 (m, 1H), 2.84 – 2.78 (m, 2H), 1.55 (d, *J* = 7.1 Hz, 3H), 1.52 – 1.34 (m, 3H), 1.28 – 1.25 (m, 4H), 1.16 (t, *J* = 7.1 Hz, 3H), 0.90 (t, *J* = 7.2 Hz, 3H). ^{13}C NMR (126 MHz, $CDCl_3$) δ 174.6, 168.5, 168.3, 157.7, 137.0, 135.7, 135.4, 133.8, 132.1, 129.4, 129.4, 129.1, 129.0, 127.20, 126.3, 126.3, 126.1, 119.1, 105.6, 65.2, 61.4, 61.2, 57.4, 55.4, 45.5, 43.4, 35.1, 34.8, 20.4, 18.4, 14.2, 13.9 (one alkyl carbon signal was not observed because of overlapping). HRMS (ESI): $[M]^{\oplus}$ calcd for $C_{35}H_{43}O_7^{\oplus}$ 575.3003, found 575.3001. HRMS (ESI): $[M+H]^{\oplus}$ calcd for $C_{35}H_{43}O_7^{\oplus}$ 575.3003, found 575.3001. HPLC analysis: Chiracel AD-H column; detected at 214 nm, 40 °C; 10% ^{i}PrOH in *n*-Hexane; flow = 0.7 mL/min; Retention time: 25.9 min (major), 28.0 min (minor).

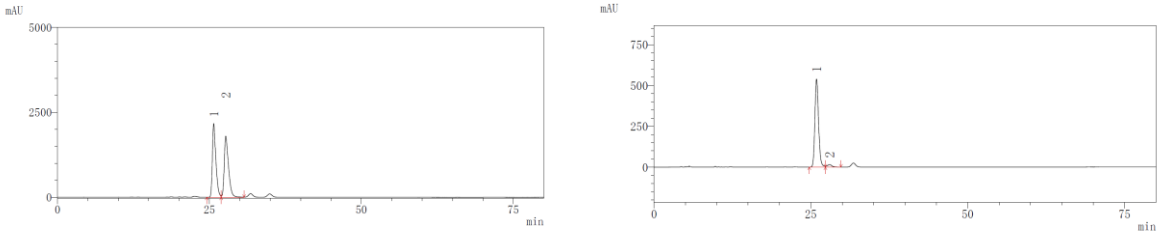

PDA Ch1 254nm

Number	Ret.Time	Area	Area%
1	25.701	85969048	49.48
2	27.686	87773256	50.52

PDA Ch1 254nm

Number	Ret.Time	Area	Area%
1	25.909	21889800	96.54
2	27.990	783757	3.46

Supplementary Fig. 33. HPLC of **4e**.

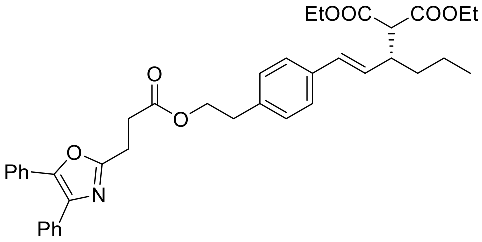

Diethyl (*R*,*E*)-2-(1-(4-(2-((3-(4,5-diphenyloxazol-2-yl)propanoyl)oxy)ethyl)phenyl)hex-1-en-3-yl)malonate (4f)

Colorless oil, 90% yield, $[\alpha]_D^{25}$ +18.7 (*c* 4.2, $CHCl_3$) for 97:3 er; ^1H NMR (500 MHz, chloroform-*d*) δ 7.66 – 7.60 (m, 2H), 7.59 – 7.52 (m, 2H), 7.39 – 7.28 (m, 6H), 7.28 – 7.23 (m, 2H), 7.16 – 7.08 (m, 2H),

6.40 (d, *J* = 15.7 Hz, 1H), 5.99 (dd, *J* = 15.7, 9.6 Hz, 1H), 4.32 (t, *J* = 7.0 Hz, 2H), 4.20 (q, *J* = 7.1 Hz, 2H), 4.14 – 4.07 (m, 2H), 3.40 (d, *J* = 8.8 Hz, 1H), 3.16 (dd, *J* = 8.3, 6.8 Hz, 2H), 2.99 – 2.86 (m, 5H), 1.56 – 1.46 (m, 1H), 1.46 – 1.33 (m, 2H), 1.33 – 1.20 (m, 4H), 1.17 (t, *J* = 7.1 Hz, 3H), 0.89 (t, *J* = 7.1 Hz, 3H). ^{13}C NMR (126 MHz, $CDCl_3$) δ 171.9, 168.4, 168.3, 161.7, 145.4, 136.9, 135.5, 135.1, 132.5, 132.0, 129.5, 129.0, 129.0, 128.6, 128.6, 128.5, 128.1, 127.9, 126.5, 126.4, 65.2, 61.3, 61.2, 57.3, 43.4, 35.0, 34.7, 31.1, 23.5, 20.4, 14.2, 13.9 (one alkyl carbon signal was not observed because of overlapping). HRMS (EI): $[M+H]^{\oplus}$ calcd for $C_{39}H_{44}O_7N^{\oplus}$ 638.3112, found 638.3111. HPLC analysis: Chiralpak AY3 column; detected at 254 nm, 40 °C; 15% ^{i}PrOH in *n*-Hexane; flow = 0.7 mL/min; Retention time: 12.5 min (major), 13.2 min (minor).

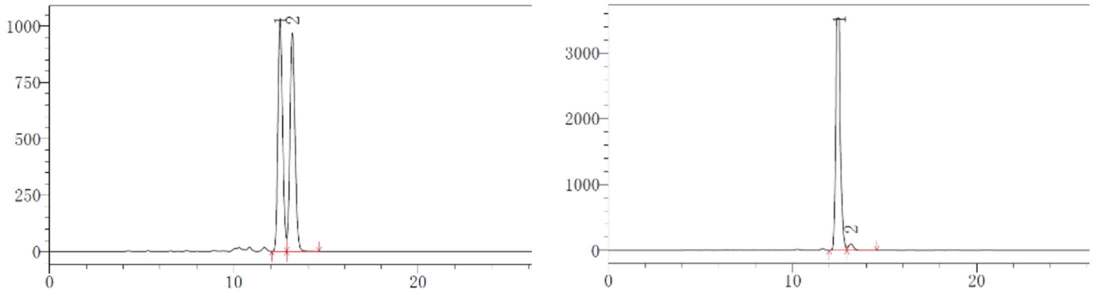

PDA Ch1 254nm

Number	Ret. Time	Area	Area%
1	12.510	18056675	49.58
2	13.173	18365714	50.42

PDA Ch1 254nm

Number	Ret. Time	Area	Area%
1	12.517	63019796	97.00
2	13.159	1946569	3.00

Supplementary Fig. 34. HPLC of **4f**.

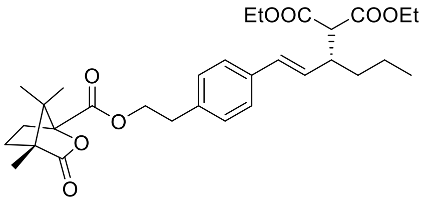

Diethyl 2-((*R*,*E*)-1-(4-(2-(((1*R*,4*S*)-4,7,7-trimethyl-3-oxo-2-oxabicyclo[2.2.1]heptane-1-carbonyl)oxy)ethyl)phenyl)hex-1-en-3-yl)malonate (4g)

Colorless oil, 54% yield, $[\alpha]_D^{25}$ +24.5 (*c* 2.1, $CHCl_3$) for 98:2 dr; ^{1}H NMR (500 MHz, chloroform-*d*) δ 7.27 (d, *J* = 7.3 Hz, 2H), 7.15 (d, *J* = 7.8 Hz, 2H), 6.40 (d, *J* = 15.8 Hz, 1H), 6.01 (dd, *J* = 15.7, 9.6 Hz, 1H), 4.43 (t, *J* = 7.0 Hz, 2H), 4.20 (q, *J* = 7.1 Hz, 2H), 4.16 – 4.06 (m, 2H), 3.41 (d, *J* = 8.8 Hz, 1H), 3.01 – 2.88 (m, 3H), 2.41 – 2.32 (m, 1H), 2.03 – 1.94 (m, 1H), 1.94 – 1.85 (m, 1H), 1.71 – 1.63 (m, 1H), 1.56 – 1.46 (m, 1H), 1.45 – 1.34 (m, 2H), 1.31 – 1.23 (m, 4H), 1.17 (t, *J* = 7.1 Hz, 3H), 1.10 (s, 3H), 0.97 (s, 3H), 0.92 – 0.82 (m, 6H). ^{13}C NMR (126 MHz, $CDCl_3$) δ 178.1, 168.4, 168.3, 167.4, 136.2, 135.7, 131.9, 129.7, 129.1, 126.4, 91.1, 65.8, 61.4, 61.2, 57.3, 54.8, 54.1, 43.4, 35.1, 34.7, 30.7, 29.0, 20.4, 16.7, 16.6, 14.18, 14.16, 13.9, 9.7. HRMS (ESI): $[M+H]^{\oplus}$ calcd for $C_{31}H_{43}O_8^{\oplus}$ 543.2952, found 539.2958. HPLC analysis: Chiracel AD-H column; detected at 254 nm, 40 °C; 10% ^{i}PrOH in *n*-Hexane; flow = 0.7 mL/min; Retention time: 32.6 min (major), 36.3 min (minor).

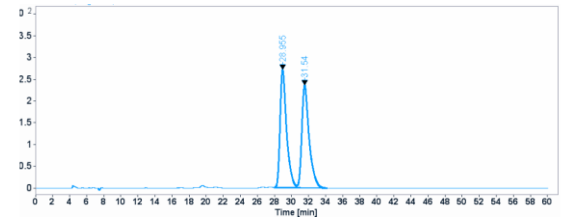

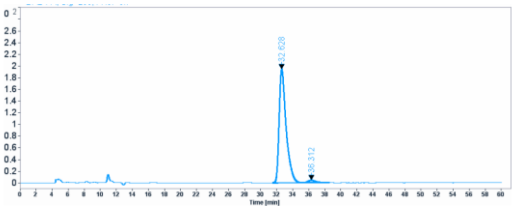

RT [min]	Width [min]	Area [mAU*S]	Height [mAU]	Area%
28.955	0.789	14273.158	270.095	50.4409
31.540	0.893	14023.617	234.876	49.5591

RT [min]	Width [min]	Area [mAU*S]	Height [mAU]	Area%
32.628	0.890	11662.672	194.805	97.7482
36.312	0.866	268.671	4.268	2.2518

Supplementary Fig. 35. HPLC of **4g**.

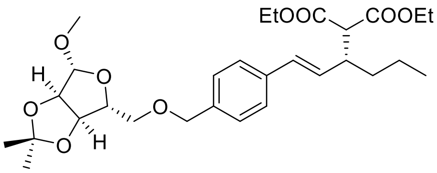

Diethyl 2-((*R*,*E*)-1-(4-((((3a*R*,4*R*,6*R*,6a*R*)-6-methoxy-2,2-dimethyltetrahydrofuro[3,4-d][1,3]dioxol-4-yl)methoxy)methyl)phenyl)hex-1-en-3-yl)malonate (4h)

Colorless oil, 80% yield, $[\alpha]_D^{25}$ -0.30 (*c* 1.5, $CHCl_3$) for 96:4 dr; ^{1}H NMR (500 MHz, chloroform-*d*) δ 7.31 (d, *J* = 8.0 Hz, 2H), 7.26 (d, *J* = 8.2 Hz, 2H), 6.42 (d, *J* = 15.7 Hz, 1H), 6.03 (dd, *J* = 15.8, 9.7 Hz, 1H), 4.96 (s, 1H), 4.67 (d, *J* = 6.0 Hz, 1H), 4.60 – 4.47 (m, 3H), 4.36 (t, *J* = 7.2 Hz, 1H), 4.21 (q, *J* = 7.1 Hz, 2H), 4.15 – 4.07 (m, 2H), 3.50 (dd, *J* = 9.5, 6.4 Hz, 1H), 3.43 (dd, *J* = 16.9, 8.6 Hz, 2H), 3.29 (s, 3H), 2.99 – 2.89 (m, 1H), 1.56 – 1.49 (m, 2H), 1.46 – 1.35 (m, 2H), 1.35 – 1.23 (m, 9H), 1.18 (t, *J* = 7.1 Hz, 3H), 0.89 (t, *J* = 7.1 Hz, 3H). ^{13}C NMR (126 MHz, $CDCl_3$) δ 168.4, 168.3, 137.1, 136.7, 132.1, 130.0, 128.0, 126.3, 112.4, 109.3, 85.22, 85.19, 82.2, 73.0, 71.0, 61.4, 61.3, 57.3, 54.9, 43.4, 35.1, 26.5, 25.1, 20.4, 14.2, 13.9 (one alkyl carbon signal was not observed because of overlapping). HRMS (EI): $[M]^{\oplus}$ calcd for $C_{29}H_{42}O_9{}^{\oplus}$ 534.2823, found 5334.2822. HPLC analysis: Chiracel AD-H column; detected at 254 nm, 40 ^{o}C; 10% ^{i}PrOH in *n*-Hexane; flow = 0.7 mL/min; Retention time: 15.2 min (major), 17.0 min (minor).

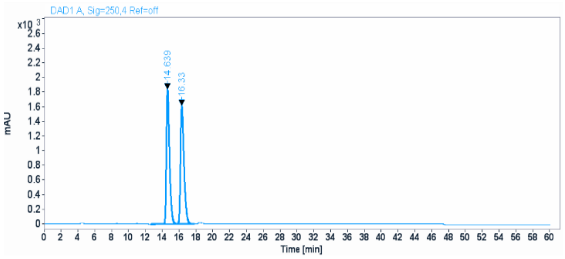

Signal: DAD1 A, Sig=250,4 Ref=off

RT [min]	Width [min]	Area [mAU*S]	Height [mAU]	Area%	ee value%
14.639	0.392	47518.449	1837.856	50.6710	RAC
16.330	0.436	46259.934	1614.657	49.3290	

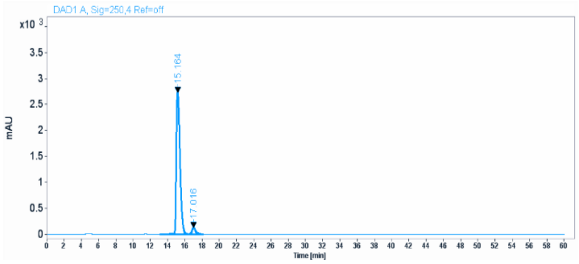

Signal: DAD1 A, Sig=250,4 Ref=off

RT [min]	Width [min]	Area [mAU*S]	Height [mAU]	Area%	ee value%
15.164	0.464	81190.961	2724.909	95.9977	91.995
17.016	0.437	3384.995	117.070	4.0023	

Supplementary Fig. 36. HPLC of **4h**.

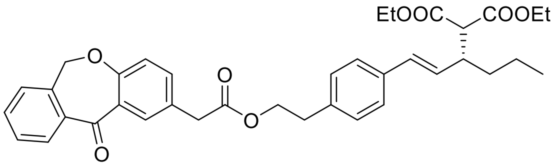

Diethyl (*R*,*E*)-2-(1-(4-(2-(2-(11-oxo-6,11-dihydrodibenzo[b,e]oxepin-2-yl)acetoxy)ethyl)phenyl)hex-1-en-3-yl)malonate (4i)

Colorless oil, 61% yield, $[\alpha]_D^{25}$ +20.6 (*c* 3.6, $CHCl_3$) for 98:2 er; ^{1}H NMR (500 MHz, chloroform-*d*) δ 8.11 (d, *J* = 2.4 Hz, 1H), 7.89 (dd, *J* = 7.8, 1.3 Hz, 1H), 7.55 (td, *J* = 7.5, 1.3 Hz, 1H), 7.47 (td, *J* = 7.6, 1.1 Hz, 1H), 7.39 – 7.32 (m, 2H), 7.24 (d, *J* = 7.9 Hz, 2H), 7.09 (d, *J* = 7.8 Hz, 2H), 7.00 (d, *J* = 8.5 Hz, 1H), 6.40 (d, *J* = 15.8 Hz, 1H), 6.00 (dd, *J* = 15.8, 9.7 Hz, 1H), 5.18 (s, 2H), 4.29 (t, *J* = 7.0 Hz, 2H), 4.23 – 4.18 (m, 2H), 4.14 – 4.08 (m, 2H), 3.61 (s, 2H), 3.41 (d, *J* = 8.9 Hz, 1H), 2.99 – 2.86 (m, 3H), 1.56 – 1.46 (m, 1H), 1.45 – 1.34 (m, 2H), 1.31 – 1.23 (m, 4H), 1.17 (t, *J* = 7.1 Hz, 3H), 0.89 (t, *J* = 7.1 Hz, 3H). ^{13}C NMR (126 MHz, $CDCl_3$) δ 190.8, 171.3, 168.4, 168.2, 160.5, 140.4, 136.8, 136.3, 135.6, 135.5, 132.8, 132.5, 132.0, 129.5, 129.5, 129.3, 129.1, 127.8, 127.7, 126.3, 125.1, 121.0, 73.6, 65.4, 61.3, 61.2, 57.3, 43.3, 40.2, 35.0, 34.7, 20.4, 14.15, 14.14, 13.9. HRMS (ESI): $[M+H]^{\oplus}$ calcd for $C_{37}H_{41}O_8^{\oplus}$ 613.2796, found 613.2794. HPLC analysis: Chiracel (AD-H)+(AD-H) column; detected at 254 nm, 40 °C; 8% ^{i}PrOH in *n*-Hexane; flow = 0.7 mL/min; Retention time: 144.5 min (major), 154.2 min (minor).

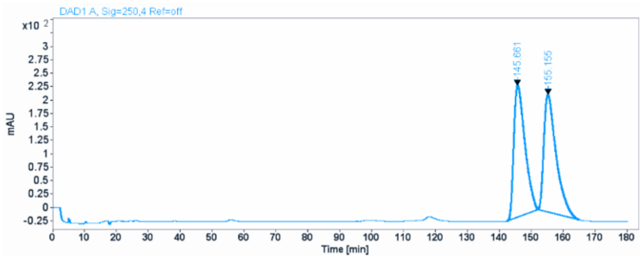

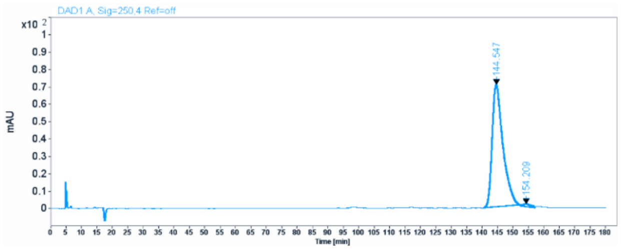

Signal: DAD1 A, Sig=250,4 Ref=off

RT [min]	Width [min]	Area [mAU*S]	Height [mAU]	Area%	ee value%
145.661	2.834	59318.648	244.920	50.1797	RAC
155.155	3.159	58893.738	218.065	49.8203	

Signal: DAD1 A, Sig=250,4 Ref=off

RT [min]	Width [min]	Area [mAU*S]	Height [mAU]	Area%	ee value%
144.547	2.854	17131.633	70.223	98.4972	96.994
154.209	3.265	261.384	1.334	1.5028	

Supplementary Fig. 37. HPLC of **4i**.

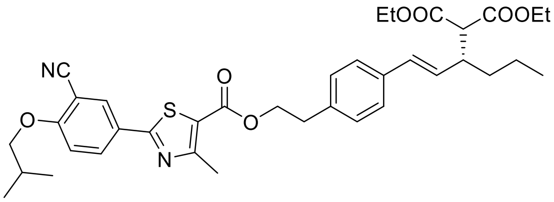

Diethyl (*R*,*E*)-2-(1-(4-(2-((2-(3-cyano-4-isobutoxyphenyl)-4-methylthiazole-5-carbonyl)oxy)ethyl)phenyl)hex-1-en-3-yl)malonate (4j)

Colorless oil, 57% yield, $[\alpha]_D^{25}$ +17.4 (c 2.9, $CHCl_3$) for 98:2 er; ^{1}H NMR (500 MHz, chloroform-*d*) δ 8.17 (d, *J* = 2.4 Hz, 1H), 8.08 (dd, *J* = 8.9, 2.4 Hz, 1H), 7.30 (d, *J* = 7.9 Hz, 2H), 7.20 (d, *J* = 8.0 Hz, 2H), 7.02 (d, *J* = 8.8 Hz, 1H), 6.42 (d, *J* = 15.7 Hz, 1H), 6.03 (dd, *J* = 15.8, 9.7 Hz, 1H), 4.48 (t, *J* = 6.8 Hz,

2H), 4.22 – 4.18 (m, 2H), 4.14 – 4.08 (m, 2H), 3.90 (d, J = 6.5 Hz, 2H), 3.41 (d, J = 8.8 Hz, 1H), 3.03 (t, J = 6.8 Hz, 2H), 2.99 – 2.90 (m, 1H), 2.72 (s, 3H), 2.23 – 2.18 (m, 1H), 1.56 – 1.46 (m, 1H), 1.46 – 1.36 (m, 3H), 1.31 – 1.24 (m, 7H), 1.17 (t, J = 7.2 Hz, 3H), 1.09 (d, J = 6.7 Hz, 6H), 0.89 (t, J = 7.1 Hz, 3H). ^{13}C NMR (126 MHz, $CDCl_3$) δ 168.4, 168.3, 167.3, 162.5, 161.9, 161.3, 136.7, 135.7, 132.6, 132.1, 132.0, 129.7, 129.1, 126.5, 126.0, 121.6, 115.4, 112.6, 102.9, 75.7, 65.8, 61.4, 61.2, 57.3, 43.4, 35.0, 34.8, 28.2, 20.4, 19.1, 17.5, 14.1, 13.9 (one alkyl carbon signal was not observed because of overlapping). HRMS (ESI): $[M+H]^{\oplus}$ calcd for $C_{37}H_{45}O_7N_2S^{\oplus}$ 661.2942, found 661.2936. HPLC analysis: Chiracel AD-H column; detected at 254 nm, 40 °C; 20% ^{i}PrOH in n-Hexane; flow = 0.7 mL/min; Retention time: 28.2 min (major), 30.8 min (minor).

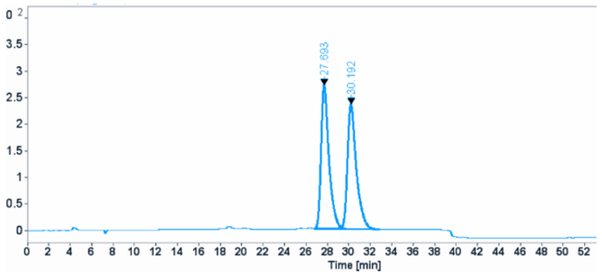

Signal: DAD1 A, Sig=250,4 Ref=off

RT [min]	Width [min]	Area [mAU*S]	Height [mAU]	Area%	ee value%
27.693	0.752	13644.440	269.777	50.4582	RAC
30.192	0.850	13396.656	233.992	49.5418	

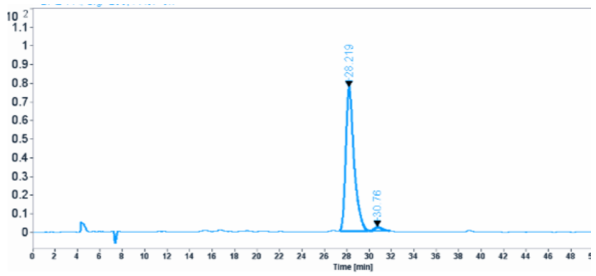

Signal: DAD1 A, Sig=250,4 Ref=off

RT [min]	Width [min]	Area [mAU*S]	Height [mAU]	Area%	ee value%
28.219	0.853	3944.942	77.056	98.0005	96.001
30.760	0.725	80.490	1.851	1.9995	

Supplementary Fig. 38. HPLC of **4j**.

5. Chain walking test of different nucleophiles

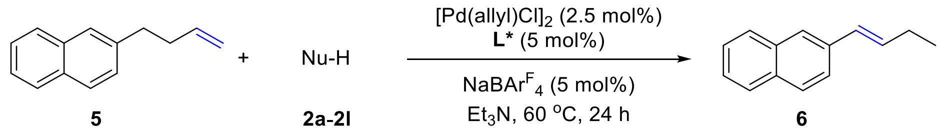

General procedure: To a 4 mL vial in the glovebox under nitrogen were added $[Pd(allyl)Cl]_2$ (0.90 mg, 0.0025 mmol), **L** (2.8 mg, 0.0050 mmol), sodium tetrakis[3,5-bis(trifluoromethyl)phenyl]borate ($NaBAr^F_4$, 4.4 mg, 0.0050 mmol) and dry Et_3N (0.2 mL). The mixture was stirred at room temperature for 5 min. Then 2-(but-3-en-1-yl)naphthalene **5** (20 mg, 0.11 mmol) was added to the solution and the reaction continued to stir for 1 min. Finally, the nucleophile **2** (0.1 mmol) was added to the reaction and the resulting mixture was stirred at 60 °C for 24 h. After this time, the reaction solution was cooled to room temperature, condensed and crude ^{1}H NMR was obtained with dibromomethane (7 µL, 0.1 mmol) as internal standard to help determine the reaction results.

The procedure for the test of migratory allylic substitution with different nucleophiles was the same as that described in the above part "General procedure for Pd-catalyzed migratory allylic substitution".

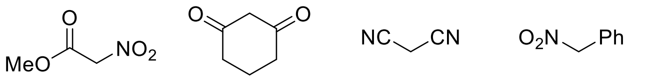

	2e	2f	2g	2h
pKa in DMSO:	~ 9.1	10.3	11.1	12.2
Yield of **6**:	46%	78%	56%	76%
Yield of **3**:	N.D.	N.D	trace	<10%

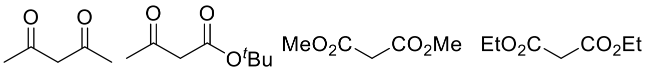

	2d	2c	2b	2a
pKa in DMSO:	13.3	~14.2	15.9	16.4
Yield of **6**:	49%	79%	75%	84%
Yield of **3**:	45%	68%	74%	82%

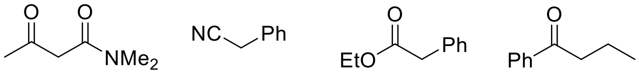

	2i	2j	2k	2l
pKa in DMSO:	18.2	21.9	22.7	~ 24.4
Yield of **6**:	55%	14%	N.D.	N.D.
Yield of **3**:	N.D.	N.D.	N.D.	N.D.

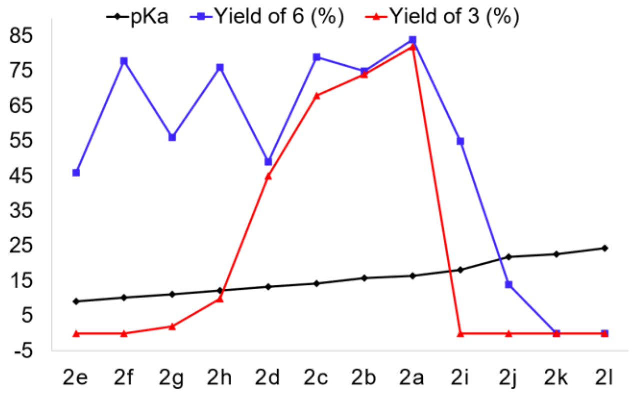

Supplementary Fig. 39. Chain-walking test of different nucleophiles.

6. Substrates ineffective for the migratory allylation

6.1 Test of diene substrates ineffective for the transformation

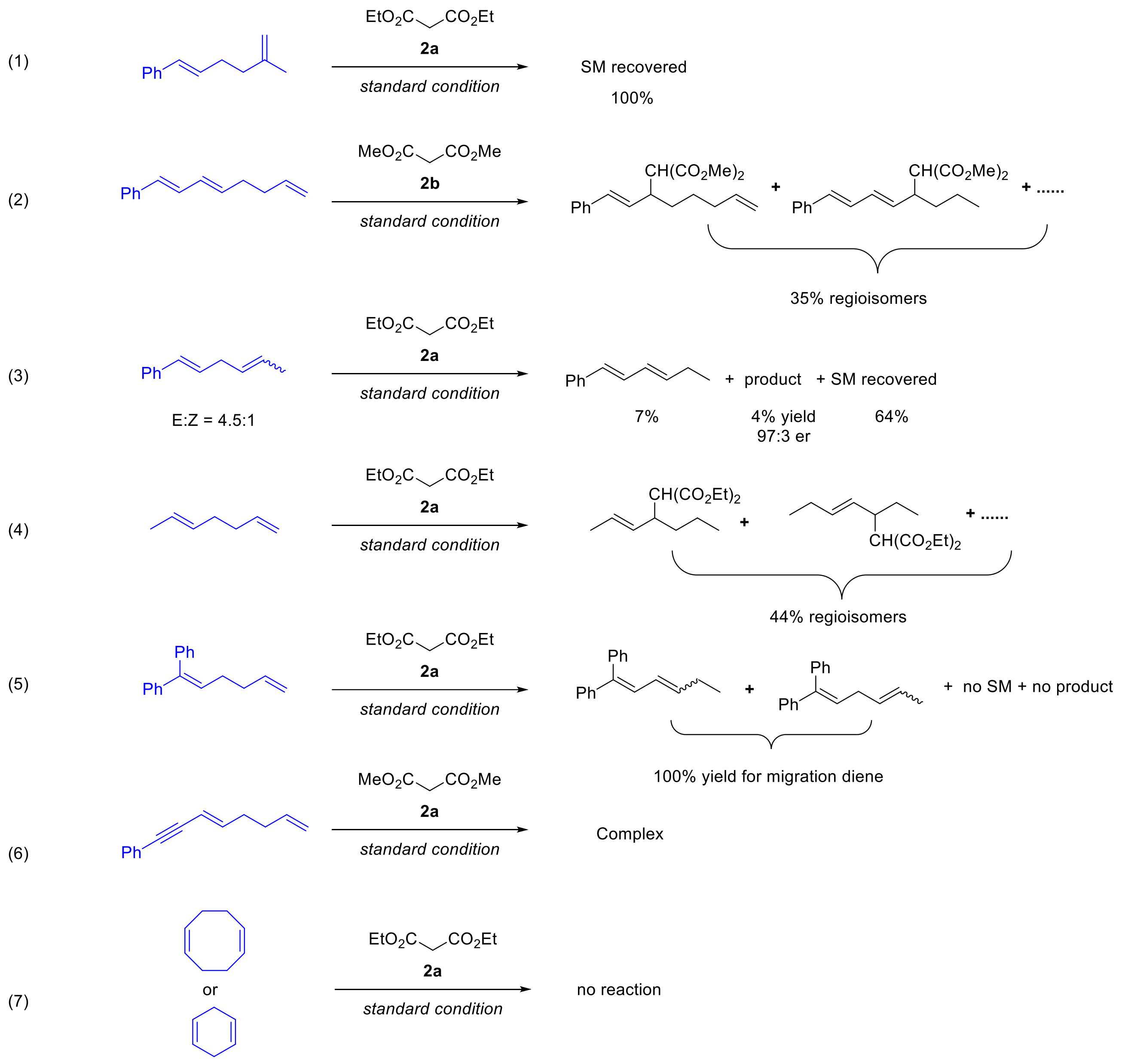

Supplementary Fig. 40. Other ineffective substrates tested for migratory allylation.

6.2 Test of nucleophiles ineffective for the transformation

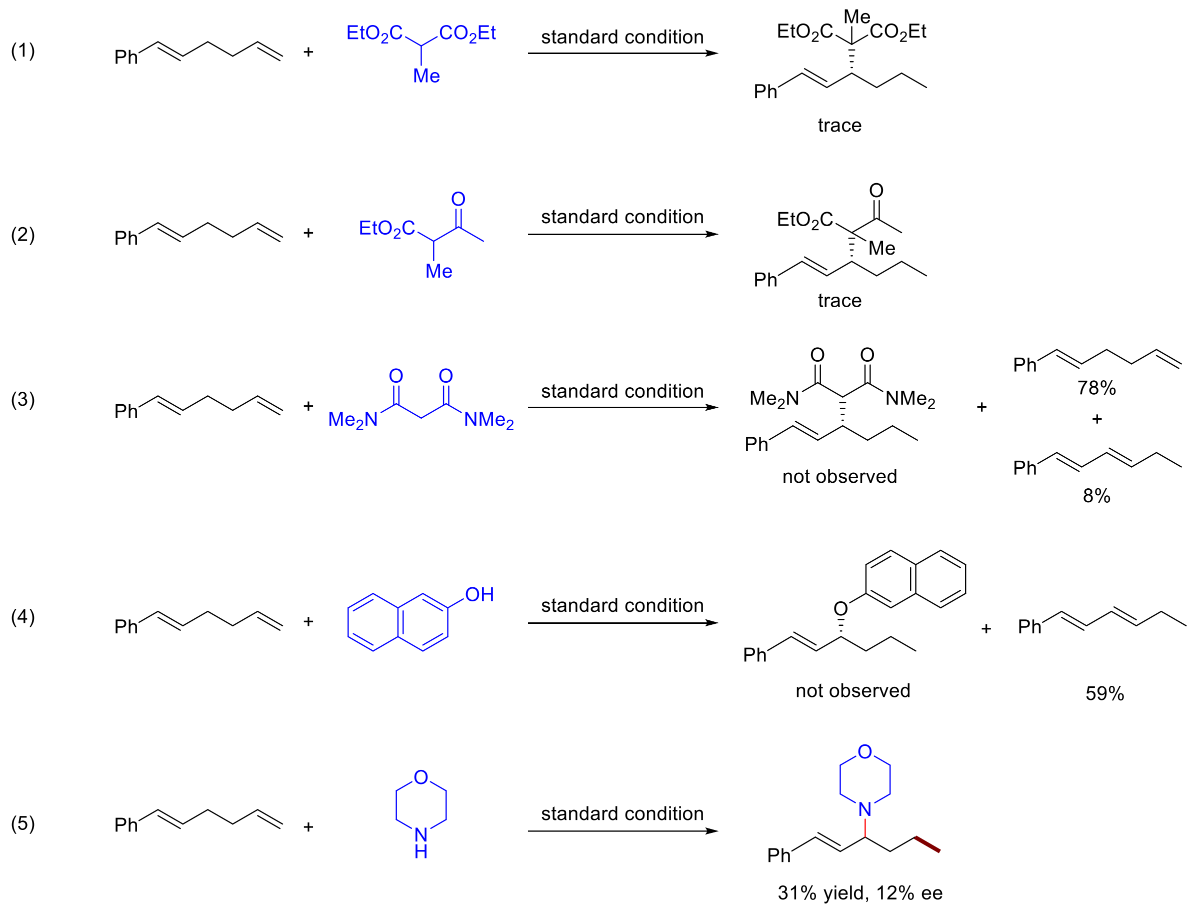

Supplementary Fig. 41. More substrates tested for migratory allylation.

7. Mechanistic studies

7.1 Control experiment

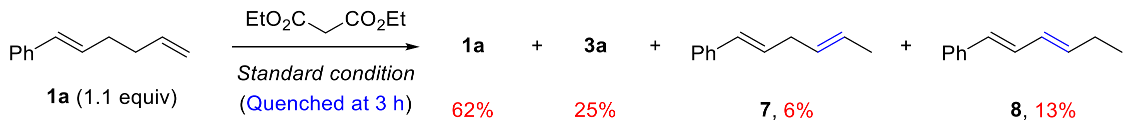

To a 4 mL vial in the glovebox under nitrogen were added $[Pd(allyl)Cl]_2$ (1.8 mg, 0.0050 mmol), **L** (5.6 mg, 0.010 mmol), sodium tetrakis[3,5-bis(trifluoromethyl)phenyl]borate ($NaBAr^F{}_4$, 8.8 mg, 0.010 mmol) and dry Et_3N (0.2 mL). The mixture was stirred at room temperature for 5 min. Then the remote diene **1a** (35 mg, 0.22 mmol) was added to the solution and the reaction continued to stir for 1 min. Finally, the

nucleophile **2a** (32 mg, 0.20 mmol) was added to the reaction and the resulting mixture was stirred at 60 °C for 3 h. After this time, the crude mixture was cooled to room temperature, condensed and crude ^{1}H NMR was obtained with dibromomethane (7 μL, 0.1 mmol) as internal standard to help determine the reaction results.

7.2 Deuteration experiment

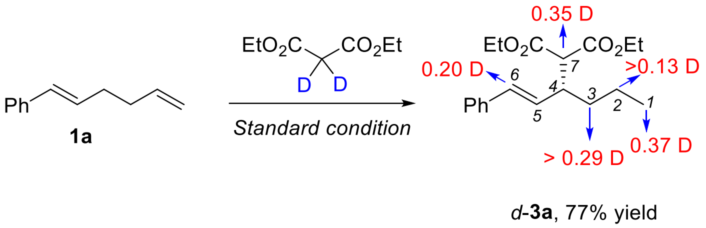

The procedure for deuteration experiment with deuterated-**2a** as nucleophile was the same as that described in the above part "General procedure for Pd-catalyzed migratory allylic substitution". The *d*-**3a** was isolated in 77% yield and according ^{1}H NMR and ^{2}D NMR were shown as below.

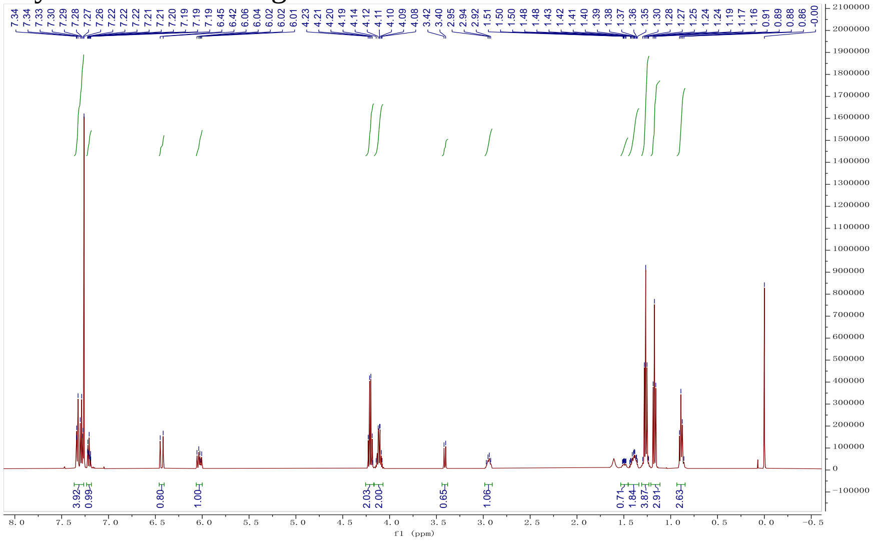

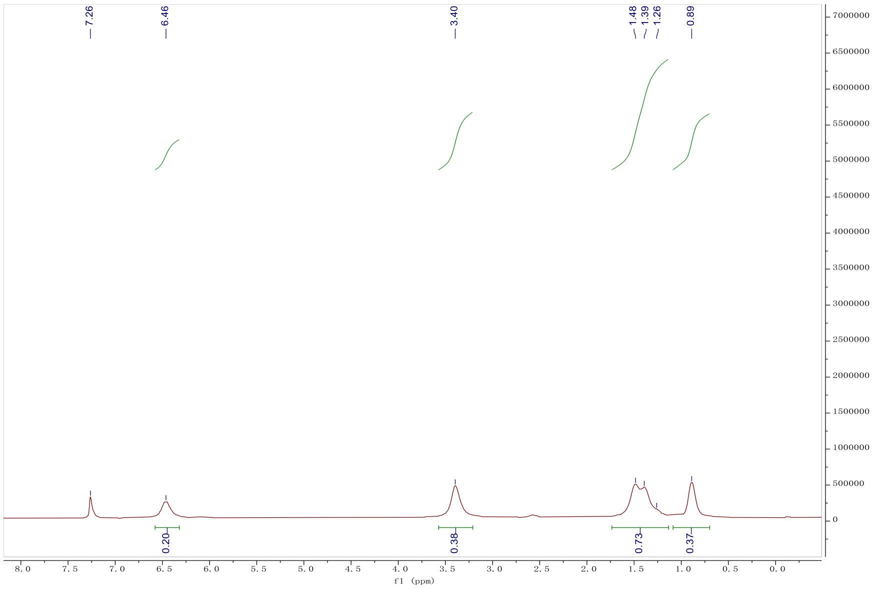

Supplementary Fig. 42. ^{1}H NMR and ^{2}D NMR of *d*-**3a**.

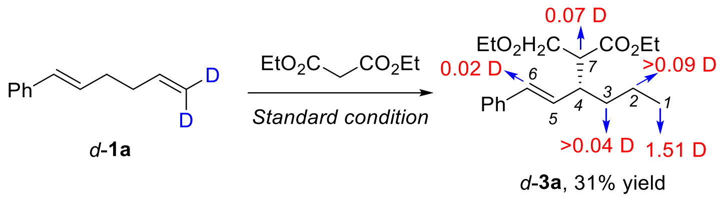

The procedure for deuteration experiment with deuterated-**1a** as electrophile was the same as that described in the above part "General procedure for Pd-catalyzed migratory allylic substitution". The *d*-**3a** was isolated in 31% yield and according ^{1}H NMR and ^{2}D NMR were shown as below.

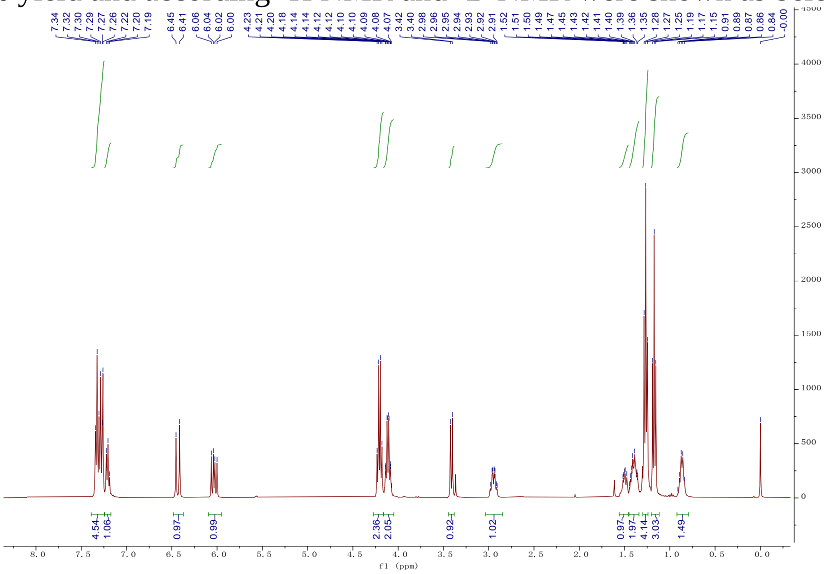

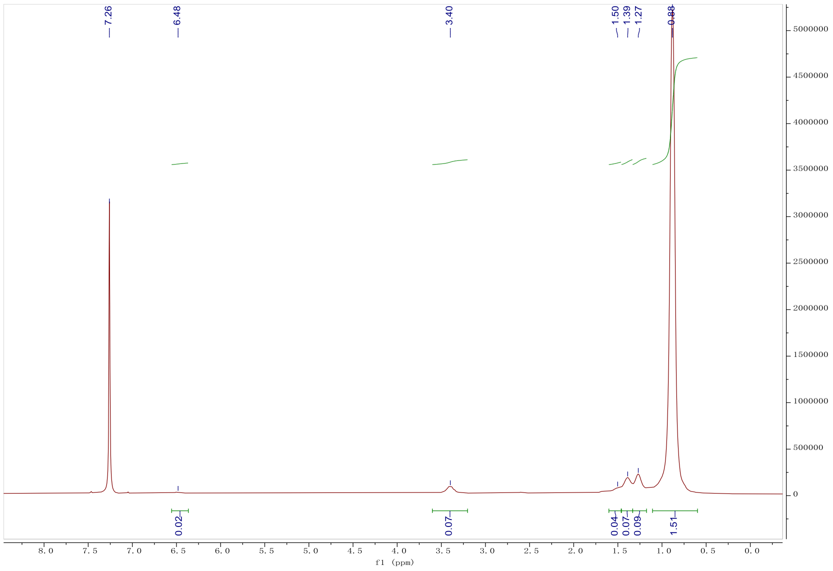

Supplementary Fig. 43. ^{1}H NMR and ^{2}D NMR of *d*-**3a**.

7.3 Crossover experiment

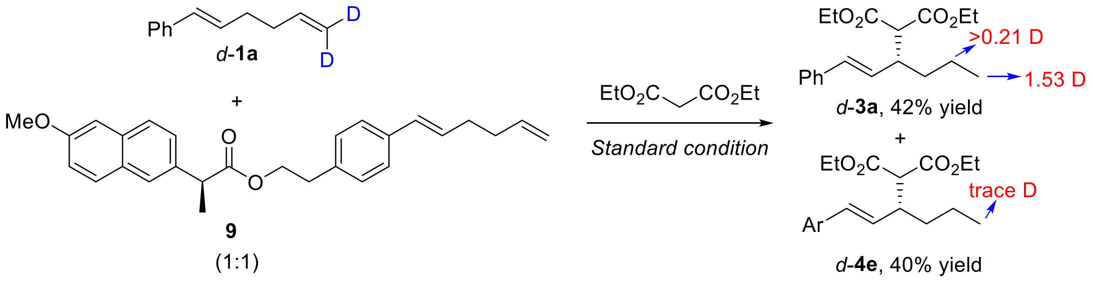

The procedure for crossover experiment with deuterated-**1a** and another diene **9** as competing electrophiles (Each electrophile was used in 0.055 mmol under standard condition) was the same as that described in the above part “General procedure for Pd-catalyzed migratory allylic substitution”. The allylation product *d*-**3a** and *d*-**4e** were isolated in 42% and 40% yield respectively.

The related ^{1}H NMR of isolated *d*-**3a** was shown as below.

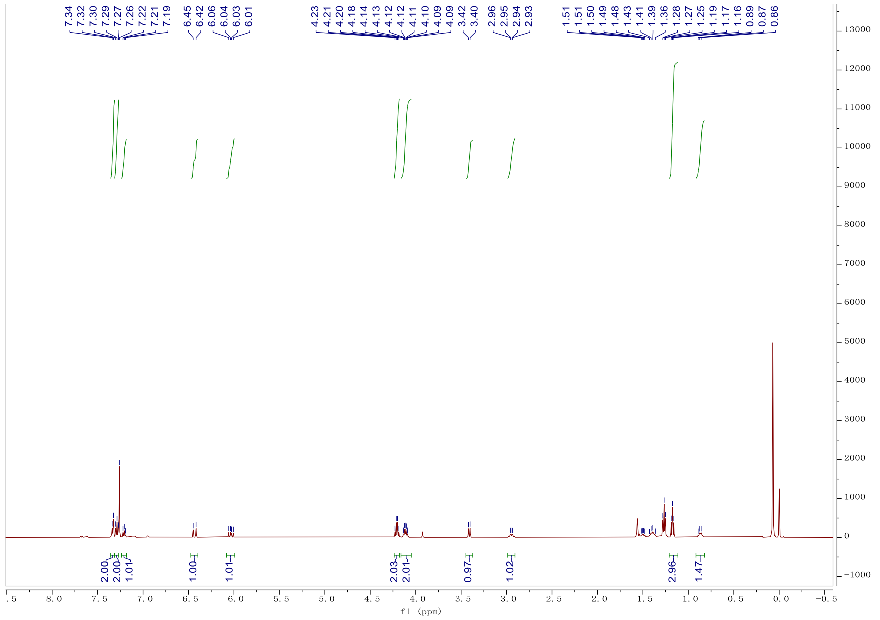

Supplementary Fig. 44. ^{1}H NMR of *d*-**3a**.

The related ^{2}D NMR of isolated *d*-**3a** was shown as below.

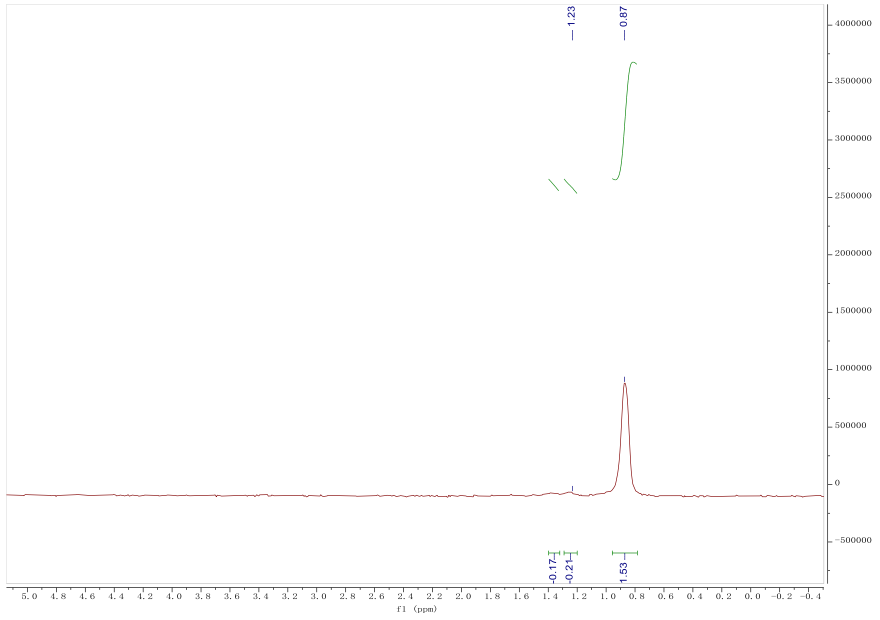

Supplementary Fig. 45. ^{2}D NMR of compound *d*-**3a**.

The related ^{2}D NMR of isolated *d*-**4e** was shown as below.

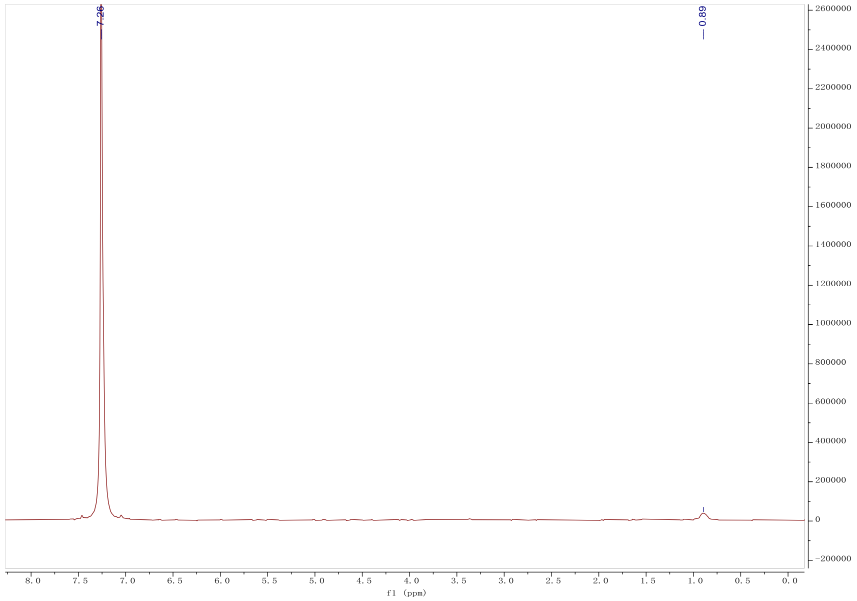

Supplementary Fig. 46. ^{2}D NMR of compound *d*-**4e**.

7.4 Kinetic isotope experiment

The non-deuteration experiment was conducted as the following procedure: To a 25 mL Schlenk tube in a N_2 box was added $[Pd(allyl)Cl]_2$ (4.6 mg, 0.013 mmol), **L9** (14 mg, 0.025 mmol), sodium tetrakis[3,5-bis(trifluoromethyl)phenyl]borate ($NaBAr^{F}_4$, 22 mg, 0.025 mmol) and dry Et_3N (5 mL). The mixture was then stirred at 25 °C for 5 min. Next, dodecane (0.050 mL, 38 mg, 0.22 mmol) and skipped diene **1a** (79 mg, 0.50 mmol) were added sequentially. After the mixture was stirred for 1 min, nucleophile **2a** (80 mg, 0.50 mmol) was added to the reaction. The reaction was stirred at 60 °C and analyzed by GC.

The deuteration experiment was conducted similarly as above, except that *d*-**2a** (81 mg, 0.50 mmol) was used instead.

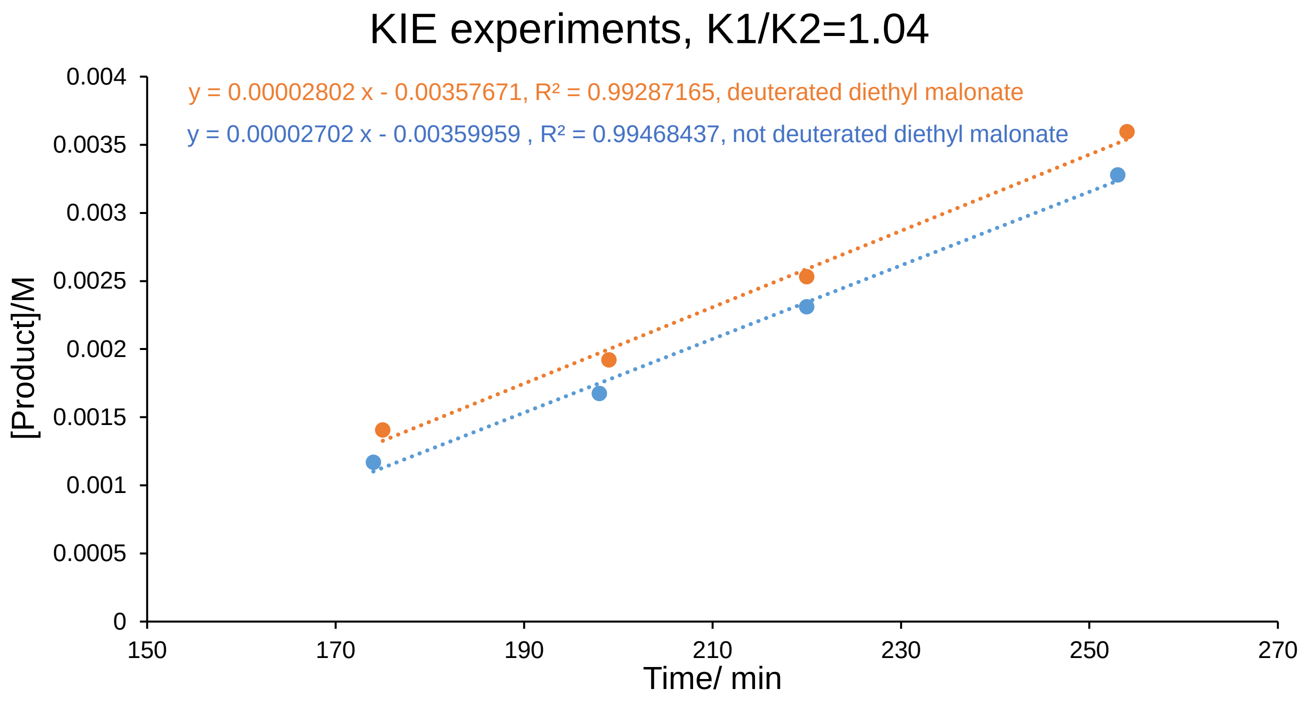

Supplementary Fig. 47. KIE test.

The KIE value was calculated as below:

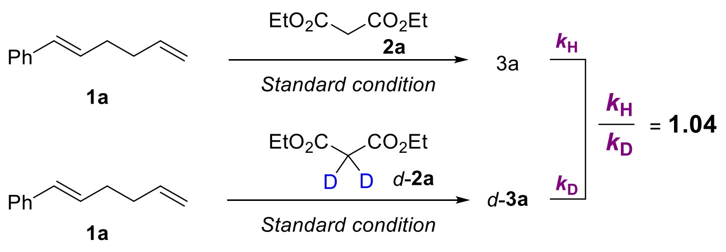

7.5 Kinetic studies

7.5.1 Kinetic order of nucleophile 2a

To a 25 mL Schlenk tube in a N_2 box was added $[Pd(allyl)Cl]_2$, **L9**, sodium tetrakis[3,5-bis(trifluoromethyl)phenyl]borate ($NaBAr^F_4$) and dry Et_3N. The mixture was then stirred at 25 °C for 5 min. Next, dodecane and skipped diene **1a** were added sequentially. After the mixture was stirred for 1 min, nucleophile **2a** was added to the reaction. The reaction was stirred at 60 °C and analyzed by GC along time. The detailed amount of each substrate was shown in the table below.

Supplementary Table 1. The amount of materials used for kinetic order test of **2a**.

Entry	Pd	**L9**	$NaBAr_4^F$	Et_3N	**1a**	**2a**	Dodecane

	mg	mg	mg	mL	mg	mg	mmol	c / mol*L^{-1}	mg
1	4.6	13.9	22.2	5	80	96.6	0.60	0.12	37.7
2	4.6	13.9	22.2	5	80	112.2	0.70	0.14	37.7
3	4.6	13.9	22.2	5	80	159.3	1.00	0.20	37.7

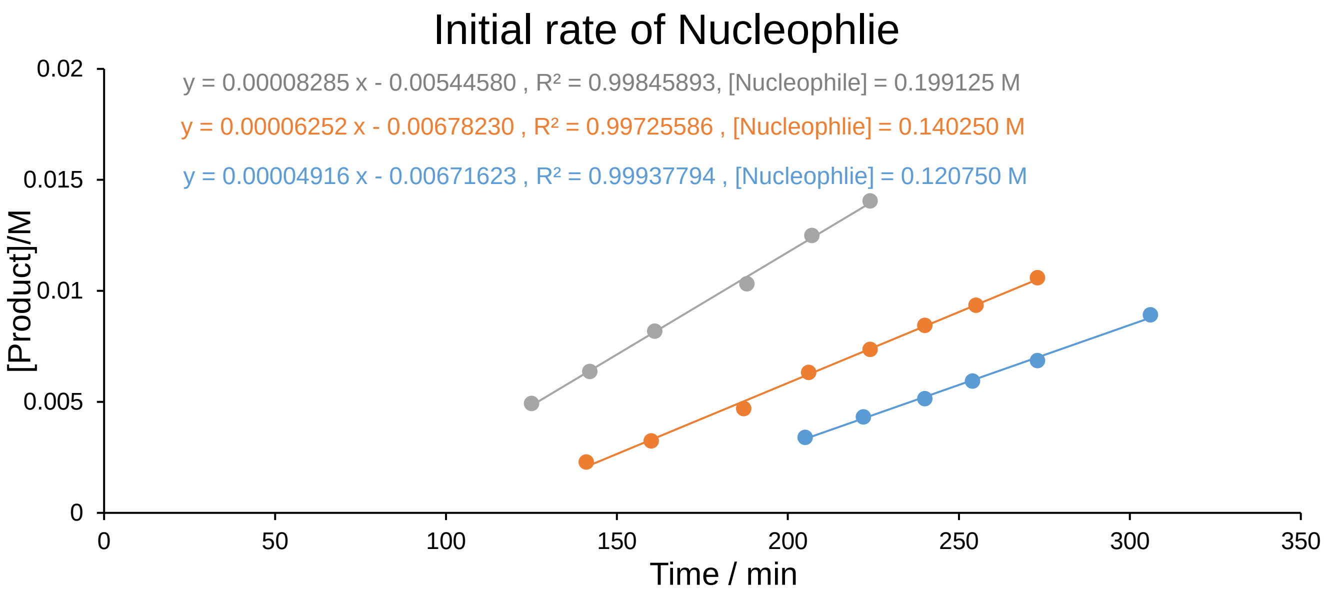

Supplementary Fig. 48. Plot of concentration of **3a** over time with reactions performed with varying concentration of **2a**

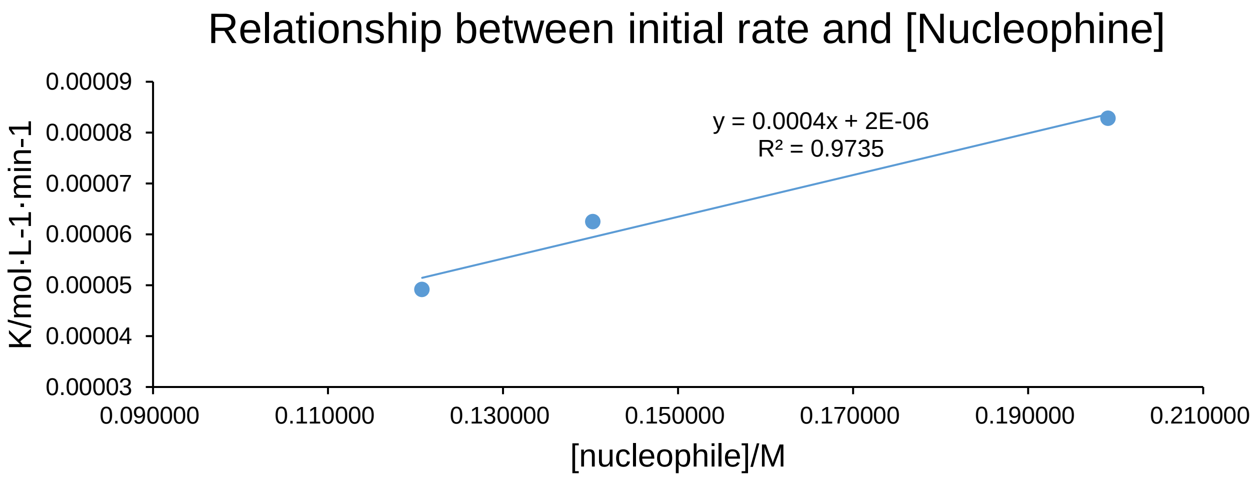

Supplementary Fig. 49. Plot of the initial rates of formation of **3a** vs. [**2a**] for the migratory allylation reaction.

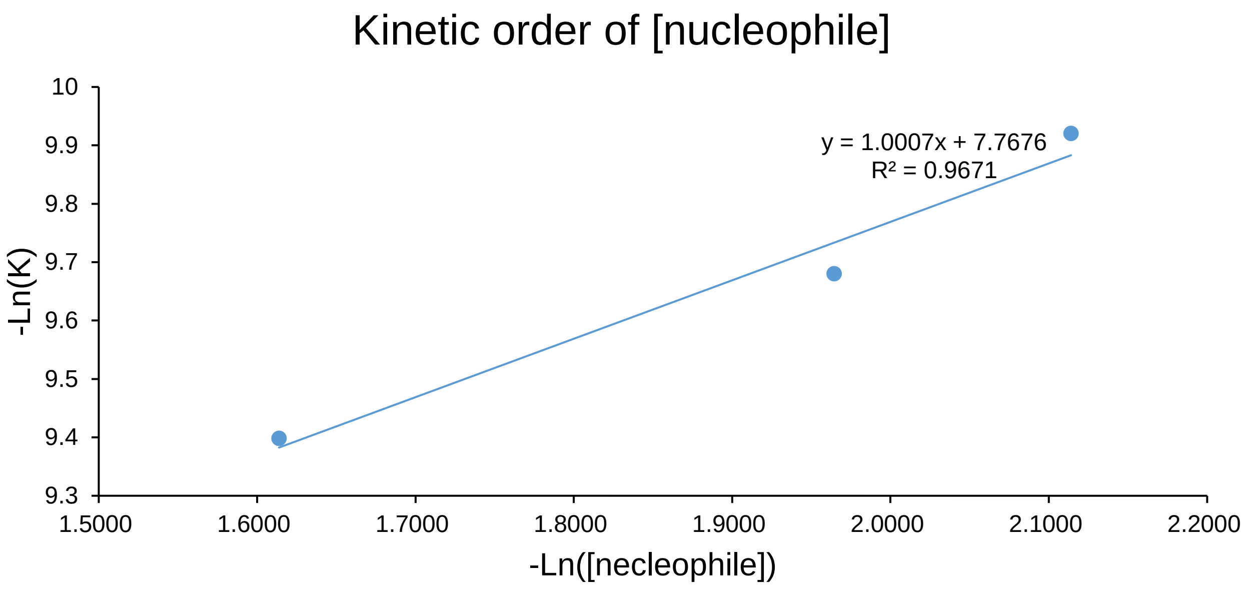

Supplementary Fig. 50. Plot of ln(Initial Rates) *vs.* ln(**2a**) for the migratory allylation reaction**.**

7.5.2 Kinetic order of catalyst

To a 25 mL Schlenk tube in a N_2 box was added $[Pd(allyl)Cl]_2$, **L9**, sodium tetrakis[3,5-bis(trifluoromethyl)phenyl]borate ($NaBAr^F_4$) and dry Et_3N. The mixture was then stirred at 25 °C for 5 min. Next, dodecane and skipped diene **1a** were added sequentially. After the mixture was stirred for 1 min, nucleophile **2a** was added to the reaction. The reaction was stirred at 60 °C and analyzed by GC along time. The detailed amount of each substrate was shown in the table below.

Supplementary Table 2. The amount of materials used for kinetic order test of catalyst.

Entry	Pd			**L9**			$NaBAr_4^F$			Et_3N	**1a**	**2a**	Dodecane
	mmol	mg	c / M	mmol	mg	c / M	mmol	mg	c / M	mL	mg	mg	mg
1	0.005	1.8	0.0010	0.01	5.5	0.002	0.01	8.9	0.002	5	79	80	37.7
2	0.01	3.7	0.0019	0.02	11.1	0.004	0.02	17.7	0.004	5	79	80	37.7
3	0.015	5.5	0.0029	0.03	16.6	0.006	0.03	26.6	0.006	5	79	80	37.7
4	0.02	7.3	0.0038	0.04	22.2	0.008	0.04	35.4	0.008	5	79	80	37.7
5	0.025	9.1	0.0048	0.05	27.7	0.010	0.05	44.3	0.010	5	79	80	37.7

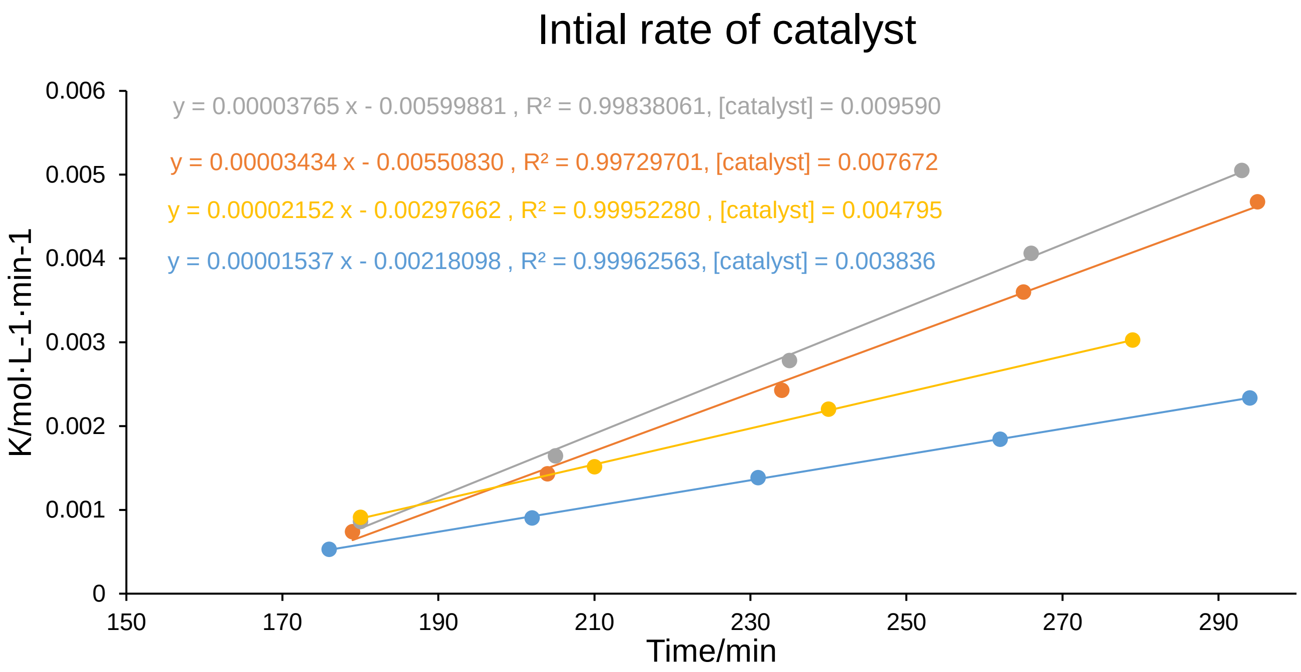

Supplementary Fig. 51. Plot of concentration of **3a** over time with reactions performed with varying concentration of catalyst.

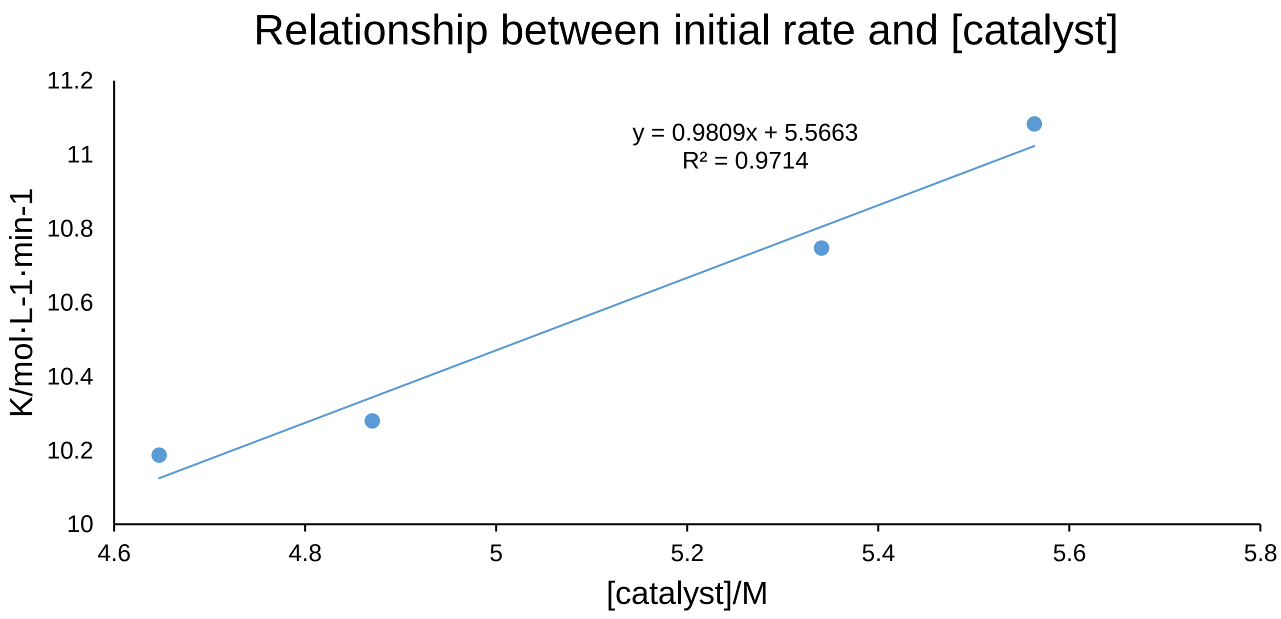

Supplementary Fig. 52. Plot of the initial rates of formation of **3a** vs. [catalyst] for the migratory allylation reaction.

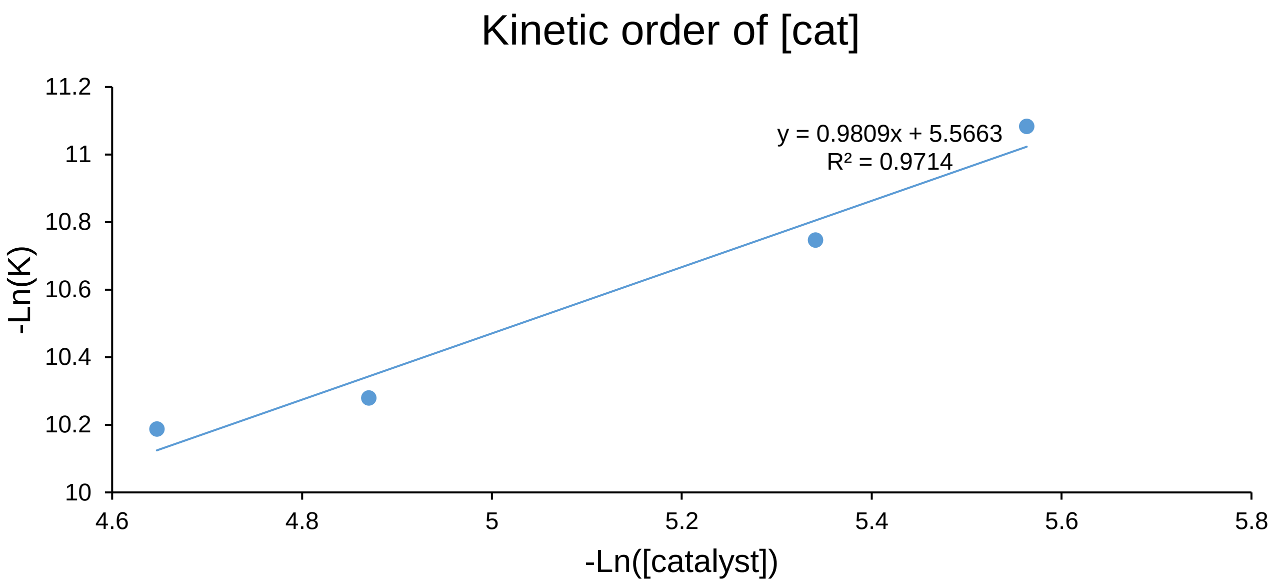

Supplementary Fig. 53. Plot of ln(Initial Rates) *vs.* ln(catalyst) for the migratory allylation reaction.

7.5.3 Kinetic studies on diene 1a

To a 25 mL Schlenk tube in a N_2 box was added $[Pd(allyl)Cl]_2$, **L9**, sodium tetrakis[3,5-bis(trifluoromethyl)phenyl]borate ($NaBAr^F{}_4$) and dry Et_3N. The mixture was then stirred at 25 °C for 5 min. Next, dodecane and skipped diene **1a** were added sequentially. After the mixture was stirred for 1 min, nucleophile **2a** was added to the reaction. The reaction was stirred at 60 °C and analyzed by GC along time. The detailed amount of each substrate was shown in the table below.

Supplementary Table 3. The amount of each material used for kinetic order test of **1a**.

Entry	Pd	**L9**	$NaBAr_4^F$	Et_3N	**1a**			**2a**	dodecane	Additional Et_3N/ mL	Total volume/ mL
	mg	mg	mg	mL	mmol	mg	c / M	mg	mg		
1	4.6	13.9	22.2	5	0.60	95.1	0.108	79	37.7	0.251	5.487
2	4.6	13.9	22.2	5	1.00	157.8	0.179	79	37.7	0.180	5.487
3	4.6	13.9	22.2	5	1.20	190.2	0.216	79	37.7	0.143	5.487
4	4.6	13.9	22.2	5	1.50	237.6	0.270	79	37.7	0.089	5.487
5	4.6	13.9	22.2	5	2.00	316.6	0.359	79	37.7	0.000	5.487

The initial kinetic data of **1a** fits perfectly with Michaelis-Menten kinetic model as shown below:

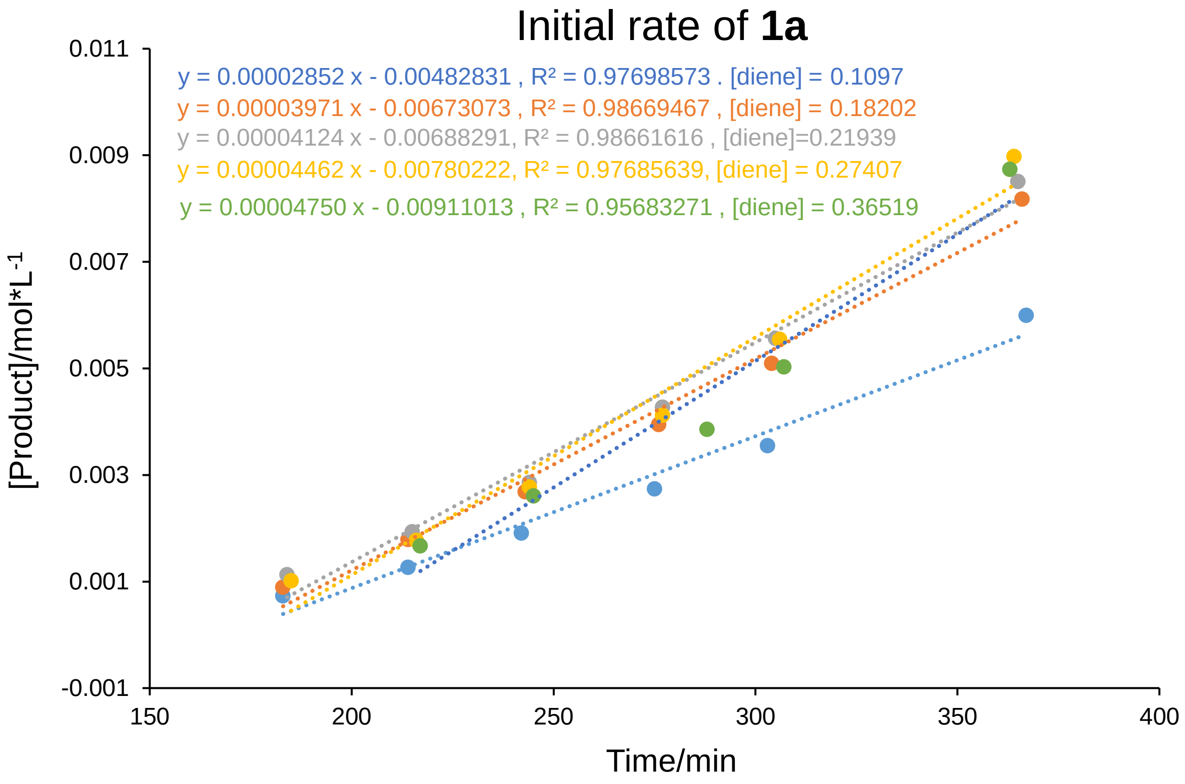

Supplementary Fig. 54. Plot of concentration of **3a** over time with reactions performed with varying concentration of **1a**.

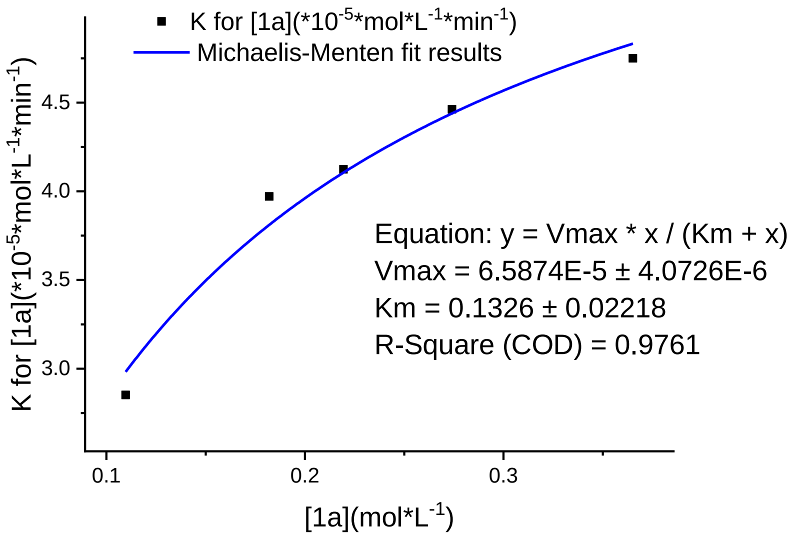

Supplementary Fig. 55. Plot of the initial rates of formation of **3a** vs. [**1a**] for the migratory allylation reaction.

Base on the Michaelis-Menten equation, the relationship of 1/k with 1/[**1a**] should be linear, consistent with our detected data below:

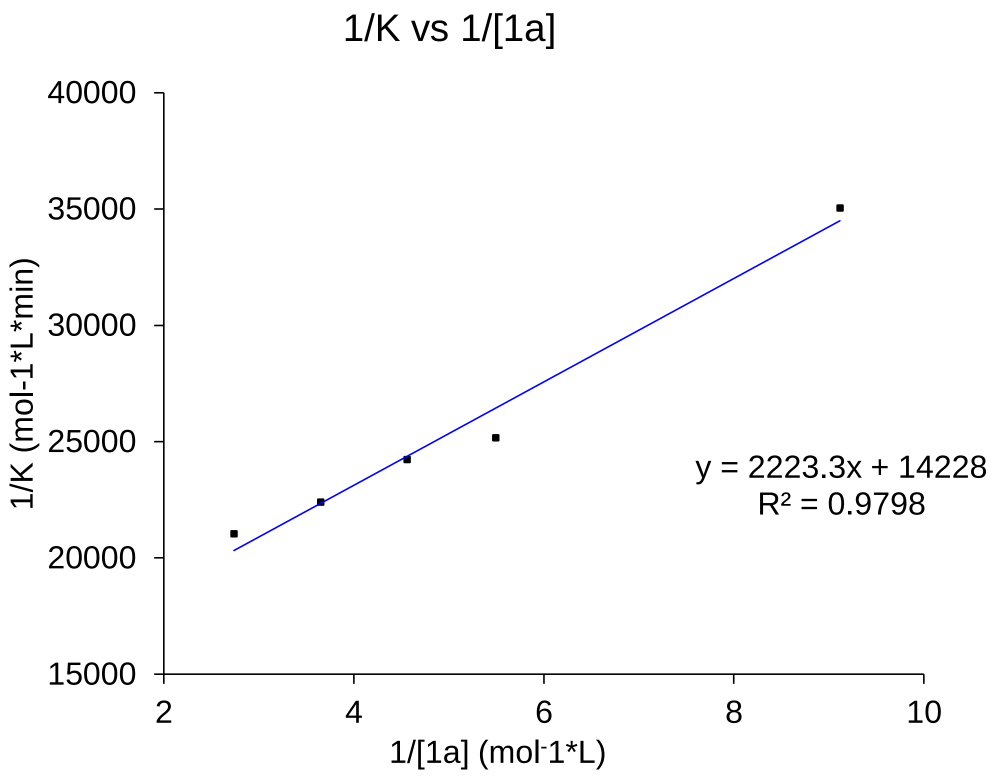

Supplementary Fig. 56. Plot of [Initial Rates]$^{-1}$ *vs.* [**1a**]$^{-1}$ for the migratory allylation reaction**.**

7.6 Determination of initial rate equation

If the allylic substitution step was the rate-determining step, then

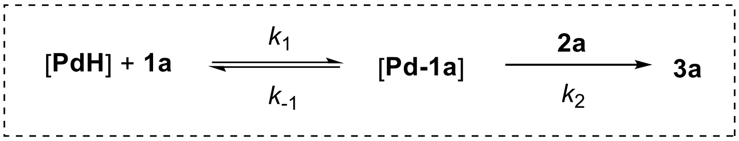

$$\frac{\mathrm{d[Pd-1a]}}{\mathrm{dt}} = k_1[\mathbf{PdH}][\mathbf{1}\boldsymbol{a}] - k_{-1}[\mathbf{Pd-1a}] - k_2[\mathbf{Pd-1a}][\mathbf{2a}] = 0$$

Based on the Steady-State Approximation (SSA) theory:

Also $$[\mathbf{PdH}] = [\mathbf{cat}] - [\mathbf{Pd-1a}]$$

So $$[\mathbf{Pd-1a}] = \frac{k_1[\mathbf{cat}][\mathbf{1a}]}{k_1[\mathbf{1a}]+k_1[\mathbf{1a}]+k_{-1}}$$

Finally $$initial\ rate = \frac{d[\mathbf{3a}]}{dt} = k_2[\mathbf{Pd-1a}][\mathbf{2a}] = \frac{k_1k_2[\mathbf{cat}][\mathbf{1a}][\mathbf{2a}]}{k_1[\mathbf{1a}]+k_2[\mathbf{1a}]+k_{-1}}$$

As $$k_2 \ll k_1 \sim k_{-1}$$

So $$initial\ rate \approx \frac{k_1k_2[\mathbf{cat}][\mathbf{1a}][\mathbf{2a}]}{k_1[\mathbf{1a}]+k_{-1}} = K_{obs}[\mathbf{cat}][\mathbf{2a}]\frac{[\mathbf{1a}]}{k_1[\mathbf{1a}]+k_{-1}}$$

- 1st order on **[cat]**
- 1st order on **[2a]**
- saturation kinetics on **[1a]**

The deduced equation above was perfectly consistent with the kinetic studies. Thus, combining with the KIE data, we proposed the rate-determining step was the allylic substitution.

On the other hand, the observed kinetic data for **1a** with a perfect fitting by Michaelis-Menten kinetic model also demonstrated the above conclusion.

7.7 Induction period detection

To a 25 mL Schlenk tube in a N_2 box was added $[Pd(allyl)Cl]_2$ (4.6 mg, 0.013 mmol), L (14 mg, 0.025 mmol), sodium tetrakis[3,5-bis(trifluoromethyl)phenyl]borate ($NaBAr^F{}_4$, 22 mg, 0.025 mmol) and dry Et_3N (5 mL). The mixture was then stirred at 25 °C for 5 min. Next, dodecane (38 mg, 0.22 mmol) and skipped diene **1a** (79 mg, 0.50 mmol) were added sequentially. After the mixture was stirred for 1 min,

skipped diene **1a** (79 mg, 0.50 mmol) were added sequentially. After the mixture was stirred for 1 min, nucleophile **2a** was added to the reaction. The reaction was stirred at 60 ºC and analyzed by GC along time.

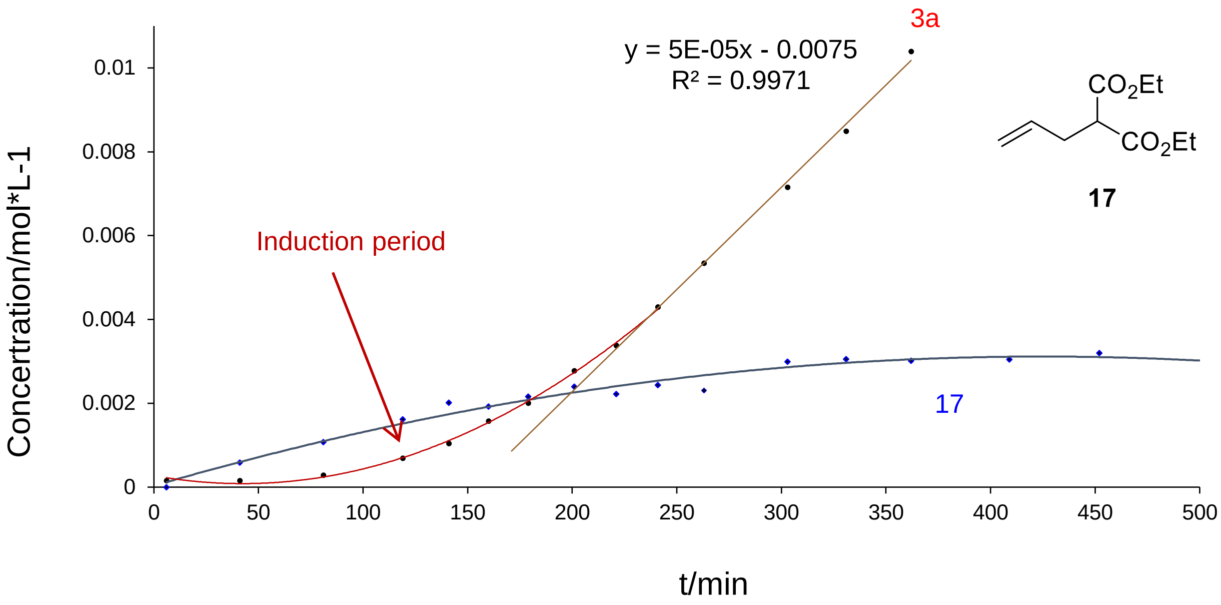

Supplementary Fig. 57. Kinetic data for the formation of **3a** and **17**.

17 generated from the allylic substitution of nucleophile with $[Pd(allyl)Cl]_2$ is a known compound (ref *J. Am. Chem. Soc.* **2009**, *131*, 8772–8774).

8. Regioconvergent synthesis, gram-scale test and transformations of allylation products

8.1 Regioconvergent synthesis

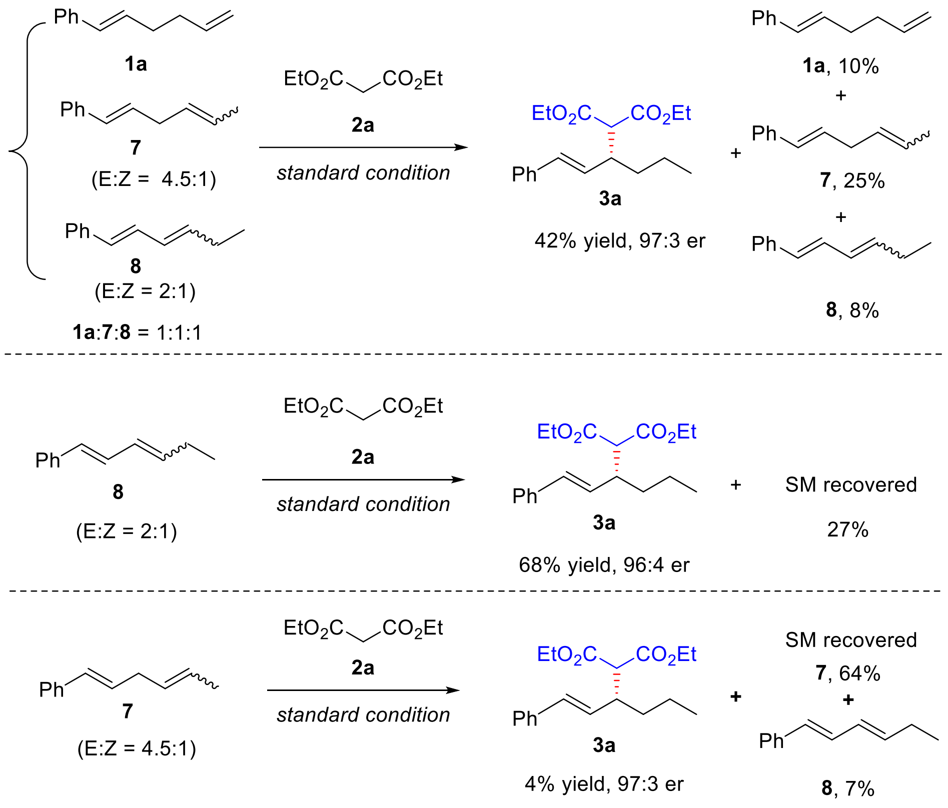

Supplementary Fig. 58. Regioconvergent synthesis.

The procedures of these experiment were similar as that described in part 4 titled "General procedure for Pd-catalyzed migratory allylic functionalization".

8.2 Gram-scale test

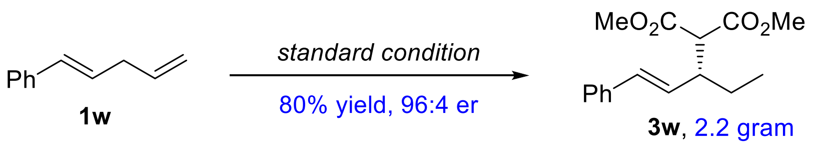

Procedure for the gram-scale test: To a 50 mL Schlenk tube in the glovebox under nitrogen were added [Pd(allyl)Cl]$_2$ (90 mg, 0.25 mmol), **L** (0.28 g, 0.50 mmol), sodium tetrakis[3,5-bis(trifluoromethyl)phenyl]borate (NaBAr$^F{}_4$, 0.44 g, 0.50 mmol) and dry Et$_3$N (10 mL). The mixture was stirred at room temperature for 5 min. Then the skipped diene **1** (1.4 g, 10 mmol) was added to the solution and the reaction continued to stir for 1 min. Finally, the nucleophile **2** (1.5 g, 10 mmol) was added to the reaction. The resulting mixture was stirred at 60 ^oC for 24 h. After this time, the reaction solution was

cooled to room temperature and then condensed. The crude ^{1}H NMR was obtained with dibromomethane (0.7 mL, 10 mmol) as internal standard to help determine the regioselectivity and conversion. The reaction was further purified by flash column chromatography to afford the pure allylation product **3w** (2.2 g) as a colorless oil in 80% yield with 96:4 er.

8.3 Transformations of allylation products

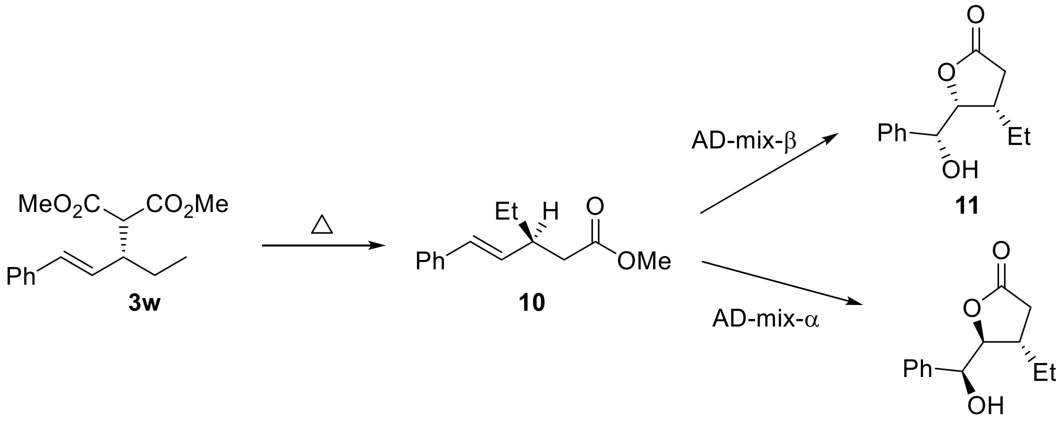

Methyl (*S,E*)-3-ethyl-5-phenylpent-4-enoate (10): To a 10 mL flask was added **3w** (0.28 g, 1.0 mmol), NaCl (0.17 g, 3.0 mmol), DMSO (1.5 mL) and H_2O (1.0 mL). The mixture was stirred at 180 °C under reflux for 8 h. After **3w** was fully consumed, the solution was cooled to room temperature, exacted by ethyl acetate (5 mL × 3), dried by anhydrous $NaSO_4$, and purified by flash column chromatography to afford the pure product **10** (0.17 g) as a colorless oil in 76% yield. ^{1}H NMR (500 MHz, chloroform-*d*) δ 7.38 – 7.32 (m, 2H), 7.30 (dd, *J* = 8.5, 6.8 Hz, 2H), 7.24 – 7.17 (m, 1H), 6.42 (d, *J* = 15.8 Hz, 1H), 6.01 (dd, *J* = 15.8, 8.7 Hz, 1H), 3.65 (s, 3H), 2.68 – 2.57 (m, 1H), 2.51 – 2.44 (m, 1H), 2.43 – 2.34 (m, 1H), 1.61 – 1.49 (m, 1H), 1.49 – 1.36 (m, 1H), 0.93 (t, *J* = 7.4 Hz, 3H). ^{13}C NMR (126 MHz, chloroform-*d*) δ = 173.0, 137.5, 132.7, 130.6, 128.5, 127.2, 126.2, 51.5, 41.6, 40.0, 27.8, 11.7. HRMS (ESI): [M+H]$^{\oplus}$ calcd for $C_{14}H_{19}O_2^{\oplus}$ 219.1380, found 219.1380.

(*4S,5R*)-4-Ethyl-5-((*R*)-hydroxy(phenyl)methyl)dihydrofuran-2(3*H*)-one (**11**): Compound **11** was prepared according to a reported literature[12]: To a 25 mL flask was added AD-mix *α* (0.78 g, 1.0 mmol), $MeSO_2NH_2$ (38 mg, 0.4 mmol) and *t*-BuOH/H_2O (1 mL/1 mL). **10** (0.87 g, 0.40 mmol) was then added to the reaction. The mixture was allowed to stir vigorously at 25 °C for 16 h. Next, sodium sulfite (0.30 g) was added and the mixture continued to stir at room temperature for 1 h. The reaction was quenched by aqueous KOH (2 M, 2 mL), extracted by EtOAc (5 mL × 3), dried by anhydrous $MgSO_4$, filtered, condensed and purified by flash column chromatography to give **11** (55 mg) in 62% yield as a white solid. $[\alpha]_D^{25}$ -150.4 (*c* 0.69, $CHCl_3$). ^{1}H NMR (500 MHz, Chloroform-*d*) δ 7.42 – 7.27 (m, 5H), 4.86 (t, *J* = 3.2 Hz, 1H), 4.57 (dd, *J* = 6.9, 2.8 Hz, 1H), 2.60 – 2.45 (m, 3H), 2.41 (d, *J* = 3.8 Hz, 1H), 1.80 – 1.61 (m, 2H), 0.98 (t, *J* = 7.4 Hz, 3H). ^{13}C NMR (126 MHz, $CDCl_3$) δ = 177.5, 140.3, 128.7, 128.4, 127.0, 84.8, 73.2, 40.4, 34.4, 22.1, 12.7. HRMS (ESI): [M+Na]$^{\oplus}$ calcd for $C_{13}H_{16}O_3Na^{\oplus}$ 243.0992, found 243.0992.

(*4S,5R*)-4-Ethyl-5-((*R*)-hydroxy(phenyl)methyl)dihydrofuran-2(3*H*)-one (**12**): The synthesis of **12** was the same as that for **11**, except that AD-mix *β* (0.47 g, 0.60 mmol), $MeSO_2NH_2$ (19 mg, 0.20 mmol), **10** (44 mg, 0.20 mmol) was used instead. The compound **12** was obtained in 84% yield (37 mg) as a colorless oil. $[\alpha]_D^{25}$ +58.1 (*c* 0.38, $CHCl_3$). ^{1}H NMR (500 MHz, chloroform-*d*) δ 7.46 – 7.31 (m, 5H), 4.70

(d, J = 5.5 Hz, 1H), 4.29 (t, J = 5.6 Hz, 1H), 2.71 – 2.57 (m, 1H), 2.54 (s, 1H), 2.45 – 2.32 (m, 1H), 2.21 – 2.13 (m, 1H), 1.24 – 1.06 (m, 4H), 0.78 (t, J = 7.0 Hz, 3H). ^{13}C NMR (126 MHz, chloroform-*d*) δ = 176.4, 138.8, 128.8, 128.8, 127.1, 88.3, 77.4, 77.1, 76.8, 75.8, 36.3, 35.9, 34.9, 20.3, 13.7. HRMS (ESI): [M+Na]$^{\oplus}$ calcd for $C_{13}H_{16}O_3Na^{\oplus}$ 243.0992, found 243.0992.

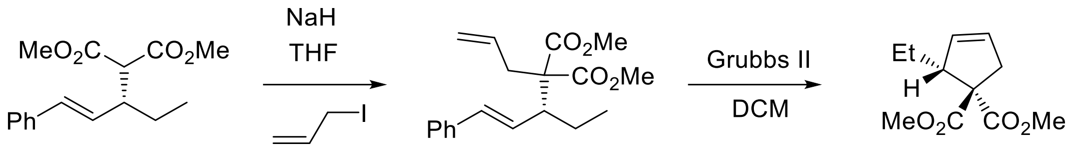

3w **13** **14**

Dimethyl (*R*,*E*)-2-allyl-2-(1-phenylpent-1-en-3-yl)malonate (**13**): To a suspension of NaH (60% wt in mineral oil, 30 mg, 0.75 mmol) in THF (2 mL) at 0 ºC under N_2 was added dimethyl malonate **3w** (0.14 g, 0.50 mmol) in 20 minutes. Then allyl iodine (0.71 g, 2.5 mmol) was added dropwise. The resulting solution was stirred at room temperature overnight. After this time, the reaction was quenched with saturated aqueous NH_4Cl solution (5 mL), extracted with ethyl acetate (5 mL × 3) and purified by flash column chromatography to give **13** (142 mg) as a colorless oil in 90% yield. ^{1}H NMR (500 MHz, chloroform-*d*) δ 7.36 (d, J = 7.0 Hz, 2H), 7.30 (t, J = 7.5 Hz, 2H), 7.22 (t, J = 7.3 Hz, 1H), 6.42 (d, J = 15.7 Hz, 1H), 5.93 (dd, J = 15.7, 10.2 Hz, 1H), 5.85 – 5.73 (m, 1H), 5.09 – 5.06 (m, 1H), 5.05 (s, 1H), 3.73 (d, J = 3.4 Hz, 6H), 2.71 – 2.58 (m, 3H), 1.82 – 1.73 (m, 1H), 1.28 – 1.22 (m, 1H), 0.89 (t, J = 7.3 Hz, 3H). ^{13}C NMR (126 MHz, $CDCl_3$) δ = 171.2, 170.9, 137.2, 133.8, 133.3, 128.58, 128.57, 127.4, 126.4, 118.6, 77.4, 77.1, 76.8, 62.0, 52.1, 52.1, 49.9, 39.2, 24.0, 12.7. HRMS (ESI): [M+H]$^{\oplus}$ calcd for $C_{19}H_{24}O_4{}^{\oplus}$ 339.1567, found 339.1567.

Dimethyl (*R*)-2-ethylcyclopent-3-ene-1,1-dicarboxylate (**14**): Diene **13** (67 mg, 0.20 mmol) was dissolved in CH_2Cl_2 (4 ml) and Grubbs 2nd generation catalyst (9 mg, 0.007 mmol) was added. The mixture was stirred at room temperature for 1 hour. Next, DMSO (0.1mL) was added and the resulting solution continued to stir for another one hour. After this time, the reaction was condensed and purified by flash column chromatography (hexane: ethyl acetate = 20:1) to afford **14** (27 mg) in 91% as a colorless oil. $[\alpha]_D^{25}$ -179.7 (*c* 0.15, $CHCl_3$) for 96:4 er; ^{1}H NMR (500 MHz, chloroform-*d*) δ 5.78 – 5.72 (m, 1H), 5.67 – 5.61 (m, 1H), 3.72 (s, 3H), 3.71 (s, 3H), 3.44 – 3.38 (m, 1H), 3.27 – 3.21 (m, 1H), 2.78 – 2.70 (m, 1H), 1.50 – 1.42 (m, 1H), 1.20 – 1.09 (m, 1H), 0.94 (t, J = 7.4 Hz, 3H). ^{13}C NMR (126 MHz, chloroform-*d*) δ = 172.9, 171.1, 132.3, 127.3, 77.4, 77.1, 76.8, 63.5, 52.8, 52.3, 52.1, 40.1, 24.0, 12.2. HRMS (EI): [M]$^{\oplus}$ calcd for $C_{11}H_{16}O_4{}^{\oplus}$ 212.1043, found 212.1045. The enantiomeric ratio was determined by chiral GC using CP-ChiraSil-DEX (J&W CP7502) (25 m×0.25 mm×0.25 um) column (N_2 carrier gas, initial flow: 1.0 ml/min; initial temp. 90 ºC, hold 50 min, ramp 10 ºC/min to 200 ºC, hold 5 min). Retention time: 32.7 min (major), 33.9 min (minor).

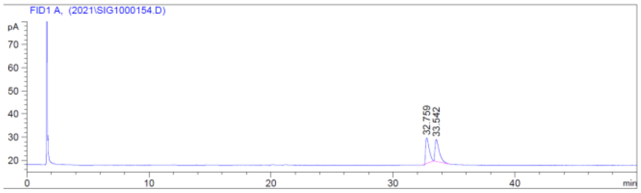

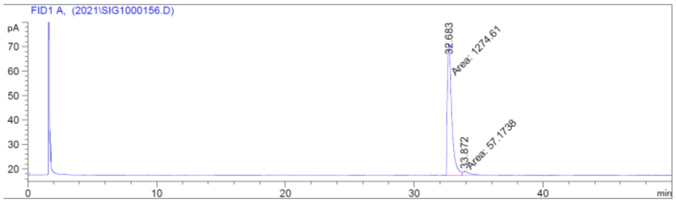

Peak #	RetTime [min]	Type	Width [min]	Area [pA*s]	Height [pA]	Area %
1	32.759	BB	0.3060	220.02219	10.97005	49.39639
2	33.542	BB	0.3601	225.39941	9.38839	50.60361

Peak #	RetTime [min]	Type	Width [min]	Area [pA*s]	Height [pA]	Area %
1	32.683	MM	0.3957	1274.61011	53.67958	95.70698
2	33.872	MM	0.6013	57.17379	1.58460	4.29302

Supplementary Fig. 59. HPLC data of compound **14**.

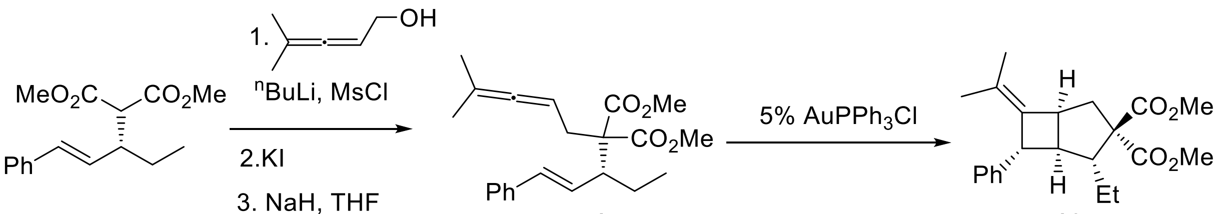

3w **15** **16**

Dimethyl (*R*,*E*)-2-(4-methylpenta-2,3-dien-1-yl)-2-(1-phenylpent-1-en-3-yl)malonate (**15**): To a dry 25mL Schlenk tube charged with a stir bar was added 4-methylpenta-2,3-dien-1-ol (98 mg, 1.0 mmol) and dry THF (5 mL) at -78 °C. Then ^{n}BuLi (2.5 M in hexane, 0.44 mL) was added dropwise to the reaction above under N_2 and the resulting mixture continued to stir for 30 min. Then methanesulfonyl chloride (0.11 g, 1.0 mmol) was added and the reaction stirred at -78 °C for another 30 min. Next, KI (0.83 g, 5.0 mmol) was added. The mixture was stirred at room temperature for 12 h. Meanwhile, to the second vial with a suspension of NaH (60% wt in mineral oil, 22 mg, 0.55 mmol.) in THF (5 ml) at 0 °C under N_2 was added **3w** (138 mg, 0.500 mmol) dropwise. After 30 min, the freshly prepared solution of the first vial was added dropwise to the second one. The mixture continued to stir at 0 °C for 24 h. The reaction was quenched with sat. NH_4Cl aqueous solution, extracted with ethyl acetate (5 mL × 3), condensed and purified by flash column chromatography to give **15** (142 mg) as a colorless oil in 80% yield. ^{1}H NMR (500 MHz, chloroform-*d*) δ 7.38 – 7.27 (m, 5H), 7.25 – 7.18 (m, 1H), 6.43 (d, *J* = 15.7 Hz, 1H), 5.95 (dd, *J* = 15.8, 10.1 Hz, 1H), 4.92 (tt, *J* = 7.4, 2.8 Hz, 1H), 3.73 (d, *J* = 2.4 Hz, 6H), 2.75 – 2.67 (m, 1H), 2.63 – 2.55 (m, 2H), 1.86 – 1.76 (m, 1H), 1.65 (t, *J* = 2.6 Hz, 6H), 1.29 – 1.16 (m, 1H), 0.89 (t, *J* = 7.3 Hz, 3H). ^{13}C NMR (126 MHz, chloroform-*d*) δ = 203.6, 171.1, 171.0, 137.3, 133.6, 128.9, 128.6, 127.4, 126.4, 95.0, 83.7, 62.1, 52.2, 52.1, 49.6, 35.0, 23.9, 20.6, 20.6, 12.8. HRMS (ESI): $[M+H]^{\oplus}$ calcd for $C_{22}H_{29}O_4^{\oplus}$ 357.2060, found 357.2060.

Dimethyl (1*S*,2*R*,5*S*,7*R*)-2-ethyl-7-phenyl-6-(propan-2-ylidene)bicyclo[3.2.0]heptane-3,3-dicarboxylate (**16**): To a 4 mL vial was added Ph_3AuCl (2.5 mg, 5.0 mol%), $AgBF_4$ (1.0 mg, 5.0 mol%) and dry CH_2Cl_2 (1 mL) under nitrogen. The mixture was stirred at room temperature for 5 minutes. Then the solution was transferred to another 4 mL vial with **15** (32 mg, 0.10 mmol) in CH_2Cl_2 (1 mL). The reaction was stirred at room temperature and monitored by TLC analysis. The reaction was filtered through a short silica plug and eluted with CH_2Cl_2, condensed and purified by flash column chromatography to obtain **16** as a colorless oil (22 mg) in 72% yield. $[\alpha]_D^{25}$ +12.7 (*c* 1.1, $CHCl_3$). ^{1}H NMR (400 MHz, chloroform-*d*) δ 7.32 – 7.26 (m, 2H), 7.23 – 7.12 (m, 3H), 3.73 (s, 1H), 3.71 (s, 3H), 3.68 (s, 3H), 3.59 (s, 1H), 2.80 – 2.69 (m, 1H), 2.58 (dd, *J* = 13.5, 8.4 Hz, 1H), 2.32 – 2.21 (m, 2H), 1.59 (s, 3H), 1.31 (d, *J* = 1.6 Hz, 3H), 1.07 – 0.95 (m, 1H), 0.92 (t, *J* = 7.0 Hz, 3H), 0.89 – 0.83 (m, 1H). ^{13}C NMR (126 MHz, chloroform-*d*) δ = 172.1, 171.5, 144.9, 135.8, 128.6, 128.5, 127.2, 125.8, 66.9, 53.8, 52.4, 52.2, 52.1, 49.8, 43.0, 39.0, 24.7, 19.0, 18.8, 13.1. HRMS (ESI): $[M+H]^{\oplus}$ calcd for $C_{22}H_{29}O_4^{\oplus}$ 357.2060, found 357.2060.

9. X-ray crystal structure of compound 11

The X-ray crystallographic data for **11** (CCDC 2076792) have been deposited at the Cambridge Crystallographic Data Center (www.ccdc.cam.ac.uk/data_request/cif).

The data was collected by using copper irradiation source for the determination of absolute configuration of **11**.

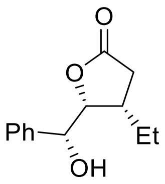

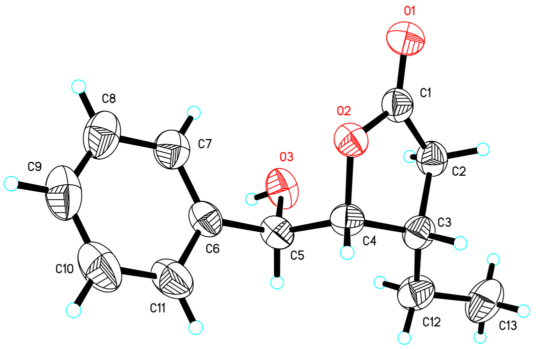

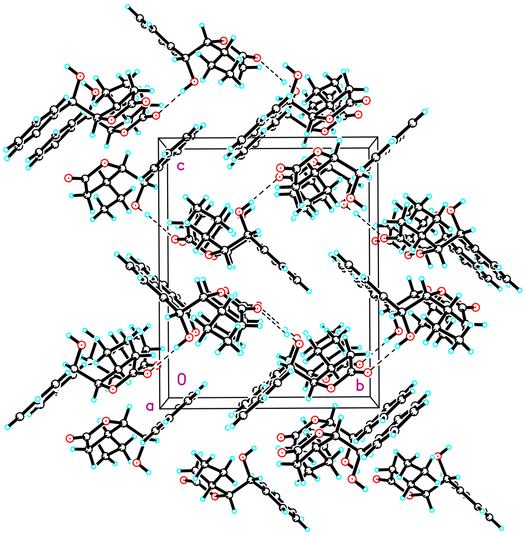

Table 1. Crystal data and structure refinement for **11**.

Identification code	**11**
Empirical formula	C13 H16 O3
Formula weight	220.26

Temperature	293(2) K	
Wavelength	1.54178 Å	
Crystal system	Orthorhombic	
Space group	P 21 21 21	
Unit cell dimensions	a = 7.9175(2) Å	a= 90°.
	b = 11.1634(2) Å	b= 90°.
	c = 13.7663(3) Å	g = 90°.
Volume	1216.75(5) $Å^3$	
Z	4	
Density (calculated)	1.202 Mg/m^3	
Absorption coefficient	0.687 mm^{-1}	
F(000)	472	
Crystal size	0.200 x 0.150 x 0.120 mm^3	
Theta range for data collection	7.561 to 70.113°.	
Index ranges	-8<=h<=9, -13<=k<=12, -14<=l<=16	
Reflections collected	7620	
Independent reflections	2245 [R(int) = 0.0305]	
Completeness to theta = 67.679°	96.8 %	
Absorption correction	Semi-empirical from equivalents	
Max. and min. transmission	0.7533 and 0.5542	
Refinement method	Full-matrix least-squares on F^2	
Data / restraints / parameters	2245 / 0 / 147	
Goodness-of-fit on F^2	1.066	
Final R indices [I>2sigma(I)]	R1 = 0.0463, wR2 = 0.1253	
R indices (all data)	R1 = 0.0476, wR2 = 0.1274	
Absolute structure parameter	-0.11(9)	
Extinction coefficient	n/a	
Largest diff. peak and hole	0.133 and -0.101 e.$Å^{-3}$	

Table 2. Atomic coordinates (x 10^4) and equivalent isotropic displacement parameters ($Å^2$x 10^3) for **11**. U(eq) is defined as one third of the trace of the orthogonalized U^{ij} tensor.

	x	y	z	U(eq)
O(1)	4480(2)	535(2)	6319(2)	79(1)
O(2)	5627(2)	2174(1)	5709(1)	61(1)
O(3)	5902(3)	3618(2)	7350(1)	78(1)
C(1)	5635(3)	1232(2)	6313(2)	62(1)
C(2)	7228(3)	1213(2)	6897(2)	66(1)
C(3)	8374(3)	2089(2)	6361(2)	63(1)
C(4)	7096(3)	2927(2)	5871(2)	59(1)
C(5)	6561(3)	4026(2)	6449(2)	64(1)
C(6)	5309(3)	4794(2)	5887(2)	65(1)
C(7)	3592(4)	4631(3)	5988(2)	75(1)
C(8)	2472(4)	5329(3)	5463(2)	90(1)
C(9)	3053(6)	6186(3)	4832(3)	101(1)
C(10)	4766(7)	6343(3)	4716(3)	108(1)
C(11)	5909(5)	5654(3)	5240(2)	91(1)
C(12)	9734(4)	2690(3)	6970(2)	81(1)
C(13)	10983(4)	1789(4)	7383(3)	105(1)

Table 3. Bond lengths [Å] and angles [°] for **11**.

O(1)-C(1)	1.201(3)
O(2)-C(1)	1.340(3)
O(2)-C(4)	1.452(3)
O(3)-C(5)	1.420(3)
O(3)-H(3)	0.8200
C(1)-C(2)	1.496(3)
C(2)-C(3)	1.525(3)
C(2)-H(2A)	0.9700
C(2)-H(2B)	0.9700
C(3)-C(12)	1.520(4)
C(3)-C(4)	1.535(3)
C(3)-H(3A)	0.9800
C(4)-C(5)	1.523(3)
C(4)-H(4)	0.9800
C(5)-C(6)	1.522(3)
C(5)-H(5)	0.9800
C(6)-C(7)	1.379(4)
C(6)-C(11)	1.392(4)
C(7)-C(8)	1.384(4)
C(7)-H(7)	0.9300
C(8)-C(9)	1.371(5)
C(8)-H(8)	0.9300
C(9)-C(10)	1.377(7)
C(9)-H(9)	0.9300
C(10)-C(11)	1.390(5)
C(10)-H(10)	0.9300
C(11)-H(11)	0.9300
C(12)-C(13)	1.521(4)
C(12)-H(12A)	0.9700
C(12)-H(12B)	0.9700
C(13)-H(13A)	0.9600
C(13)-H(13B)	0.9600
C(13)-H(13C)	0.9600
C(1)-O(2)-C(4)	110.79(17)
C(5)-O(3)-H(3)	109.5
O(1)-C(1)-O(2)	120.6(2)
O(1)-C(1)-C(2)	128.9(2)
O(2)-C(1)-C(2)	110.43(19)
C(1)-C(2)-C(3)	103.43(18)
C(1)-C(2)-H(2A)	111.1
C(3)-C(2)-H(2A)	111.1
C(1)-C(2)-H(2B)	111.1

C(3)-C(2)-H(2B)	111.1
H(2A)-C(2)-H(2B)	109.0
C(12)-C(3)-C(2)	115.9(2)
C(12)-C(3)-C(4)	116.1(2)
C(2)-C(3)-C(4)	102.19(17)
C(12)-C(3)-H(3A)	107.3
C(2)-C(3)-H(3A)	107.3
C(4)-C(3)-H(3A)	107.3
O(2)-C(4)-C(5)	108.92(18)
O(2)-C(4)-C(3)	104.04(17)
C(5)-C(4)-C(3)	116.40(18)
O(2)-C(4)-H(4)	109.1
C(5)-C(4)-H(4)	109.1
C(3)-C(4)-H(4)	109.1
O(3)-C(5)-C(6)	112.7(2)
O(3)-C(5)-C(4)	107.48(18)
C(6)-C(5)-C(4)	111.66(18)
O(3)-C(5)-H(5)	108.3
C(6)-C(5)-H(5)	108.3
C(4)-C(5)-H(5)	108.3
C(7)-C(6)-C(11)	119.5(3)
C(7)-C(6)-C(5)	121.1(2)
C(11)-C(6)-C(5)	119.4(2)
C(6)-C(7)-C(8)	120.3(3)
C(6)-C(7)-H(7)	119.8
C(8)-C(7)-H(7)	119.8
C(9)-C(8)-C(7)	120.5(3)
C(9)-C(8)-H(8)	119.7
C(7)-C(8)-H(8)	119.7
C(8)-C(9)-C(10)	119.5(3)
C(8)-C(9)-H(9)	120.2
C(10)-C(9)-H(9)	120.2
C(9)-C(10)-C(11)	120.7(4)
C(9)-C(10)-H(10)	119.7
C(11)-C(10)-H(10)	119.7
C(10)-C(11)-C(6)	119.5(3)
C(10)-C(11)-H(11)	120.3
C(6)-C(11)-H(11)	120.3
C(3)-C(12)-C(13)	112.0(3)
C(3)-C(12)-H(12A)	109.2
C(13)-C(12)-H(12A)	109.2
C(3)-C(12)-H(12B)	109.2
C(13)-C(12)-H(12B)	109.2
H(12A)-C(12)-H(12B)	107.9
C(12)-C(13)-H(13A)	109.5

C(12)-C(13)-H(13B)	109.5
H(13A)-C(13)-H(13B)	109.5
C(12)-C(13)-H(13C)	109.5
H(13A)-C(13)-H(13C)	109.5
H(13B)-C(13)-H(13C)	109.5

Symmetry transformations used to generate equivalent atoms:

Table 4. Anisotropic displacement parameters ($Å^2$x 10^3) for **11**. The anisotropic displacement factor exponent takes the form: $-2p^2$[h^2 $a^{*2}U^{11}$ + ... + 2 h k a* b* U^{12}]

	U^{11}	U^{22}	U^{33}	U^{23}	U^{13}	U^{12}
O(1)	74(1)	71(1)	92(1)	7(1)	8(1)	-10(1)
O(2)	65(1)	58(1)	59(1)	0(1)	-4(1)	-1(1)
O(3)	110(1)	66(1)	57(1)	-3(1)	5(1)	13(1)
C(1)	66(1)	57(1)	63(1)	0(1)	6(1)	4(1)
C(2)	74(1)	63(1)	62(1)	6(1)	3(1)	7(1)
C(3)	63(1)	70(1)	58(1)	0(1)	5(1)	5(1)
C(4)	62(1)	63(1)	53(1)	4(1)	2(1)	-4(1)
C(5)	75(1)	57(1)	61(1)	2(1)	-6(1)	-5(1)
C(6)	81(1)	51(1)	64(1)	-4(1)	-6(1)	-3(1)
C(7)	82(2)	73(1)	69(1)	-10(1)	-1(1)	5(1)
C(8)	93(2)	95(2)	84(2)	-22(2)	-15(2)	26(2)
C(9)	128(3)	80(2)	95(2)	-7(2)	-34(2)	29(2)
C(10)	148(4)	75(2)	102(2)	25(2)	-20(2)	-4(2)
C(11)	107(2)	72(2)	95(2)	22(1)	-15(2)	-15(2)
C(12)	68(1)	95(2)	81(2)	3(1)	-10(1)	-1(1)
C(13)	76(2)	140(3)	100(2)	-8(2)	-18(2)	27(2)

Table 5. Hydrogen coordinates (x 10^4) and isotropic displacement parameters ($Å^2$x 10^3) for **11**.

	x	y	z	U(eq)
H(3)	5753	4192	7712	116
H(2A)	7717	417	6910	79
H(2B)	7023	1476	7559	79
H(3A)	8947	1640	5845	76
H(4)	7548	3185	5242	71
H(5)	7568	4510	6581	77
H(7)	3184	4050	6411	90
H(8)	1315	5215	5538	108
H(9)	2296	6658	4486	121
H(10)	5163	6916	4282	130
H(11)	7064	5766	5160	109
H(12A)	9205	3123	7500	97
H(12B)	10337	3265	6572	97
H(13A)	11499	1351	6862	158
H(13B)	10400	1243	7805	158
H(13C)	11839	2205	7745	158

Table 6. Torsion angles [°] for **11**.

C(4)-O(2)-C(1)-O(1)	176.6(2)
C(4)-O(2)-C(1)-C(2)	-4.8(2)
O(1)-C(1)-C(2)-C(3)	163.8(2)
O(2)-C(1)-C(2)-C(3)	-14.7(2)
C(1)-C(2)-C(3)-C(12)	153.9(2)
C(1)-C(2)-C(3)-C(4)	26.6(2)
C(1)-O(2)-C(4)-C(5)	-102.6(2)
C(1)-O(2)-C(4)-C(3)	22.1(2)
C(12)-C(3)-C(4)-O(2)	-156.8(2)
C(2)-C(3)-C(4)-O(2)	-29.6(2)
C(12)-C(3)-C(4)-C(5)	-36.9(3)
C(2)-C(3)-C(4)-C(5)	90.3(2)
O(2)-C(4)-C(5)-O(3)	60.6(2)
C(3)-C(4)-C(5)-O(3)	-56.5(3)
O(2)-C(4)-C(5)-C(6)	-63.5(2)
C(3)-C(4)-C(5)-C(6)	179.37(19)
O(3)-C(5)-C(6)-C(7)	-28.9(3)
C(4)-C(5)-C(6)-C(7)	92.2(3)
O(3)-C(5)-C(6)-C(11)	152.8(2)
C(4)-C(5)-C(6)-C(11)	-86.1(3)
C(11)-C(6)-C(7)-C(8)	-1.0(4)
C(5)-C(6)-C(7)-C(8)	-179.3(2)
C(6)-C(7)-C(8)-C(9)	0.3(4)
C(7)-C(8)-C(9)-C(10)	0.7(5)
C(8)-C(9)-C(10)-C(11)	-0.9(6)
C(9)-C(10)-C(11)-C(6)	0.2(6)
C(7)-C(6)-C(11)-C(10)	0.8(5)
C(5)-C(6)-C(11)-C(10)	179.0(3)
C(2)-C(3)-C(12)-C(13)	62.7(3)
C(4)-C(3)-C(12)-C(13)	-177.3(3)

Symmetry transformations used to generate equivalent atoms:

Table 7. Hydrogen bonds for **11** [Å and °].

D-H...A	d(D-H)	d(H...A)	d(D...A)	<(DHA)
O(3)-H(3)...O(1)#1	0.82	2.02	2.833(3)	175.2
O(3)-H(3)...O(1)#1	0.82	2.02	2.833(3)	175.2

Symmetry transformations used to generate equivalent atoms:
#1 -x+1,y+1/2,-z+3/2

10. Copies of ^{1}H NMR, ^{13}C NMR and ^{19}F NMR spectra

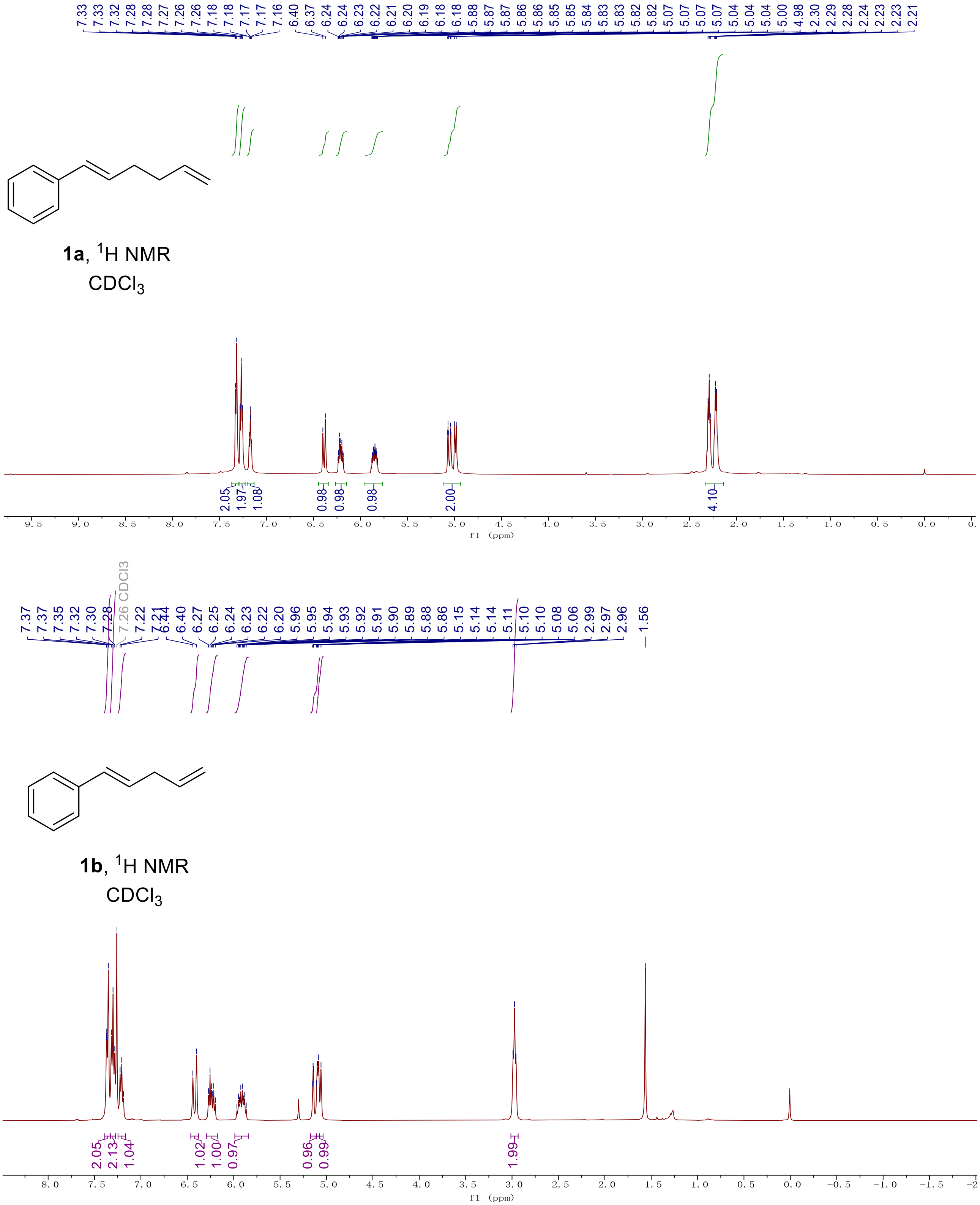

Supplementary Fig. 60. ^{1}H NMR spectra of compound **1a** and **1b**.

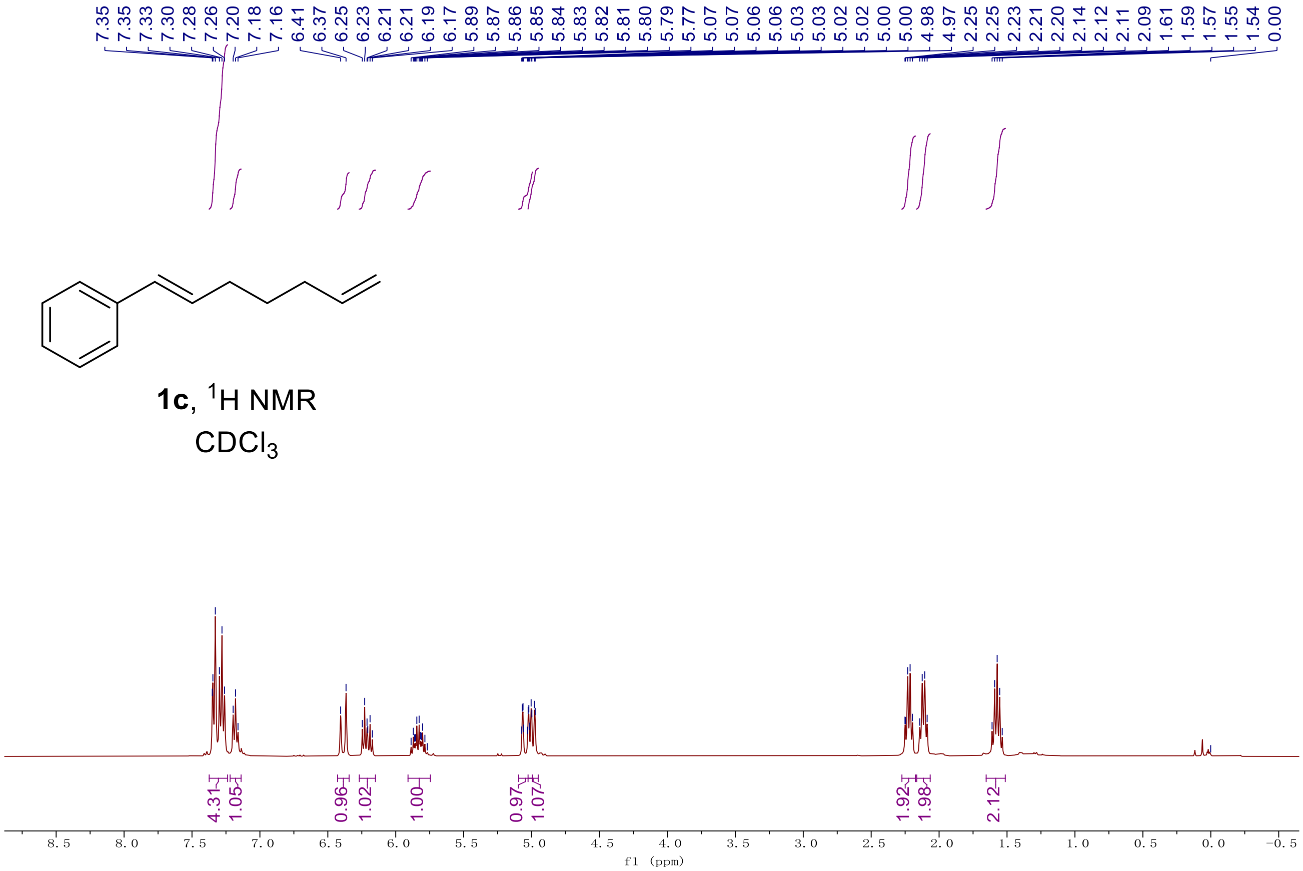

Supplementary Fig. 61. ^{1}H NMR spectra of compound **1c**.

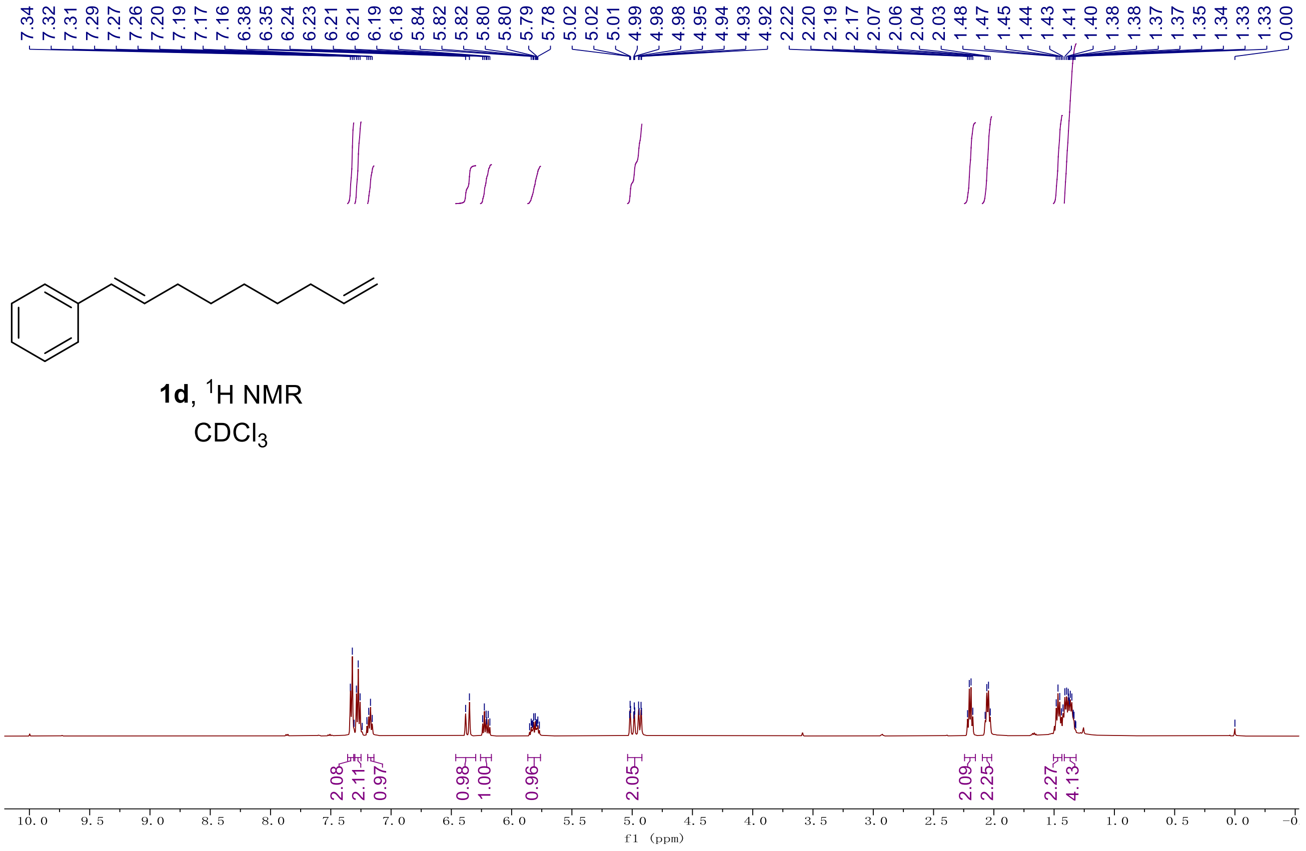

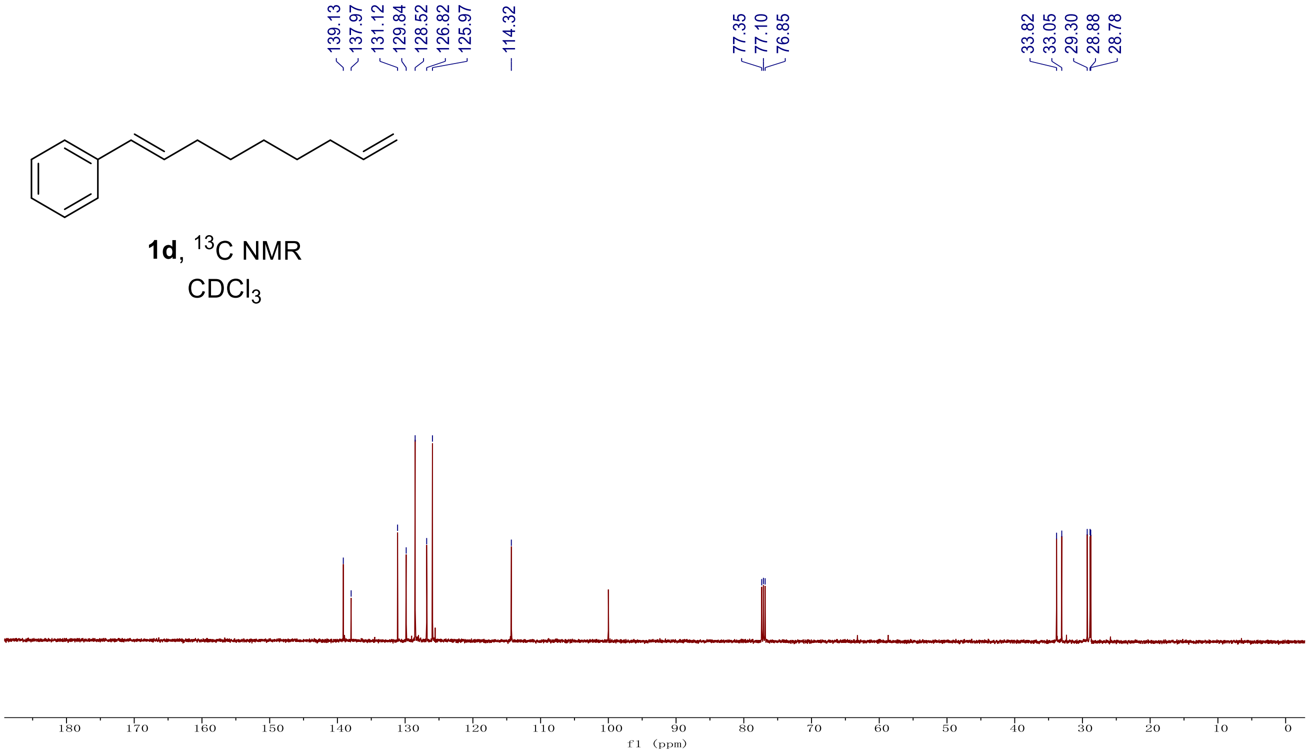

Supplementary Fig. 62. ^{1}H NMR and ^{13}C NMR spectra of compound **1d**.

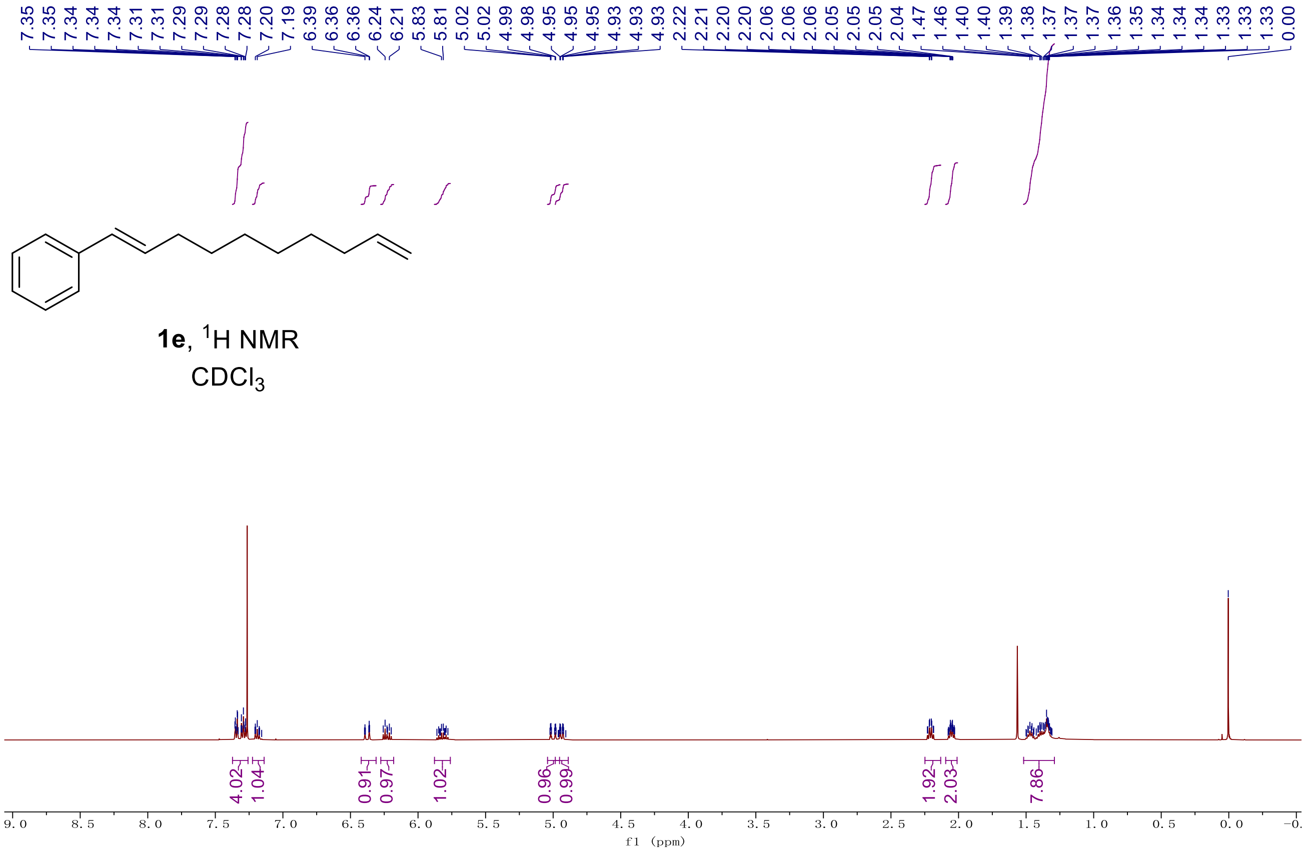

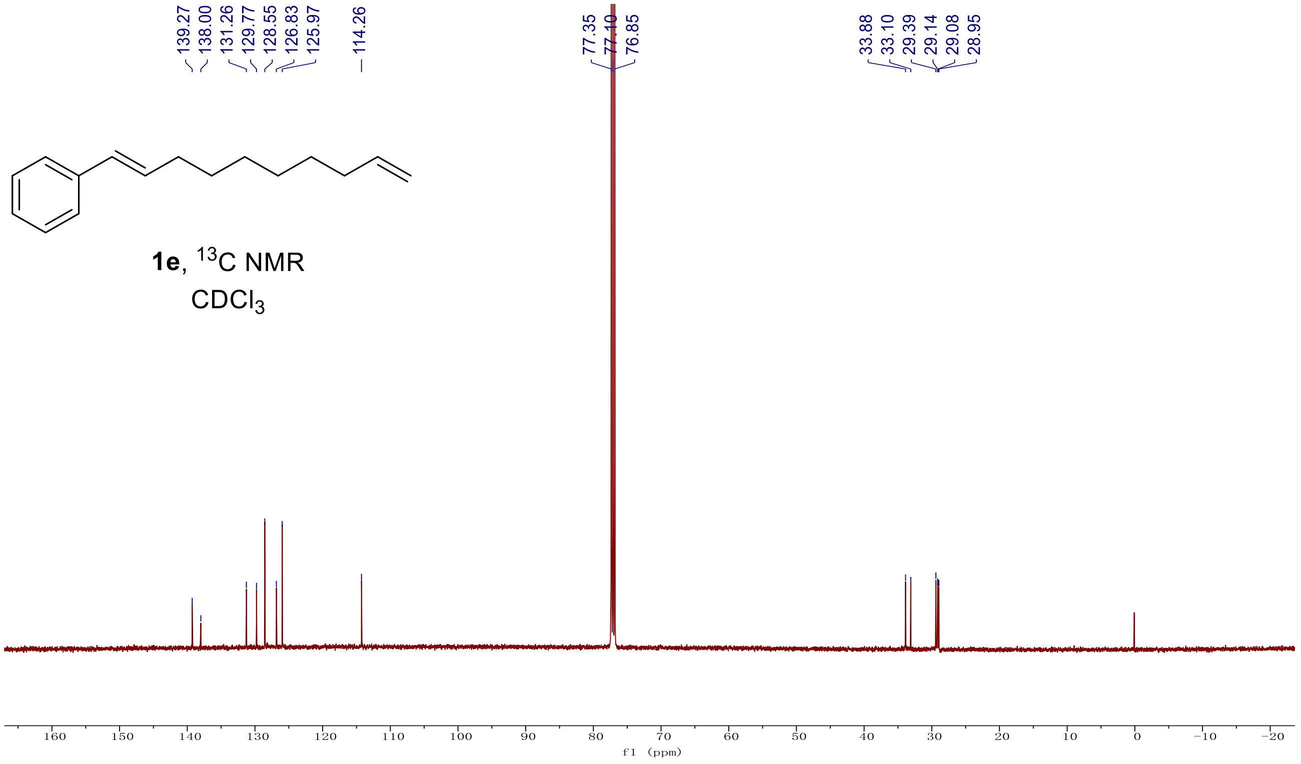

Supplementary Fig. 63. ^{1}H NMR and ^{13}C NMR spectra of compound **1e**.

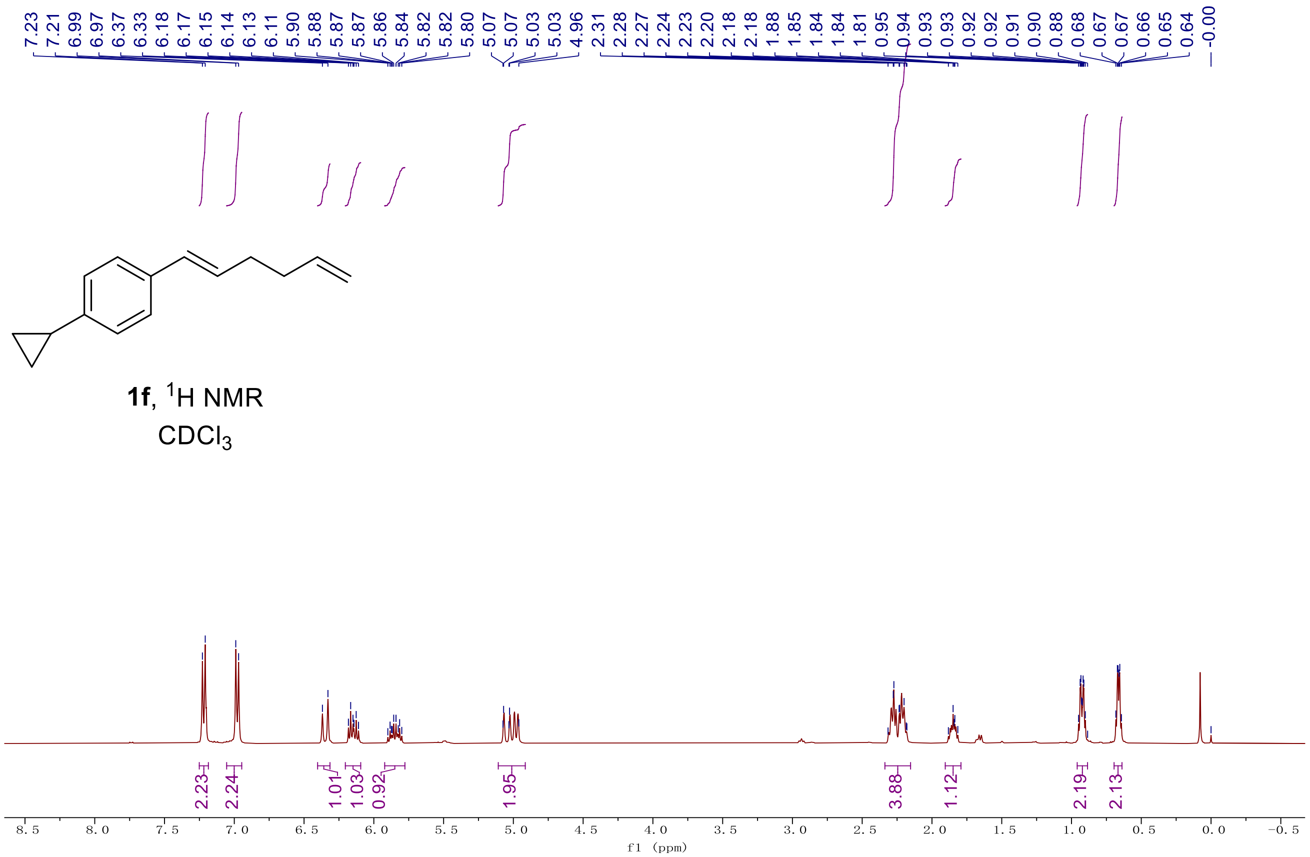

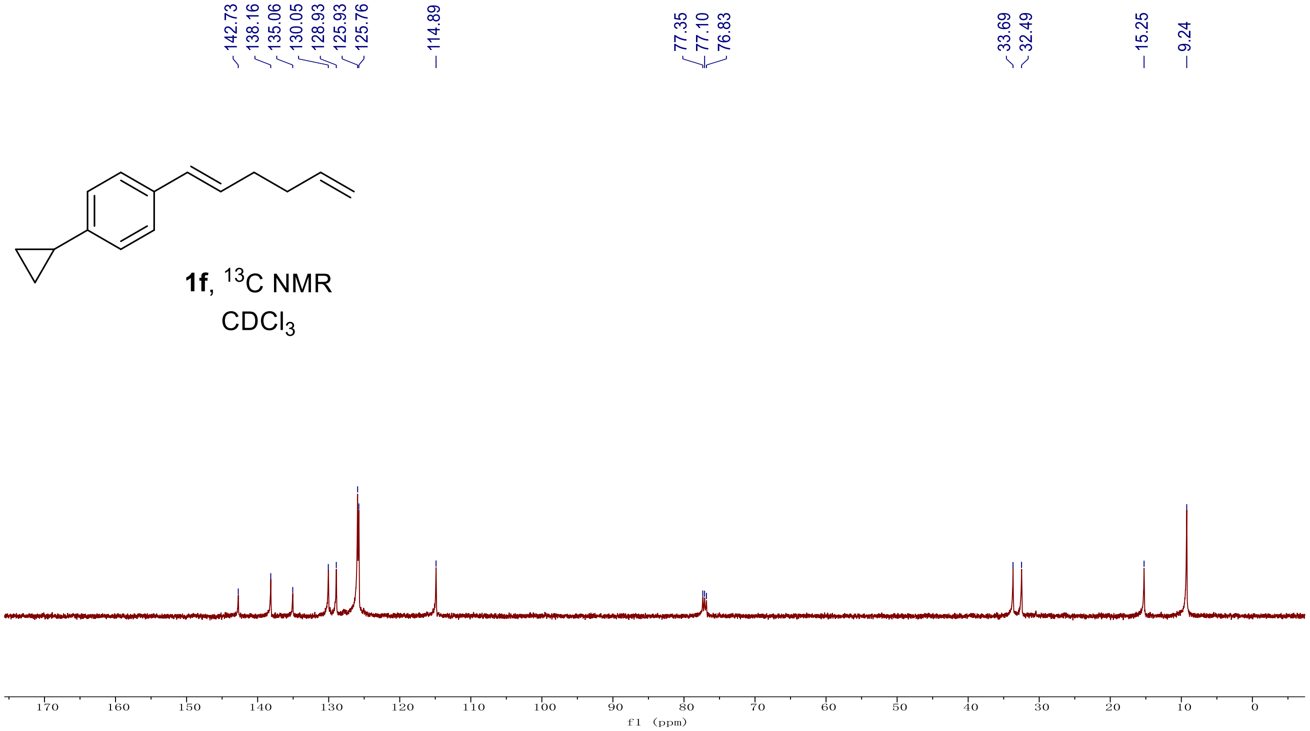

Supplementary Fig. 64. ^{1}H NMR and ^{13}C NMR spectra of compound **1f**.

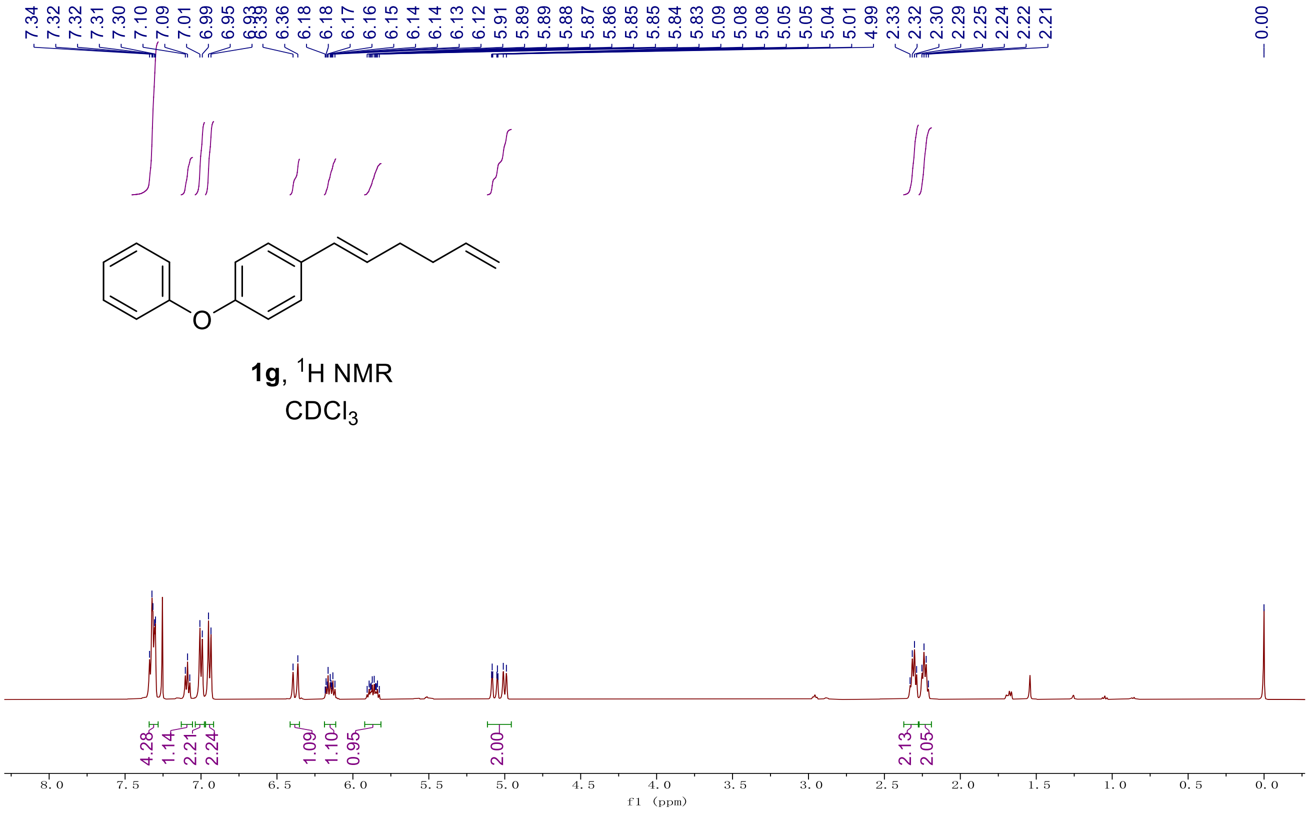

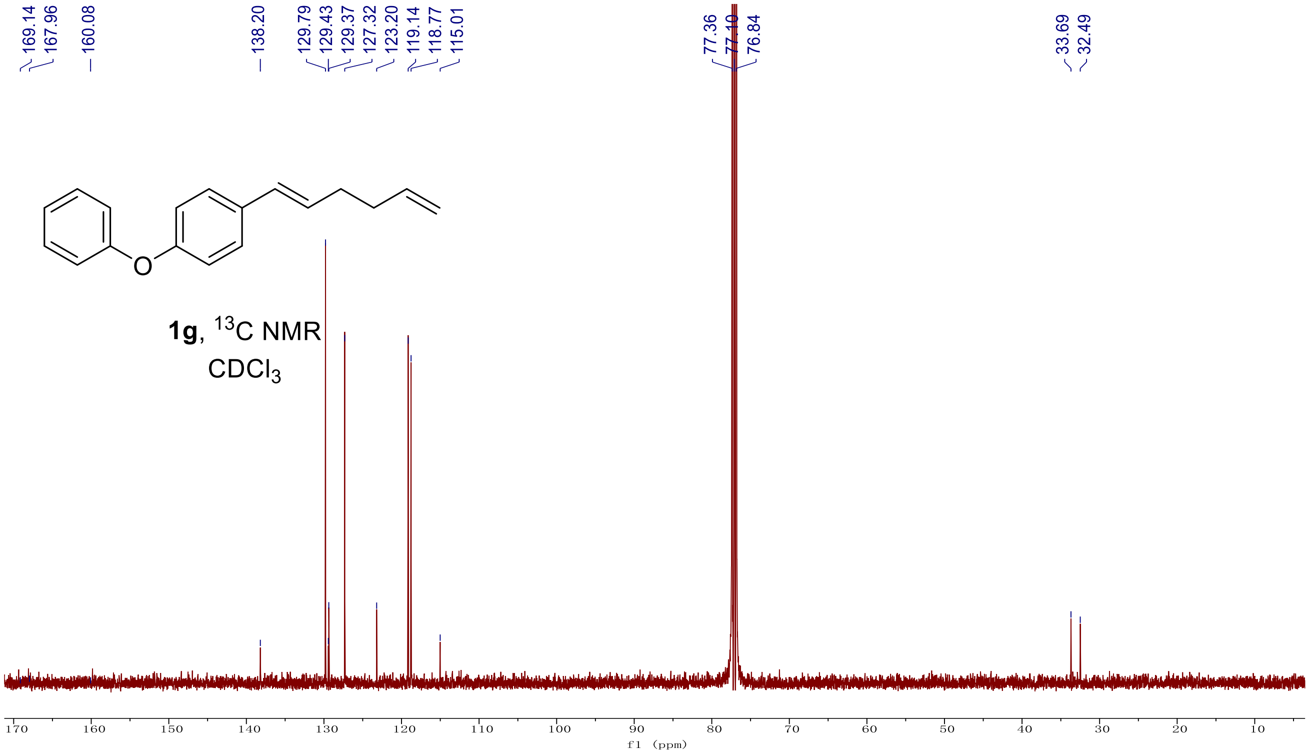

Supplementary Fig. 65. ^{1}H NMR and ^{13}C NMR spectra of compound **1g**.

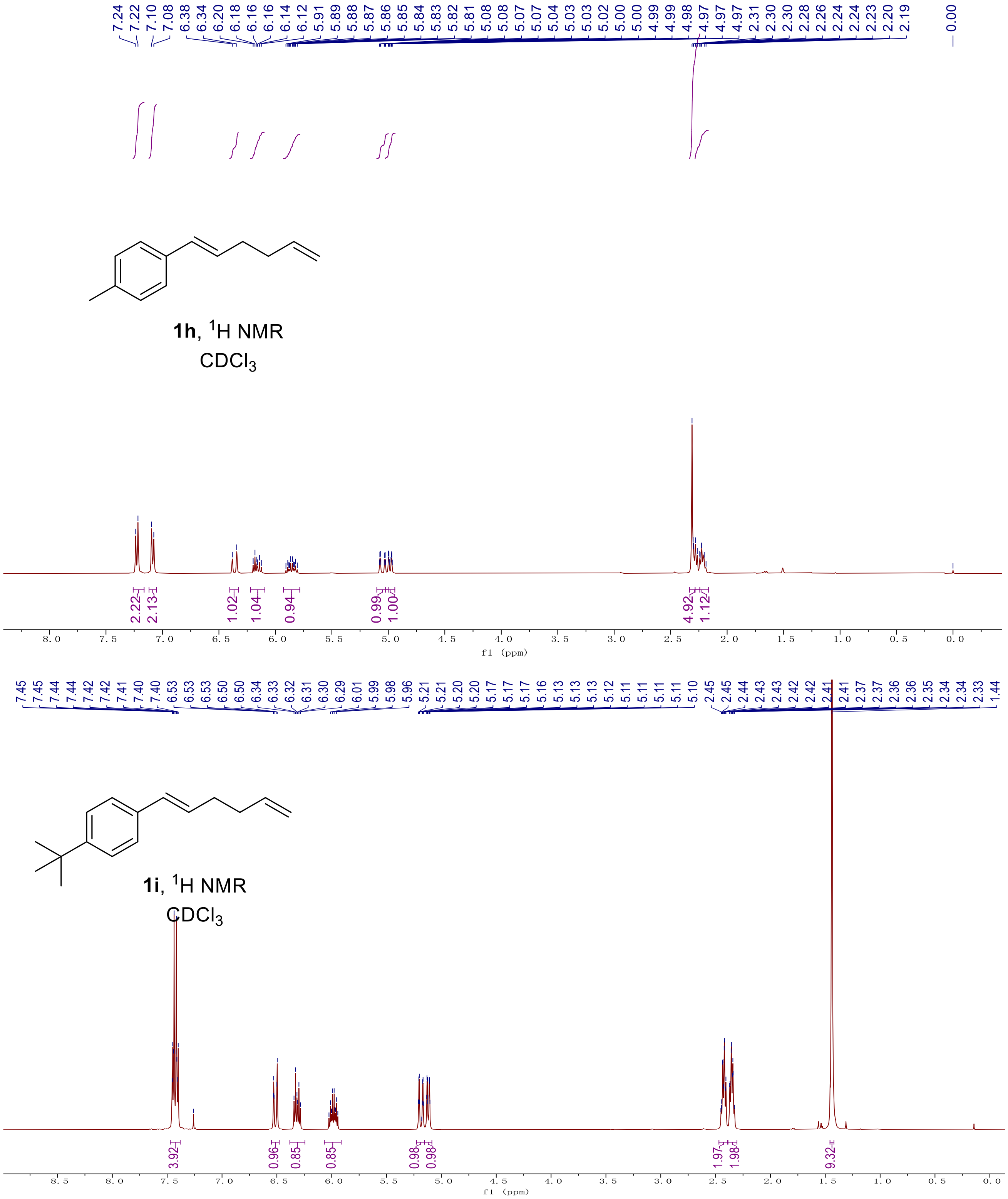

Supplementary Fig. 66. ^{1}H NMR spectra of compound **1h** and **1i**.

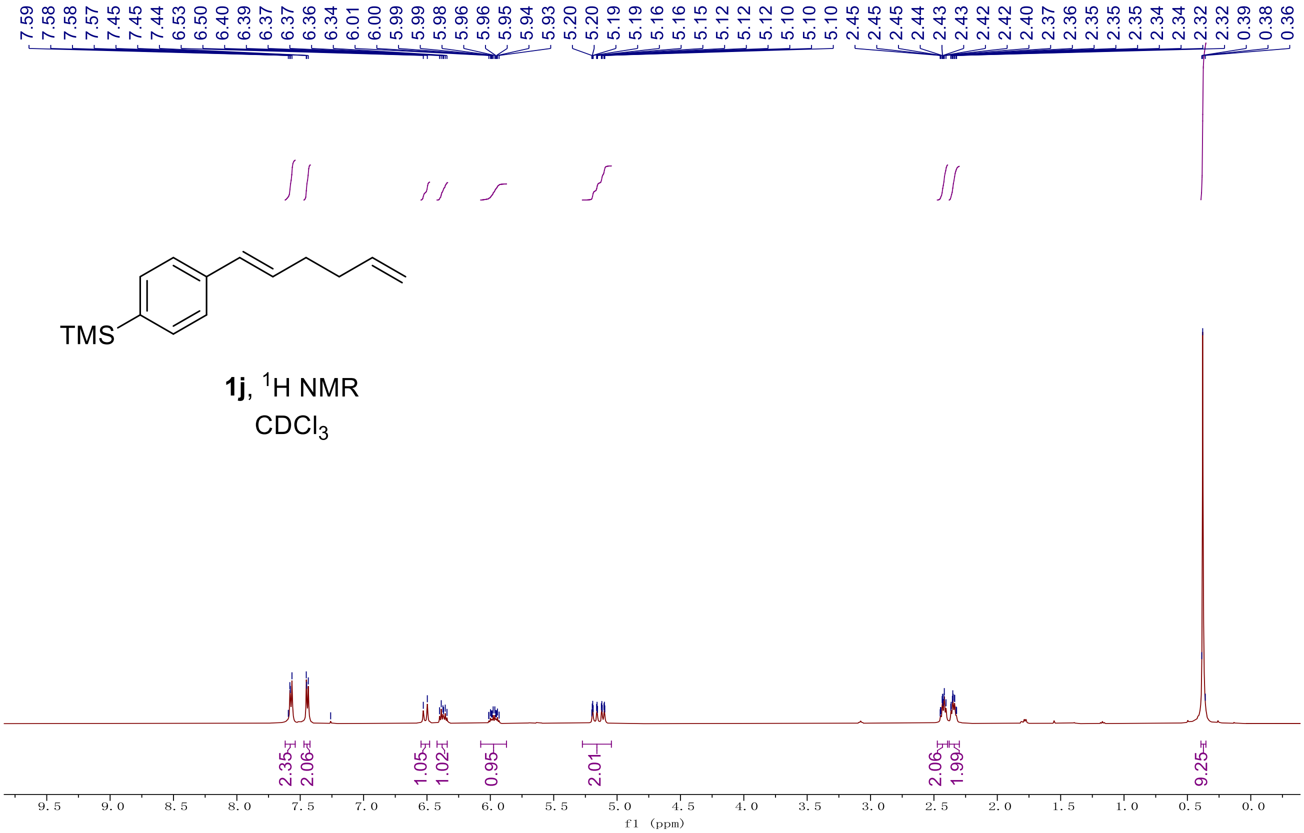

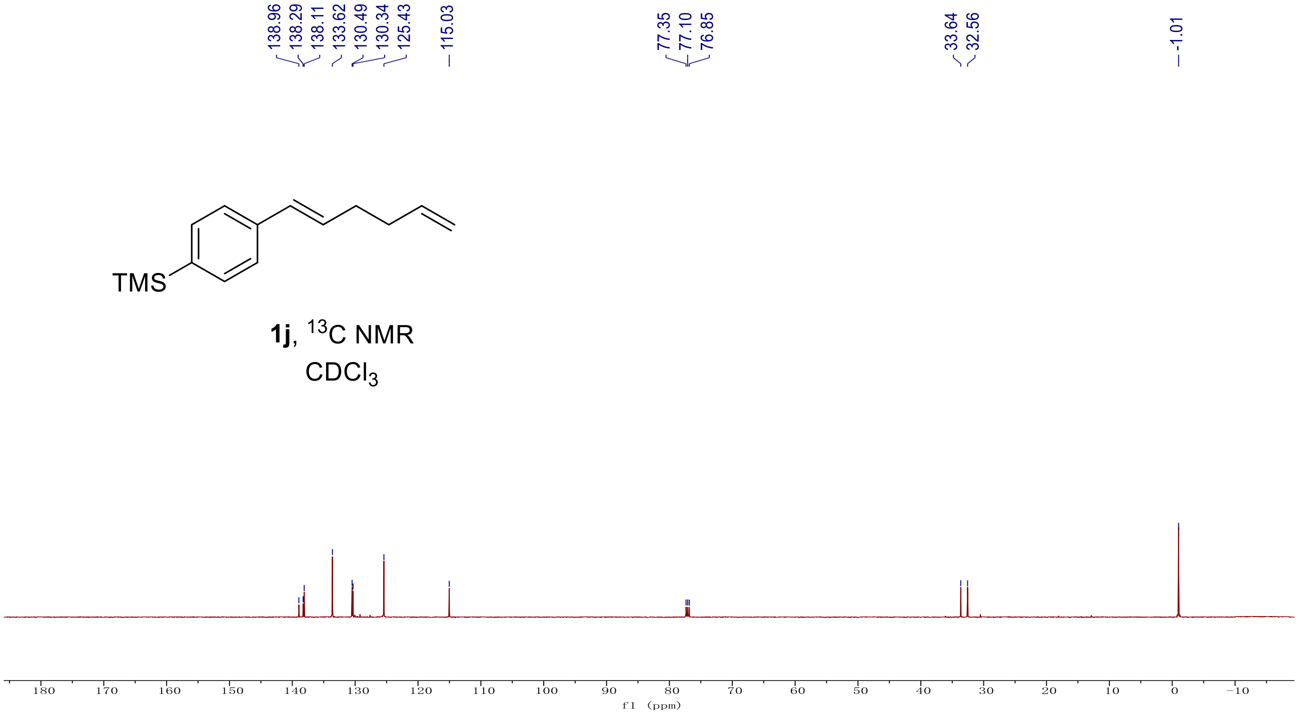

Supplementary Fig. 67. ^{1}H NMR and ^{13}C NMR spectra of compound **1j**.

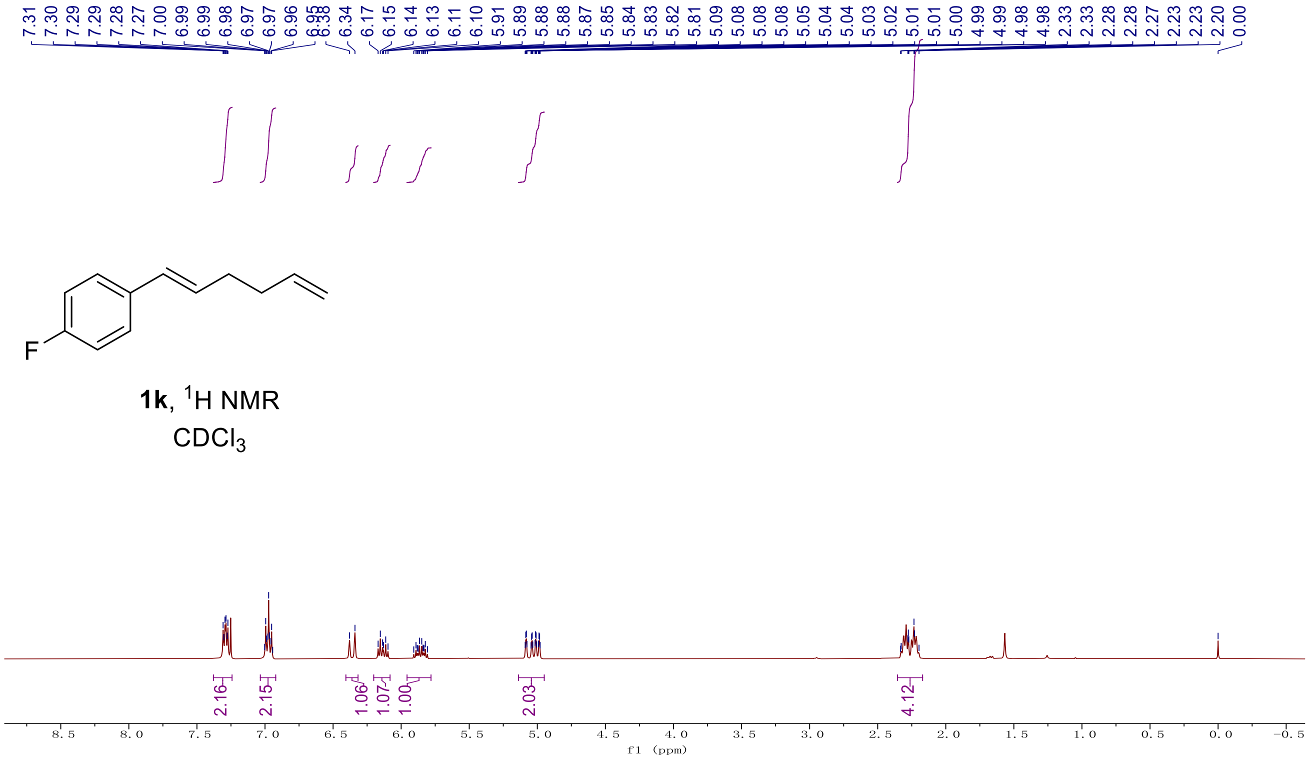

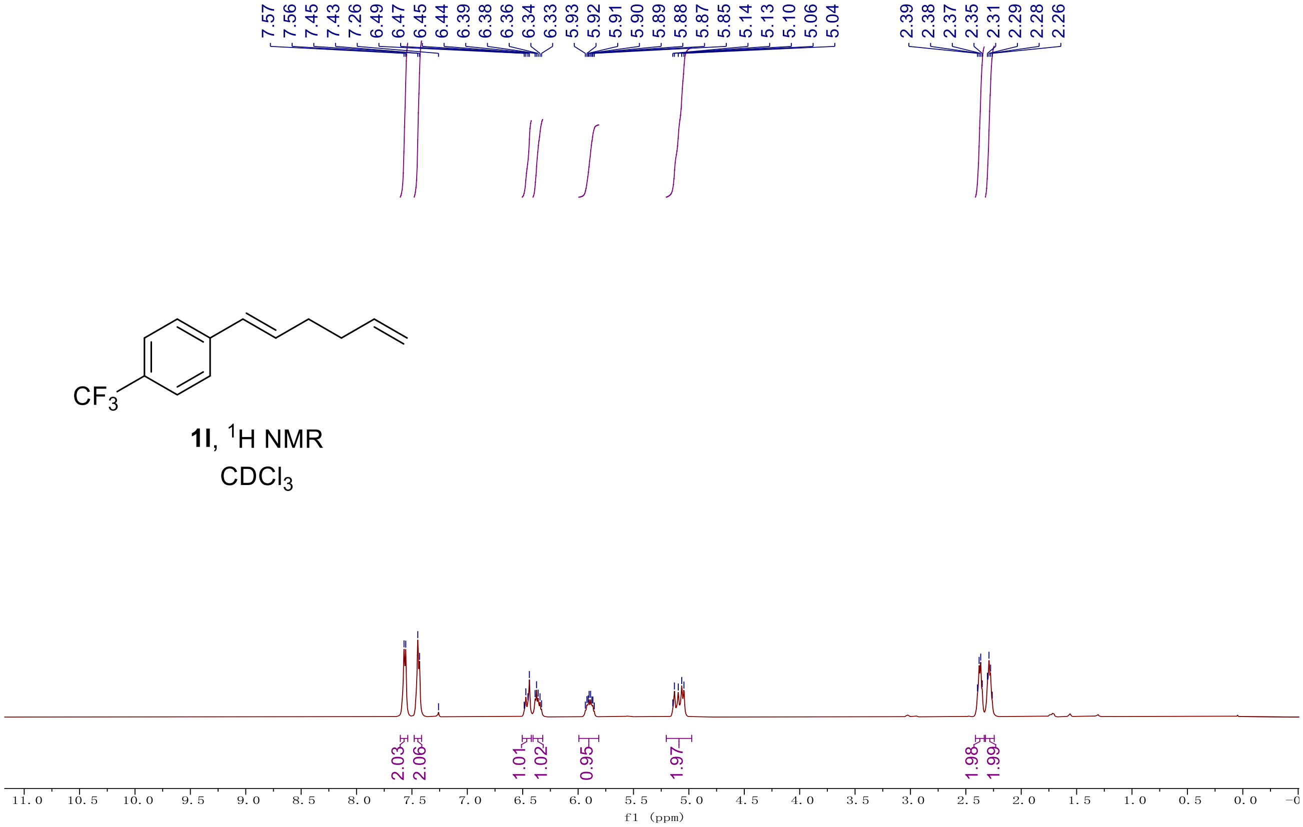

Supplementary Fig. 68. ^{1}H NMR spectra of compound **1k** and **1l**.

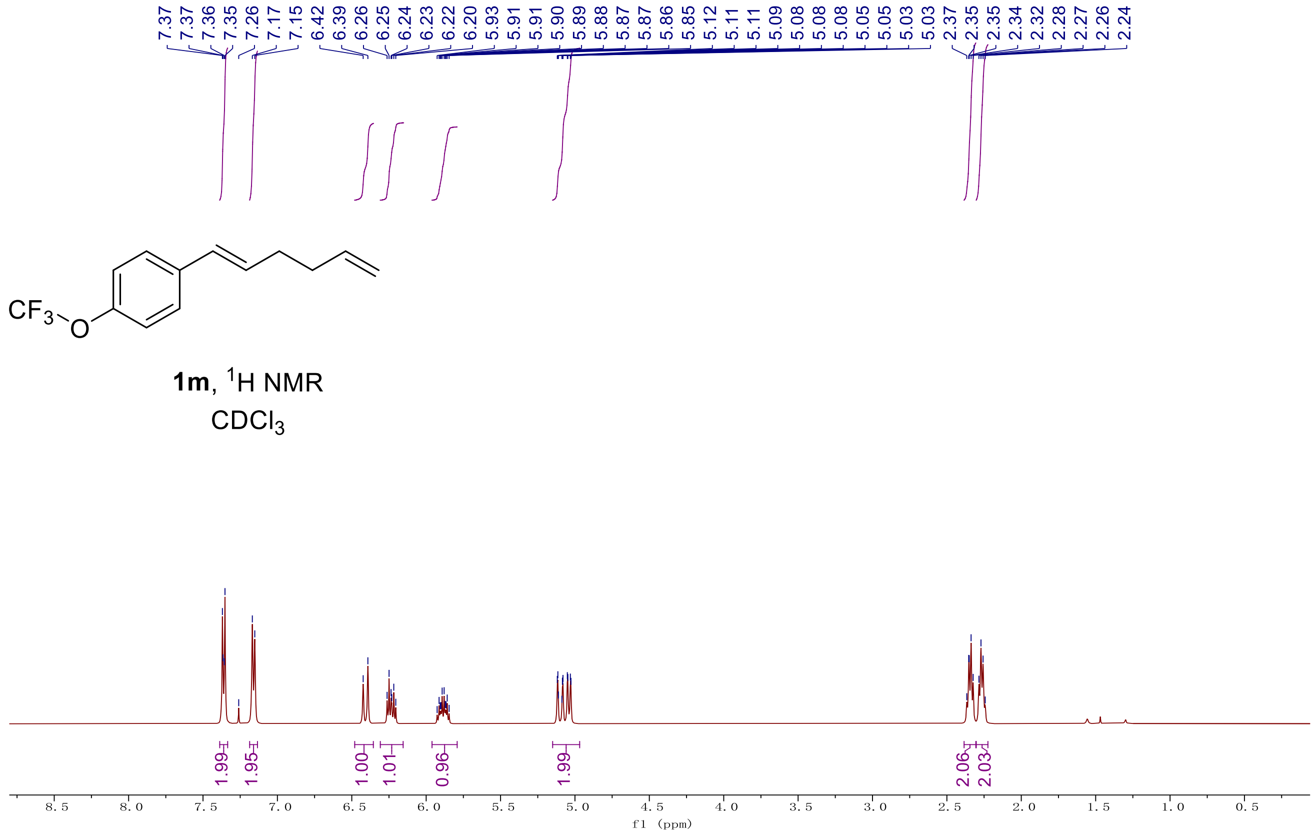

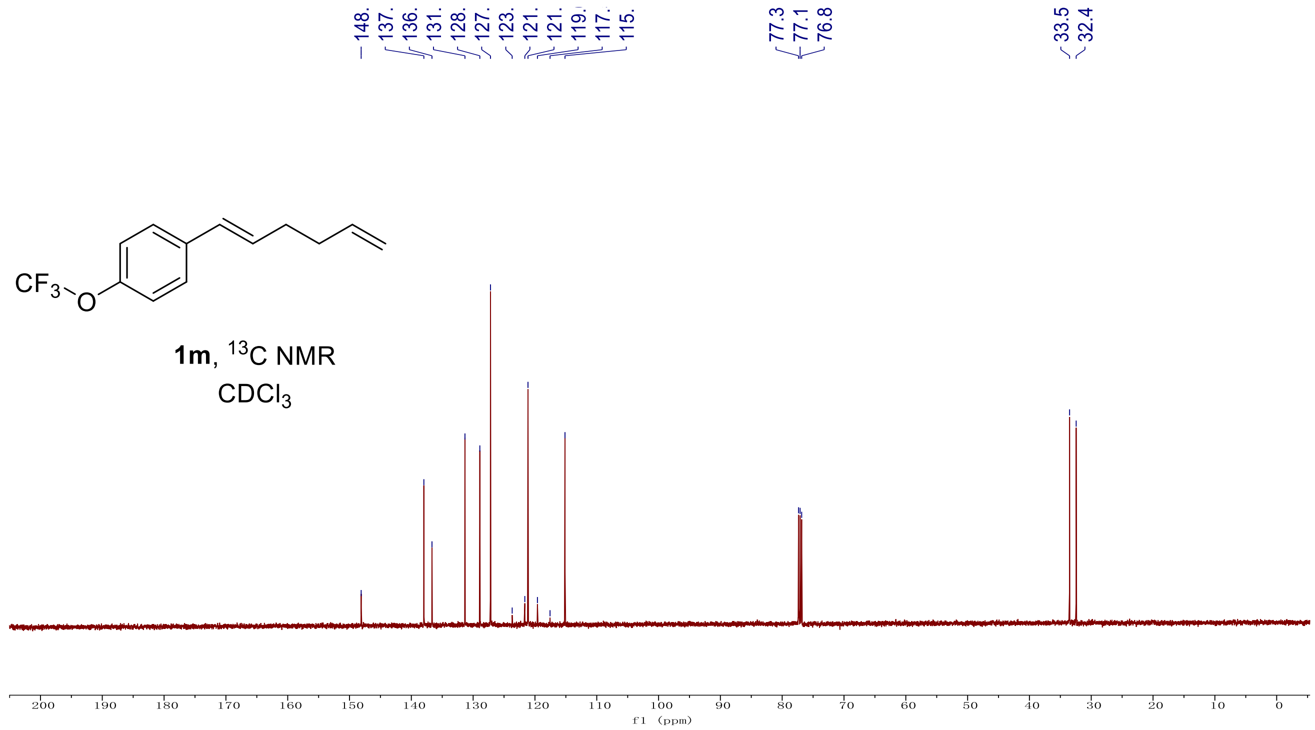

Supplementary Fig. 69. ^{1}H NMR and ^{13}C NMR spectra of compound **1m**.

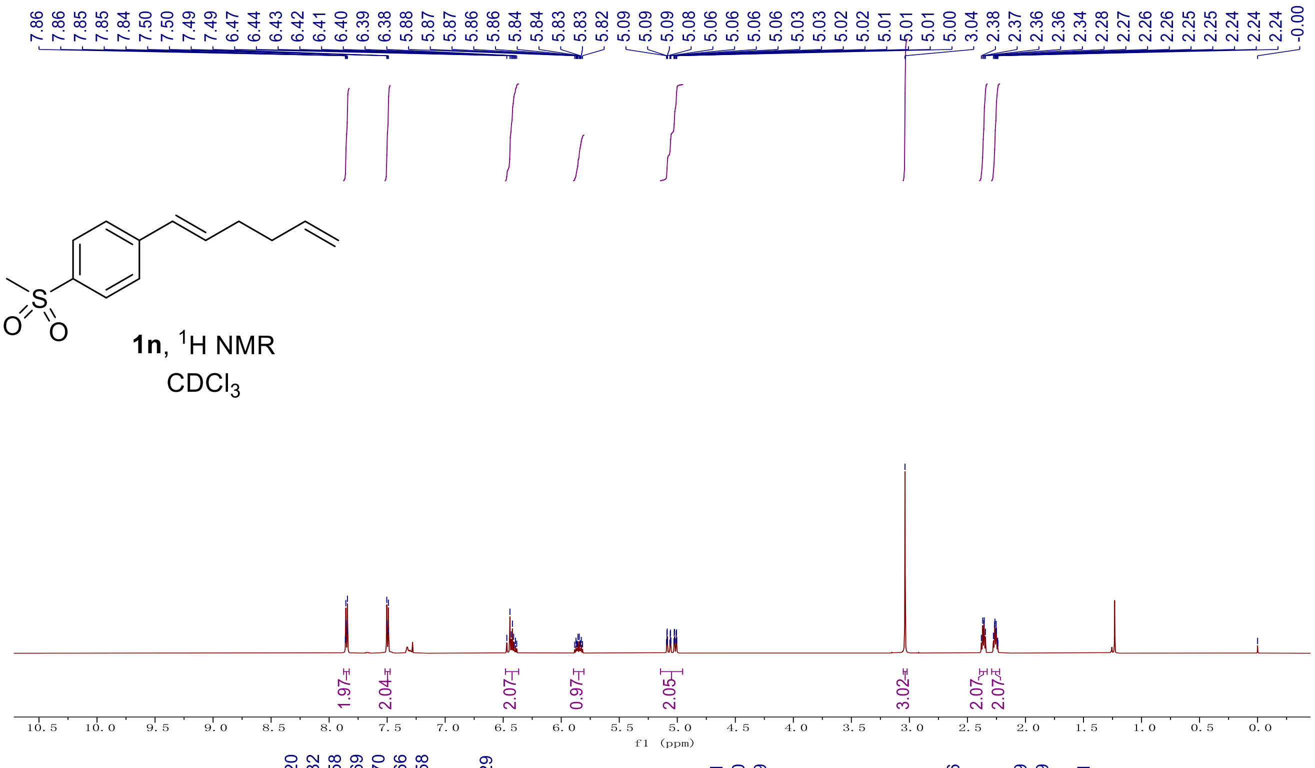

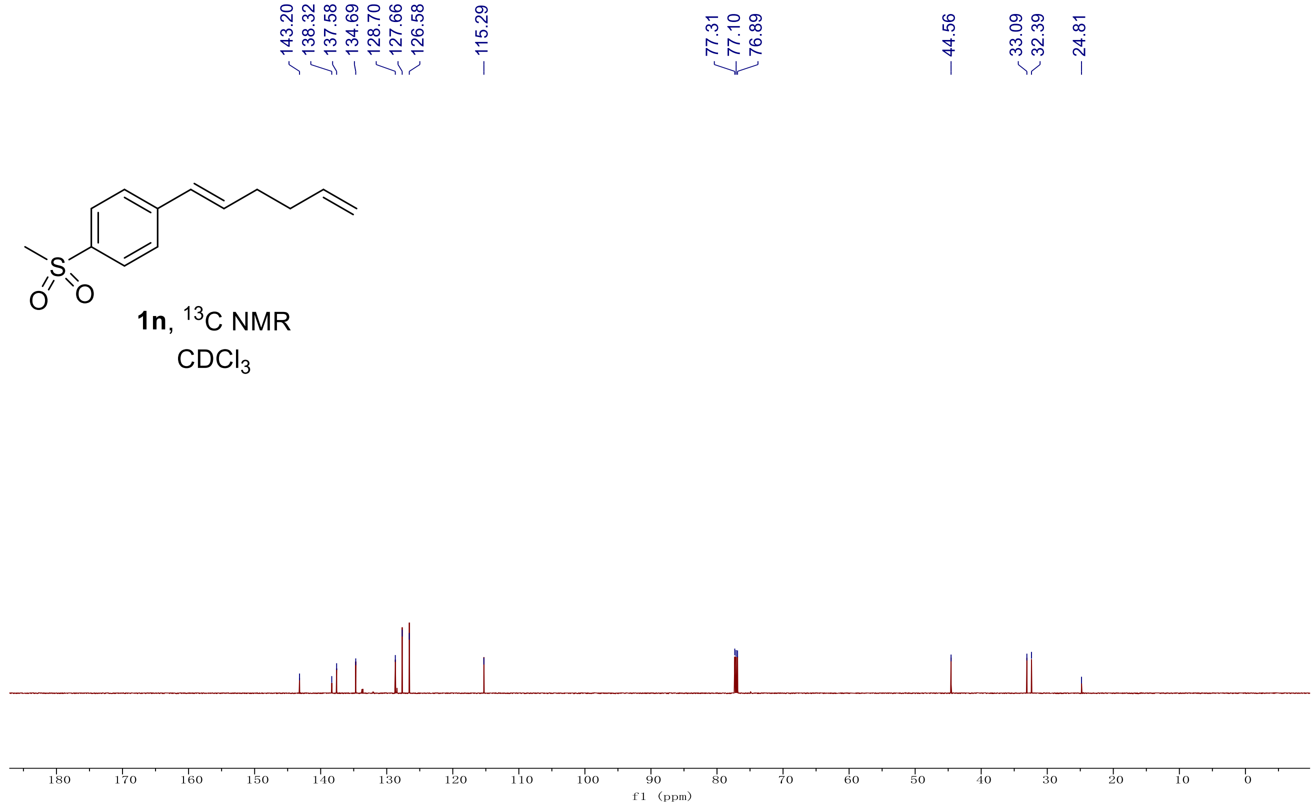

Supplementary Fig. 70. ^{1}H NMR and ^{13}C NMR spectra of compound **1n**.

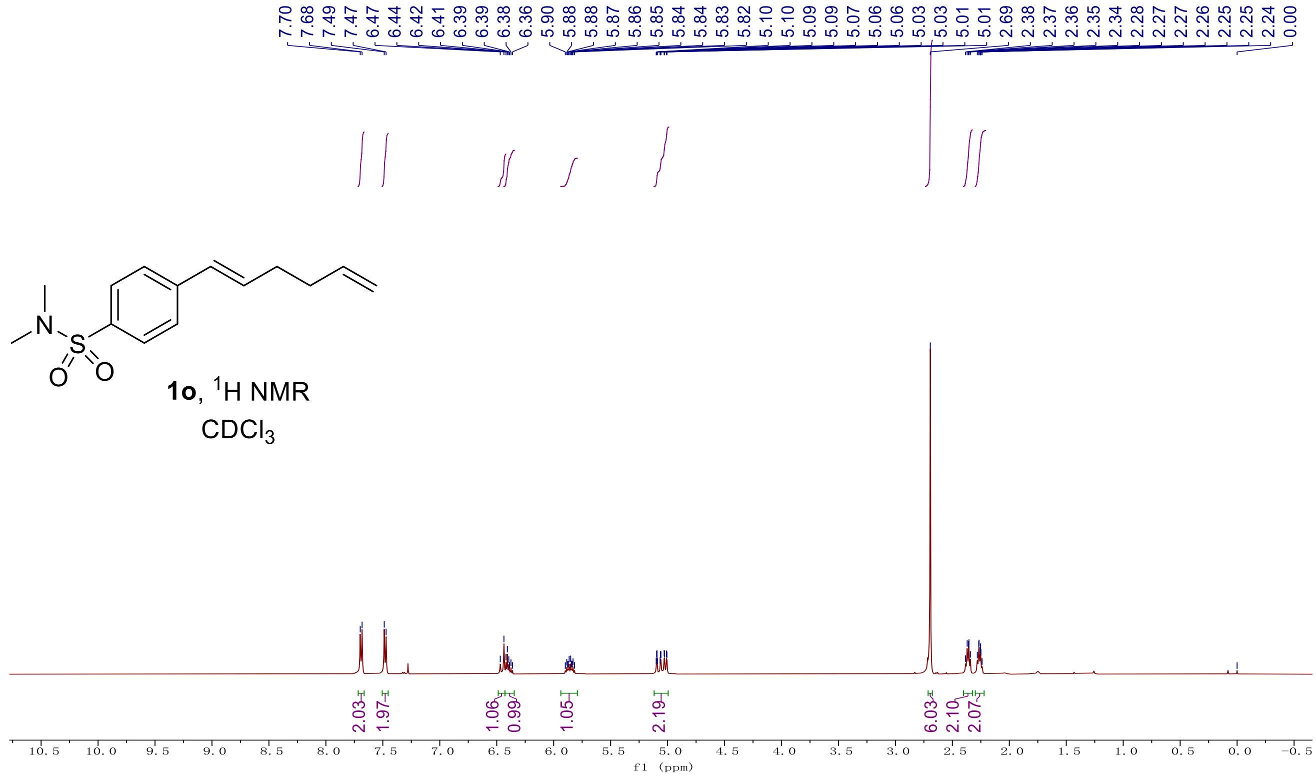

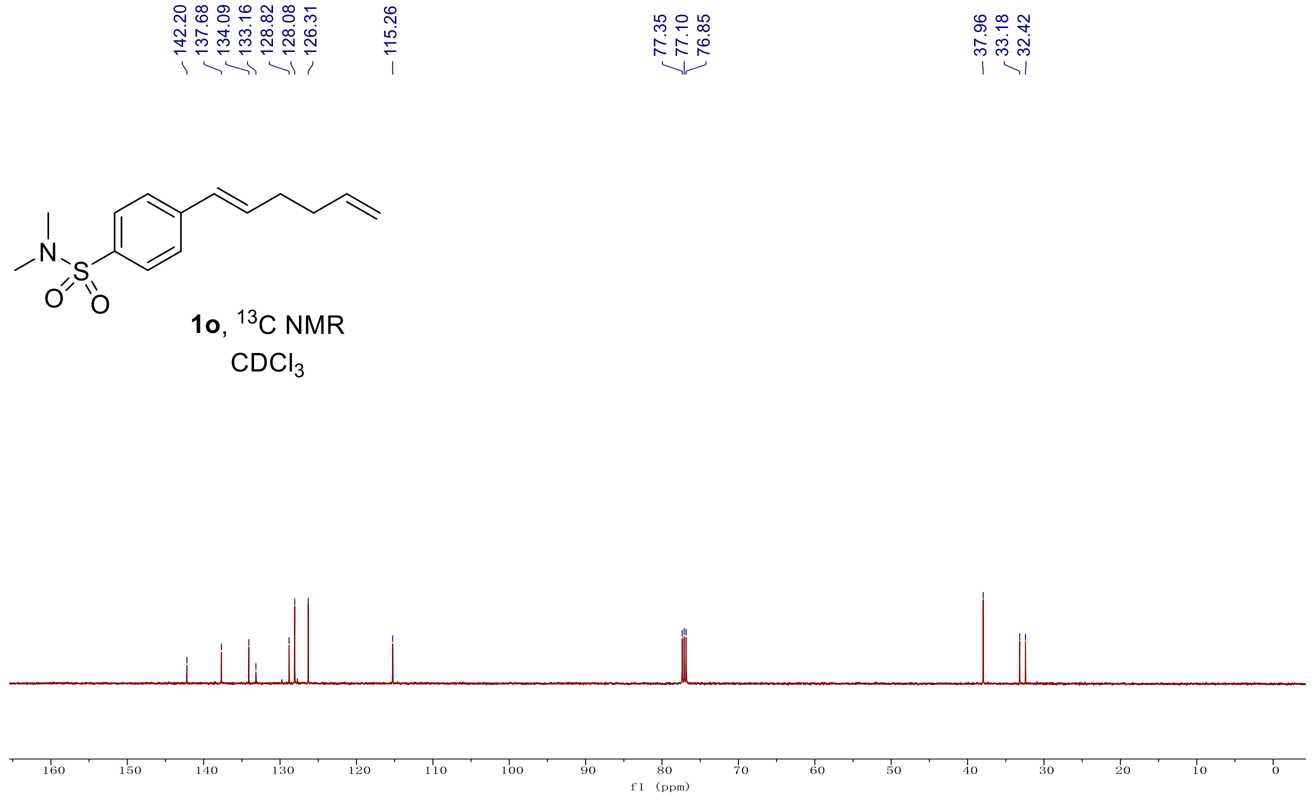

Supplementary Fig. 71. ^{1}H NMR and ^{13}C NMR spectra of compound **1o**.

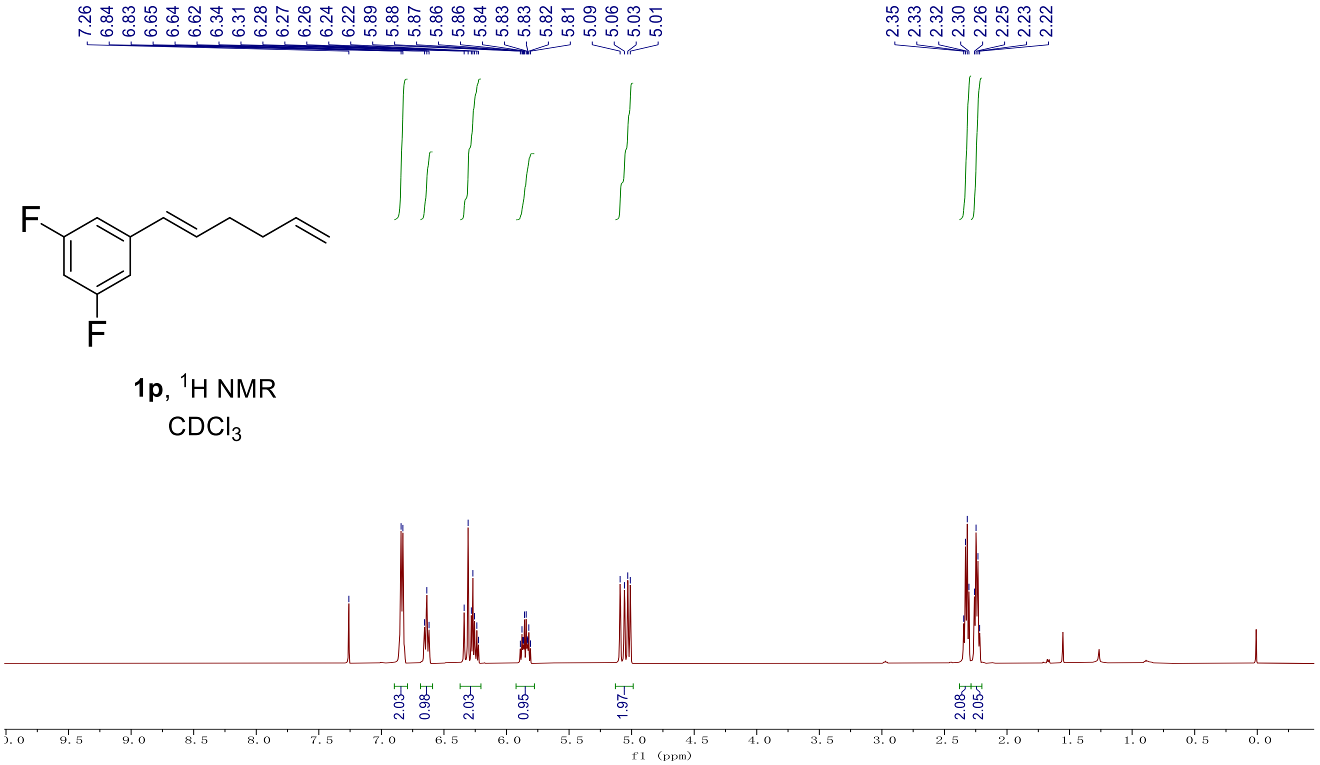

1p, ^{1}H NMR

$CDCl_3$

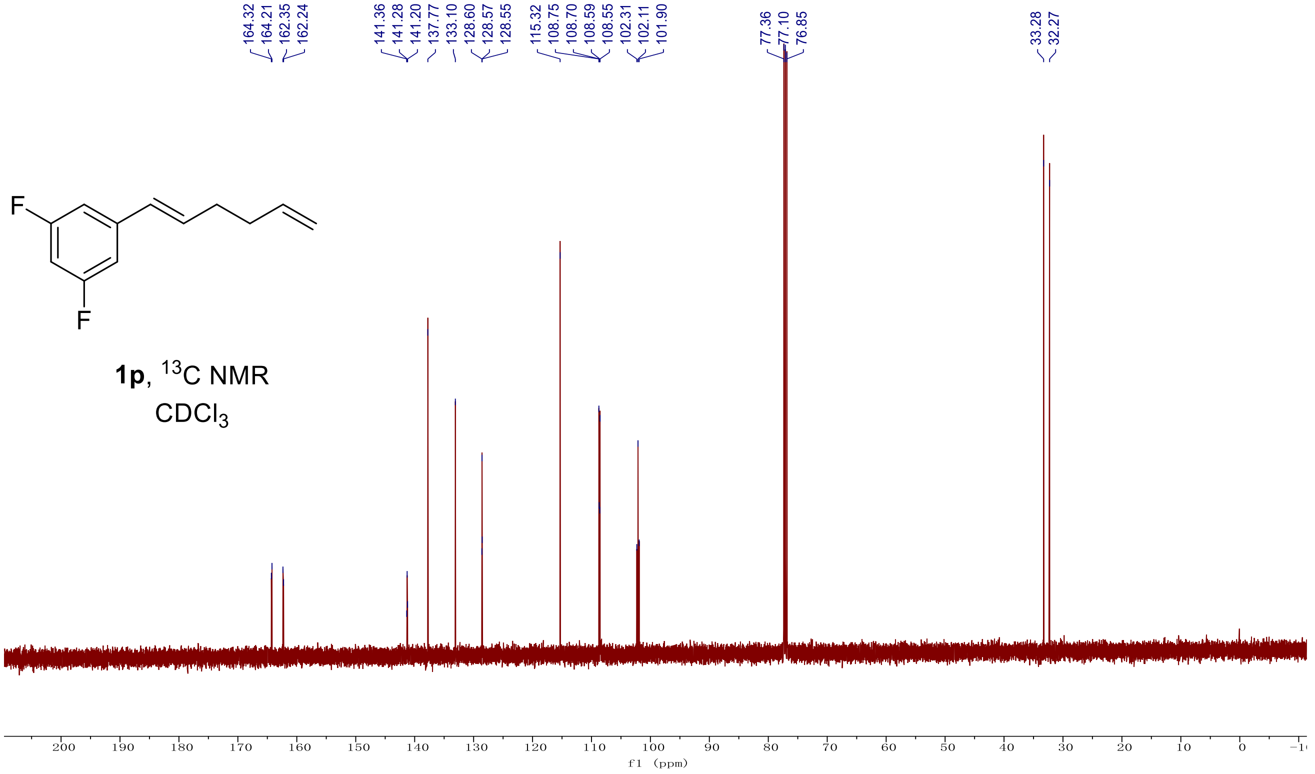

1p, ^{13}C NMR

$CDCl_3$

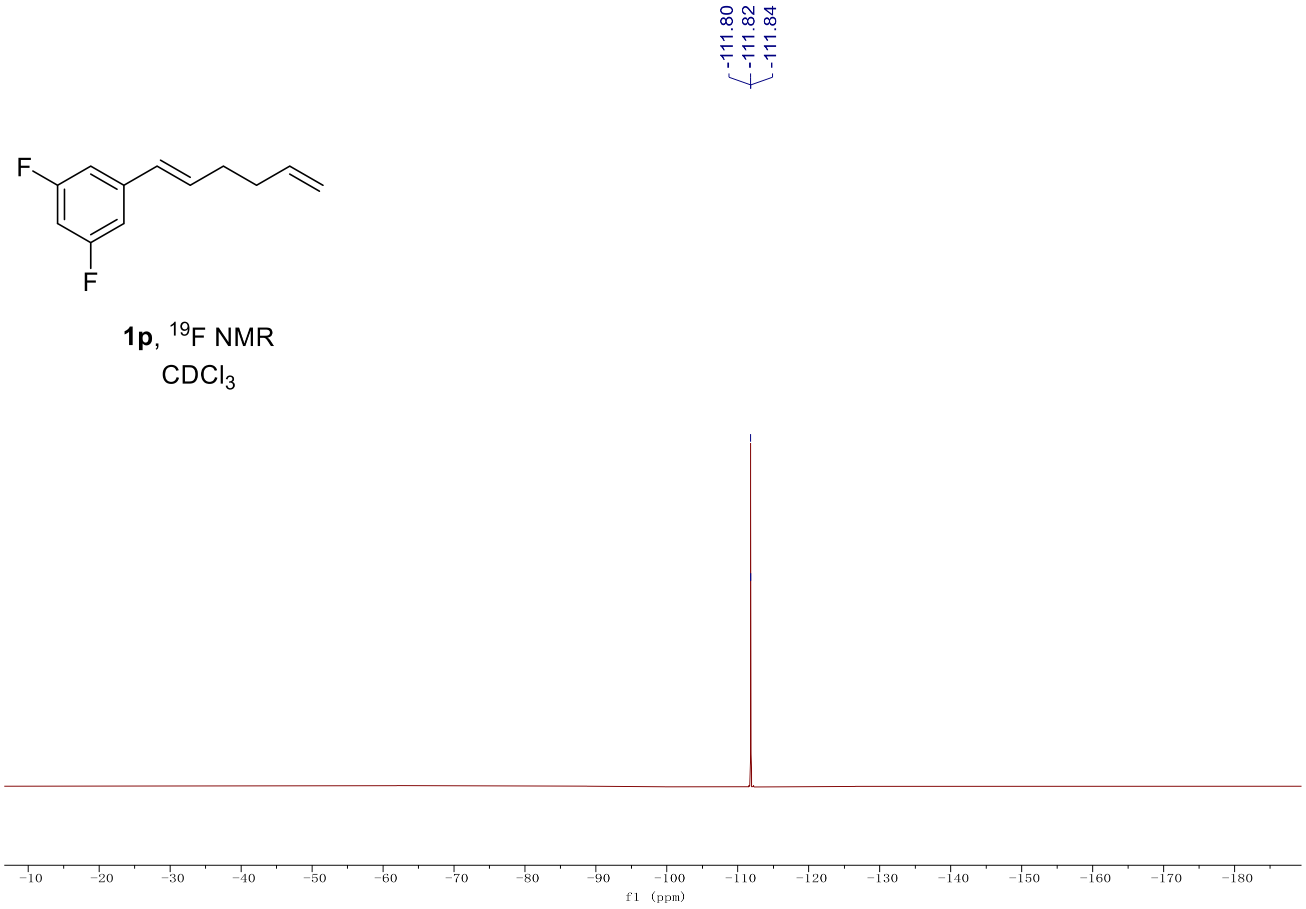

Supplementary Fig. 72. ^{1}H NMR, ^{13}C NMR and ^{19}F NMR spectra of compound **1p**.

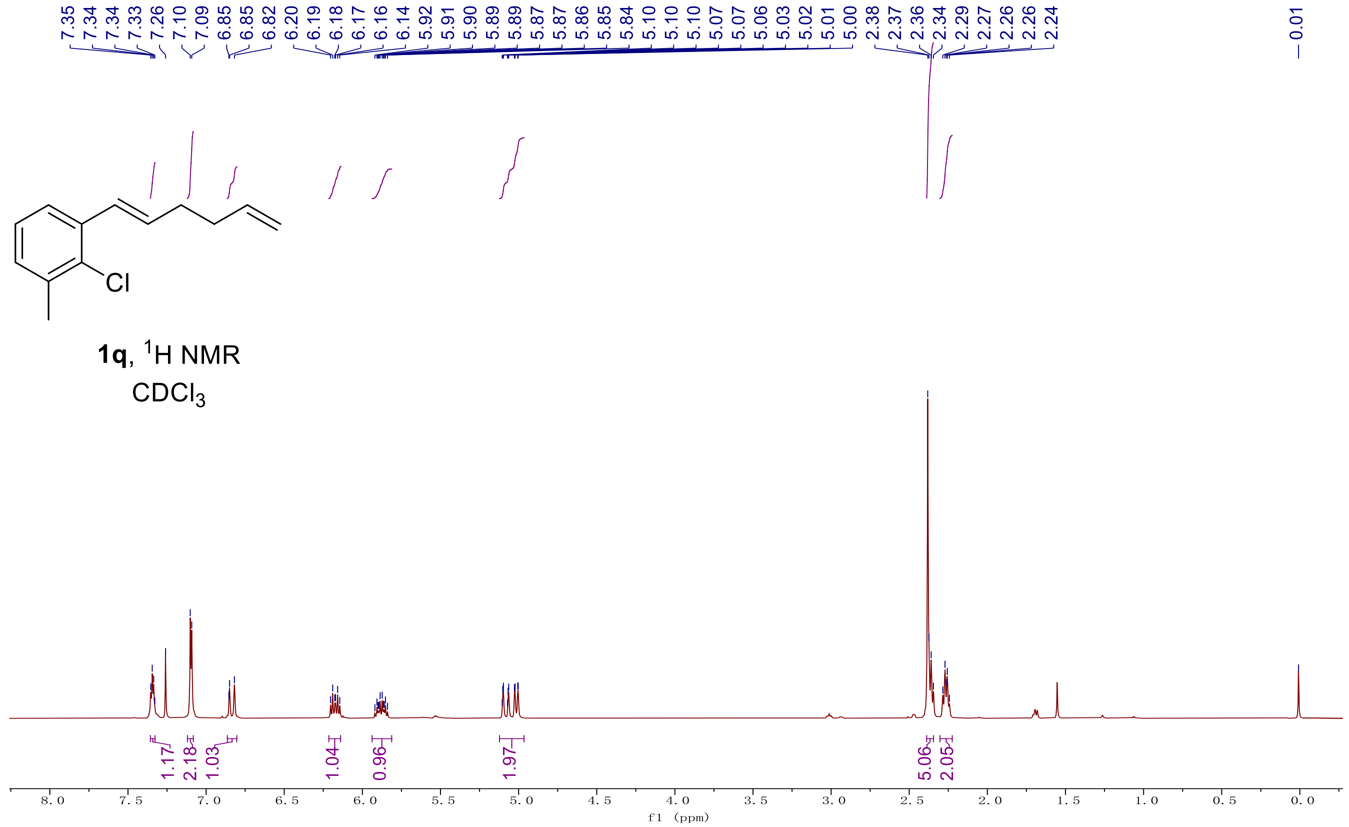

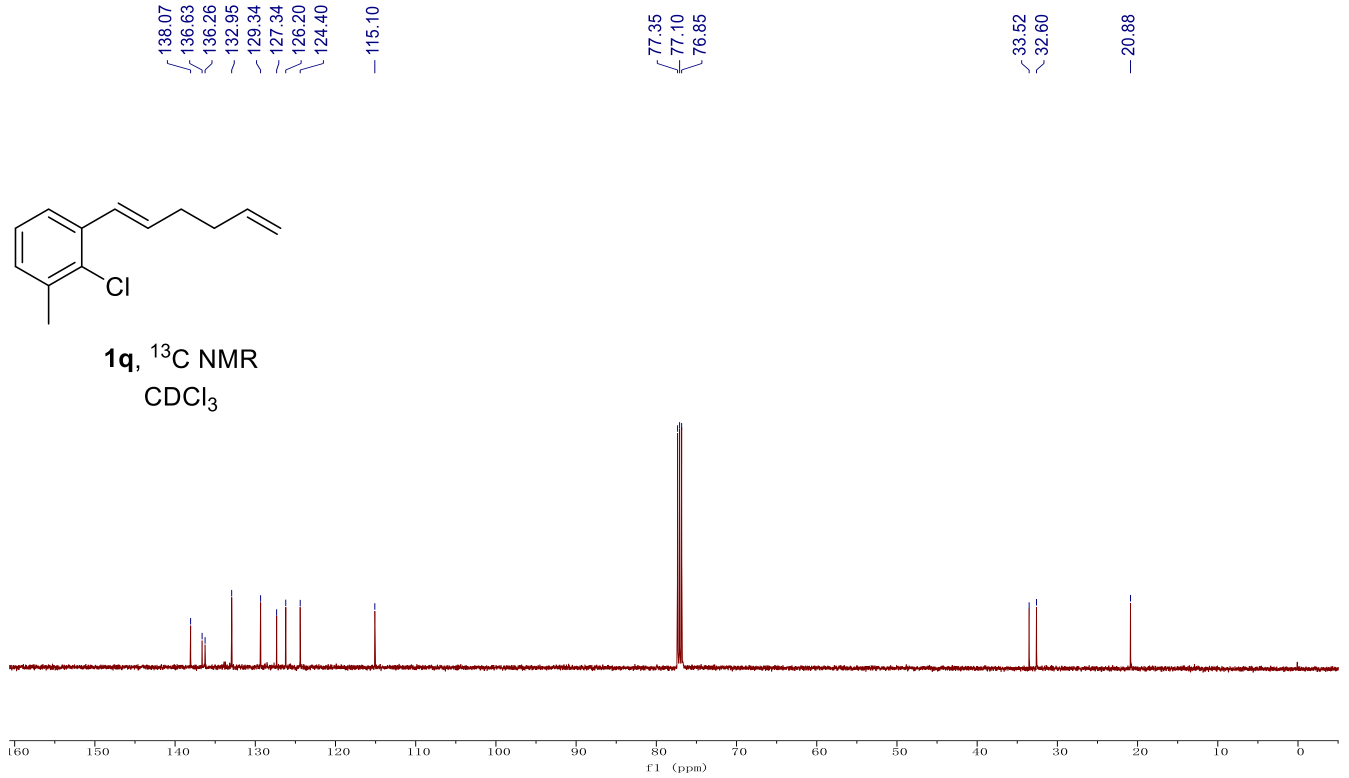

Supplementary Fig. 73. ^{1}H NMR and ^{13}C NMR spectra of compound **1q**.

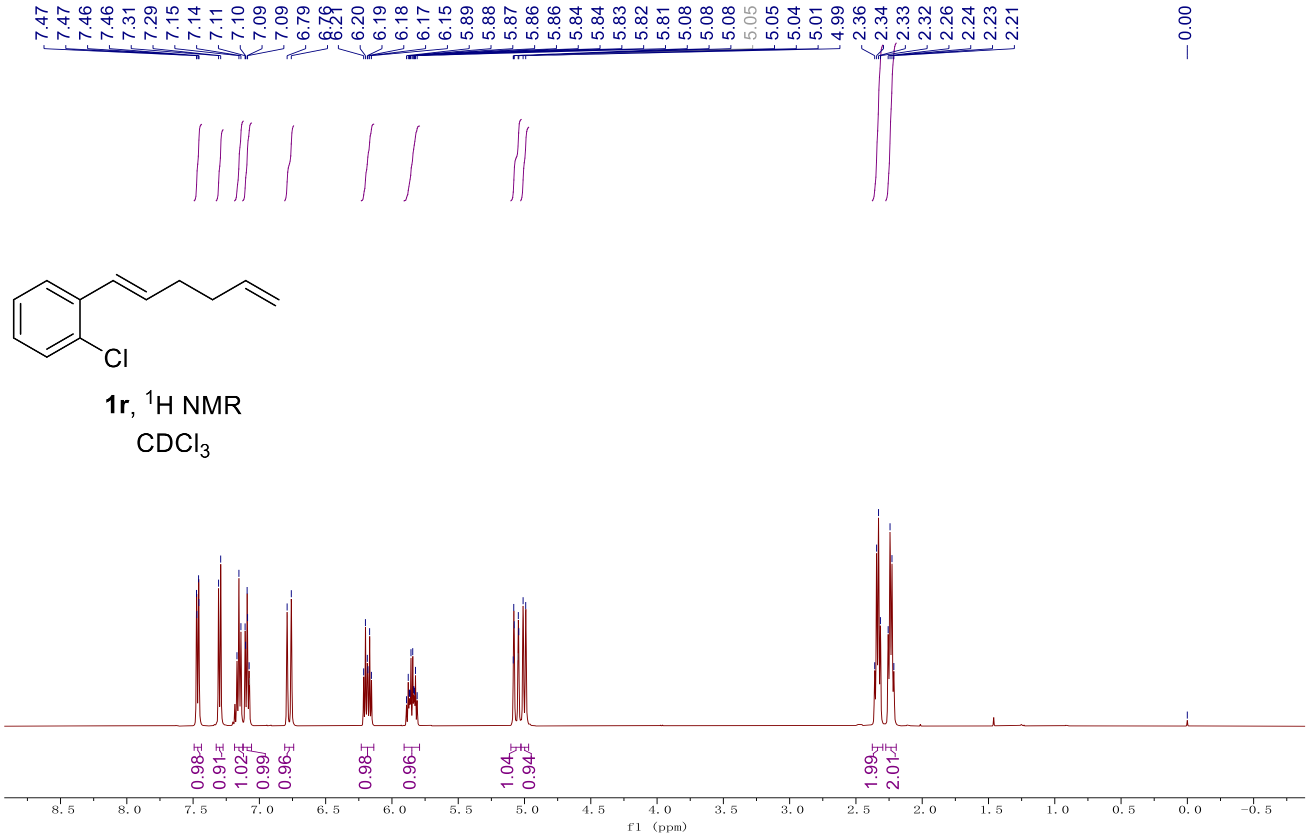

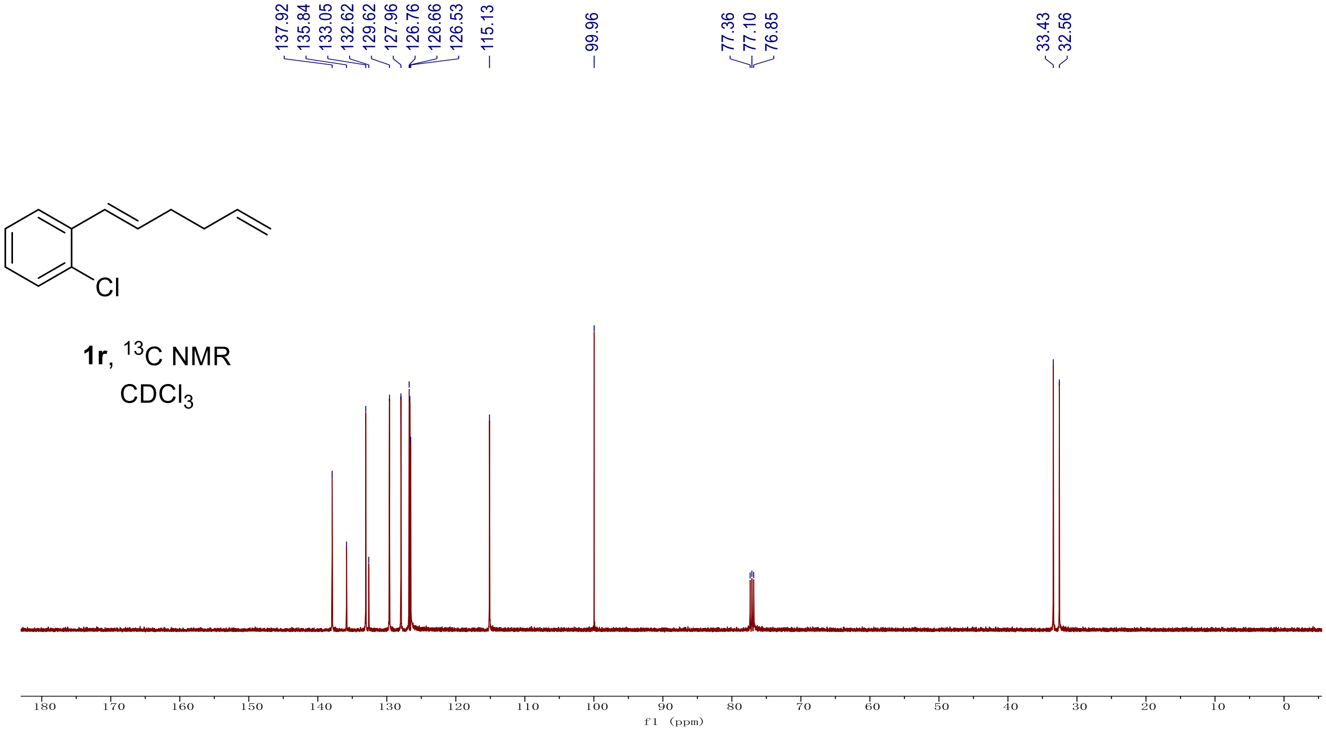

Supplementary Fig. 74. ^{1}H NMR and ^{13}C NMR spectra of compound **1r**.

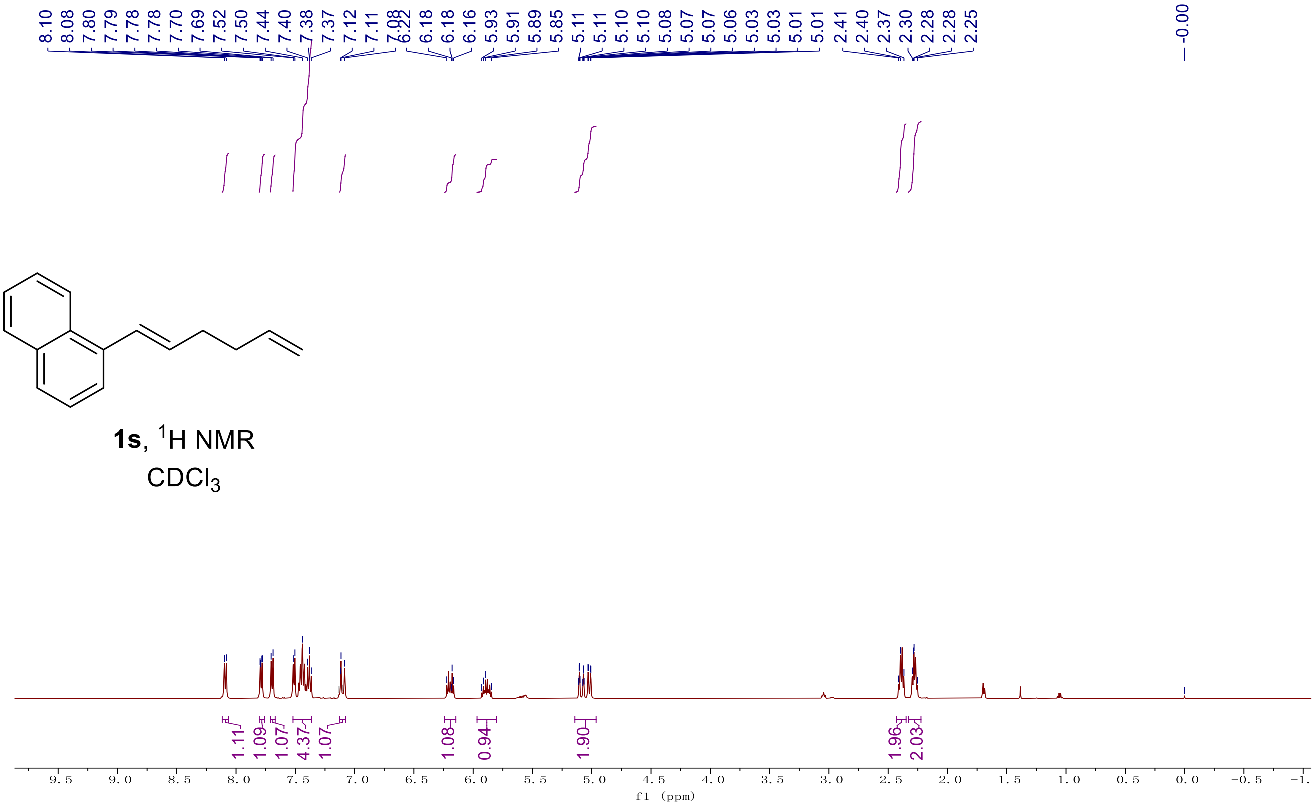

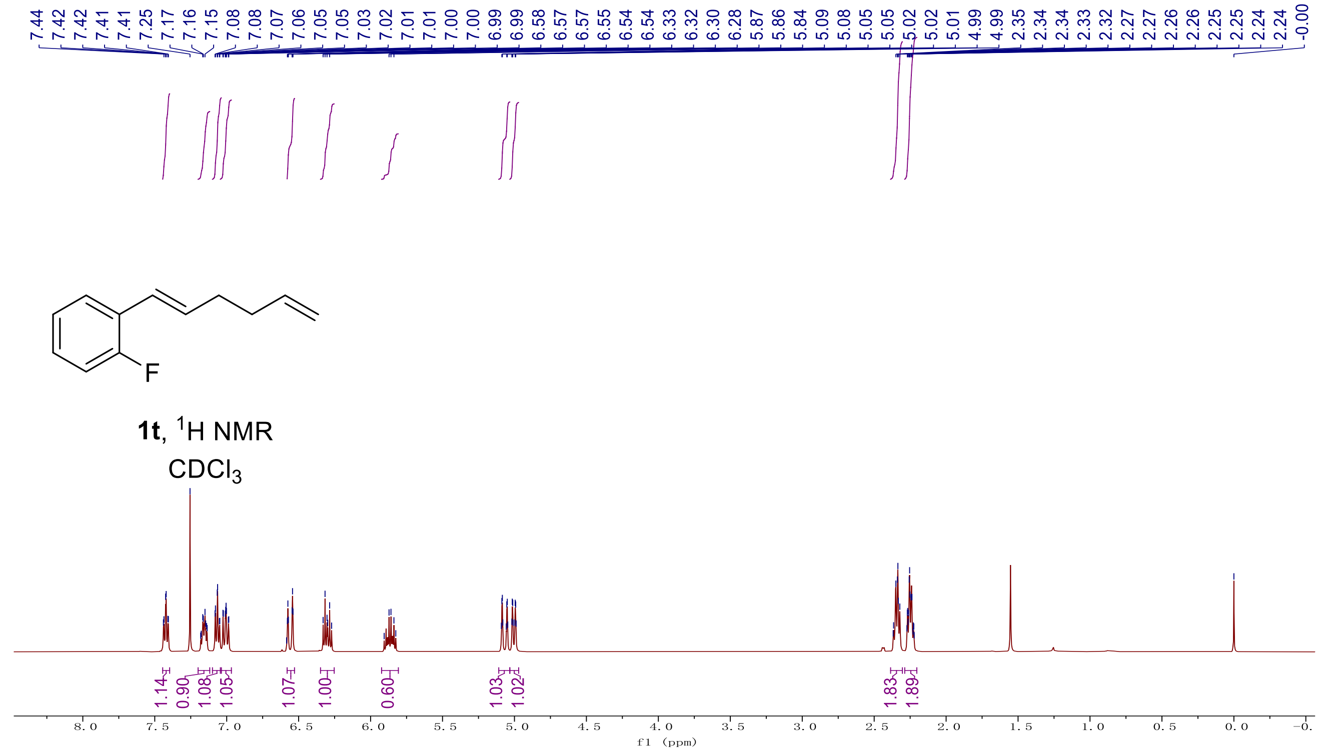

Supplementary Fig. 75. ^{1}H NMR spectra of compound **1s** and **1t**.

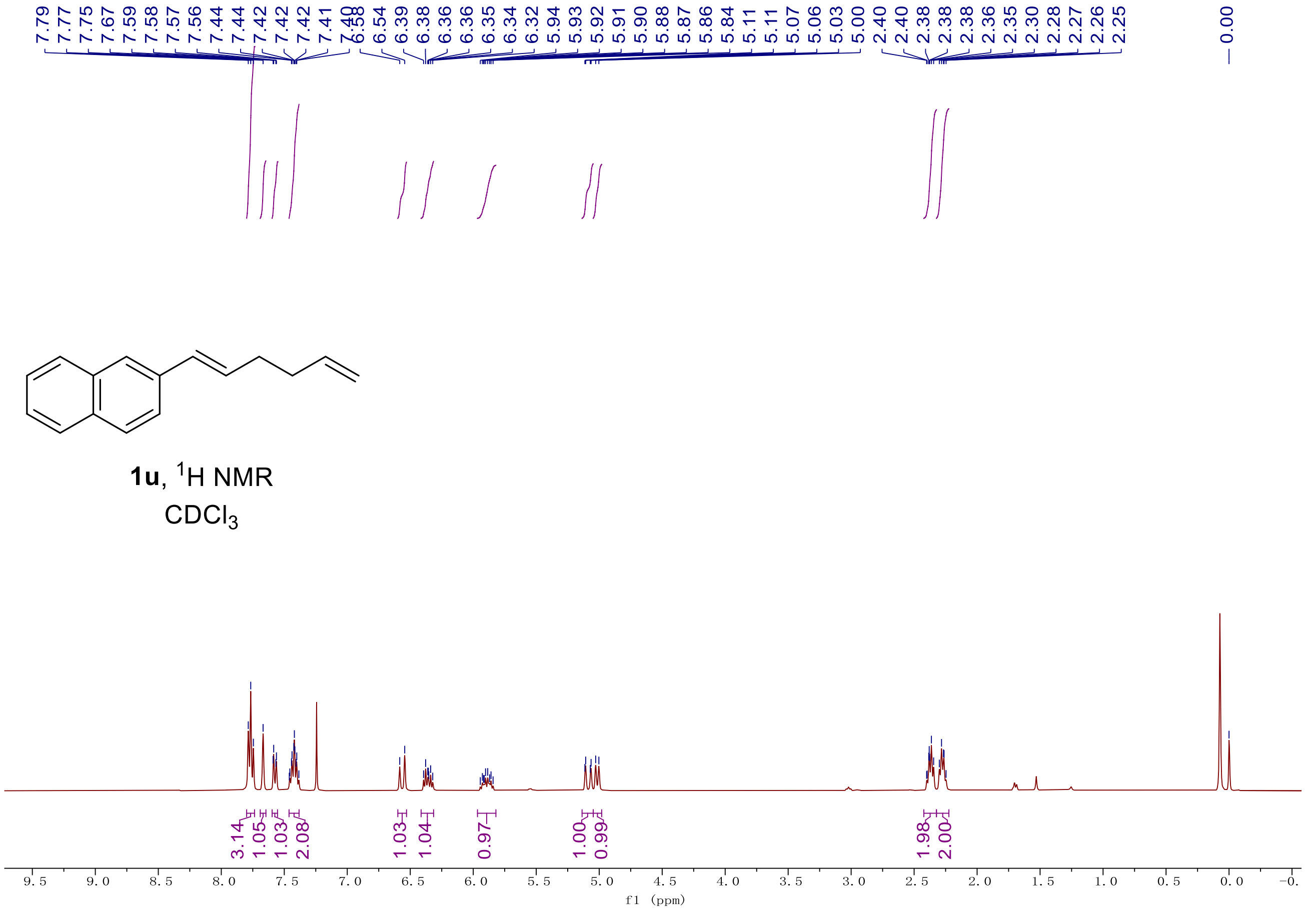

Supplementary Fig. 76. ^{1}H NMR spectra of compound **1u**.

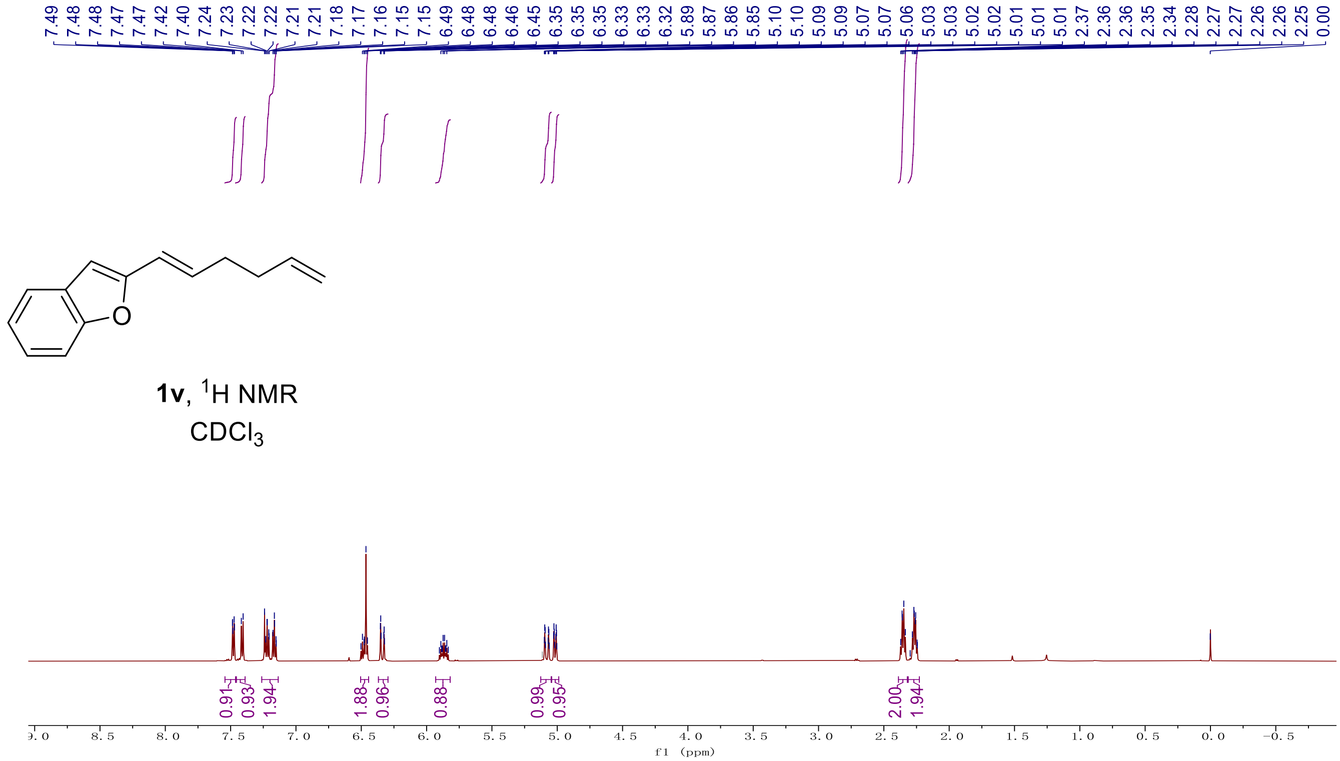

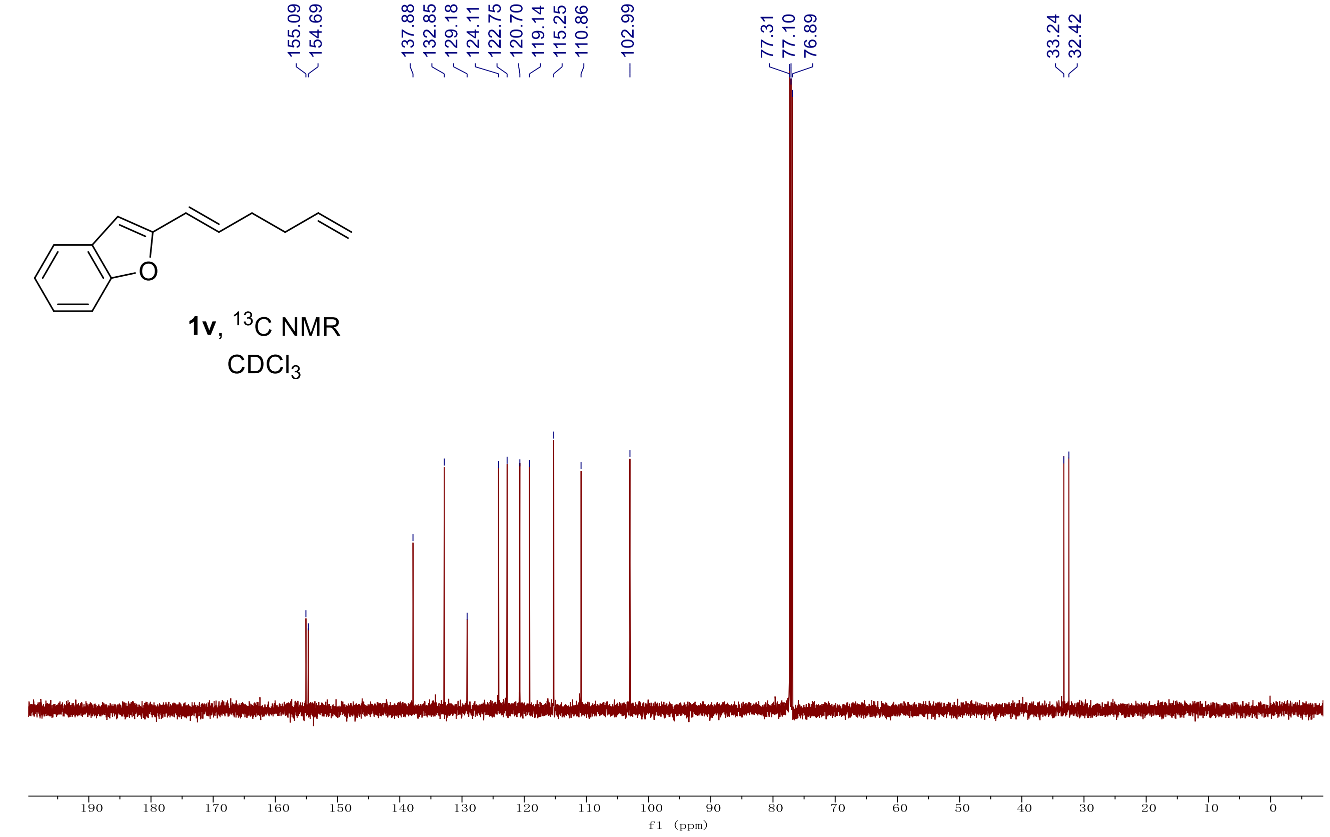

Supplementary Fig. 77. ^{1}H NMR and ^{13}C NMR spectra of compound **1v**.

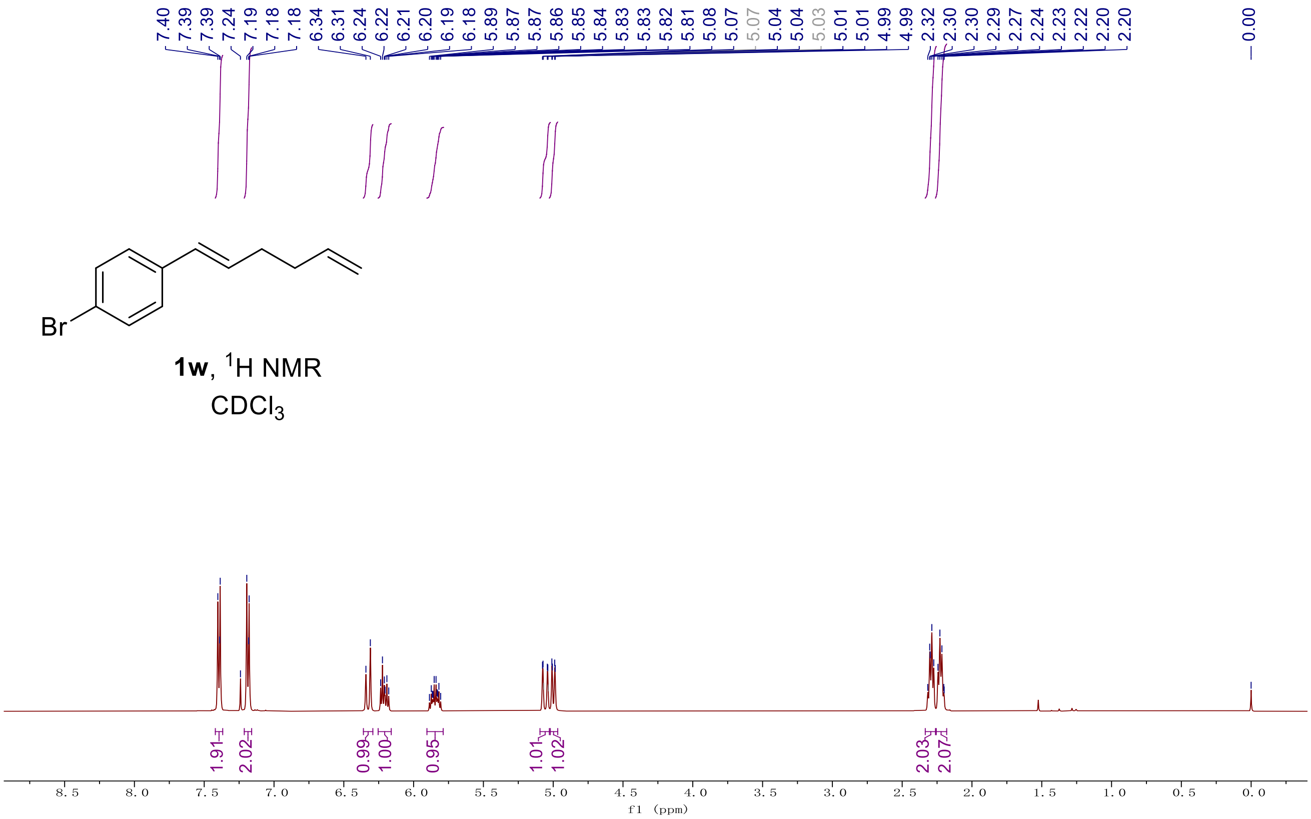

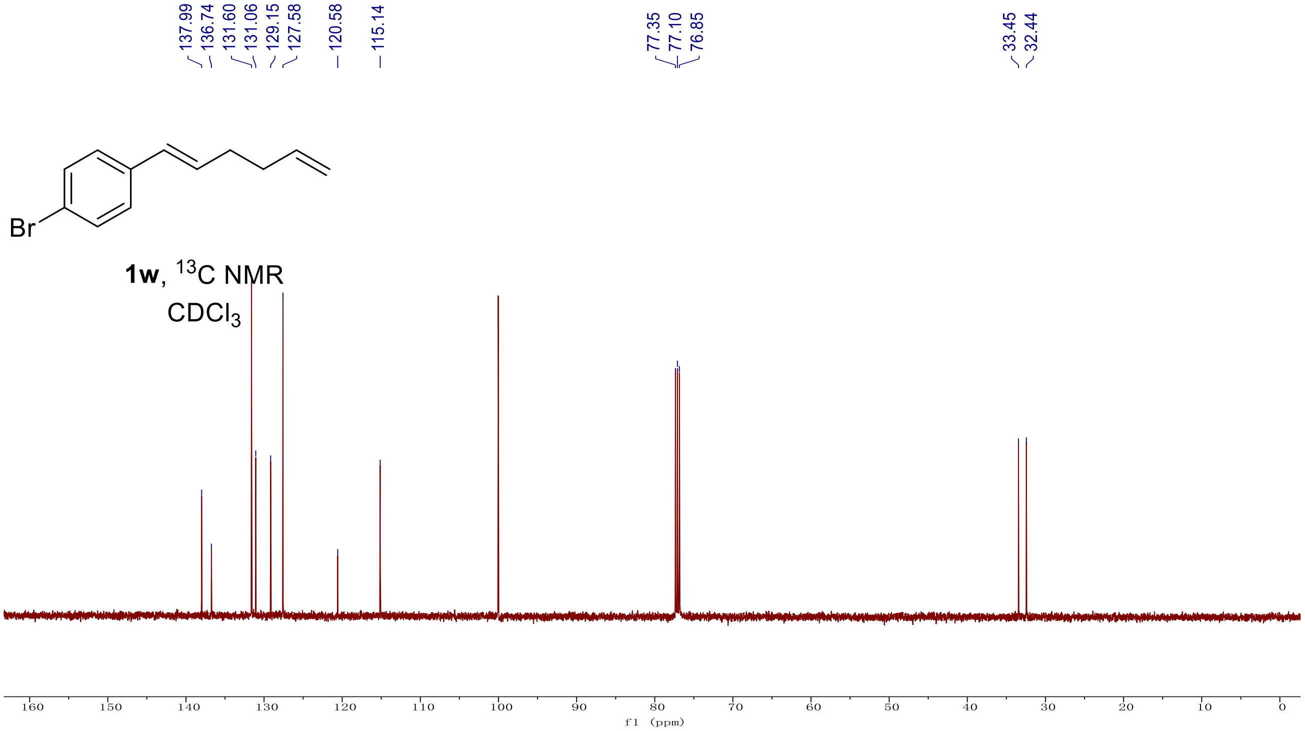

Supplementary Fig. 78. ^{1}H NMR and ^{13}C NMR spectra of compound **1w**.

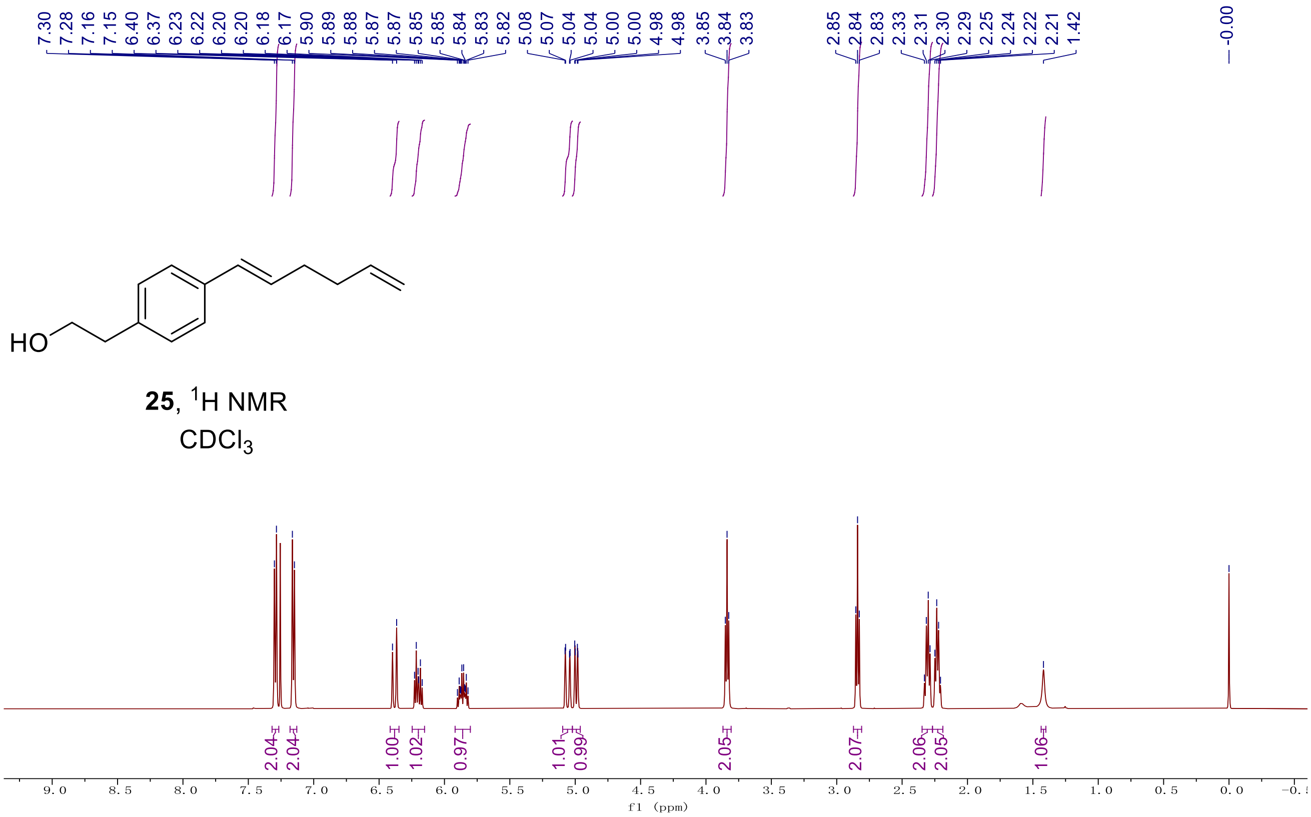

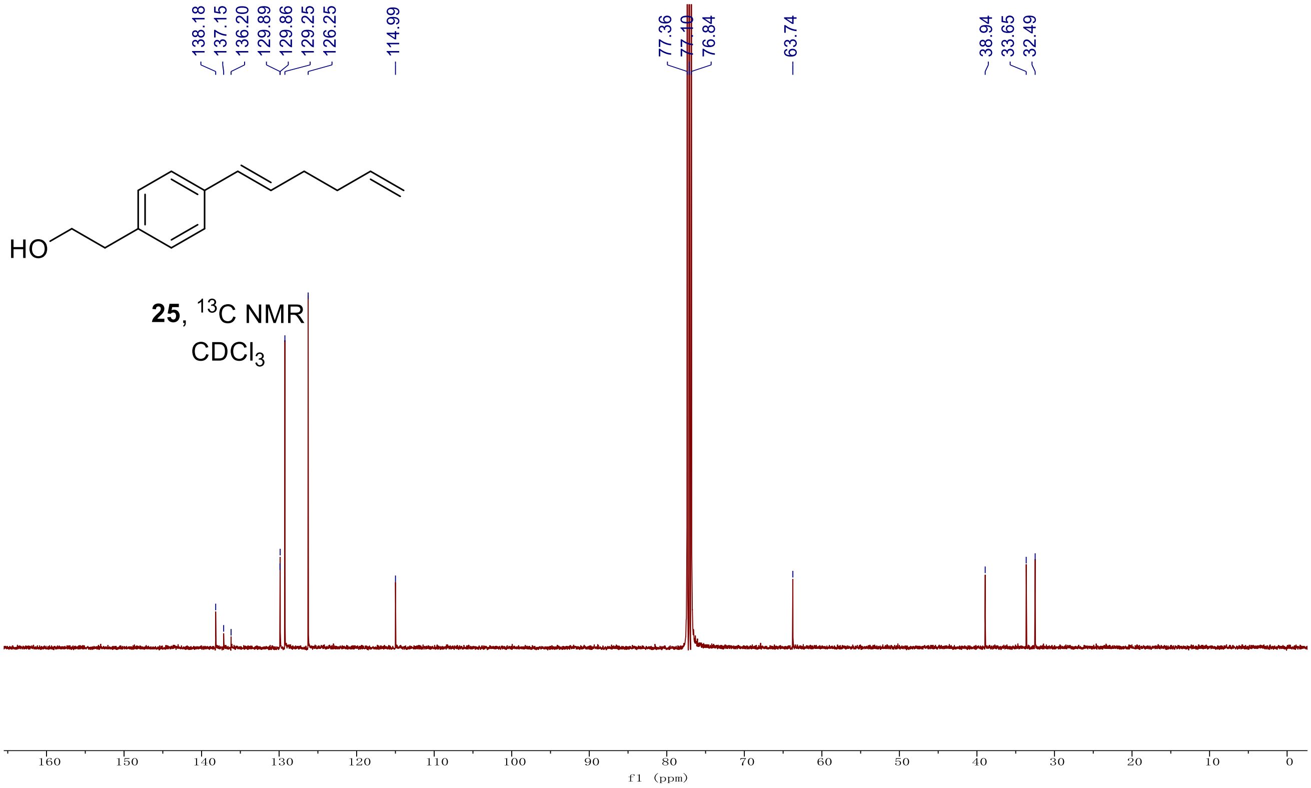

Supplementary Fig. 79. ^{1}H NMR and ^{13}C NMR spectra of compound **25**.

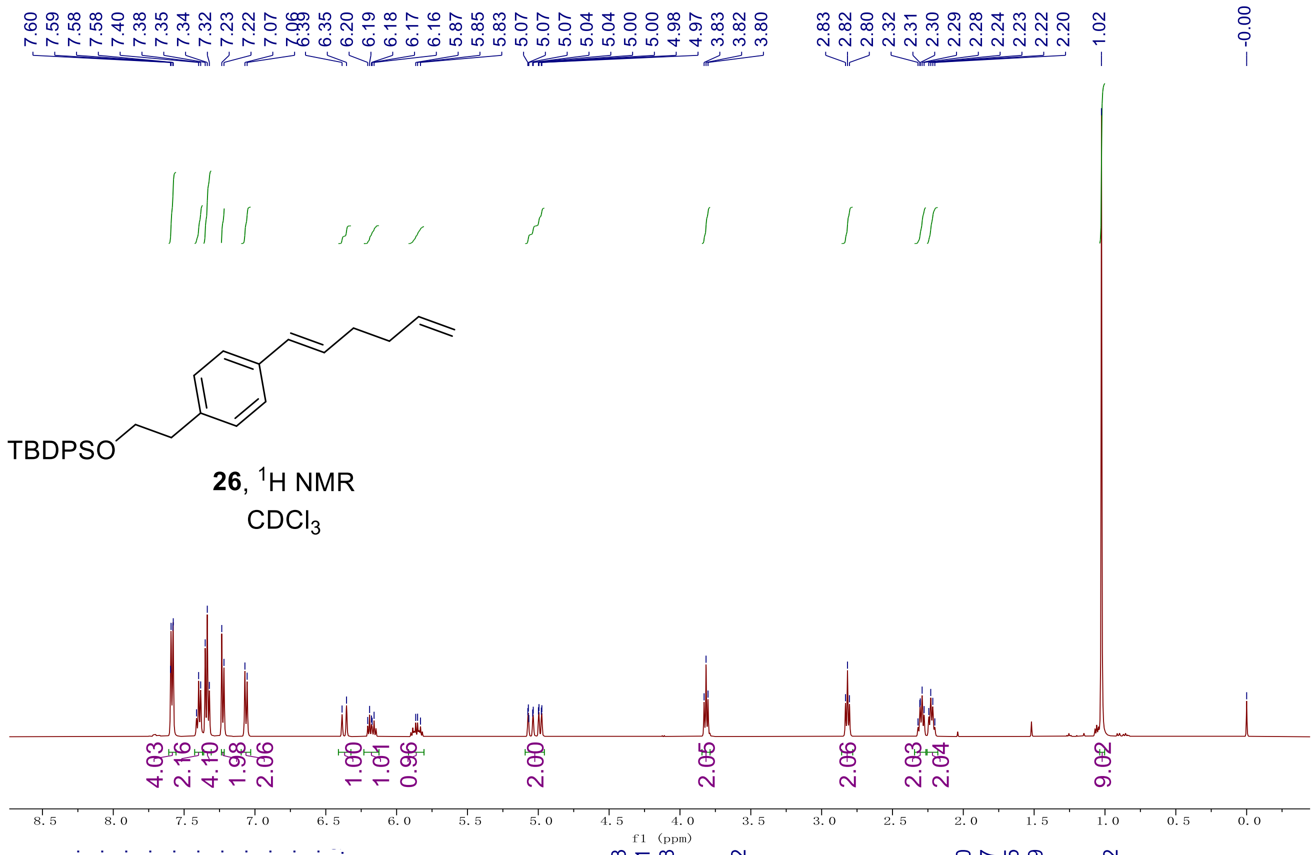

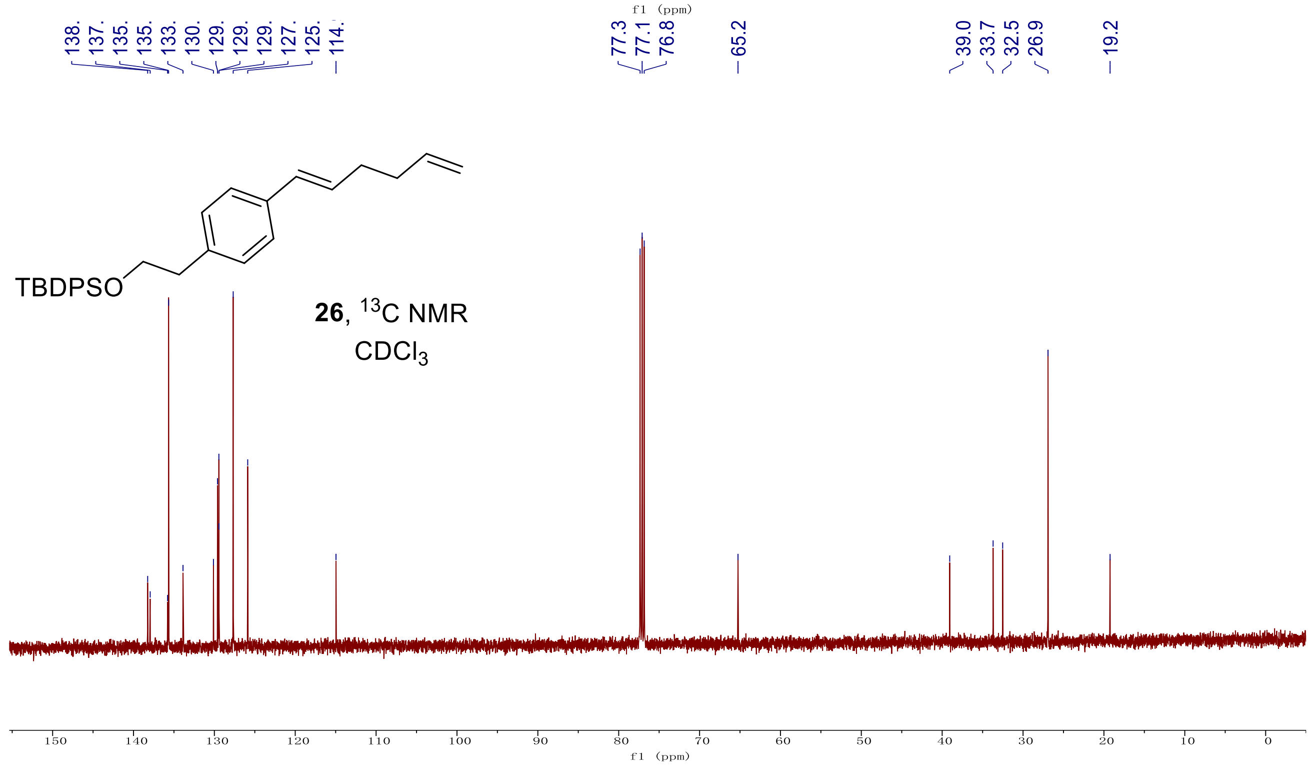

Supplementary Fig. 80. ^{1}H NMR and ^{13}C NMR spectra of compound **26**.

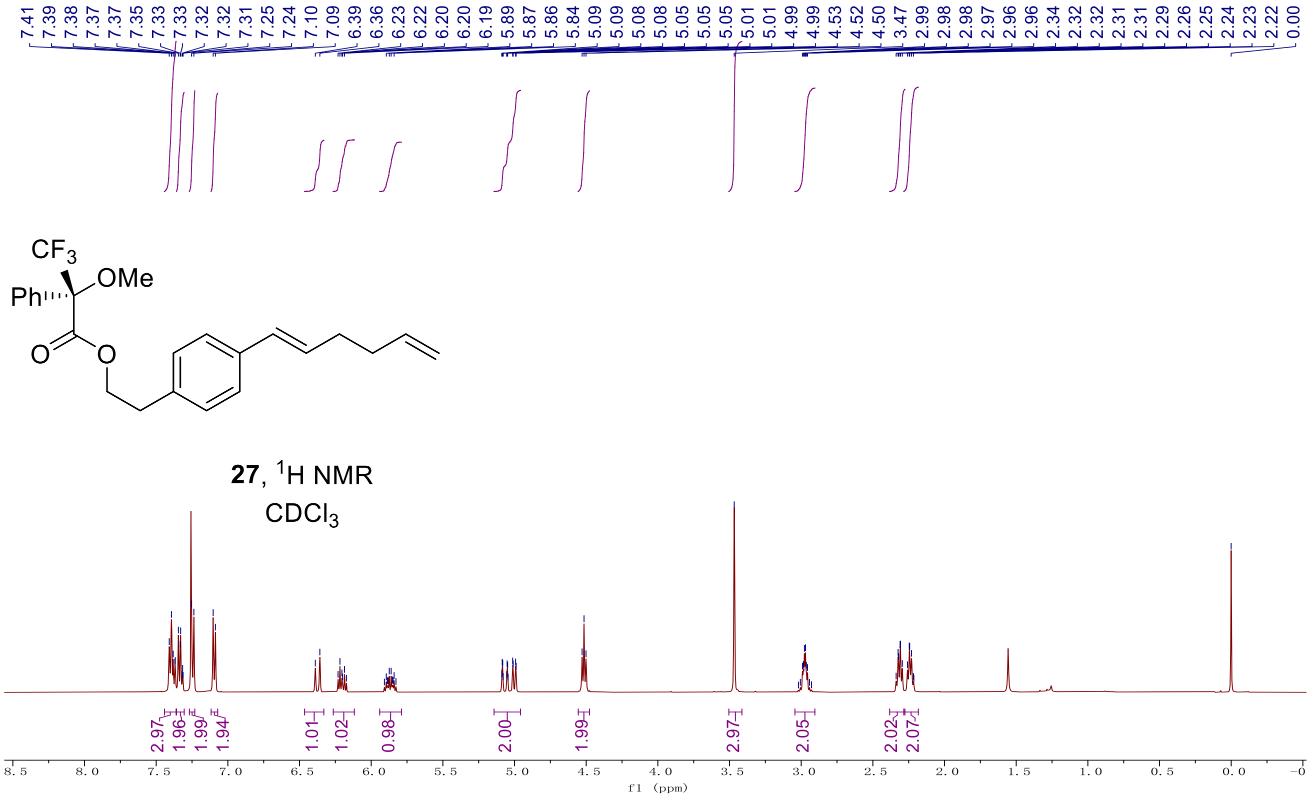

27, ^{1}H NMR
$CDCl_3$

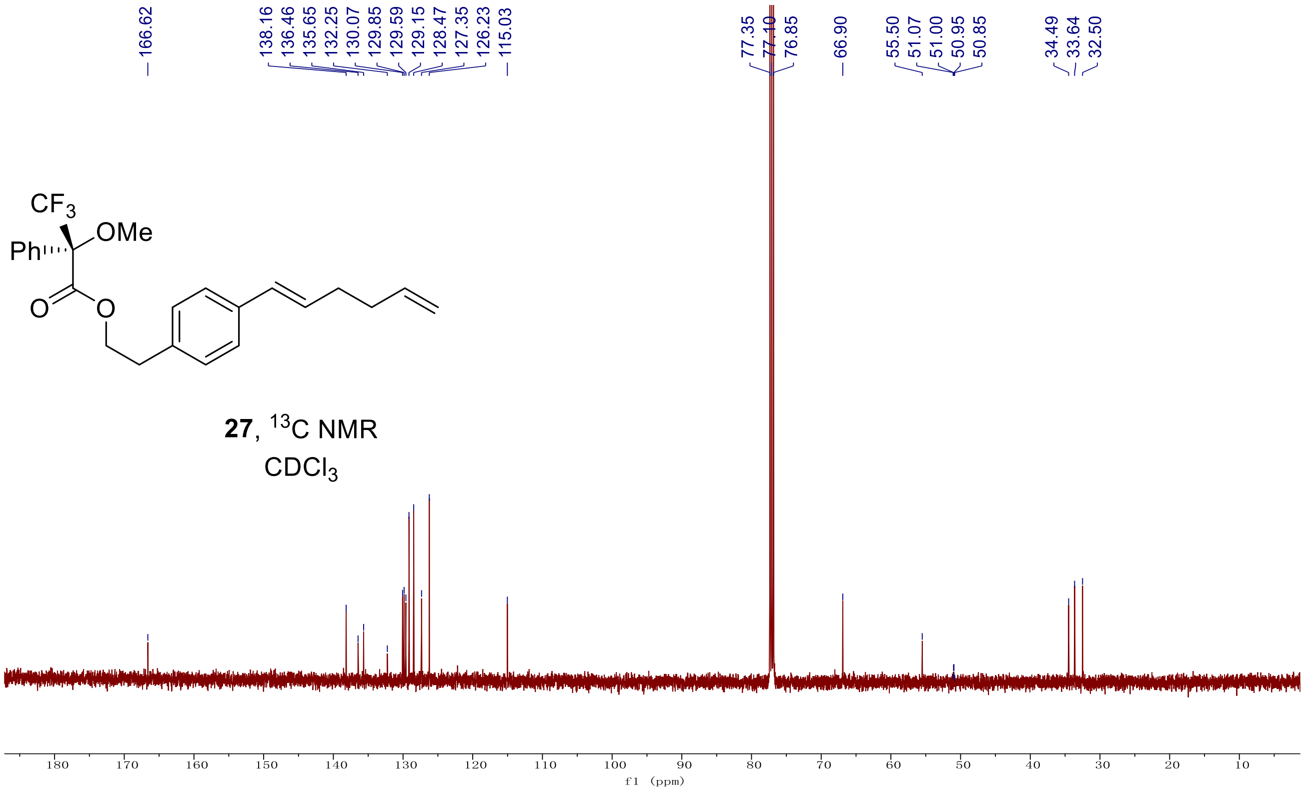

27, ^{13}C NMR
$CDCl_3$

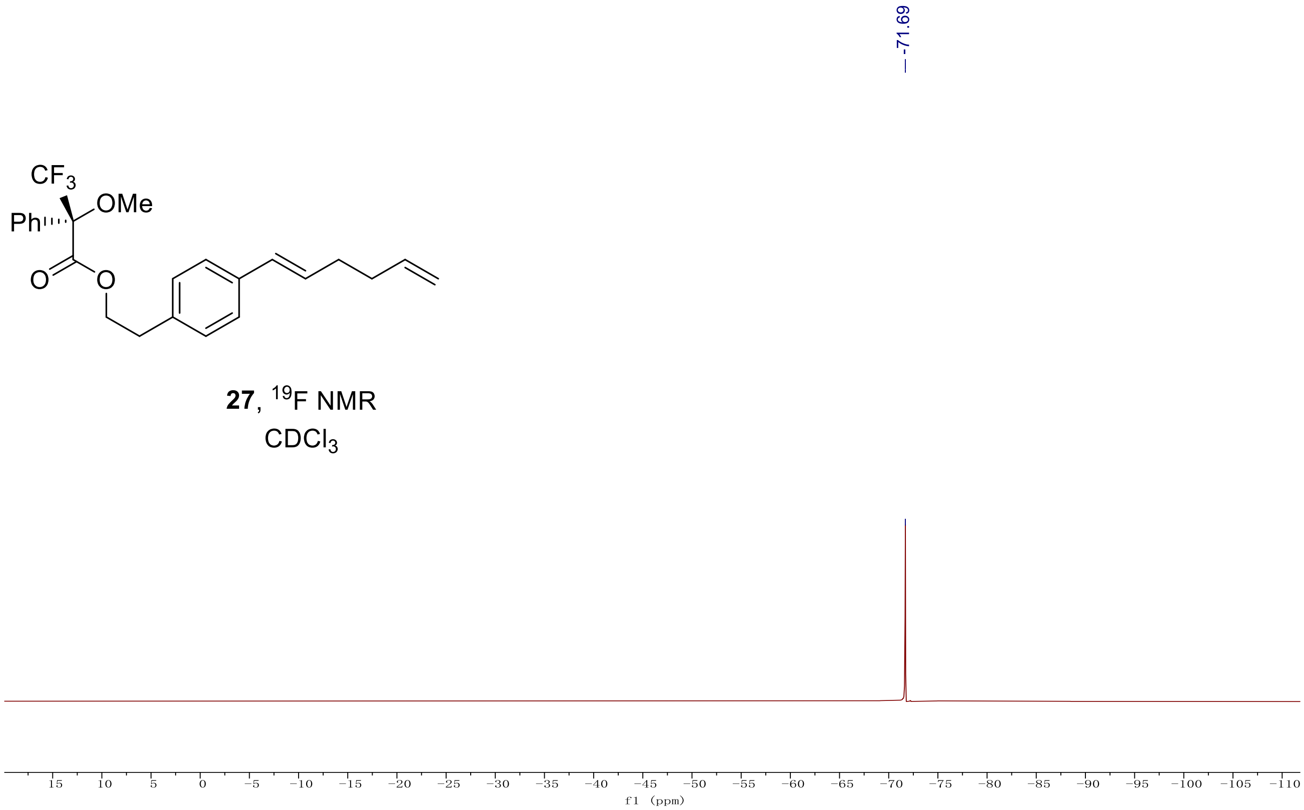

Supplementary Fig. 81. ^{1}H NMR, ^{13}C NMR and ^{19}F NMR spectra of compound **27**.

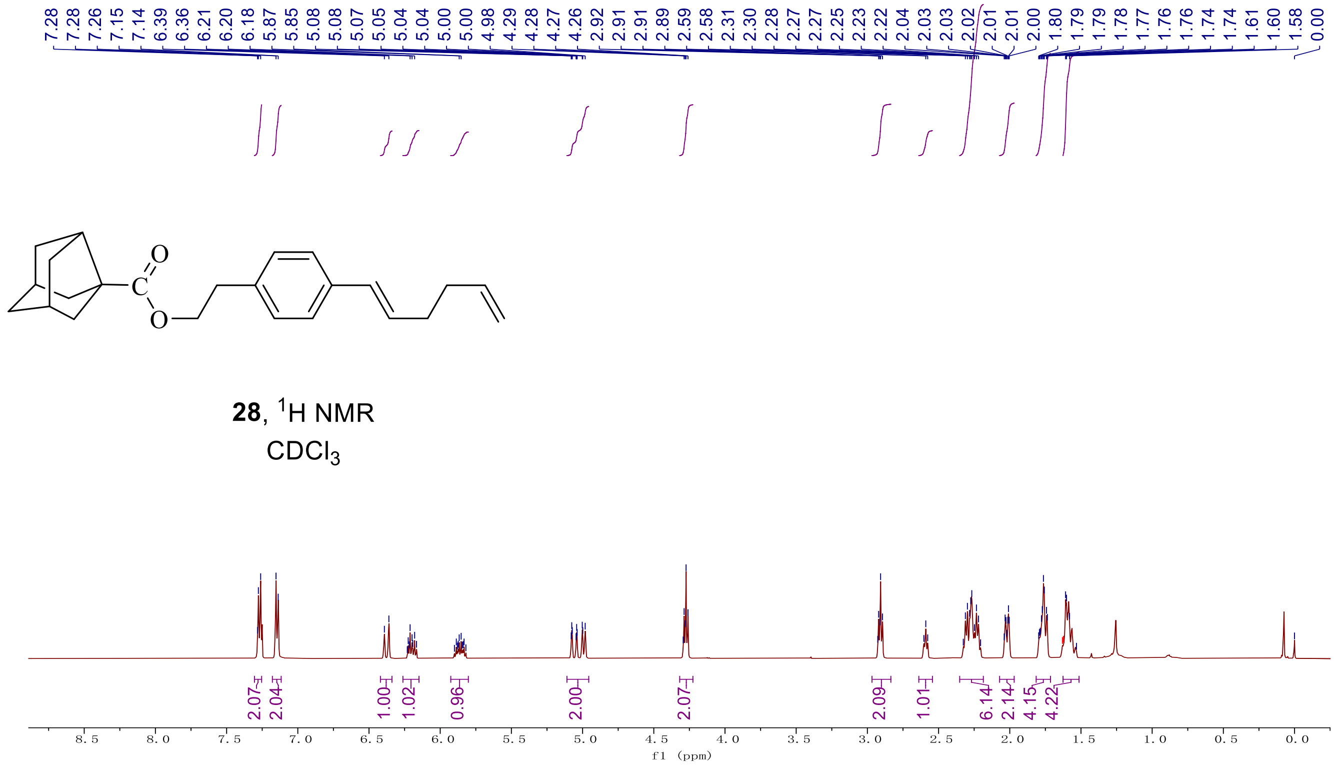

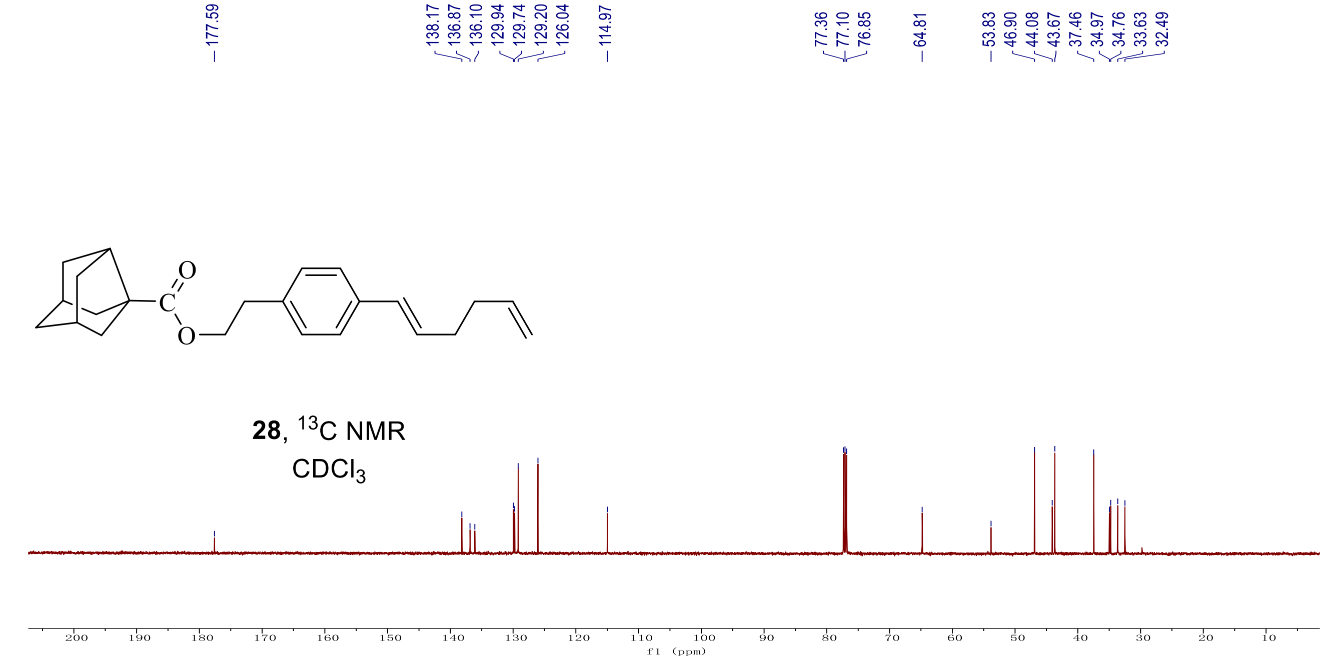

Supplementary Fig. 82. ^{1}H NMR, ^{13}C NMR and ^{19}F NMR spectra of compound **28**.

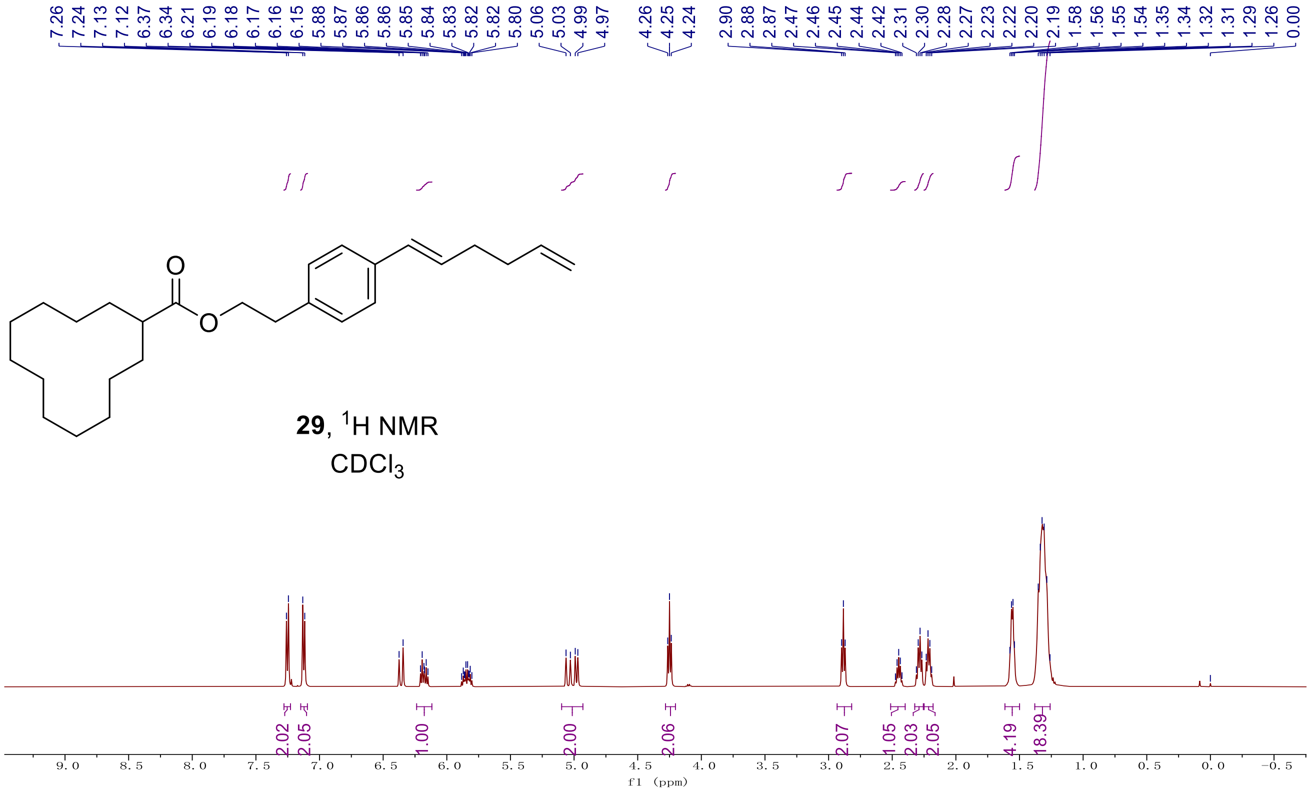

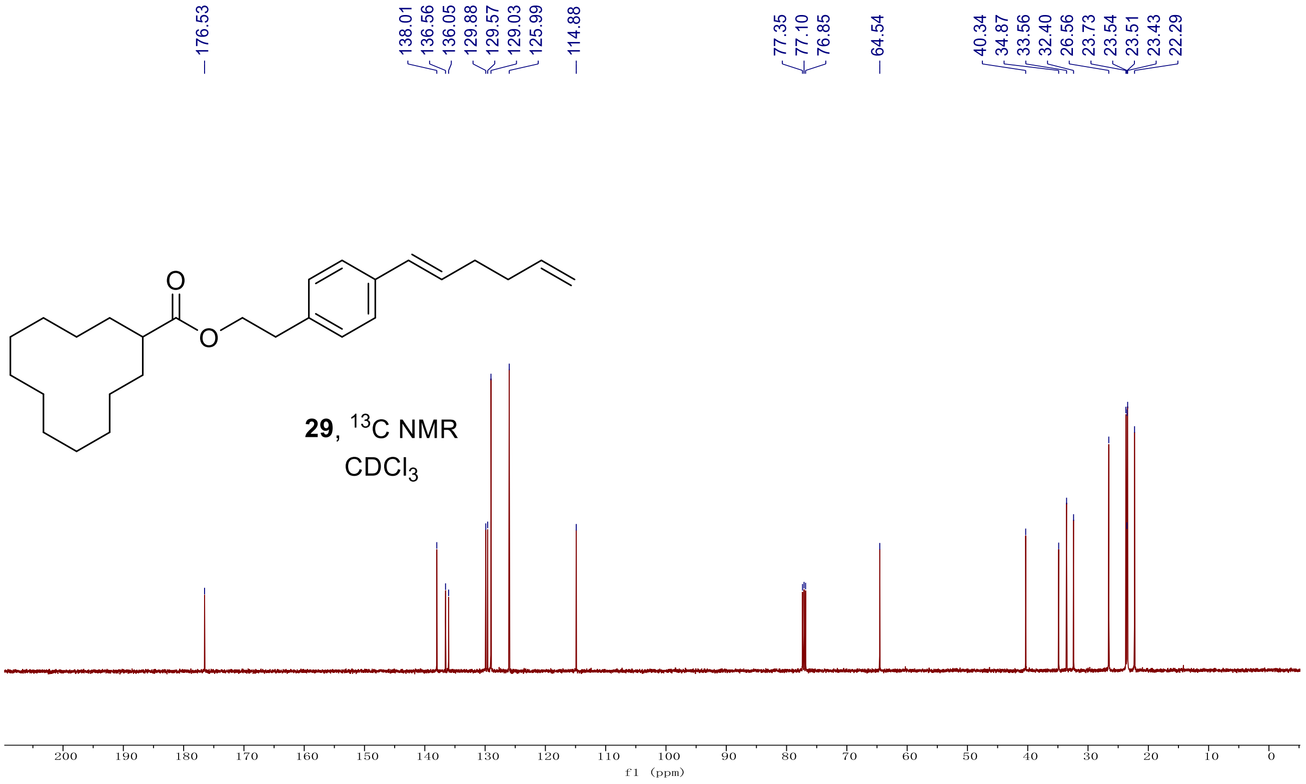

Supplementary Fig. 83. ^{1}H NMR and ^{13}C NMR spectra of compound **29**.

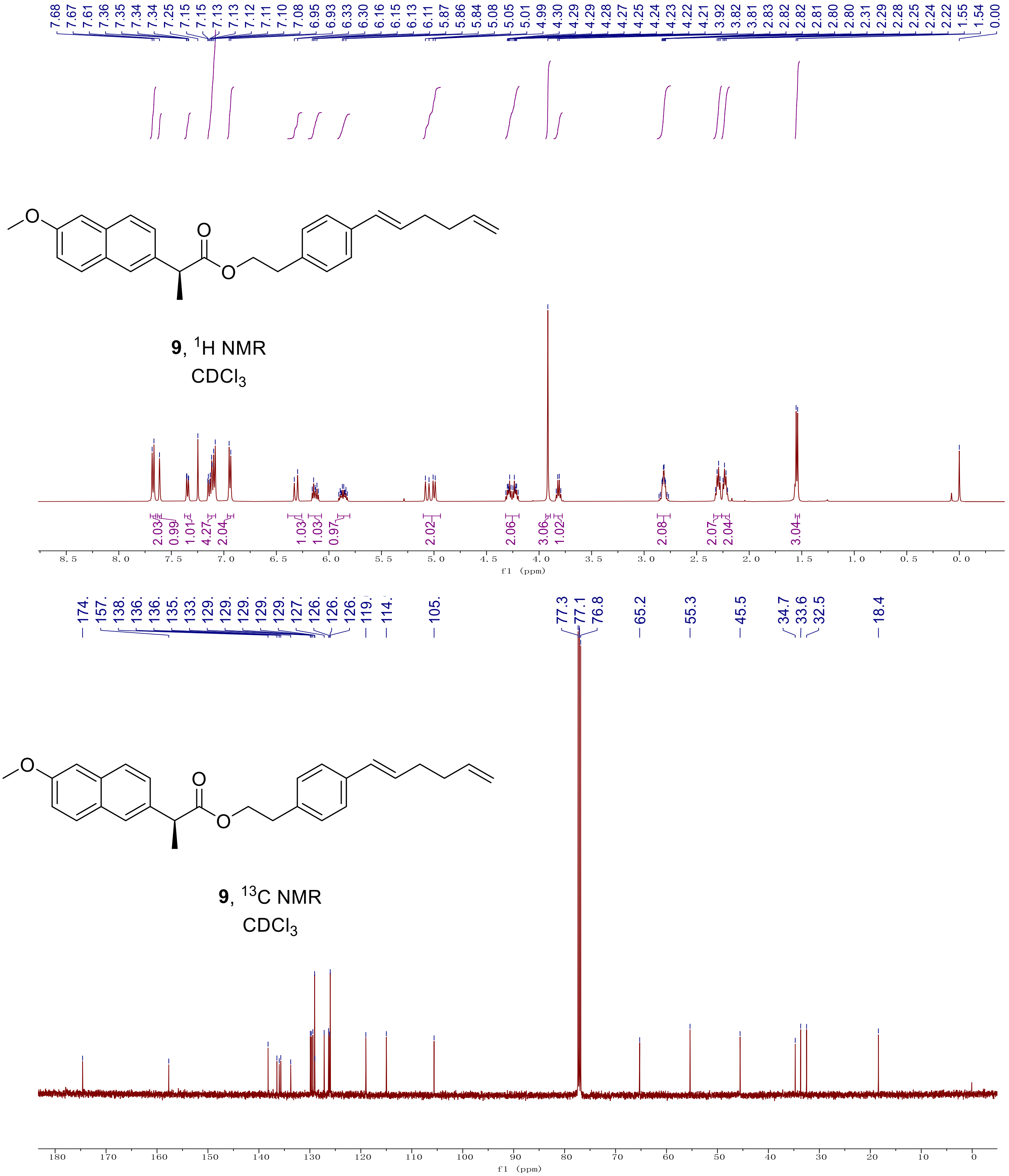

Supplementary Fig. 84. ^{1}H NMR and ^{13}C NMR spectra of compound **9**.

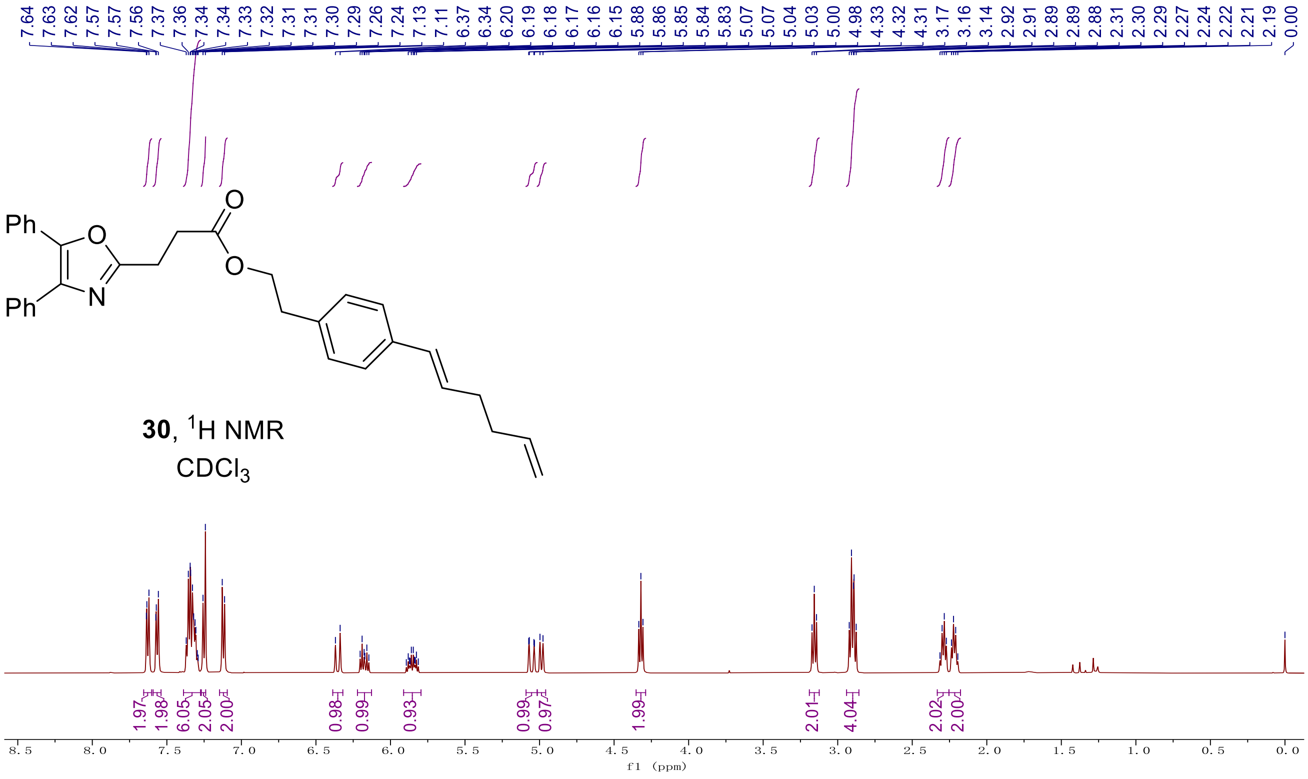

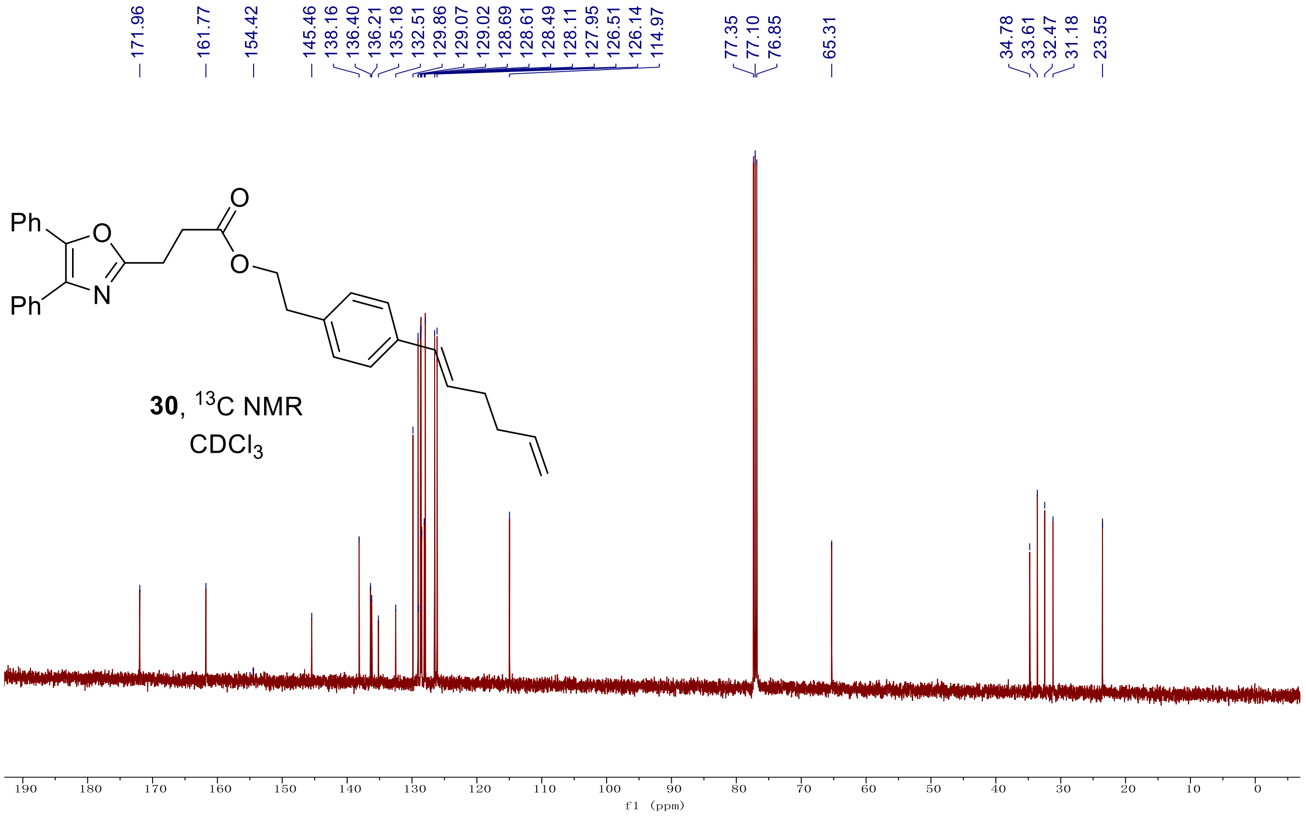

Supplementary Fig. 85. ^{1}H NMR and ^{13}C NMR spectra of compound **30**.

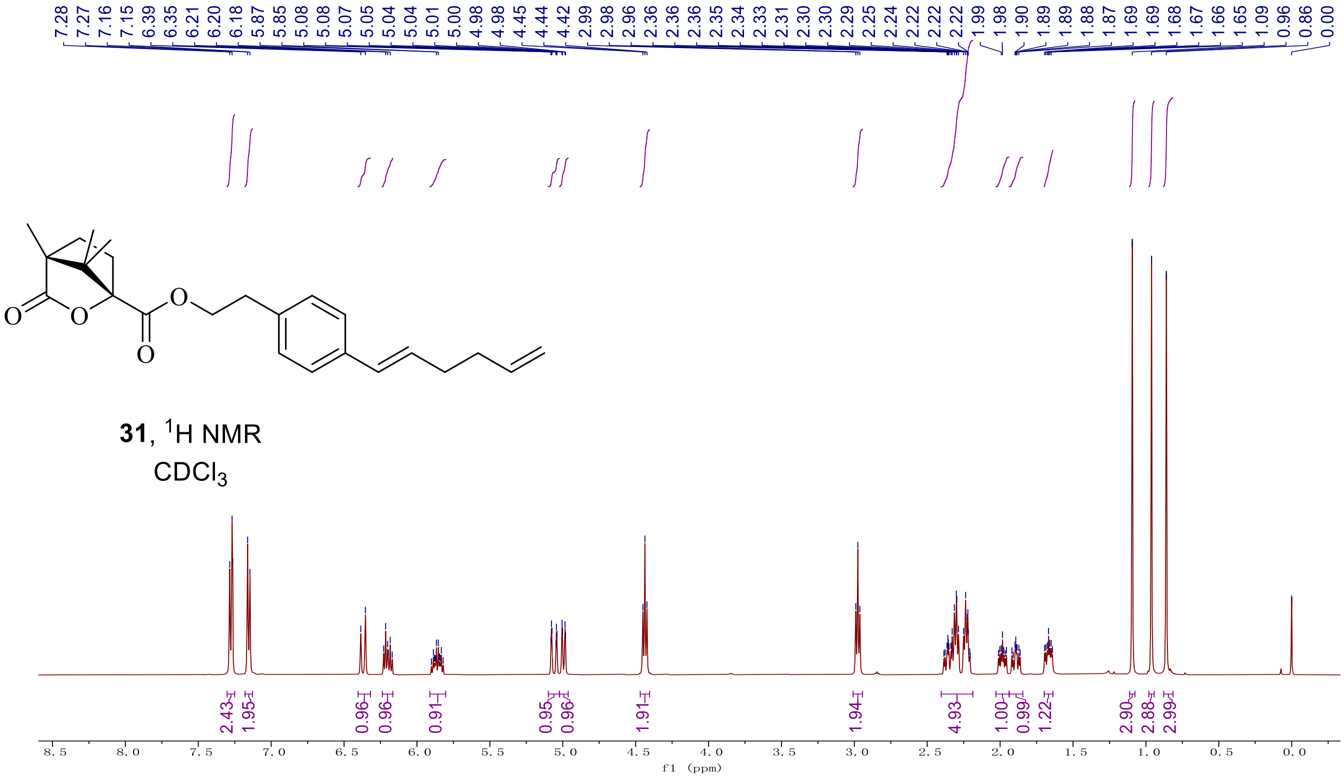

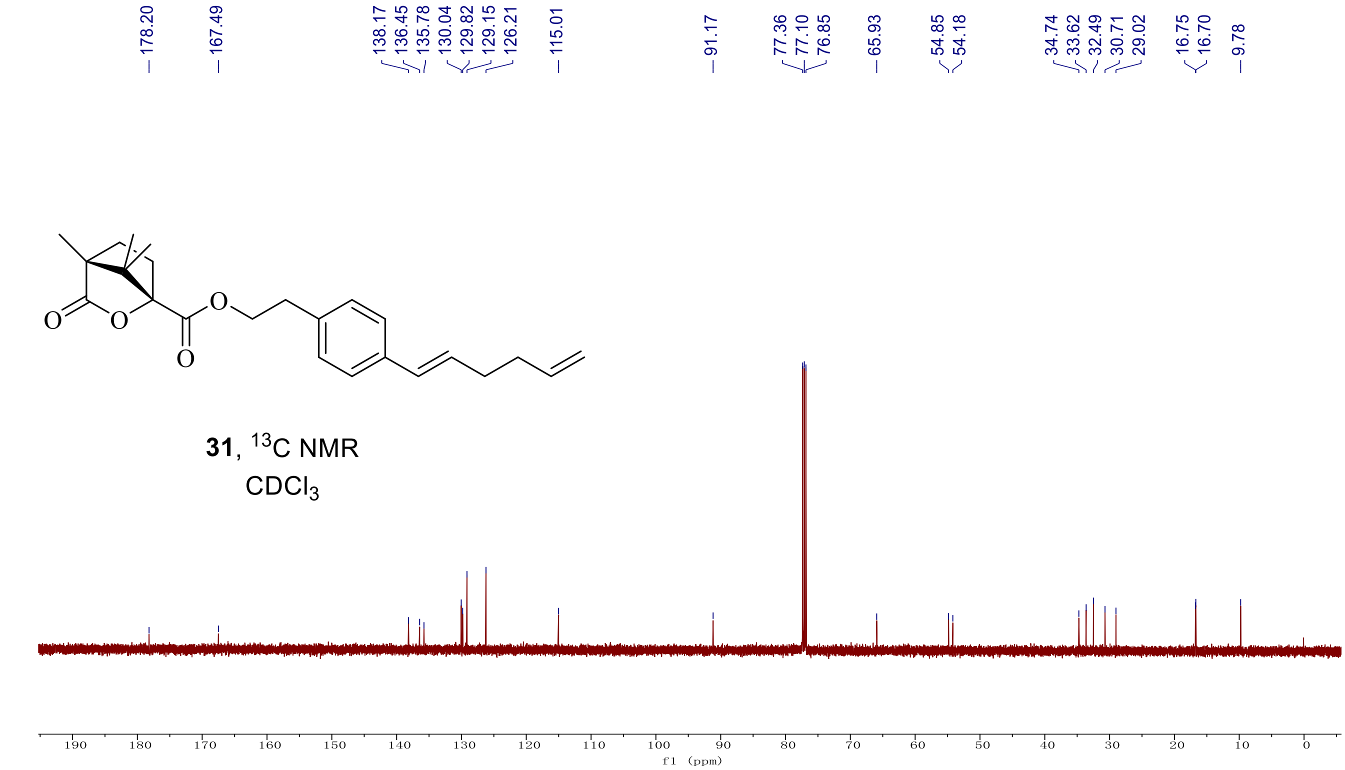

Supplementary Fig. 86. ^{1}H NMR and ^{13}C NMR spectra of compound **31**.

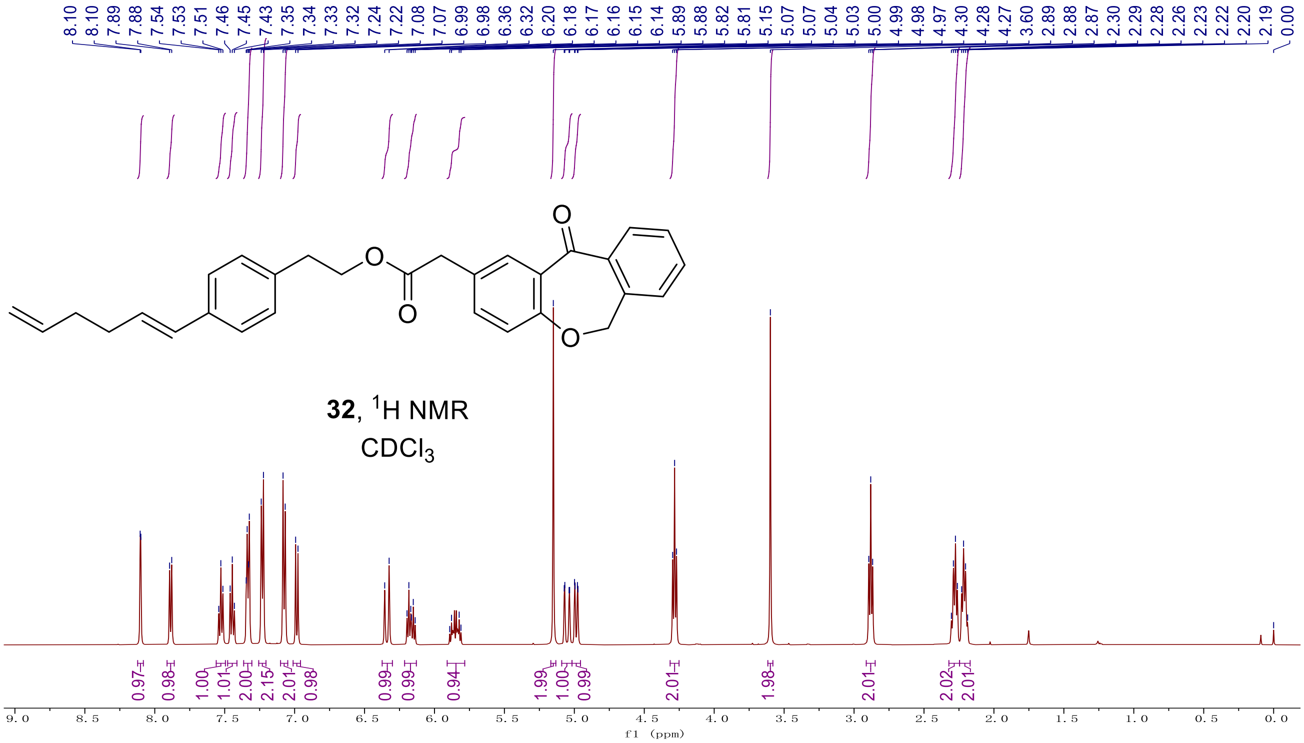

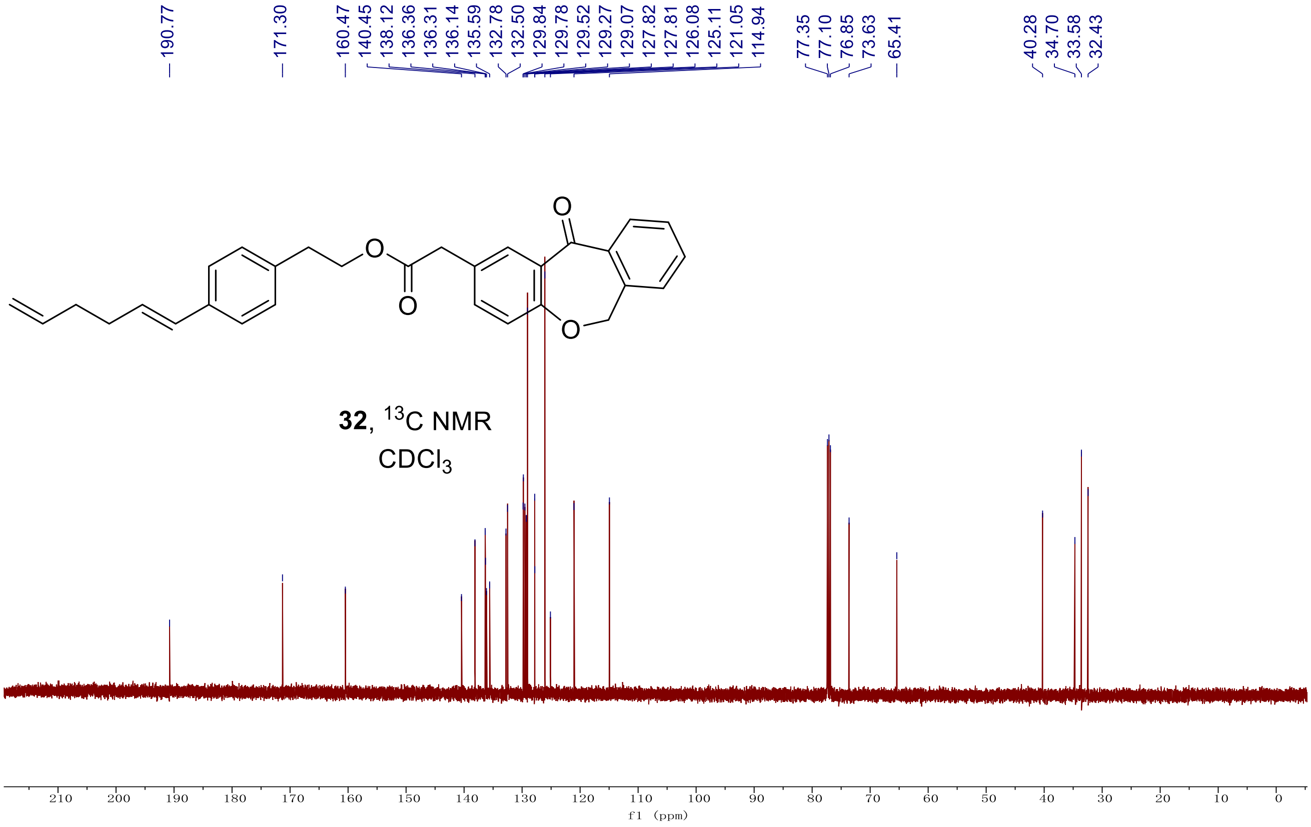

Supplementary Fig. 87. ^{1}H NMR and ^{13}C NMR spectra of compound **32**.

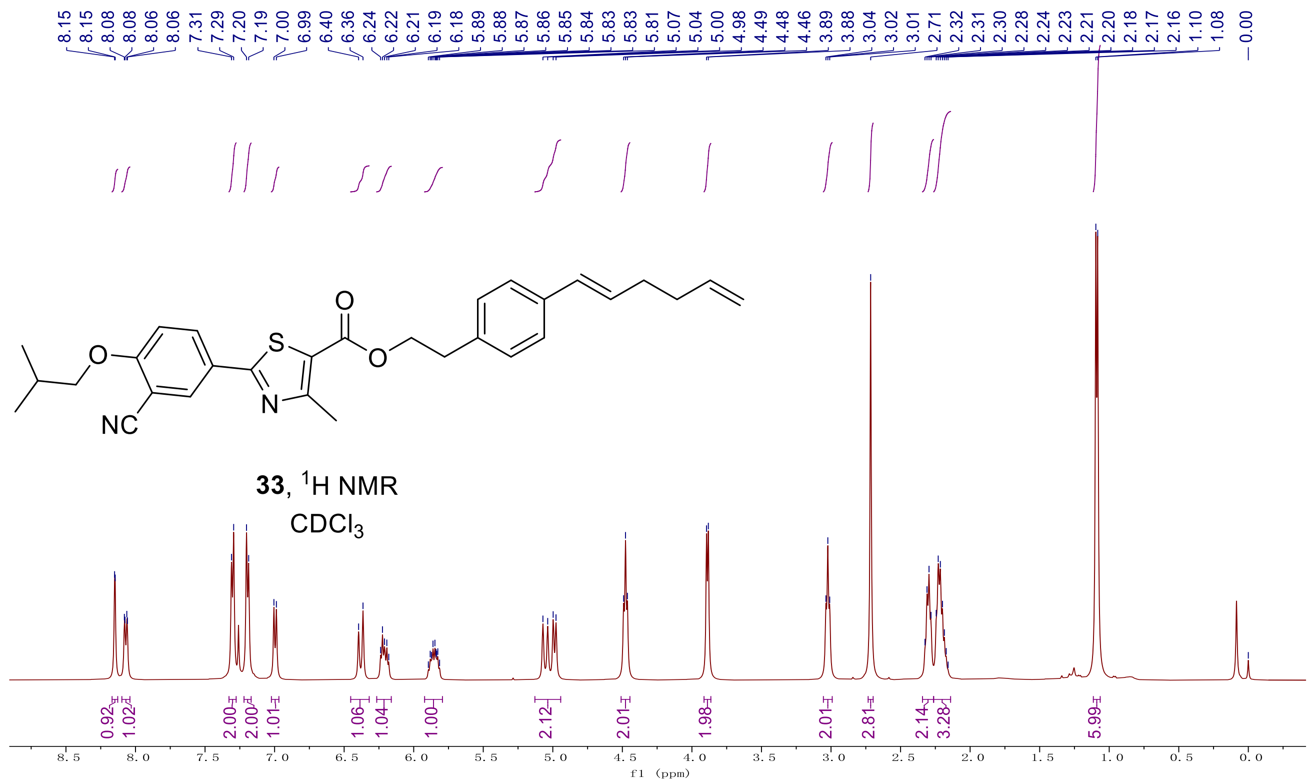

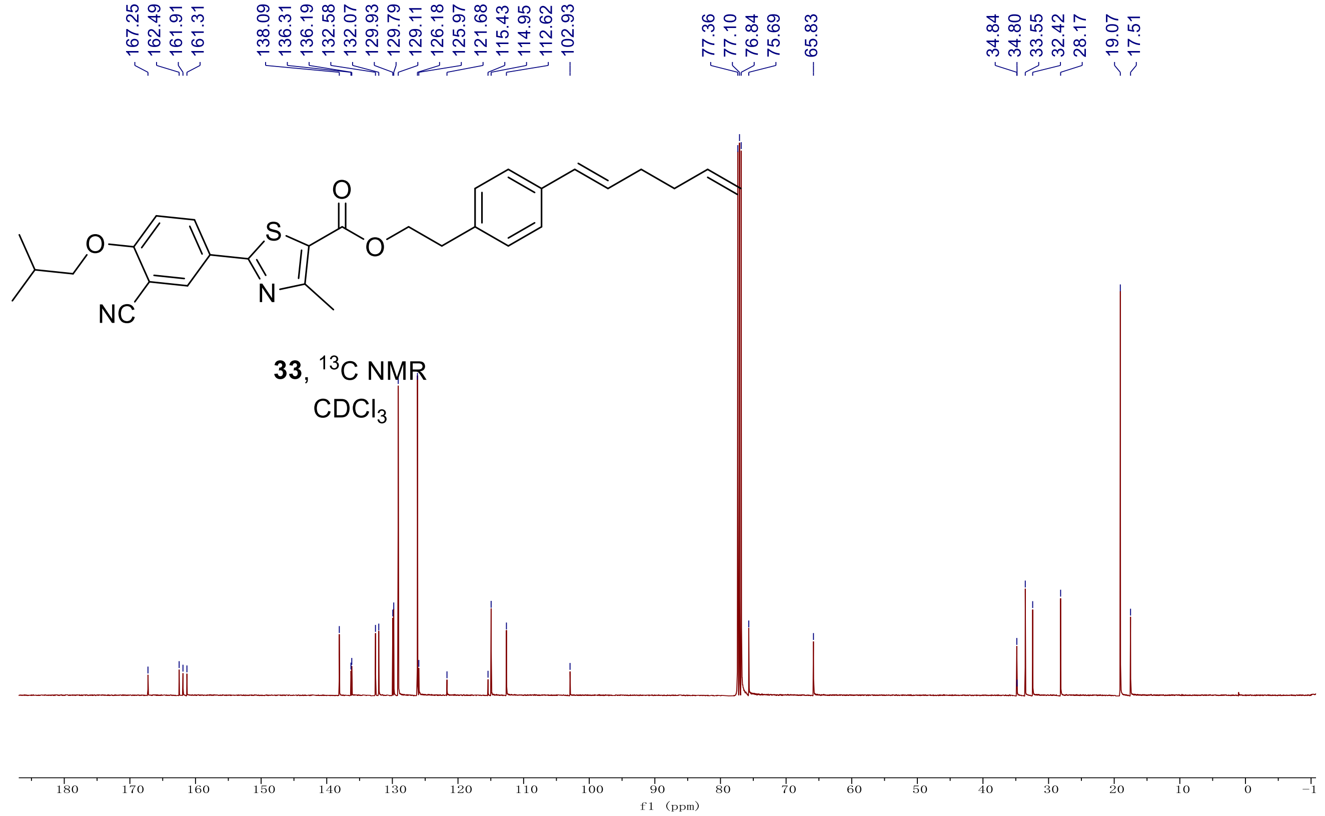

Supplementary Fig. 88. ^{1}H NMR and ^{13}C NMR spectra of compound **33**.

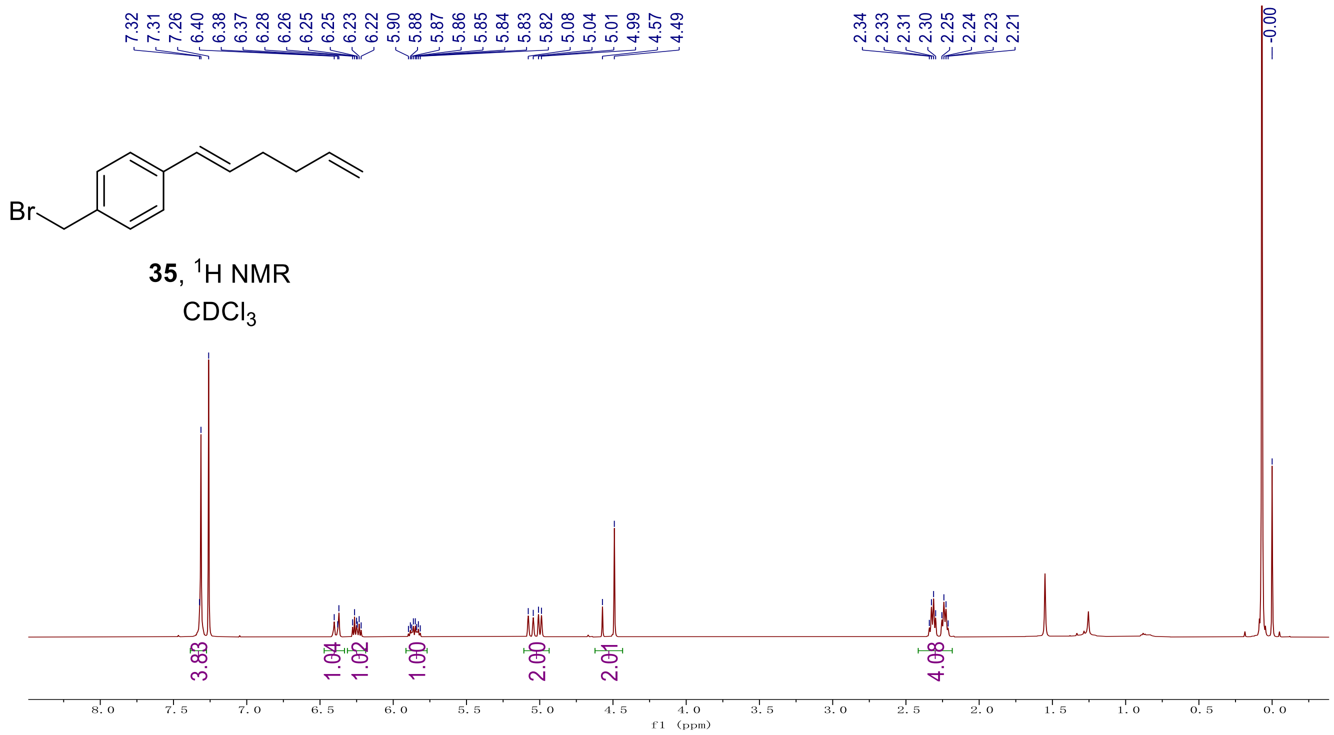

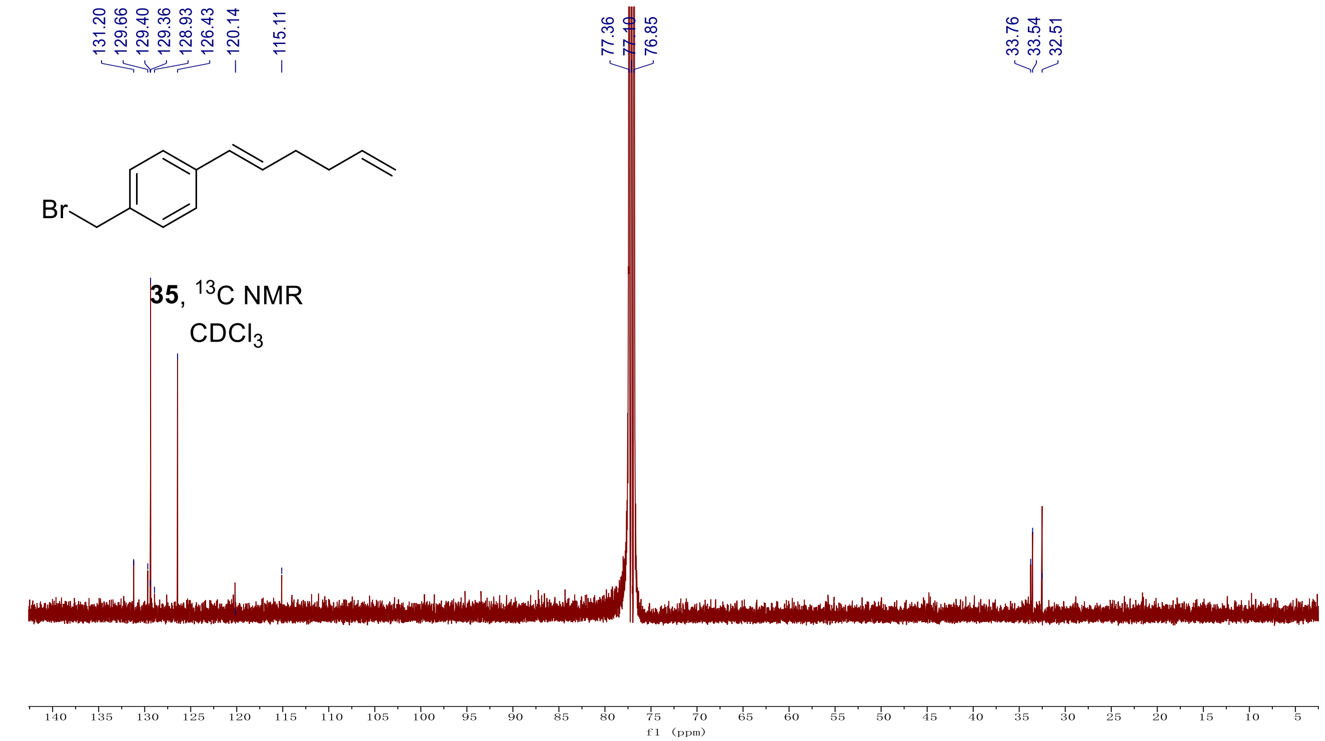

Supplementary Fig. 89. ^{1}H NMR and ^{13}C NMR spectra of compound **35**.

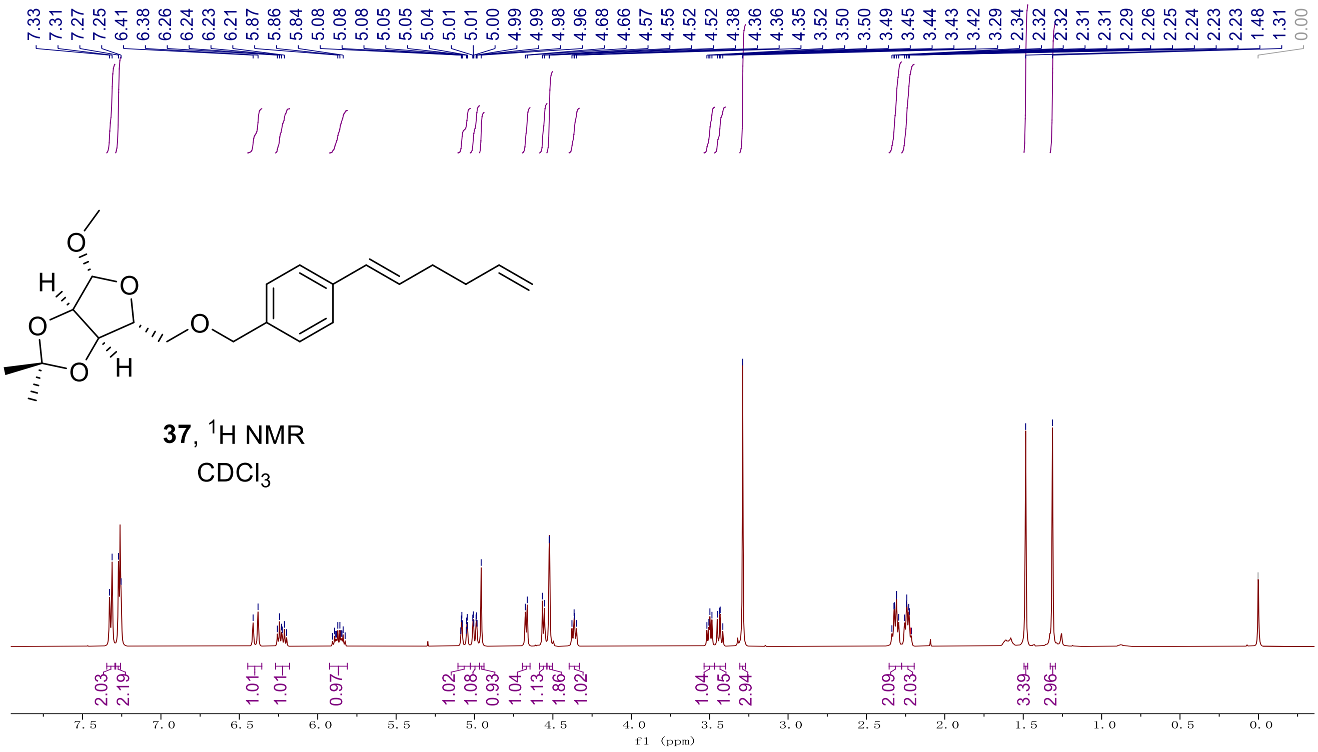

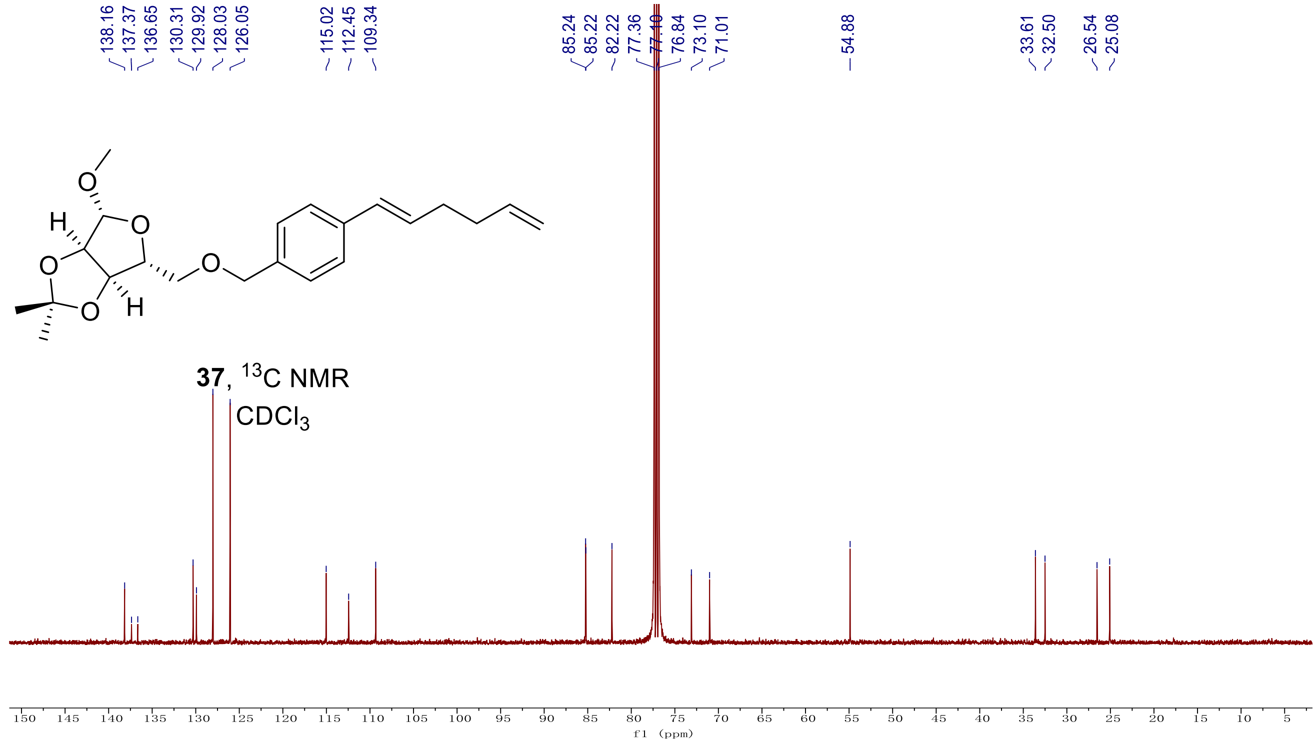

Supplementary Fig. 90. ^{1}H NMR and ^{13}C NMR spectra of compound **37**.

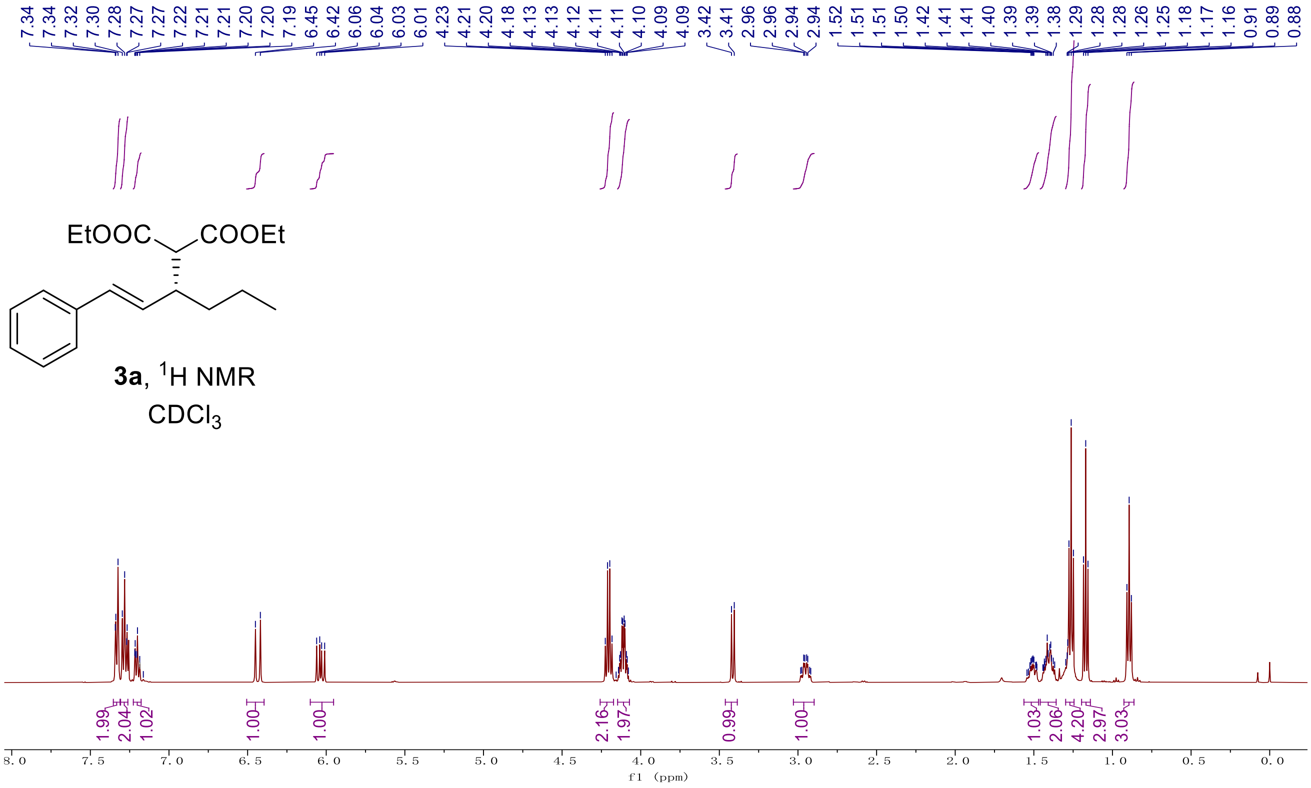

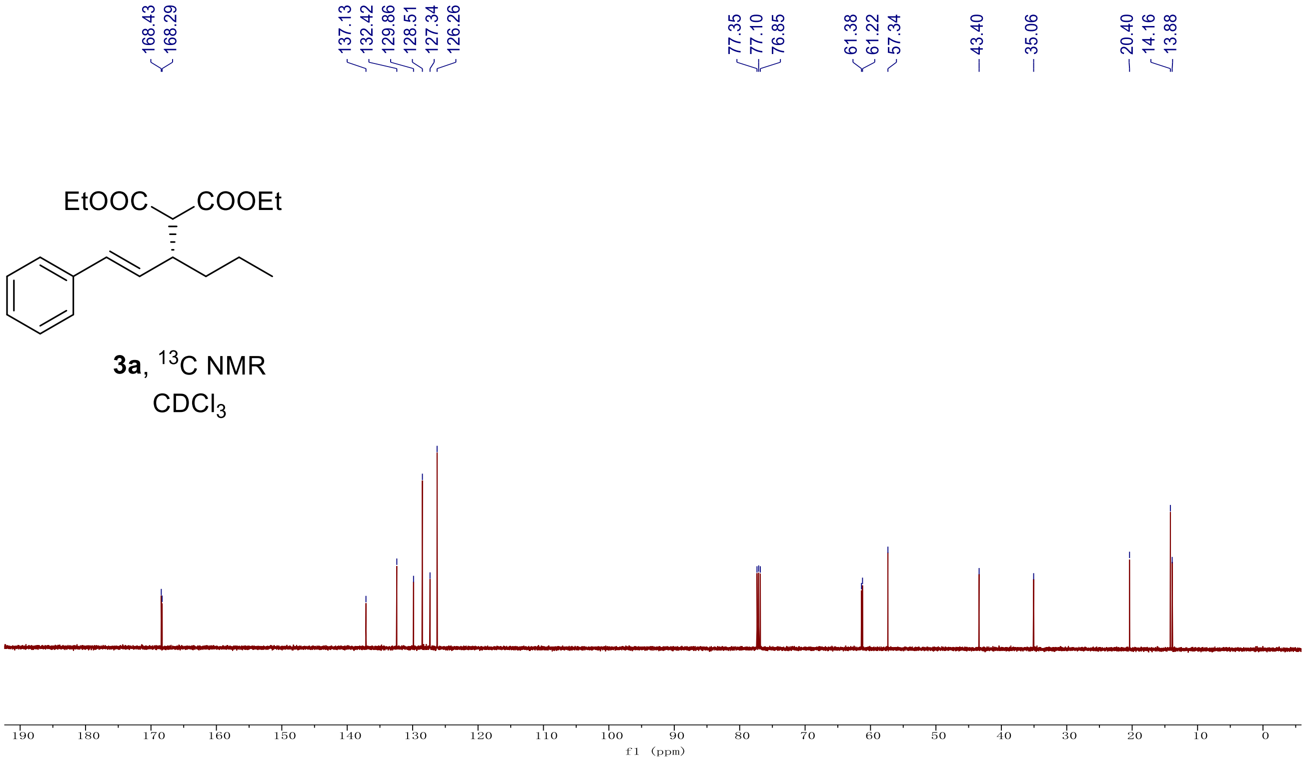

Supplementary Fig. 91. ^{1}H NMR and ^{13}C NMR spectra of compound **3a**.

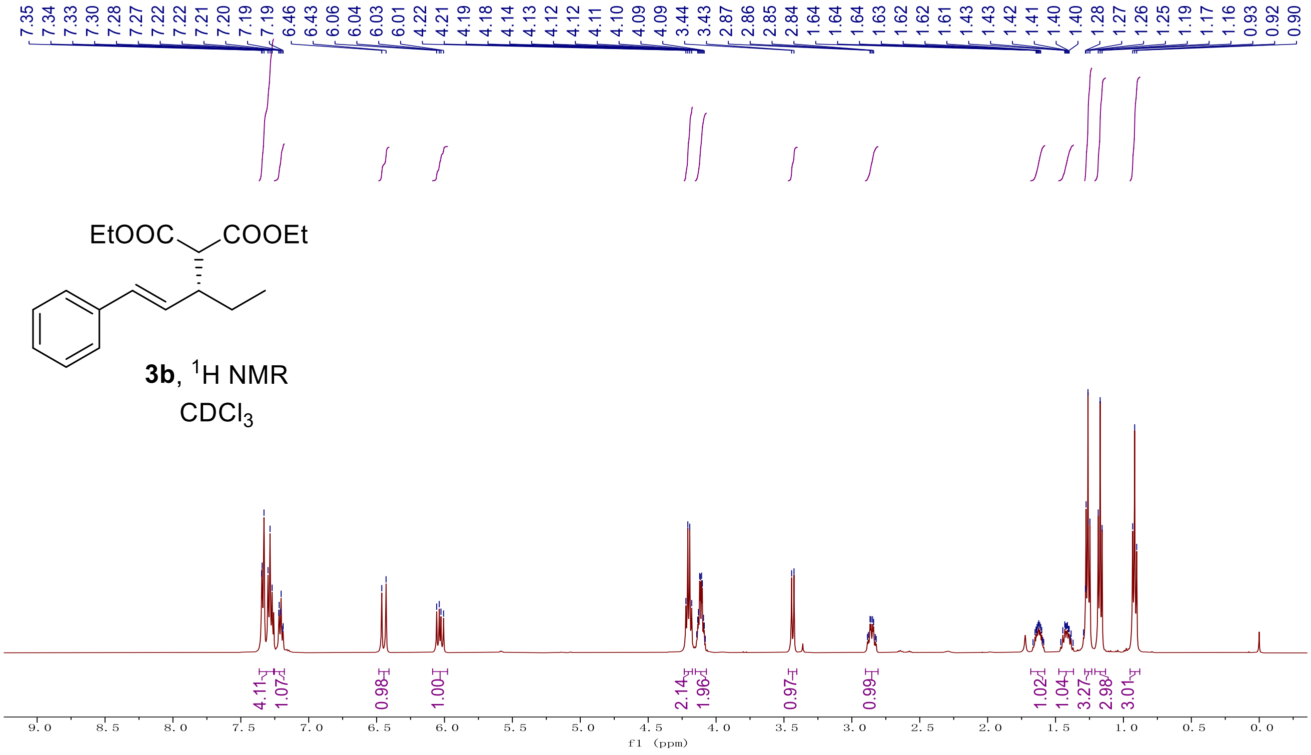

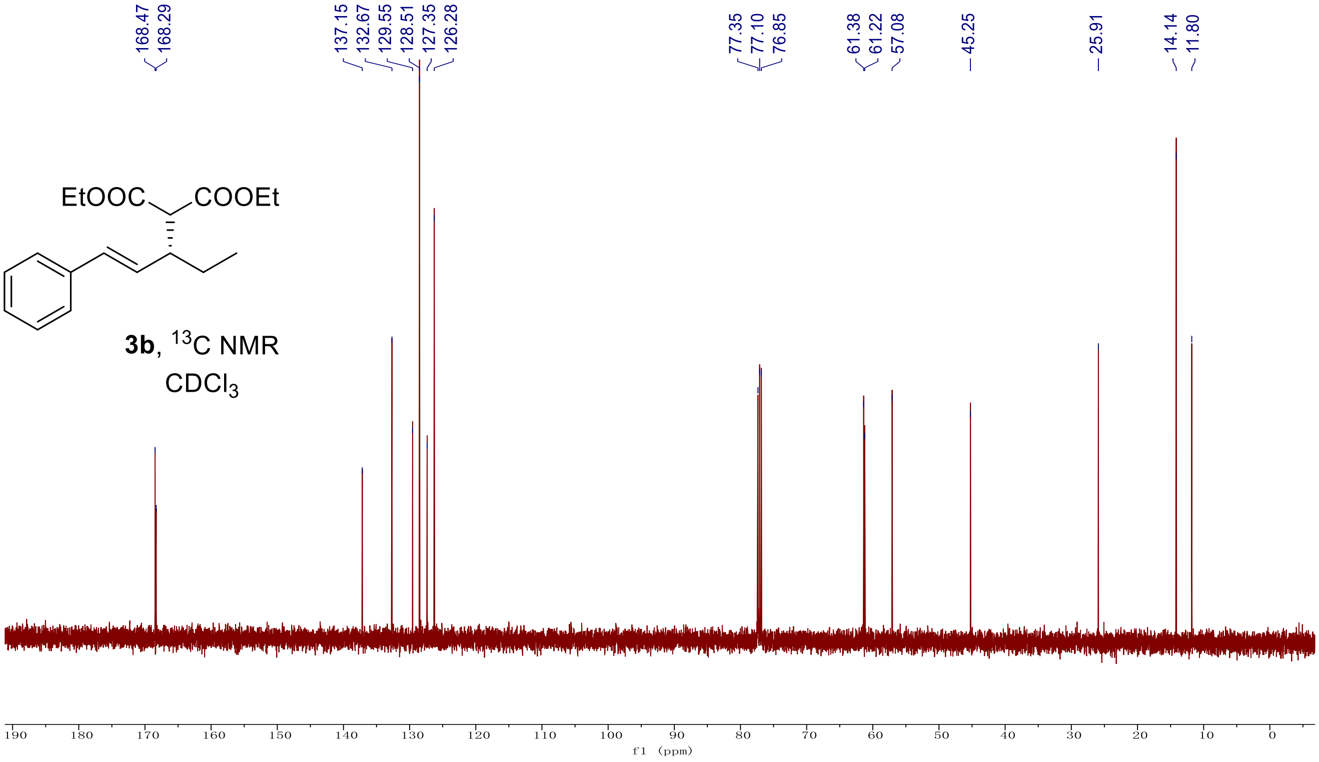

Supplementary Fig. 92. ^{1}H NMR and ^{13}C NMR spectra of compound **3b**.

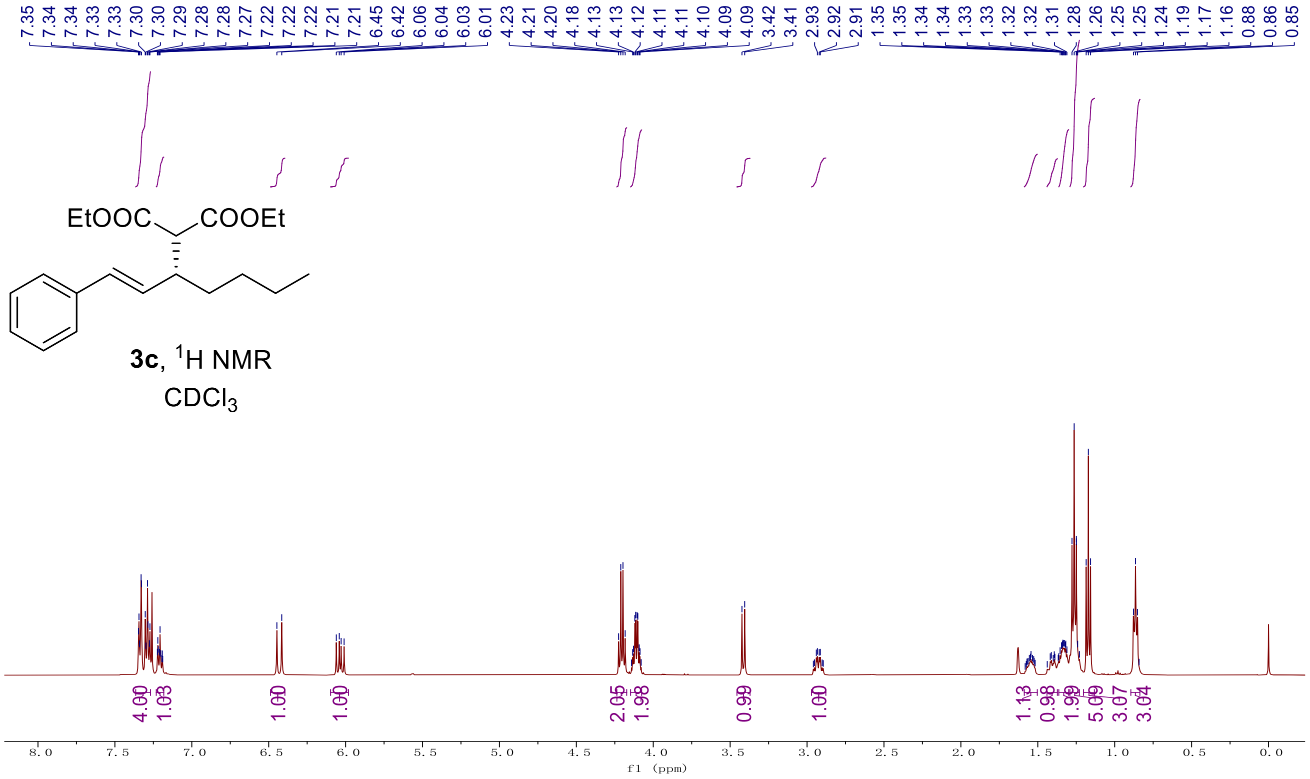

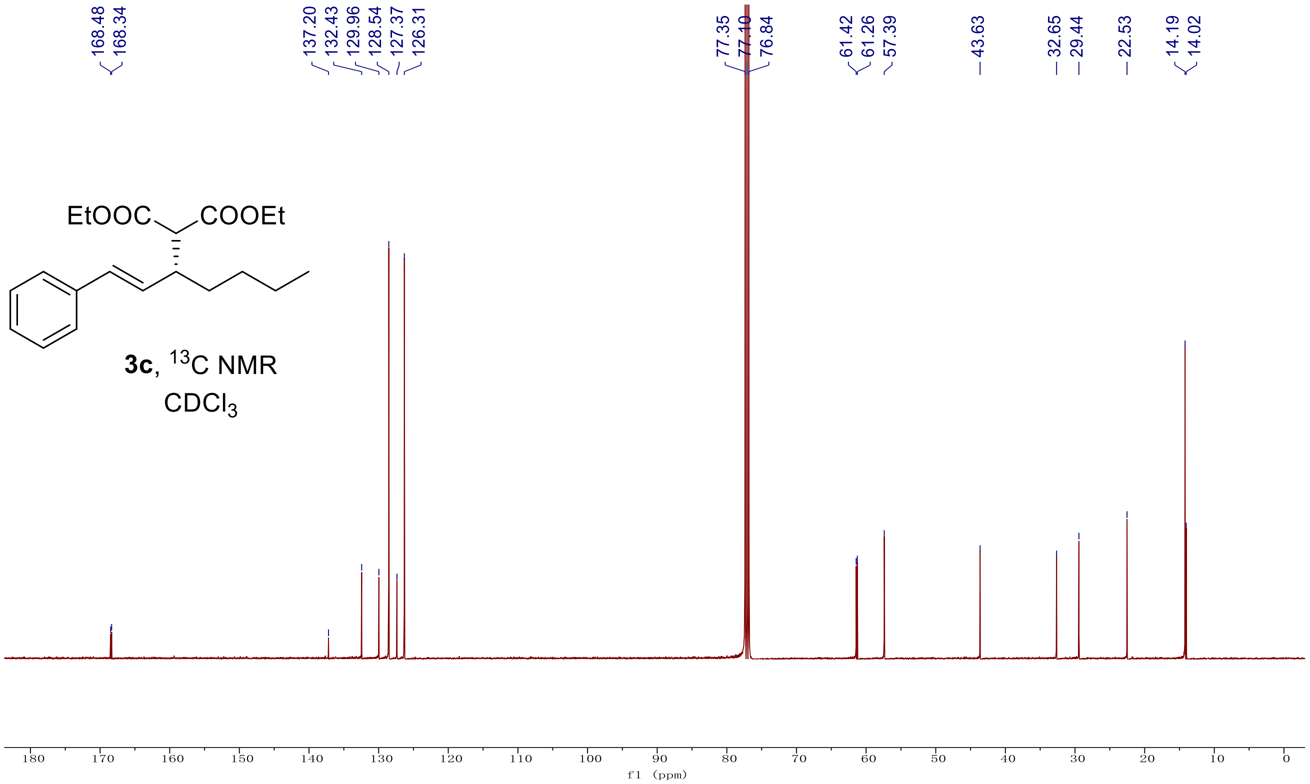

Supplementary Fig. 93. ^{1}H NMR and ^{13}C NMR spectra of compound **3c**.

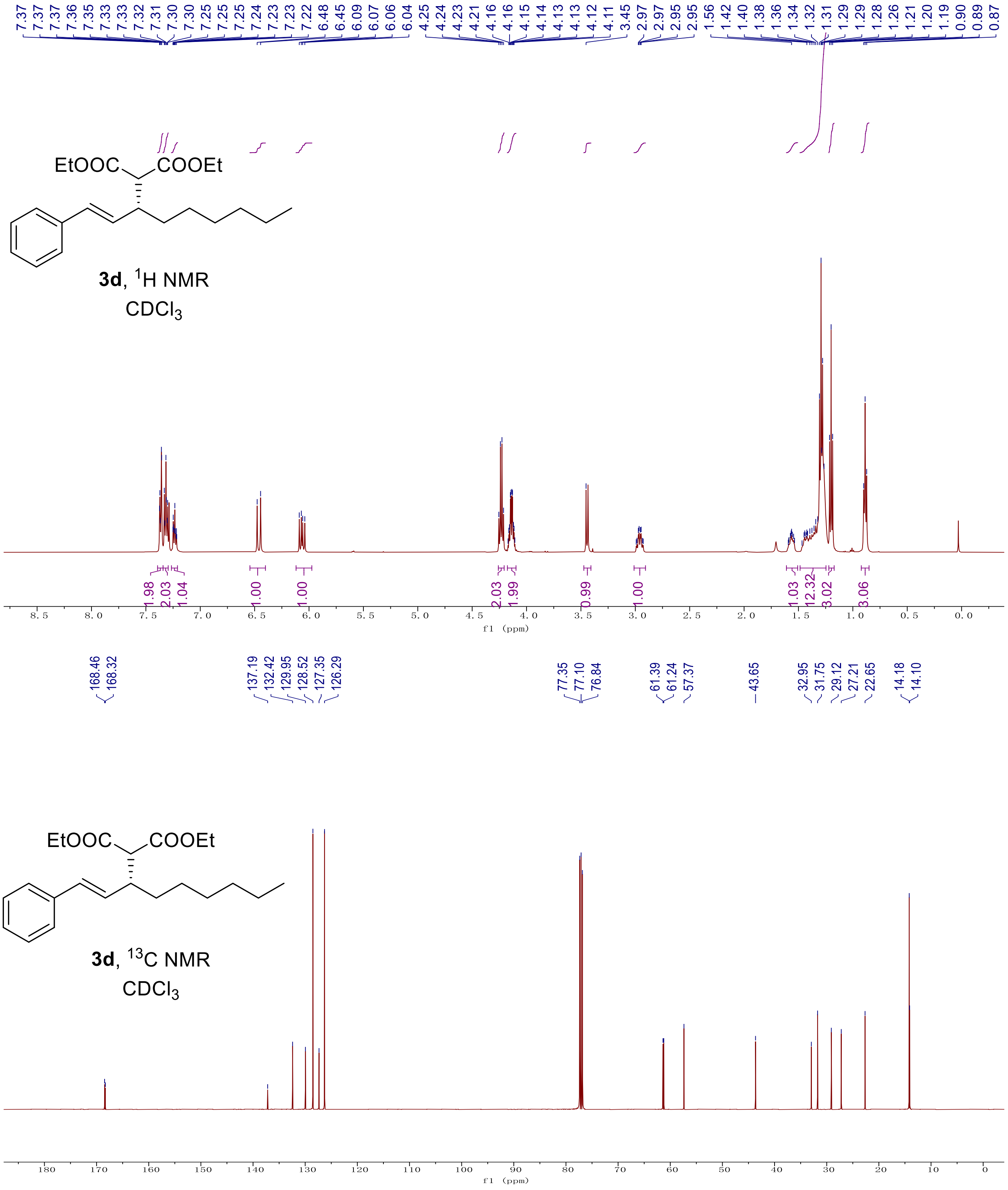

Supplementary Fig. 94. ^{1}H NMR and ^{13}C NMR spectra of compound **3d**.

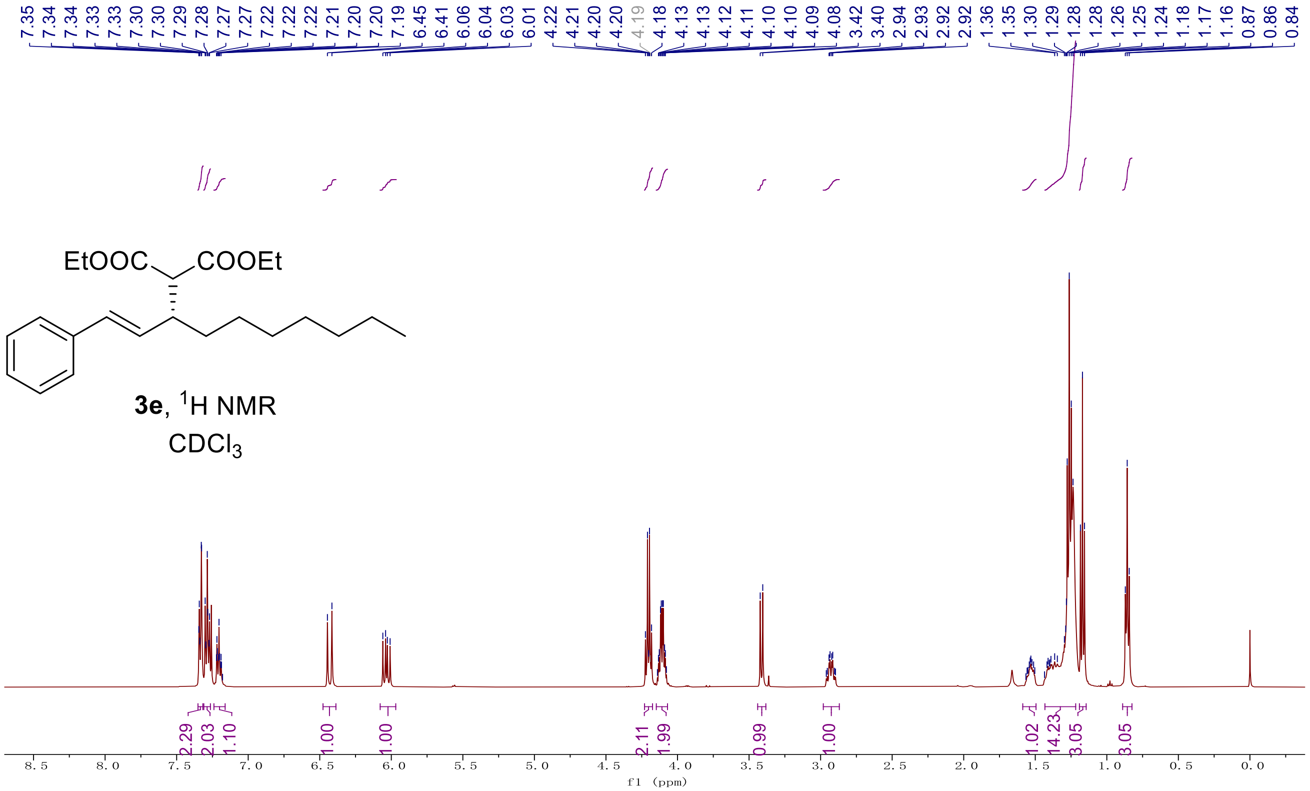

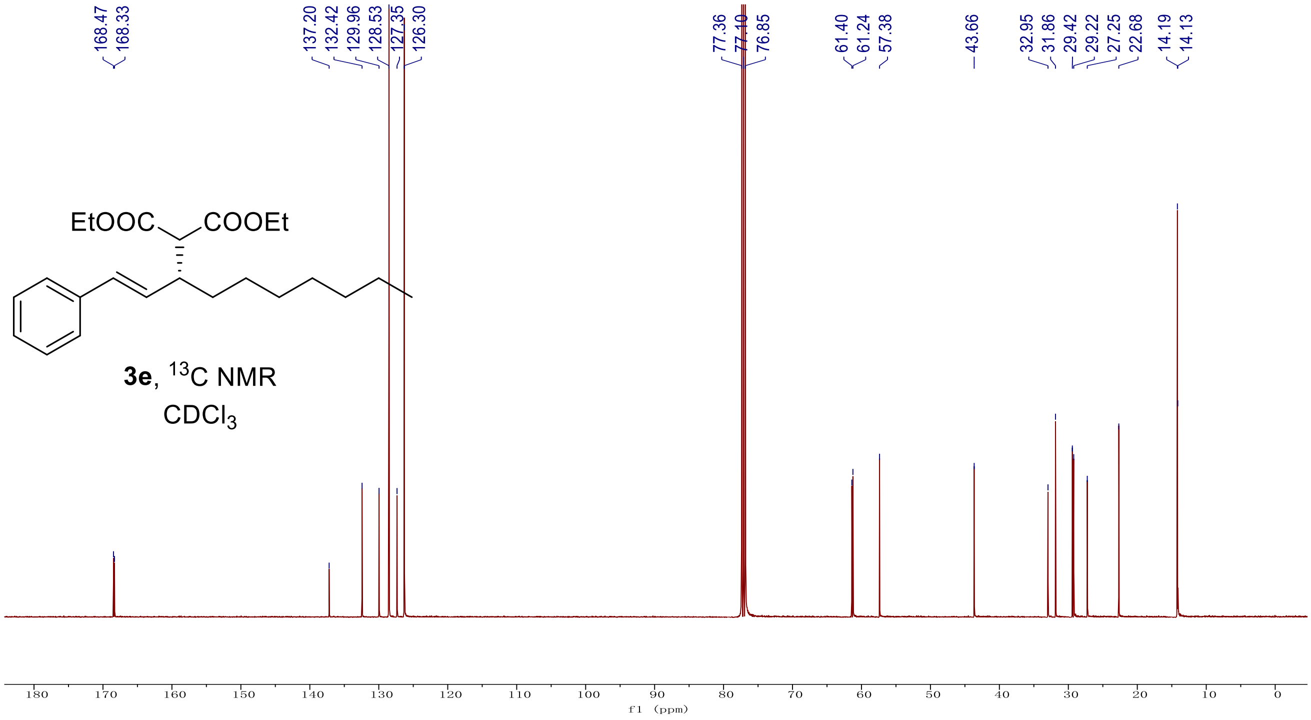

Supplementary Fig. 95. ^{1}H NMR and ^{13}C NMR spectra of compound **3e**.

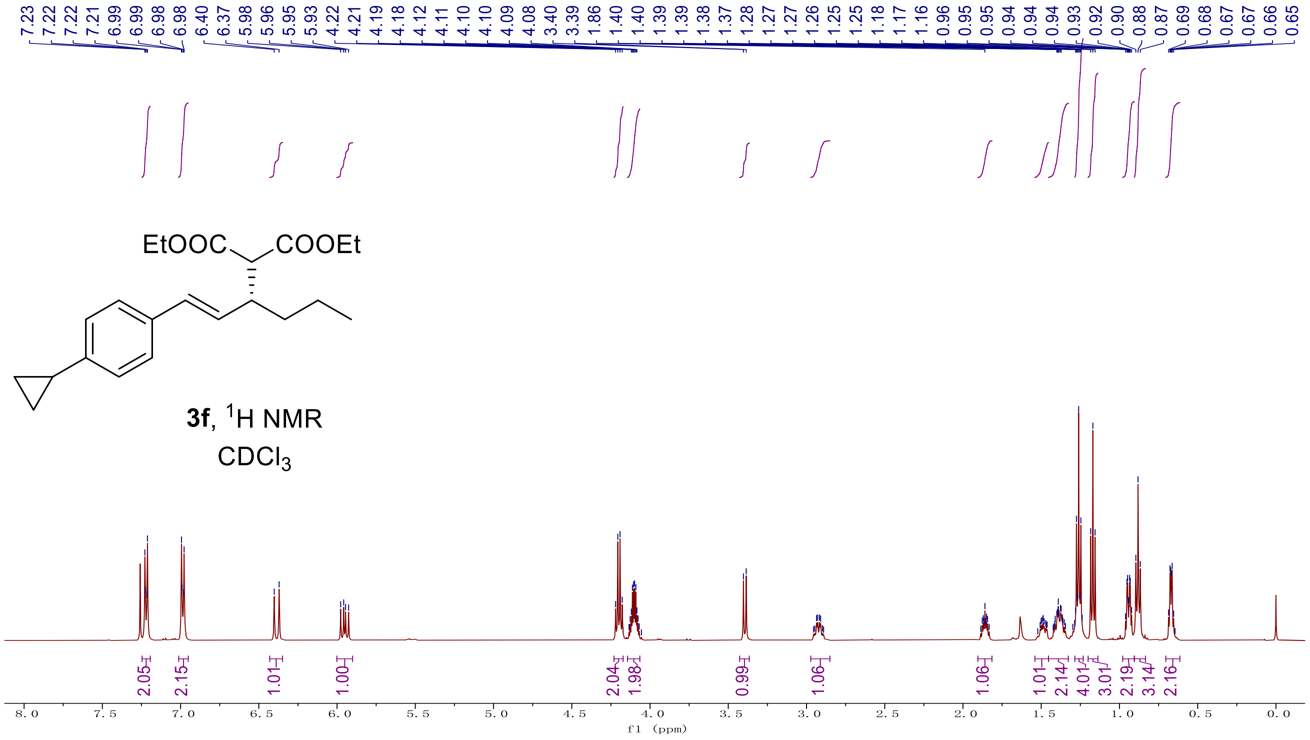

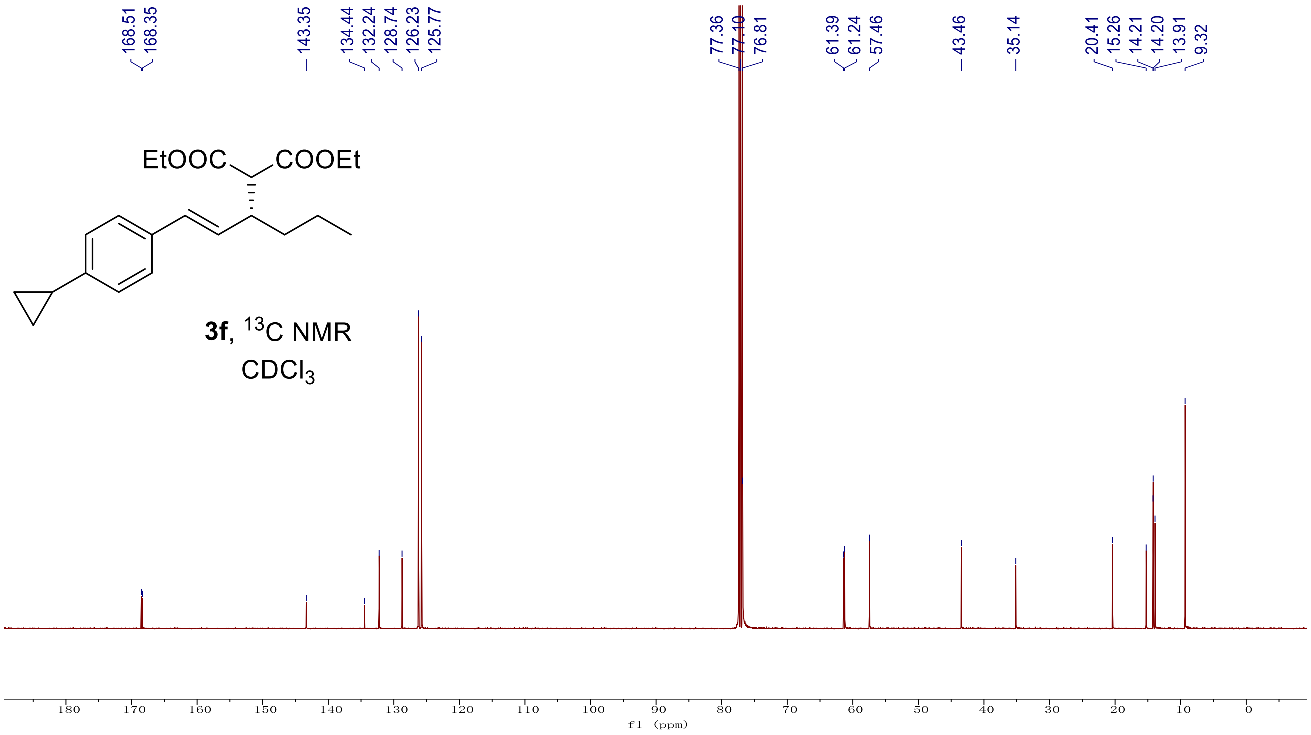

Supplementary Fig. 96. ^{1}H NMR and ^{13}C NMR spectra of compound **3f**.

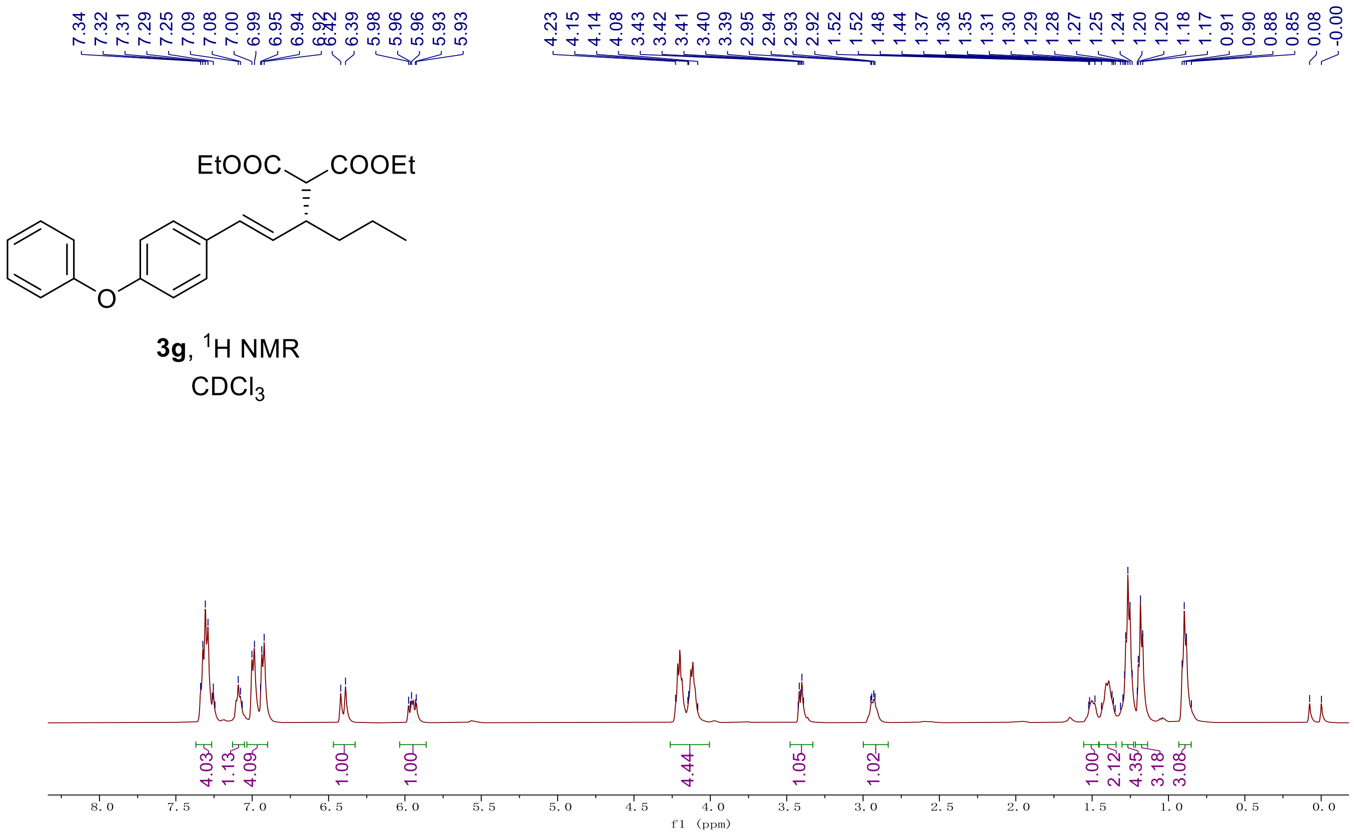

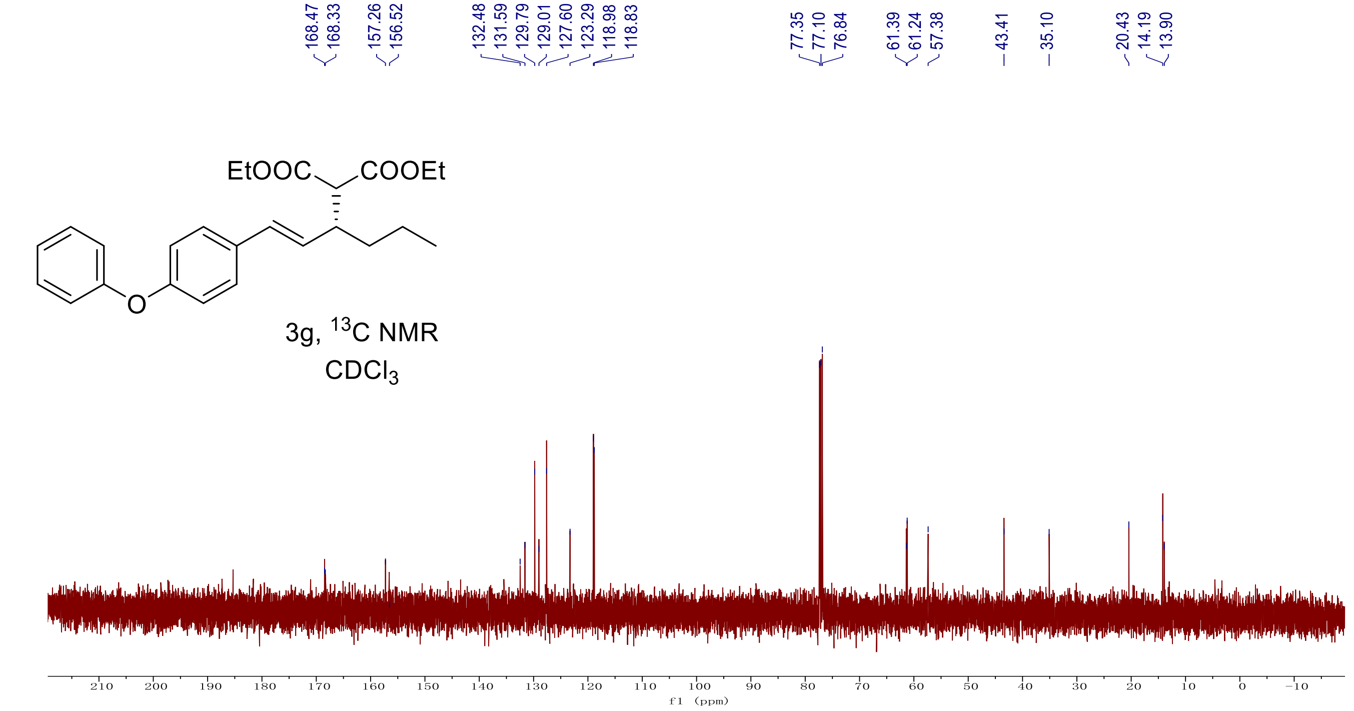

Supplementary Fig. 97. ^{1}H NMR and ^{13}C NMR spectra of compound **3g**.

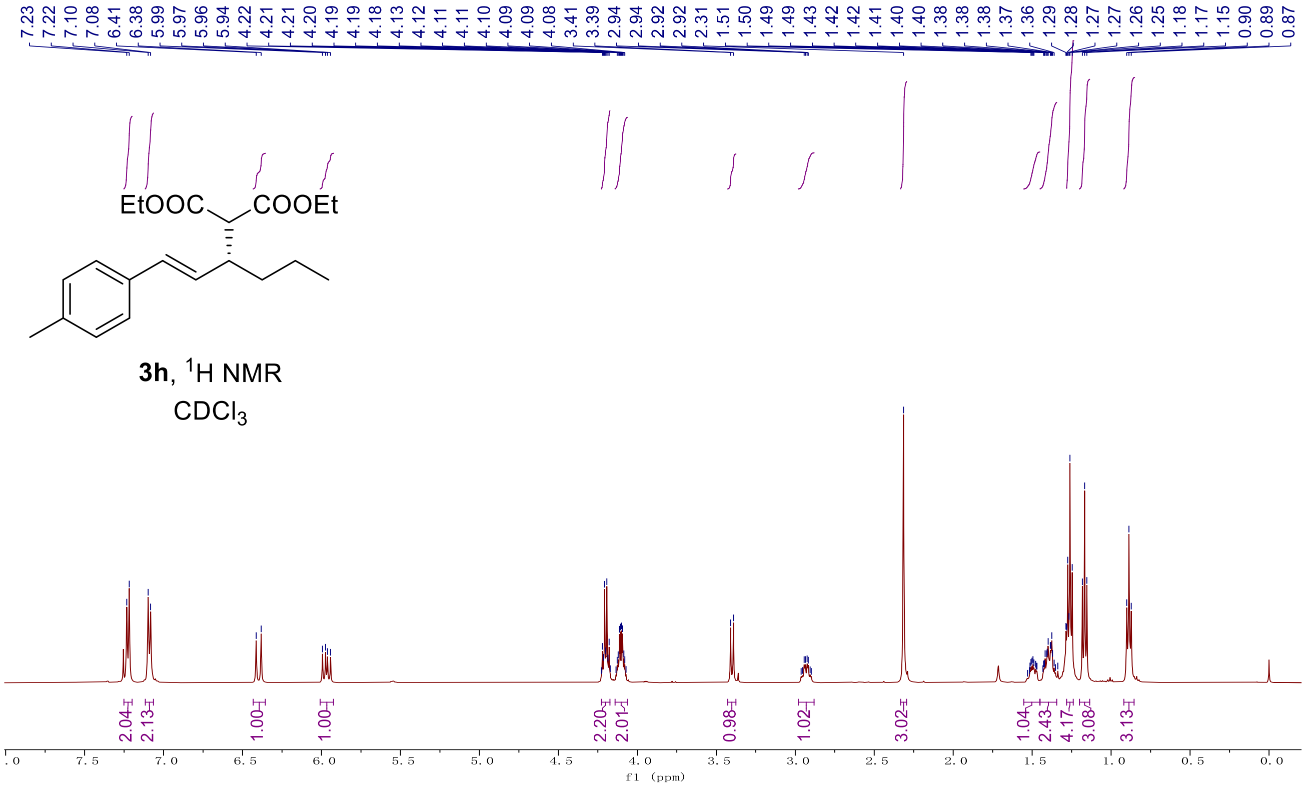

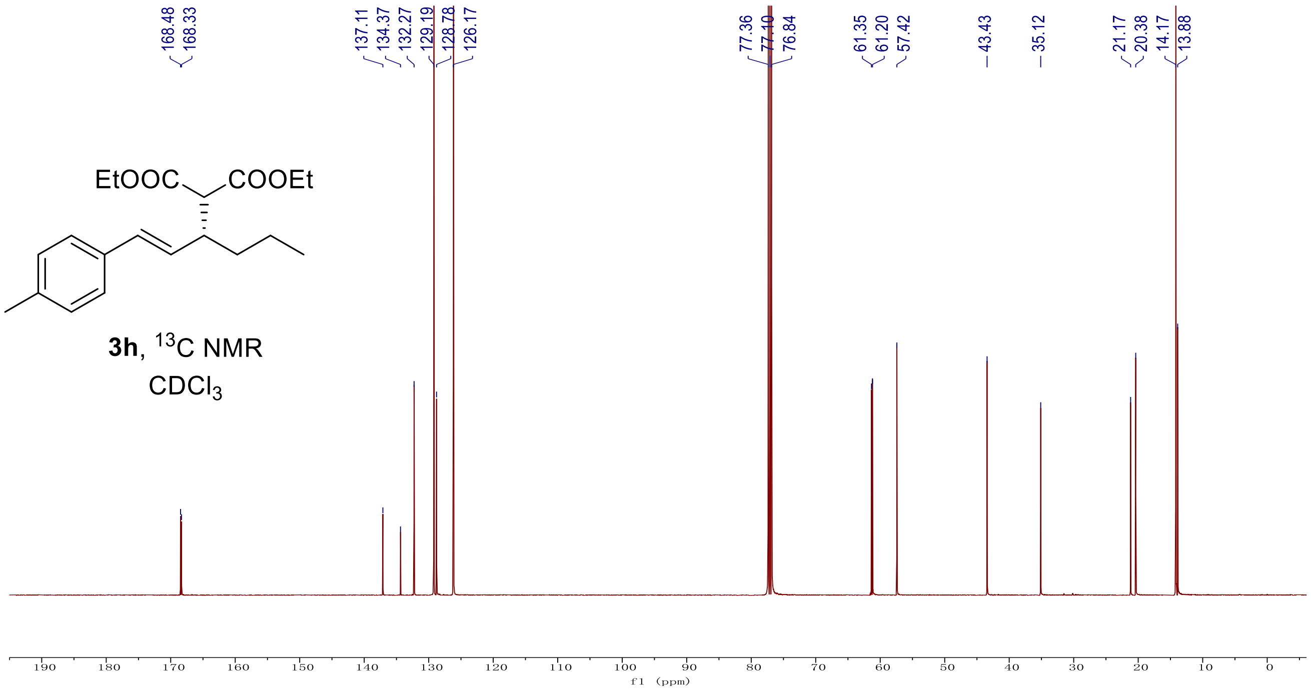

Supplementary Fig. 98. ^{1}H NMR and ^{13}C NMR spectra of compound **3h**.

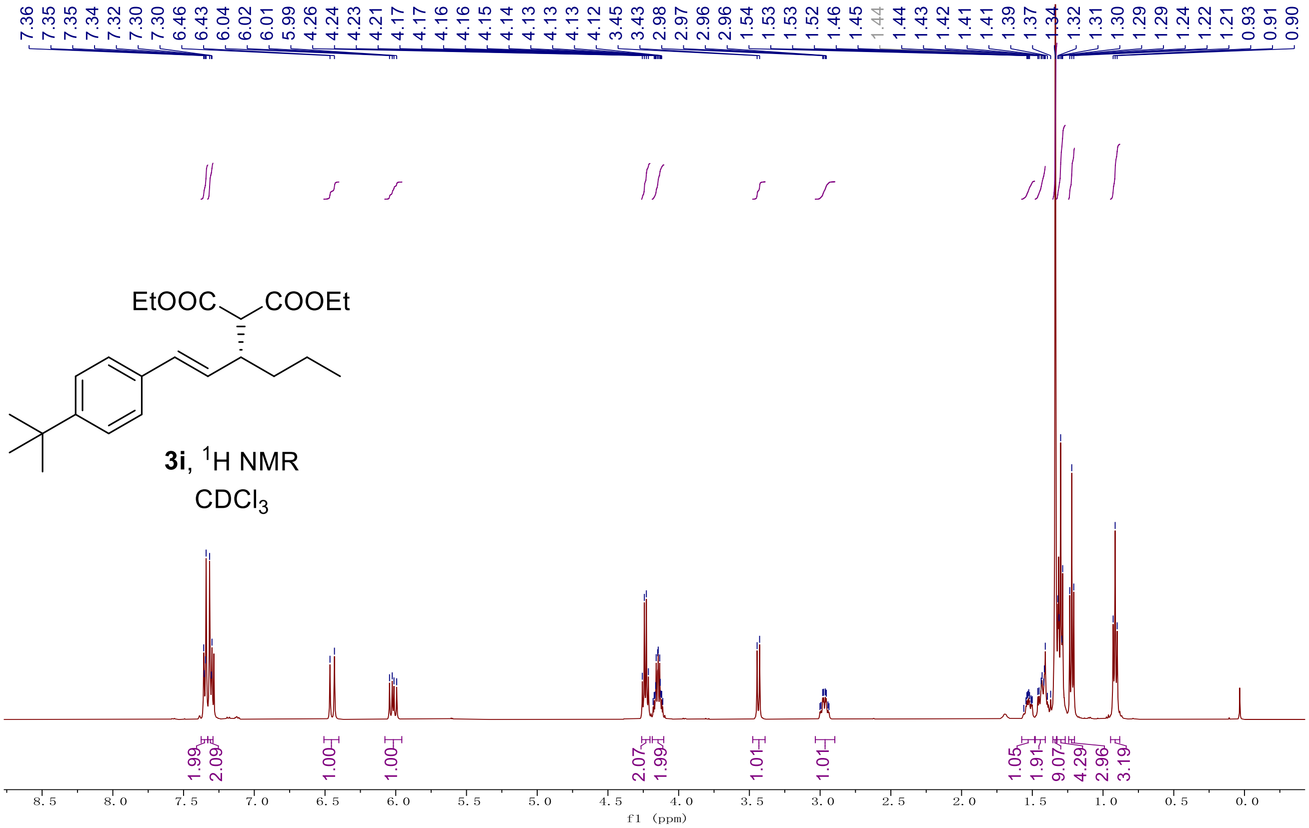

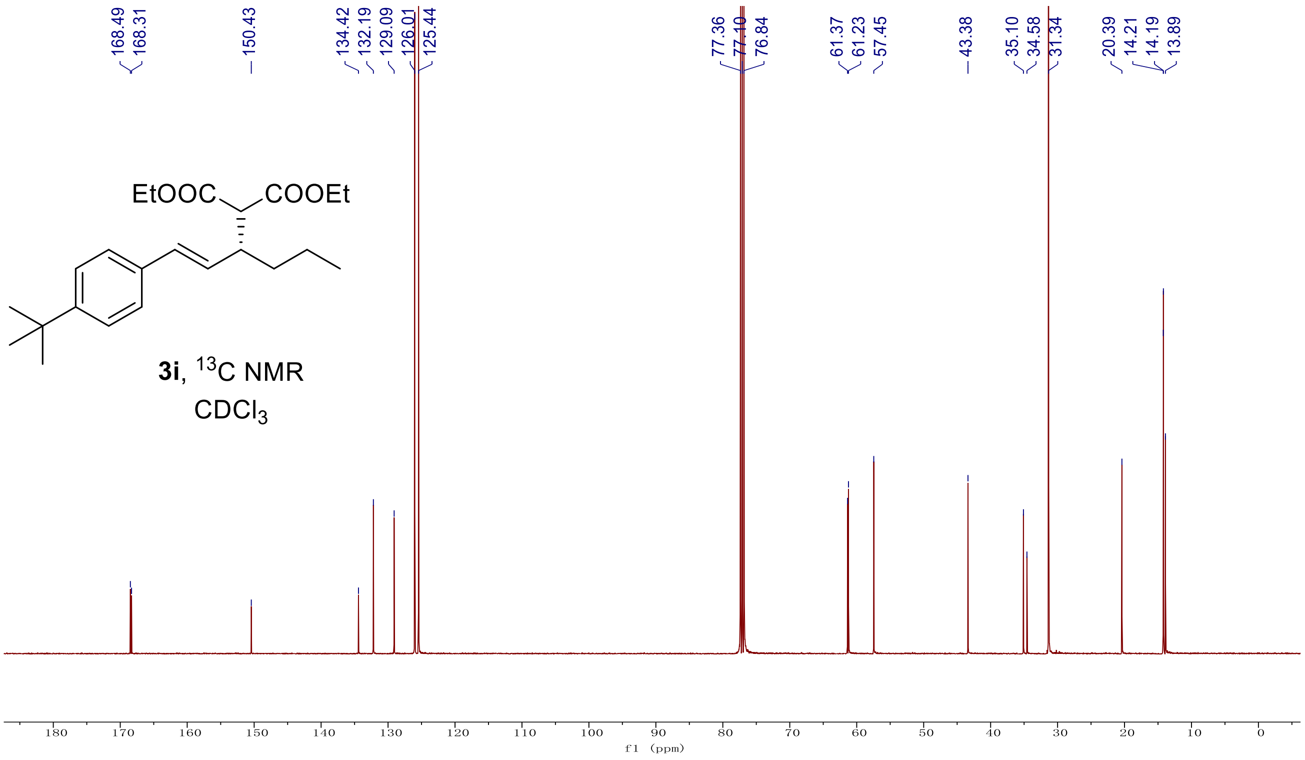

Supplementary Fig. 99. ^{1}H NMR and ^{13}C NMR spectra of compound **3i**.

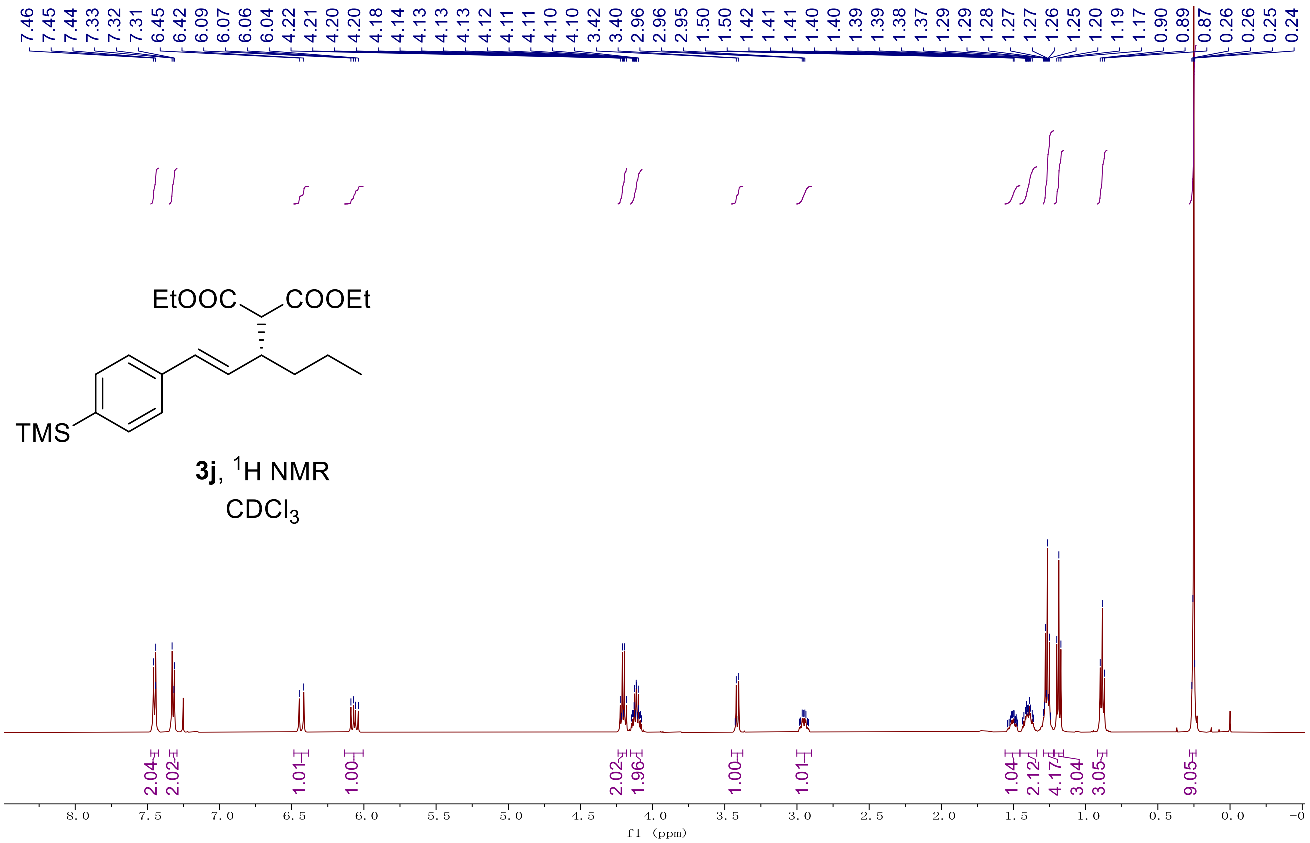

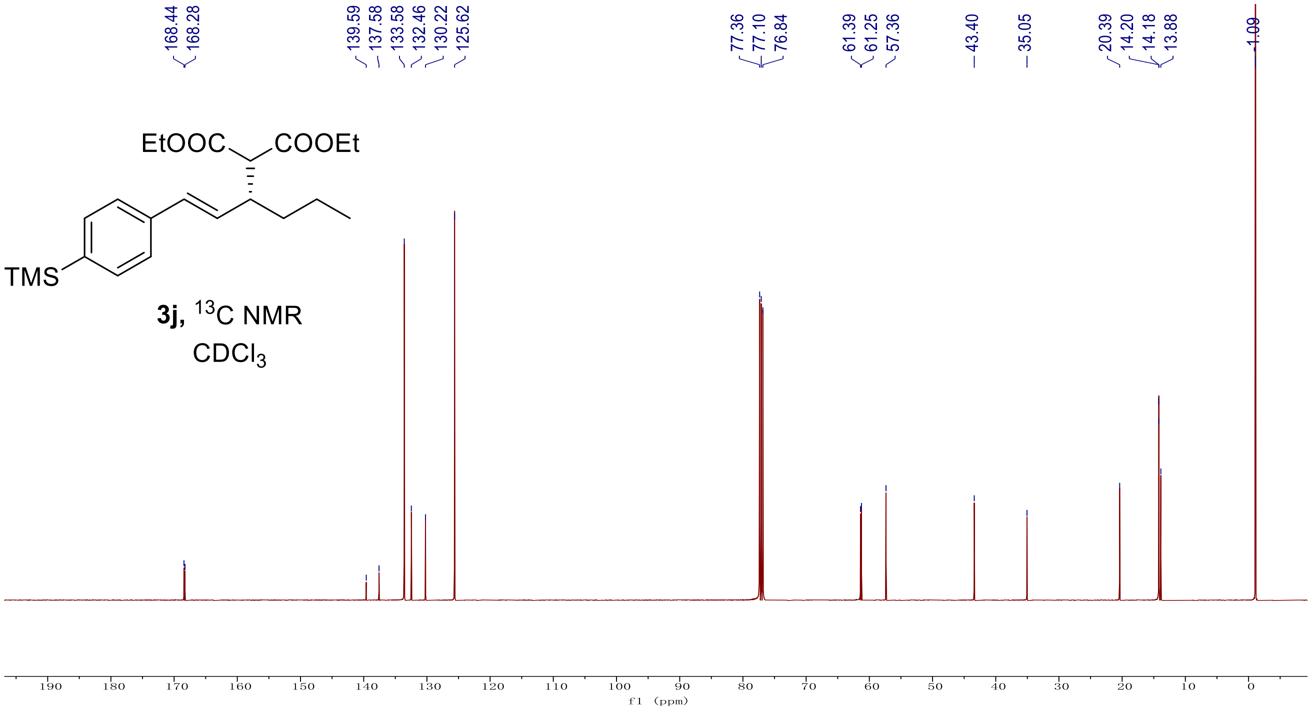

Supplementary Fig. 100. ^{1}H NMR and ^{13}C NMR spectra of compound **3j**.

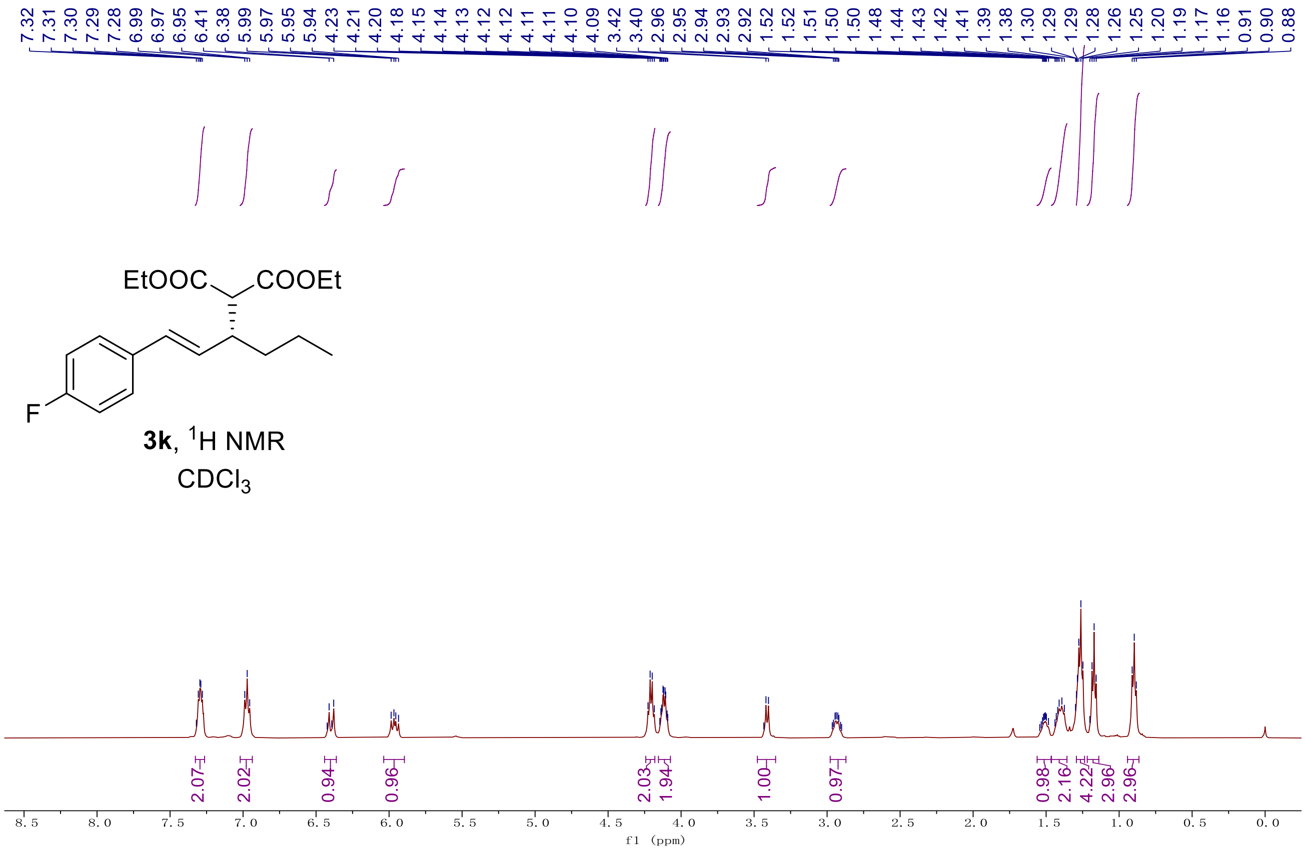

3k, ^{1}H NMR
$CDCl_3$

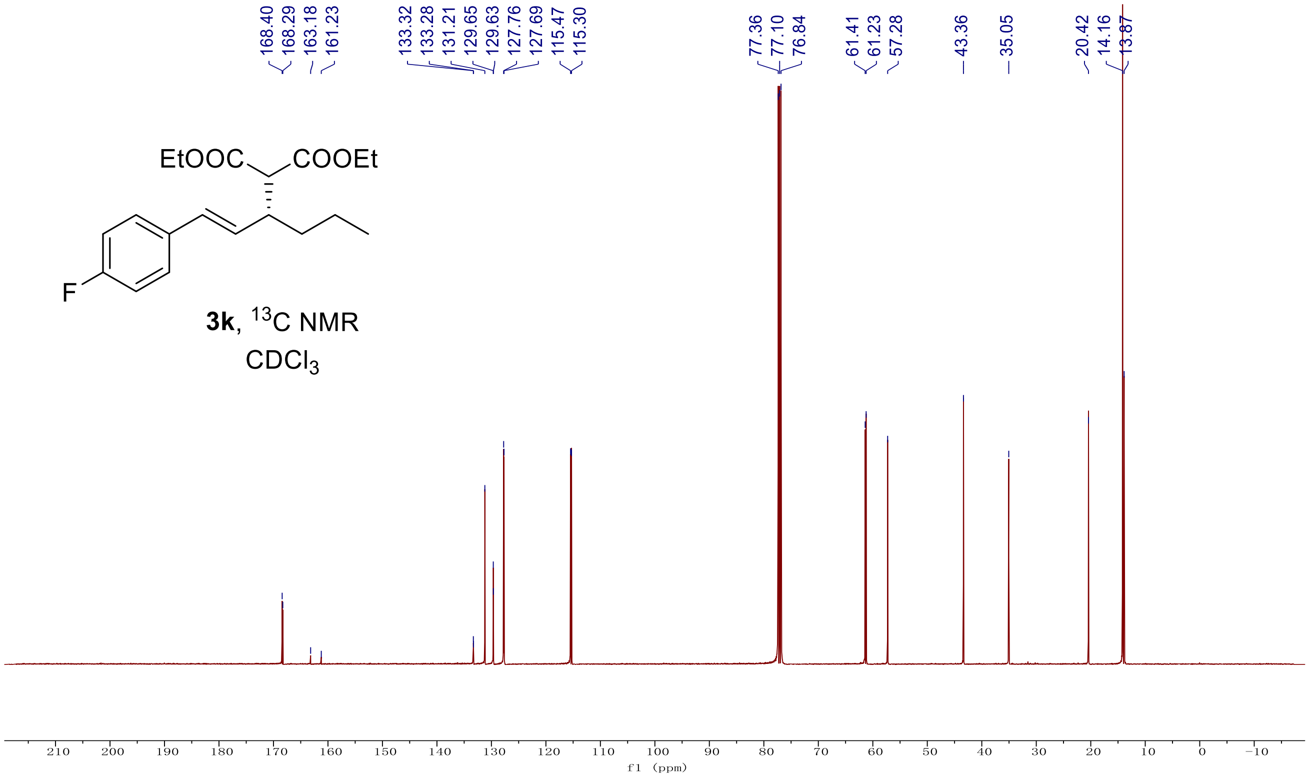

3k, ^{13}C NMR
$CDCl_3$

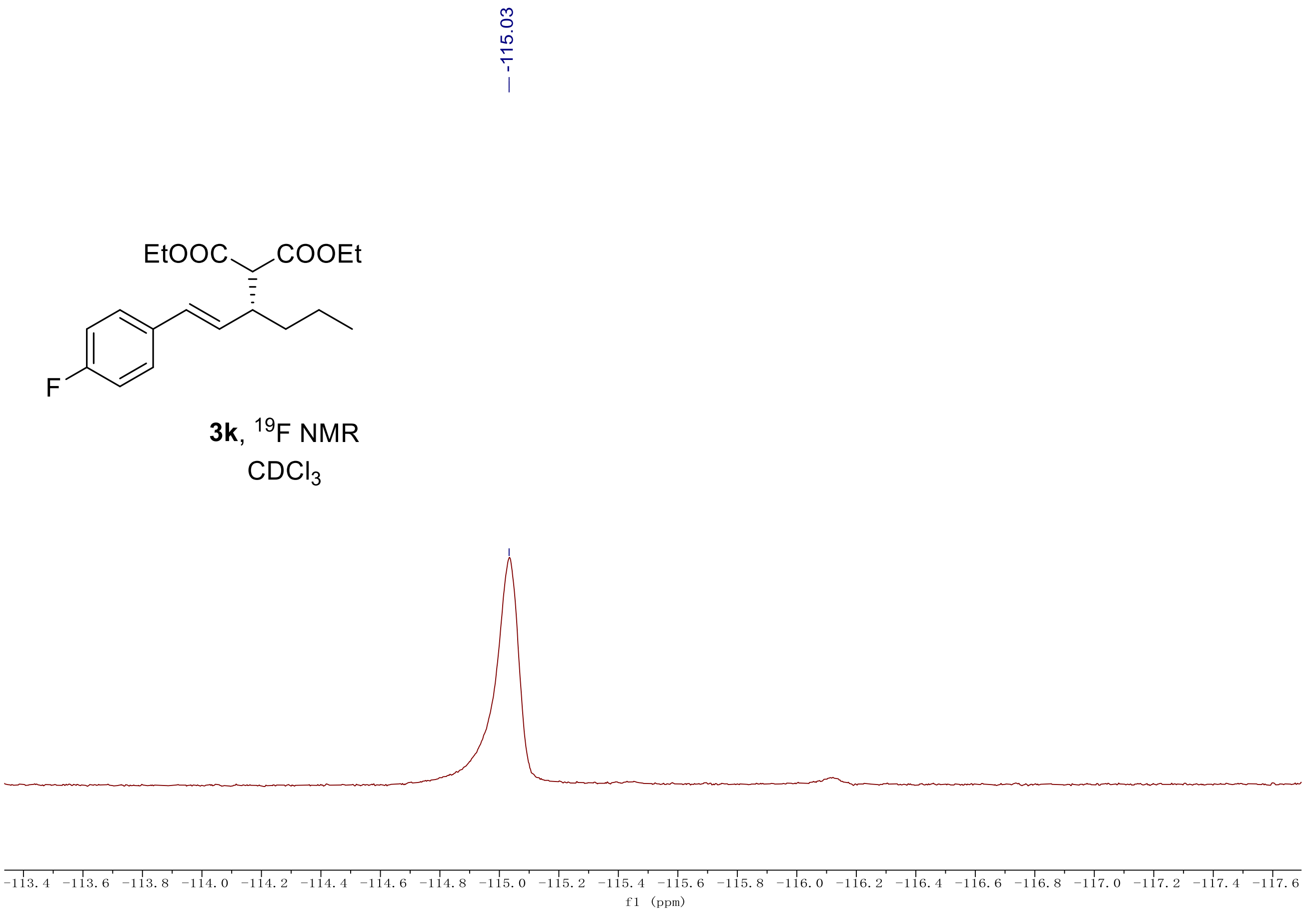

Supplementary Fig. 101. ^{1}H NMR ^{13}C NMR and ^{19}F NMR spectra of compound **3k**.

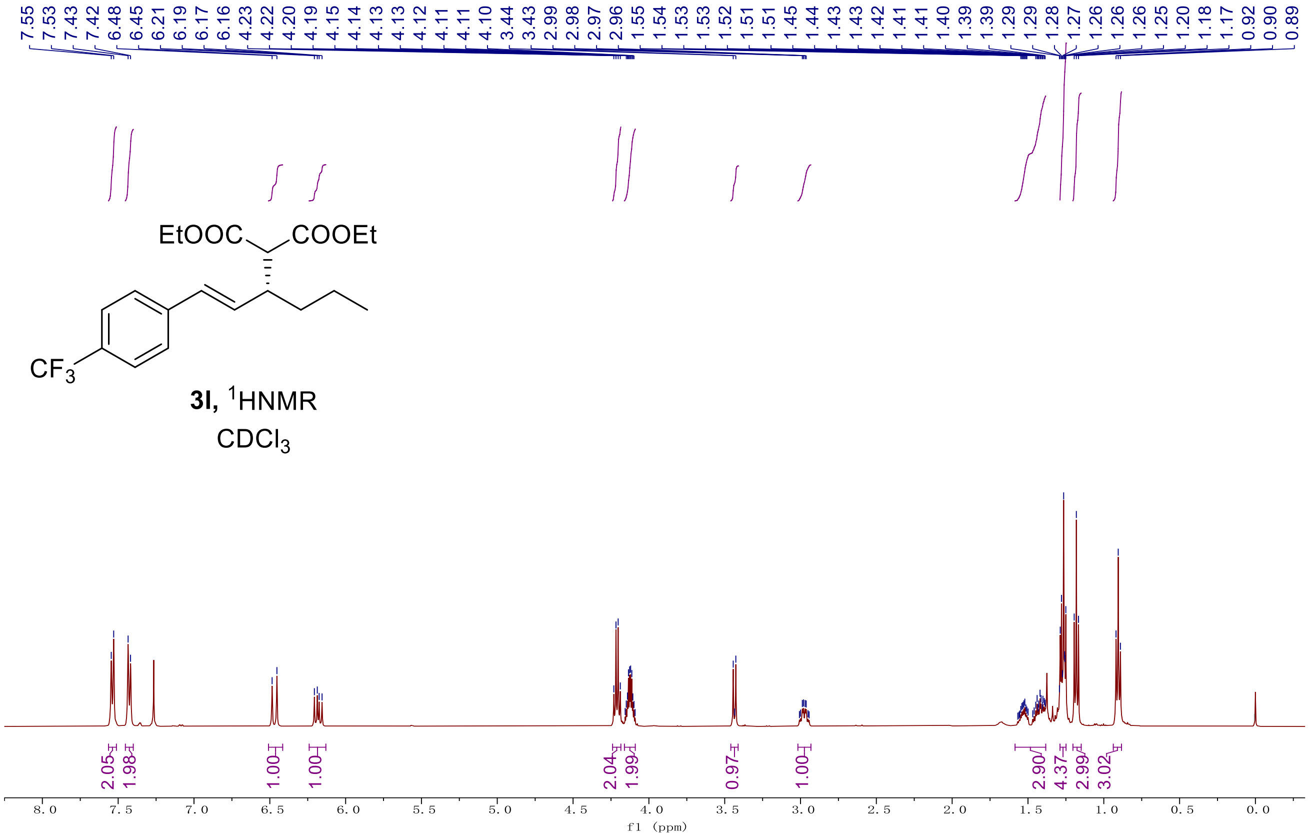
EtOOC
COOEt
CF3
3l, 1HNMR
CDCl3
f1 (ppm)

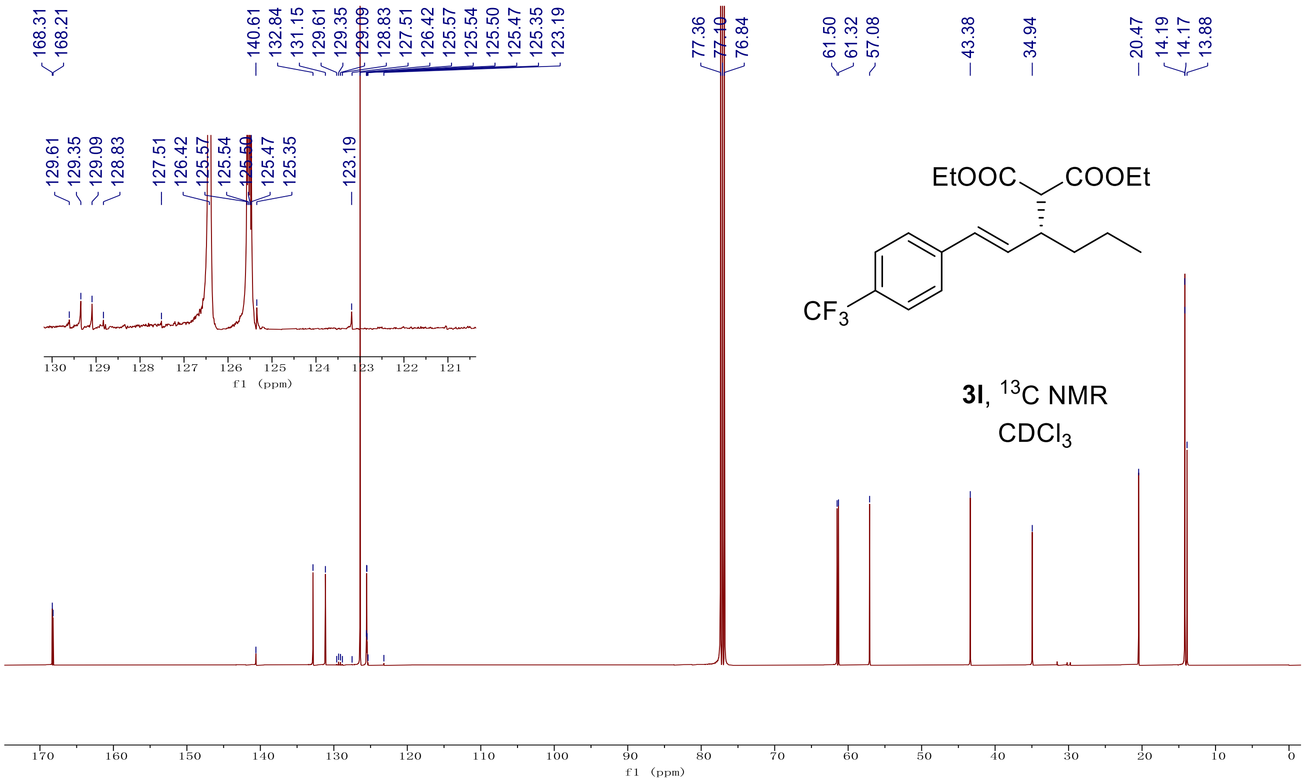
EtOOC
COOEt
CF3
3l, 13C NMR
CDCl3
f1 (ppm)

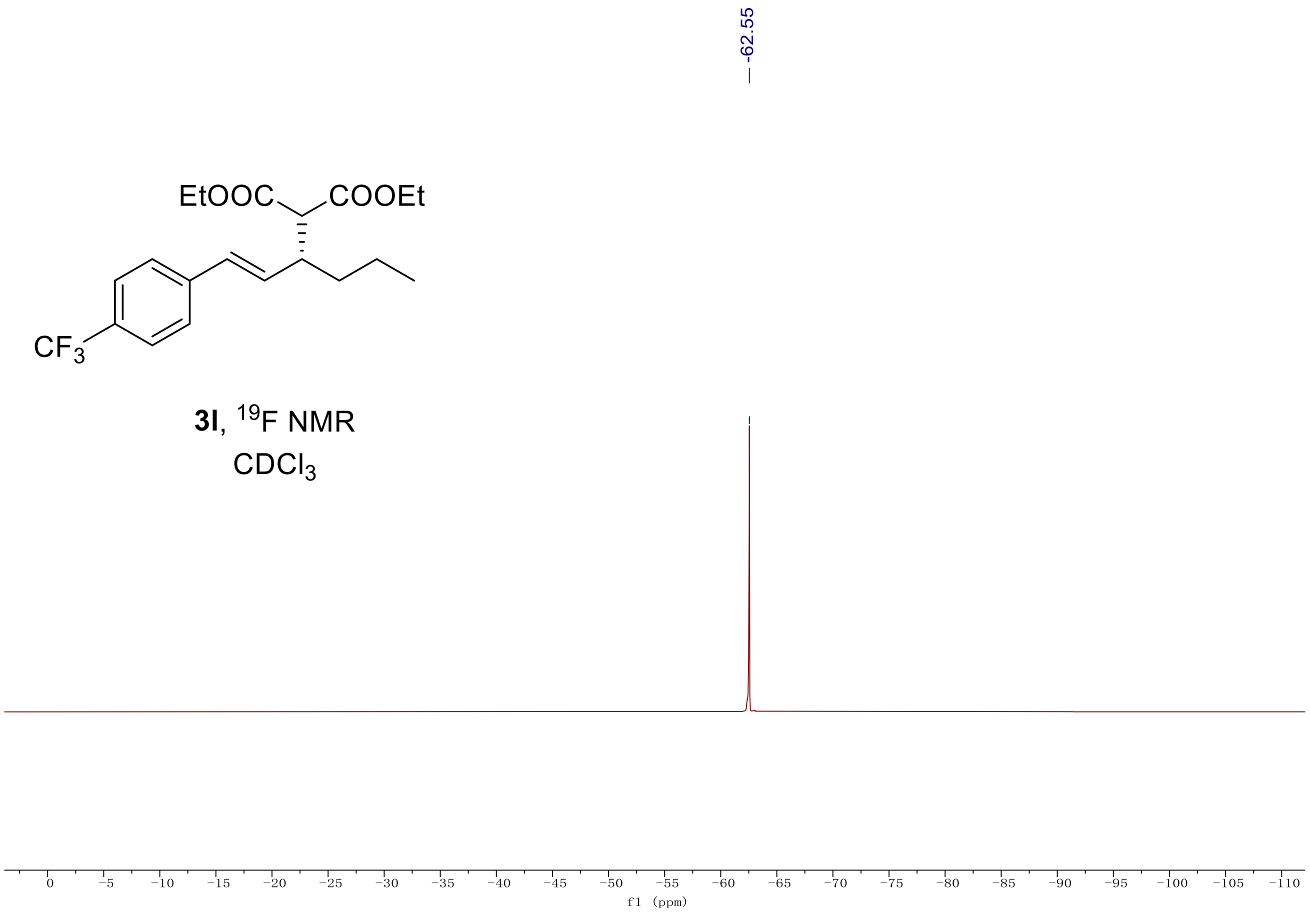

Supplementary Fig. 102. ^{1}H NMR, ^{13}C NMR and ^{19}F NMR spectra of compound **3l**.

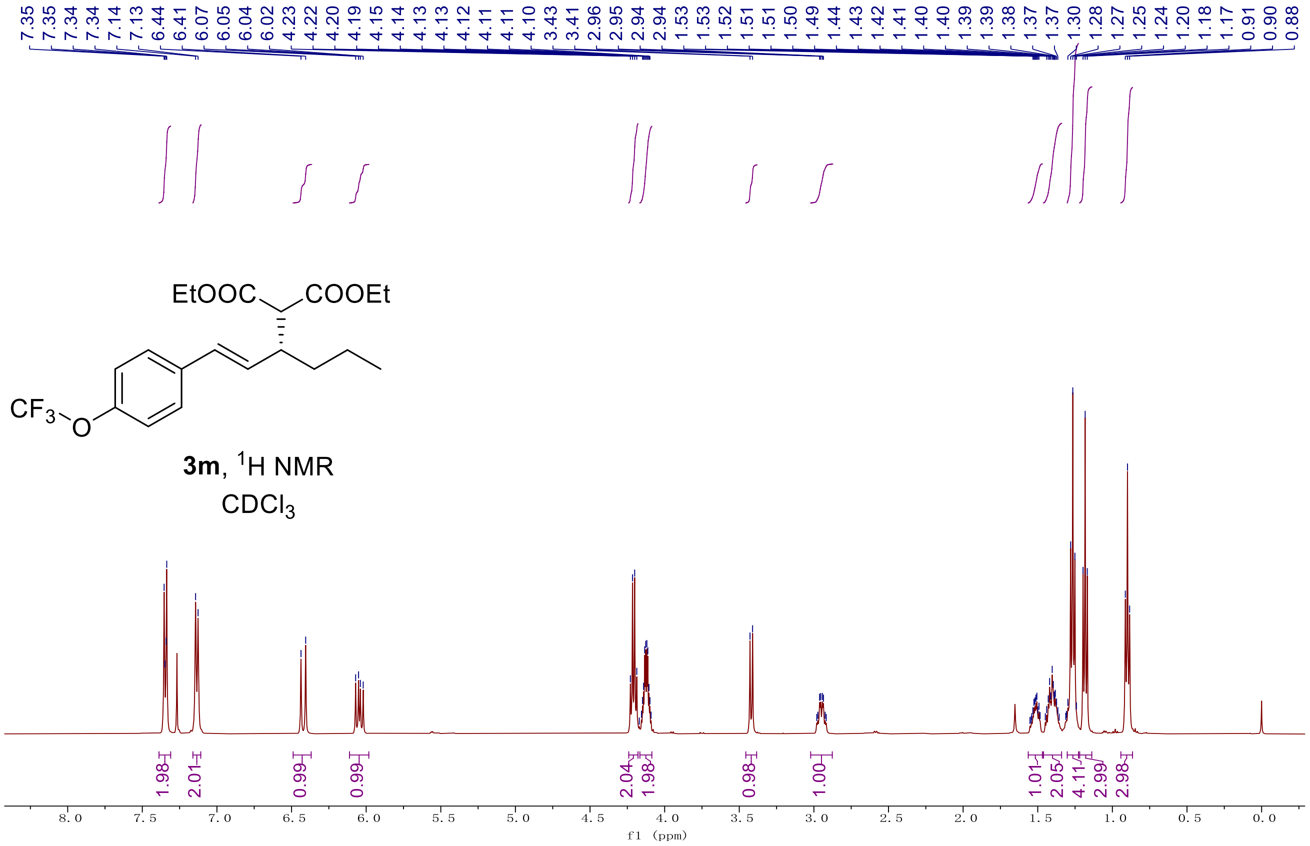
7.35 7.35 7.34 7.34 7.14 7.13 6.44 6.41 6.07 6.05 6.04 6.02 4.23 4.22 4.20 4.19 4.15 4.14 4.13 4.13 4.12 4.11 4.11 4.10 3.43 3.41 2.96 2.95 2.94 2.94 1.53 1.53 1.52 1.51 1.51 1.50 1.49 1.44 1.43 1.42 1.41 1.40 1.39 1.39 1.38 1.37 1.37 1.30 1.28 1.27 1.25 1.24 1.20 1.18 1.17 0.91 0.90 0.88
EtOOC
COOEt
CF3
O
3m, 1H NMR
CDCl3
1.98 2.01 0.99 0.99 2.04 1.98 0.98 1.00 1.01 2.05 4.11 2.99 2.98
8.0 7.5 7.0 6.5 6.0 5.5 5.0 4.5 4.0 3.5 3.0 2.5 2.0 1.5 1.0 0.5 0.0
f1 (ppm)

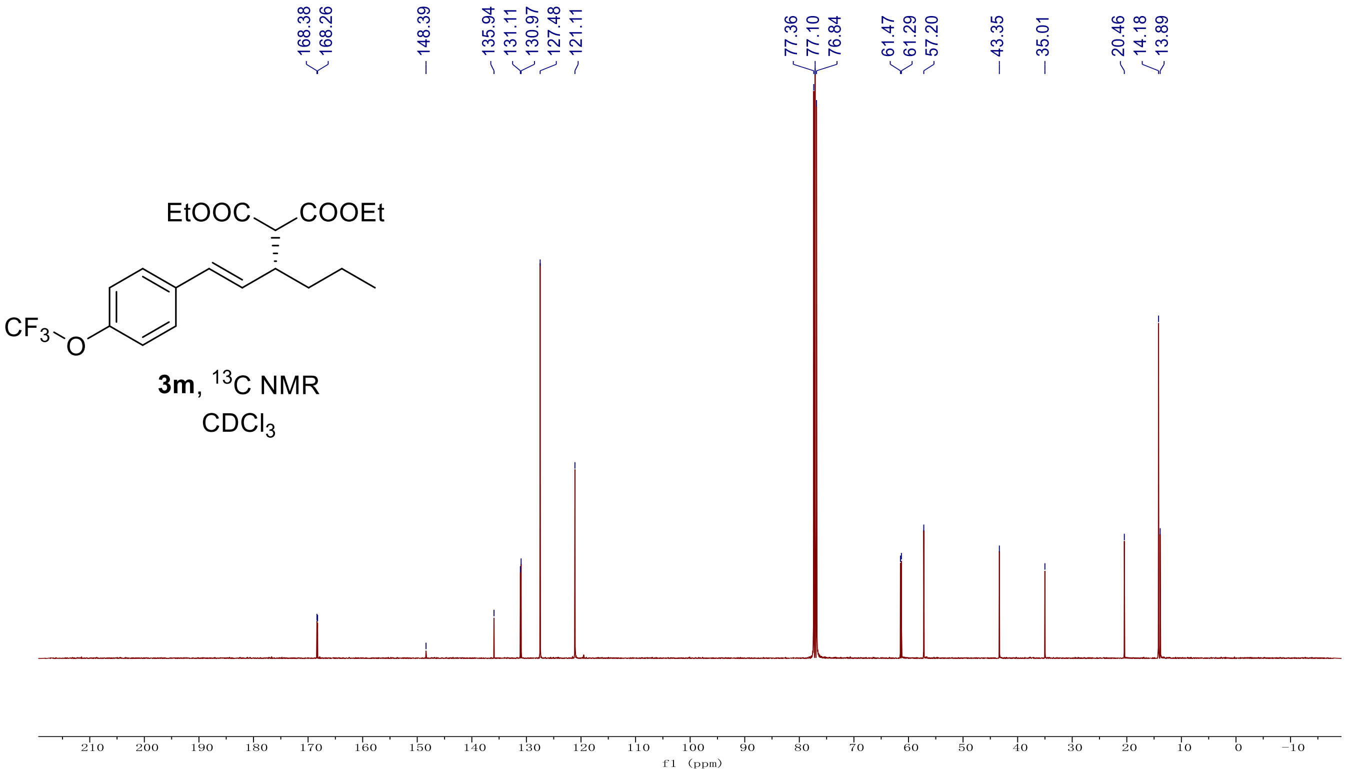
168.38 168.26 148.39 135.94 131.11 130.97 127.48 121.11 77.36 77.10 76.84 61.47 61.29 57.20 43.35 35.01 20.46 14.18 13.89
EtOOC
COOEt
CF3
O
3m, 13C NMR
CDCl3
210 200 190 180 170 160 150 140 130 120 110 100 90 80 70 60 50 40 30 20 10 0 -10
f1 (ppm)

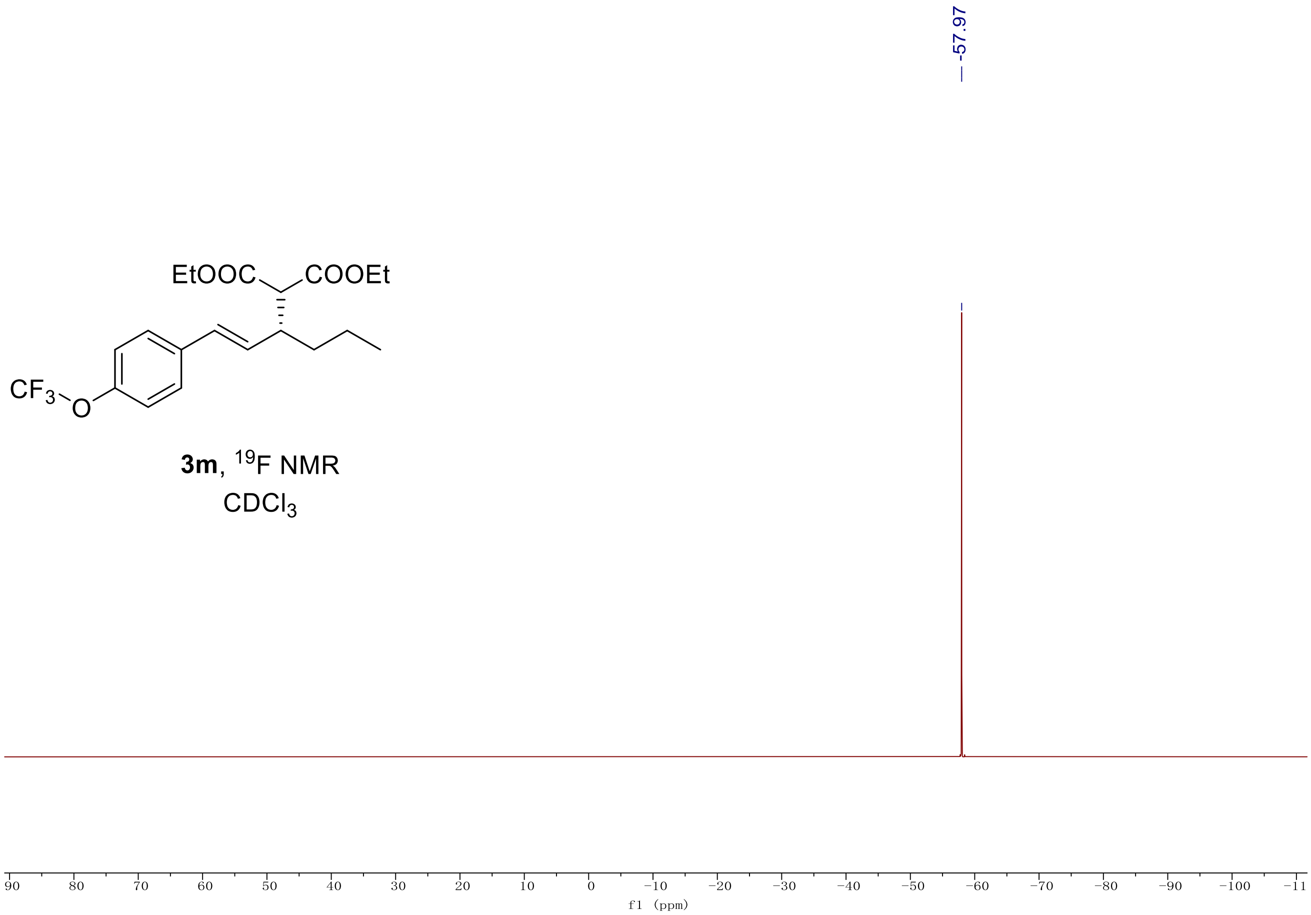

Supplementary Fig. 103. ^{1}H NMR, ^{13}C NMR and ^{19}F NMR spectra of compound **3m**.

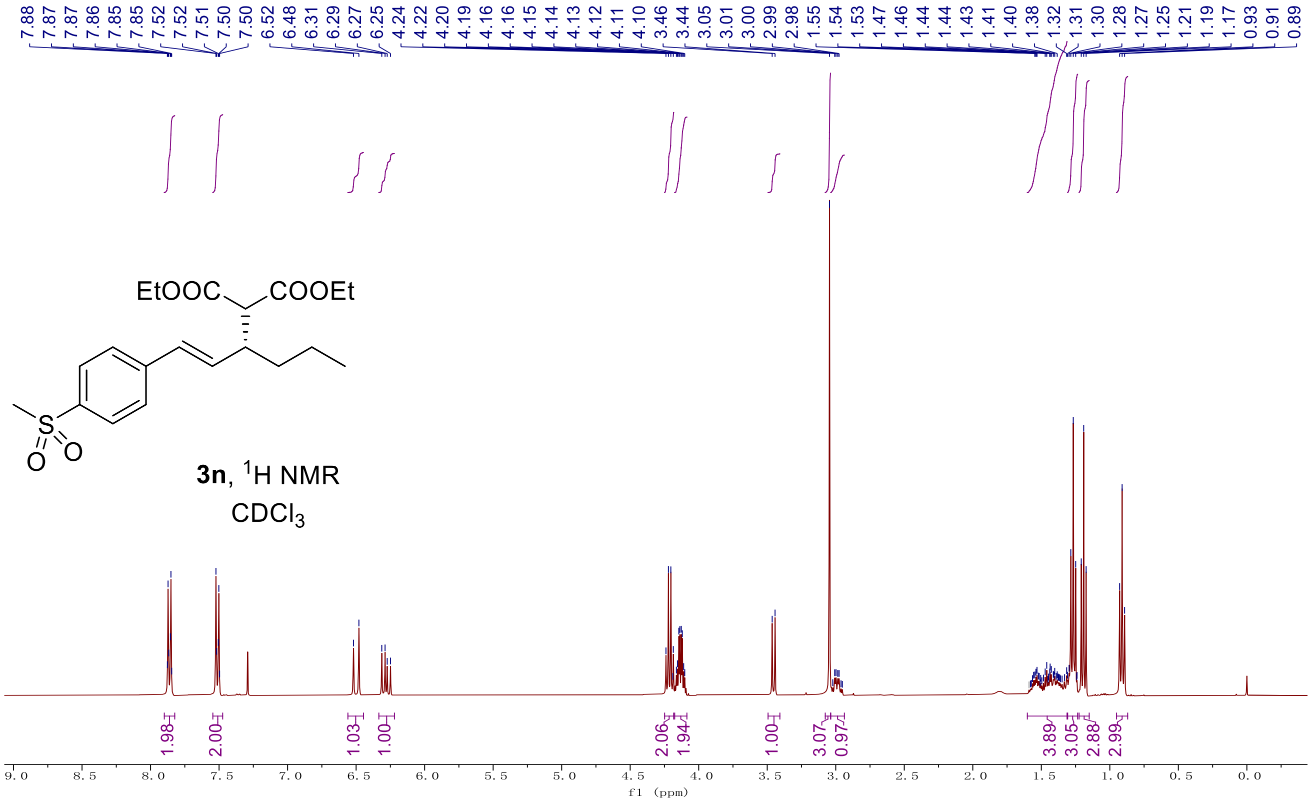

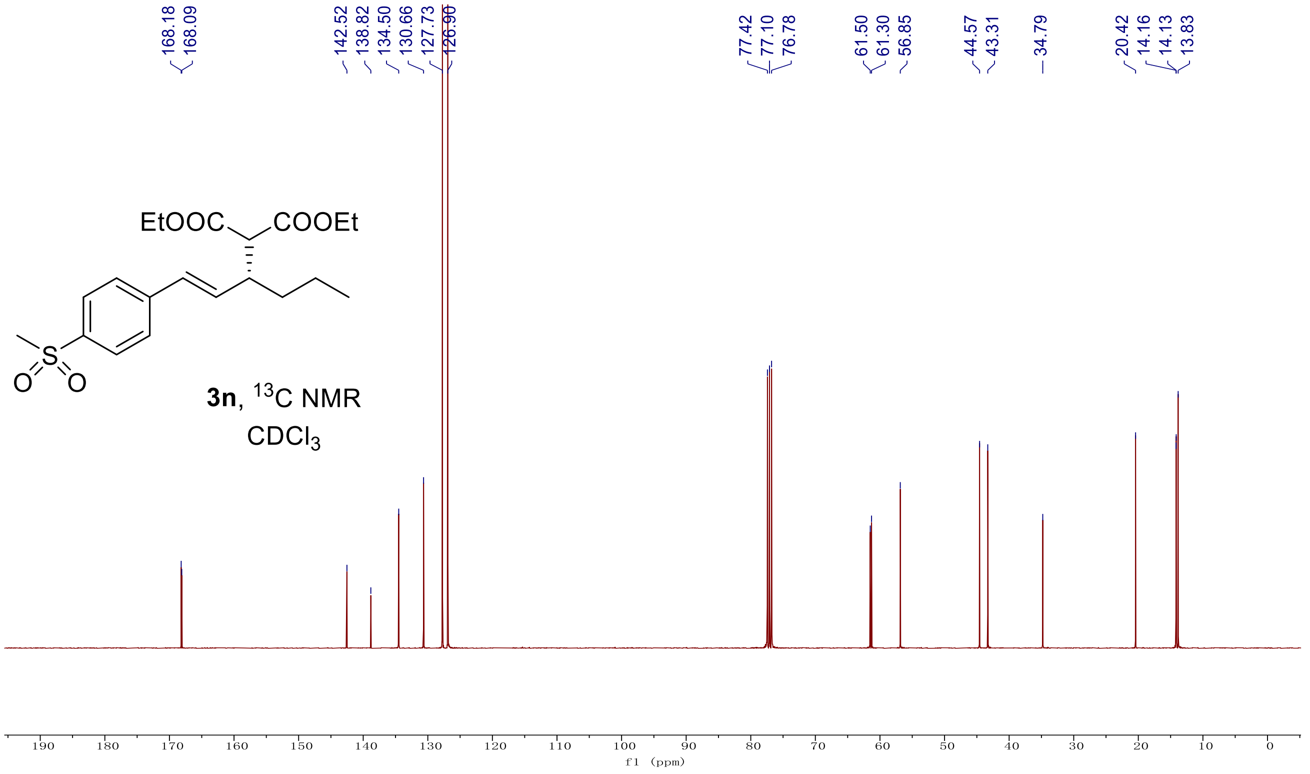

Supplementary Fig. 104. ^{1}H NMR and ^{13}C NMR spectra of compound **3n**.

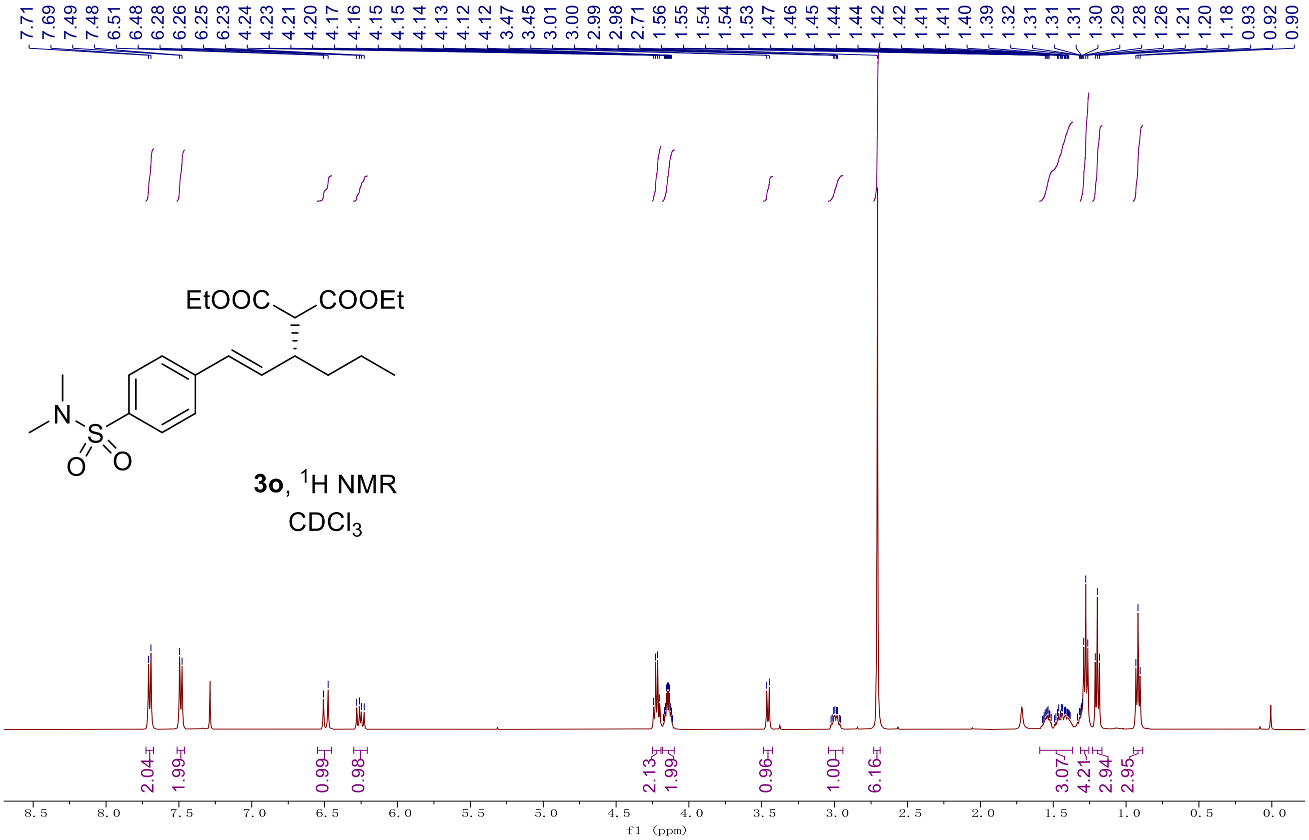

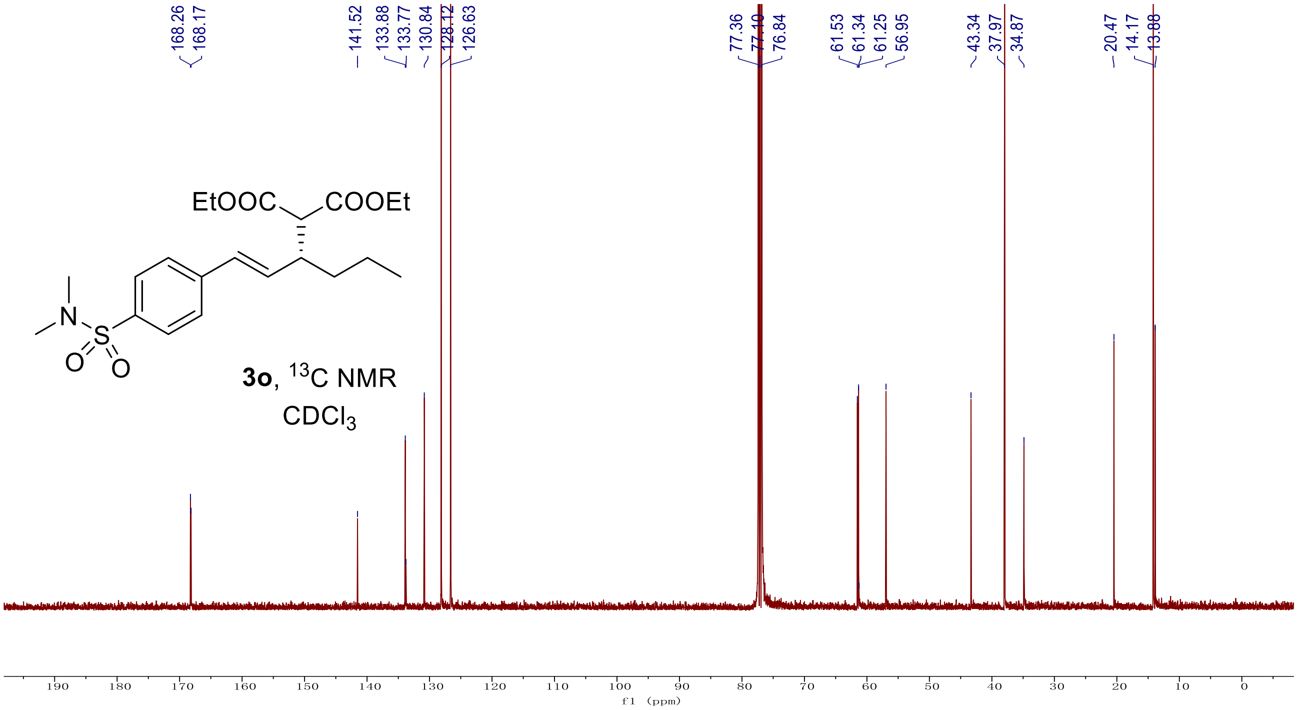

Supplementary Fig. 105. ^{1}H NMR and ^{13}C NMR spectra of compound **3o**.

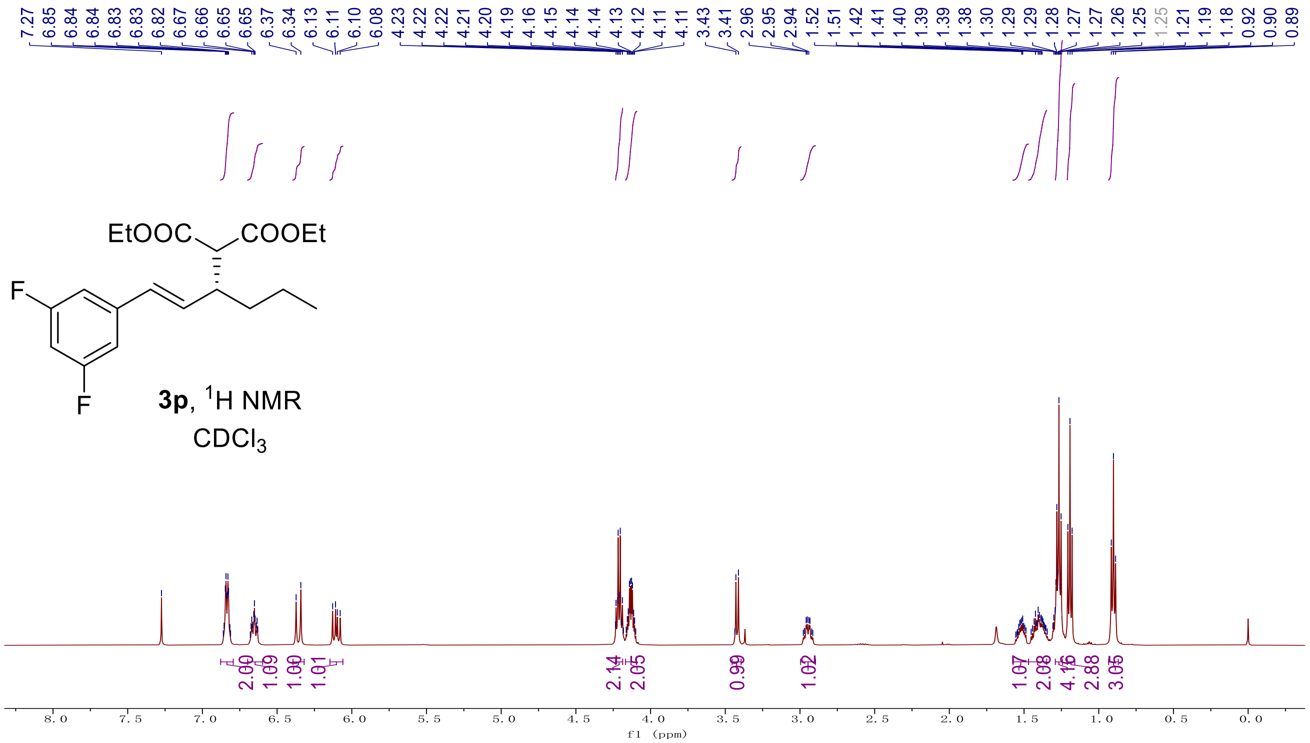
7.27 6.85 6.84 6.84 6.83 6.83 6.82 6.67 6.66 6.65 6.65 6.37 6.34 6.13 6.11 6.10 6.08 4.23 4.22 4.22 4.21 4.20 4.19 4.16 4.15 4.14 4.14 4.13 4.12 4.11 4.11 3.43 3.41 2.96 2.95 2.94 1.52 1.51 1.42 1.41 1.40 1.39 1.39 1.38 1.30 1.29 1.29 1.28 1.27 1.26 1.25 1.25 1.21 1.19 1.18 0.92 0.90 0.89
EtOOC
COOEt
F
F
3p, 1H NMR
CDCl3
2.00 1.09 1.00 1.01 2.14 2.05 0.99 1.02 1.07 2.08 4.16 2.88 3.05
f1 (ppm)

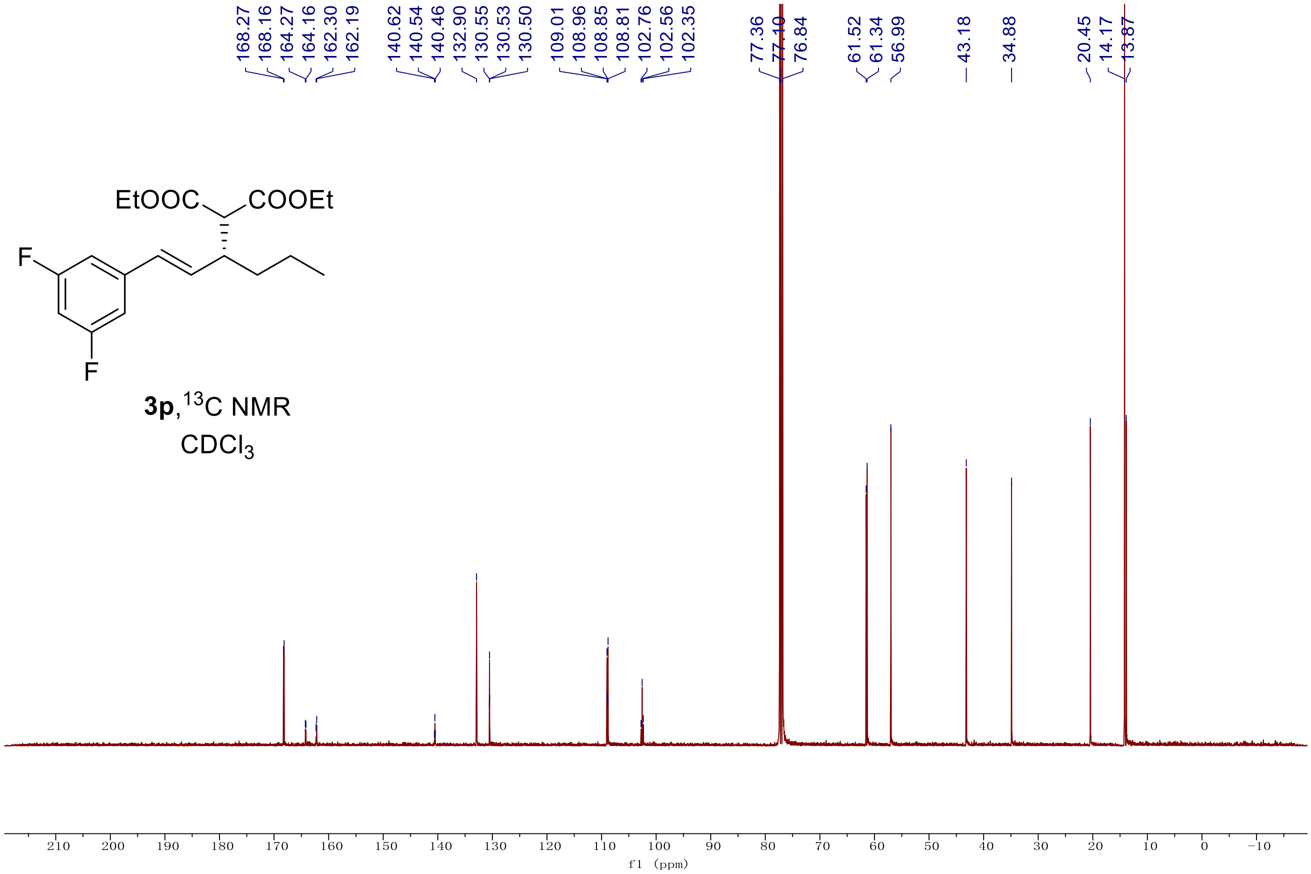
168.27 168.16 164.27 164.16 162.30 162.19 140.62 140.54 140.46 132.90 130.55 130.53 130.50 109.01 108.96 108.85 108.81 102.76 102.56 102.35 77.36 77.10 76.84 61.52 61.34 56.99 43.18 34.88 20.45 14.17 13.87
EtOOC
COOEt
F
F
3p, 13C NMR
CDCl3
f1 (ppm)

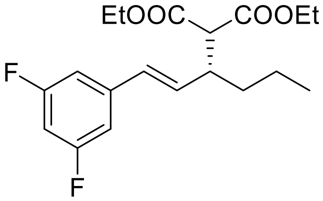

3p, ^{19}F NMR
$CDCl_3$

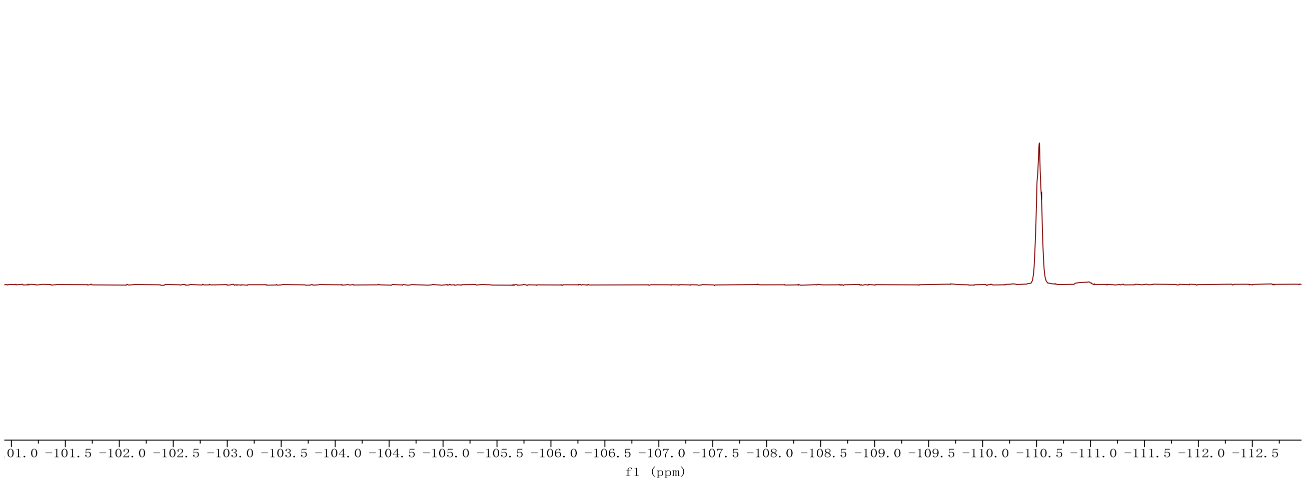

Supplementary Fig. 106. ^{1}H NMR, ^{13}C NMR and ^{19}F NMR spectra of compound **3p**.

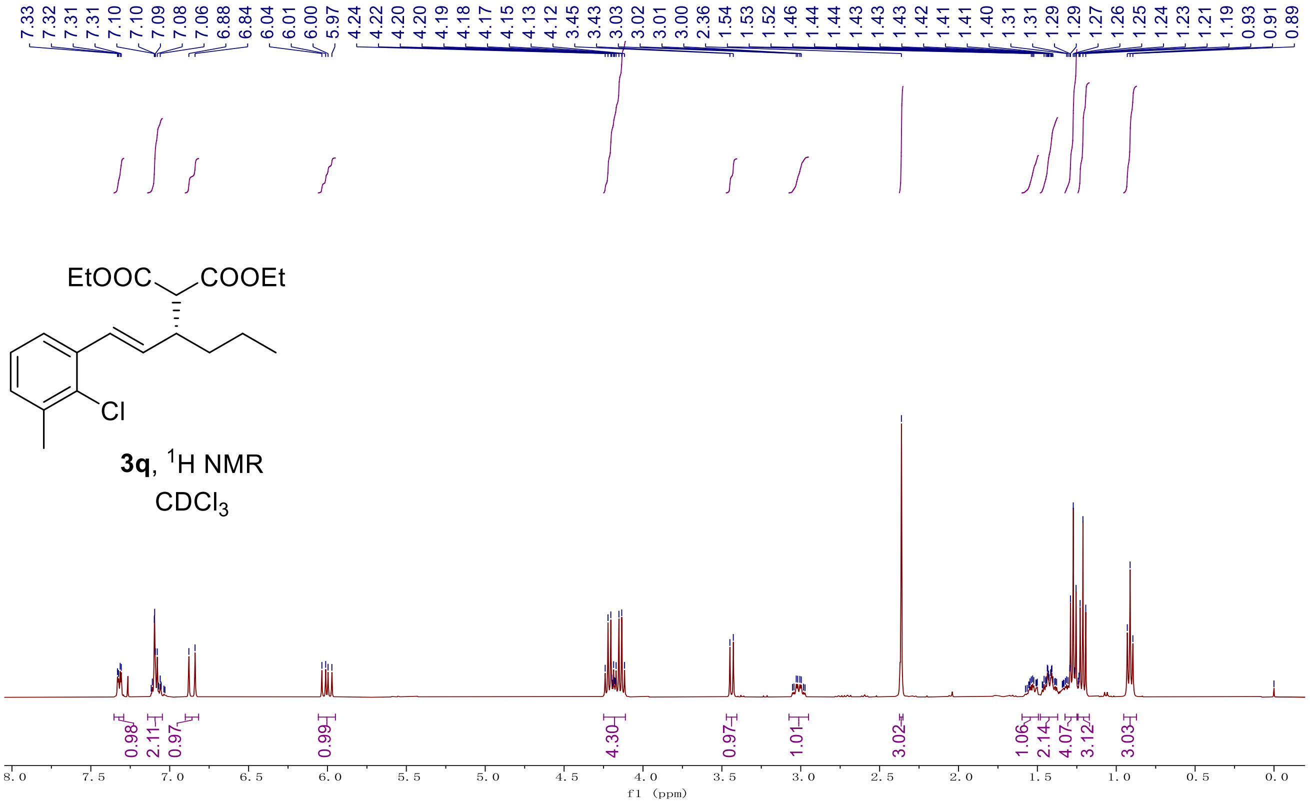

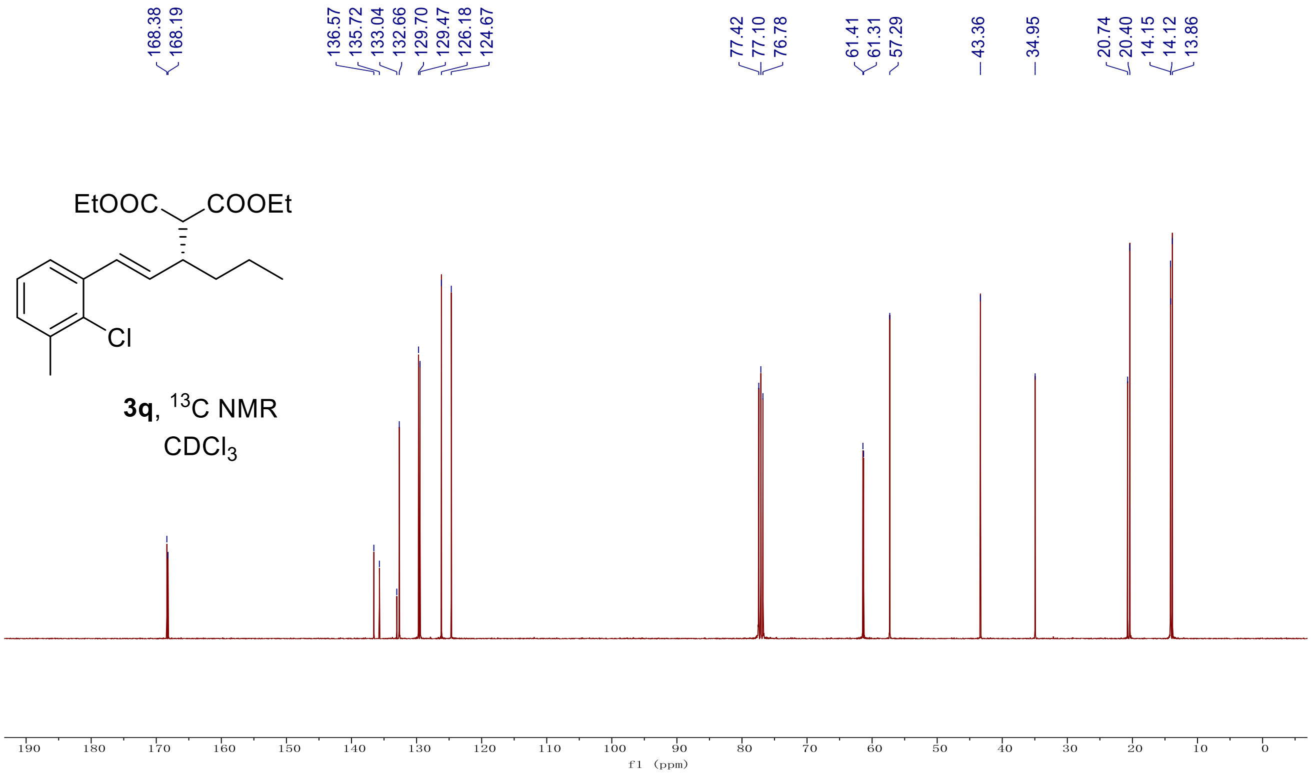

Supplementary Fig. 107. ^{1}H NMR amd ^{13}C NMR spectra of compound **3q**.

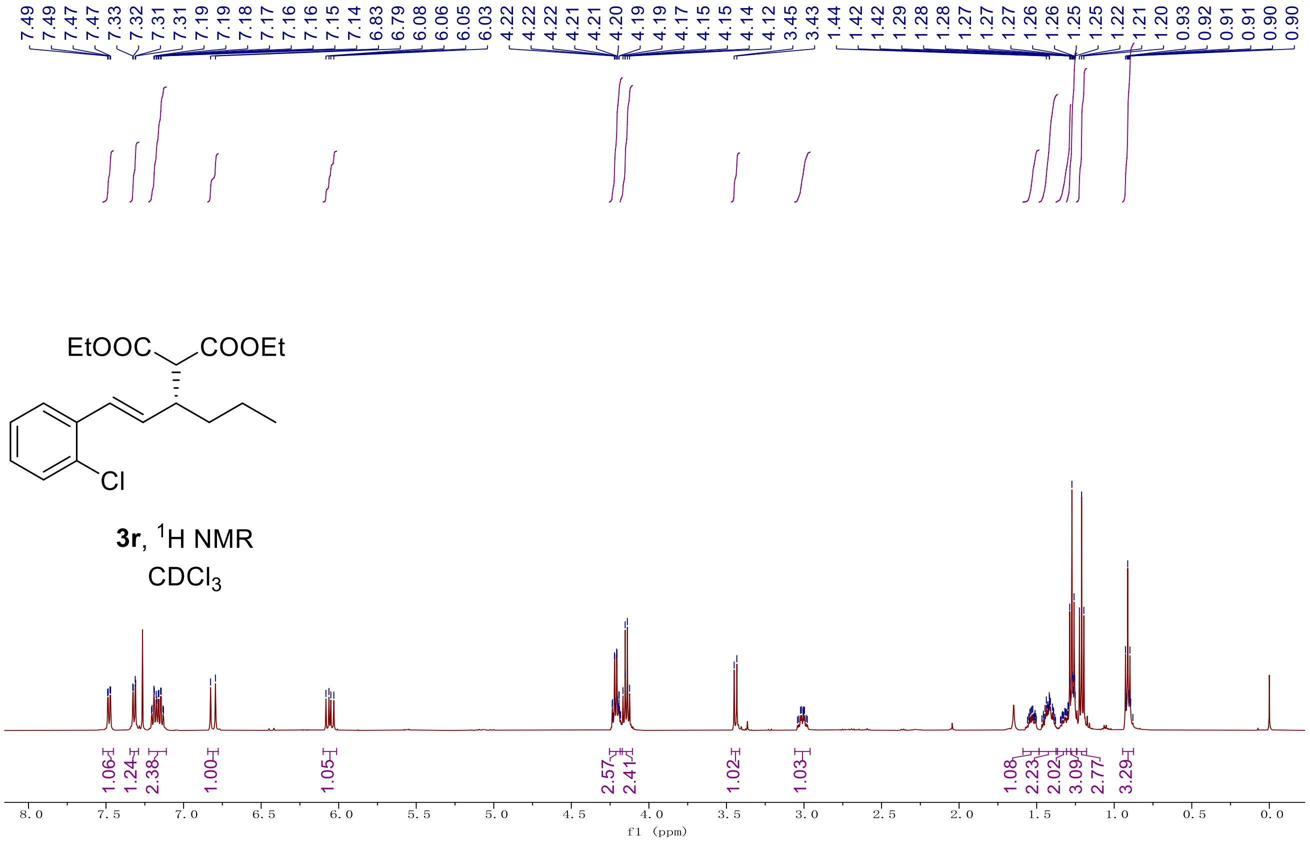

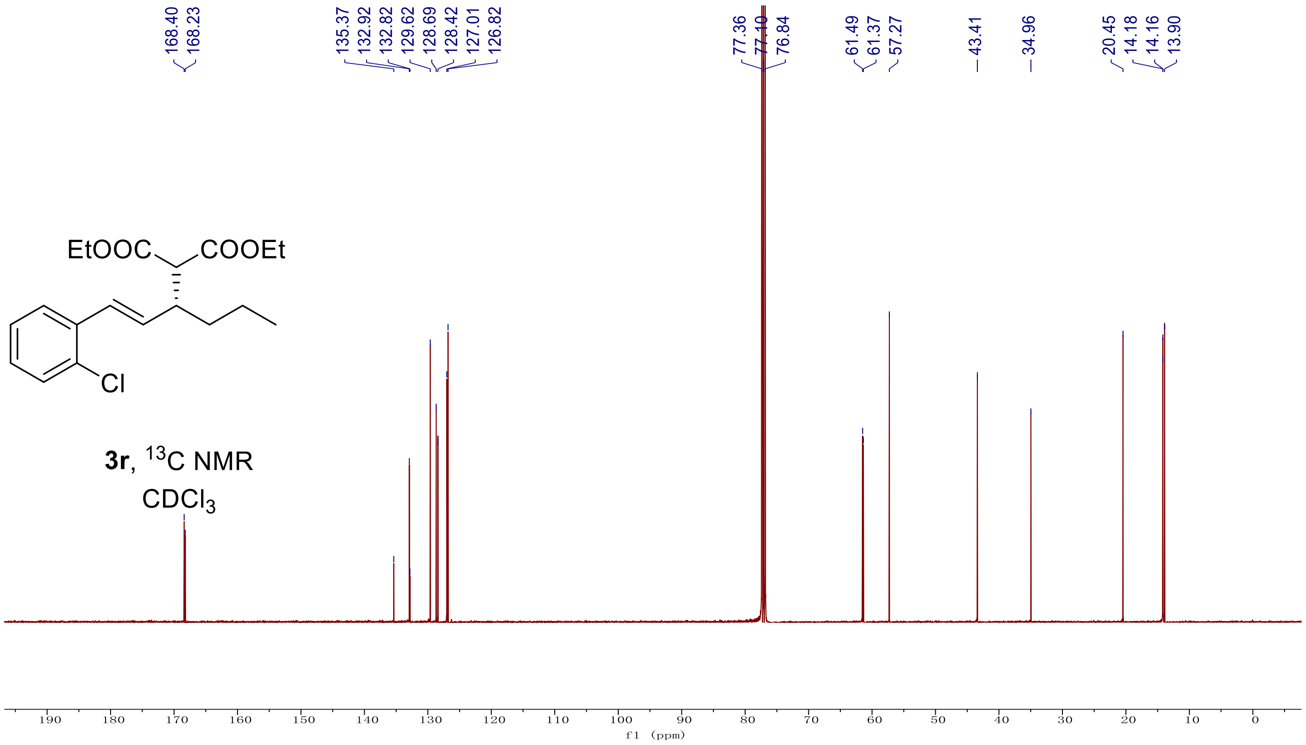

Supplementary Fig. 108. ^{1}H NMR and ^{13}C NMR spectra of compound **3r**.

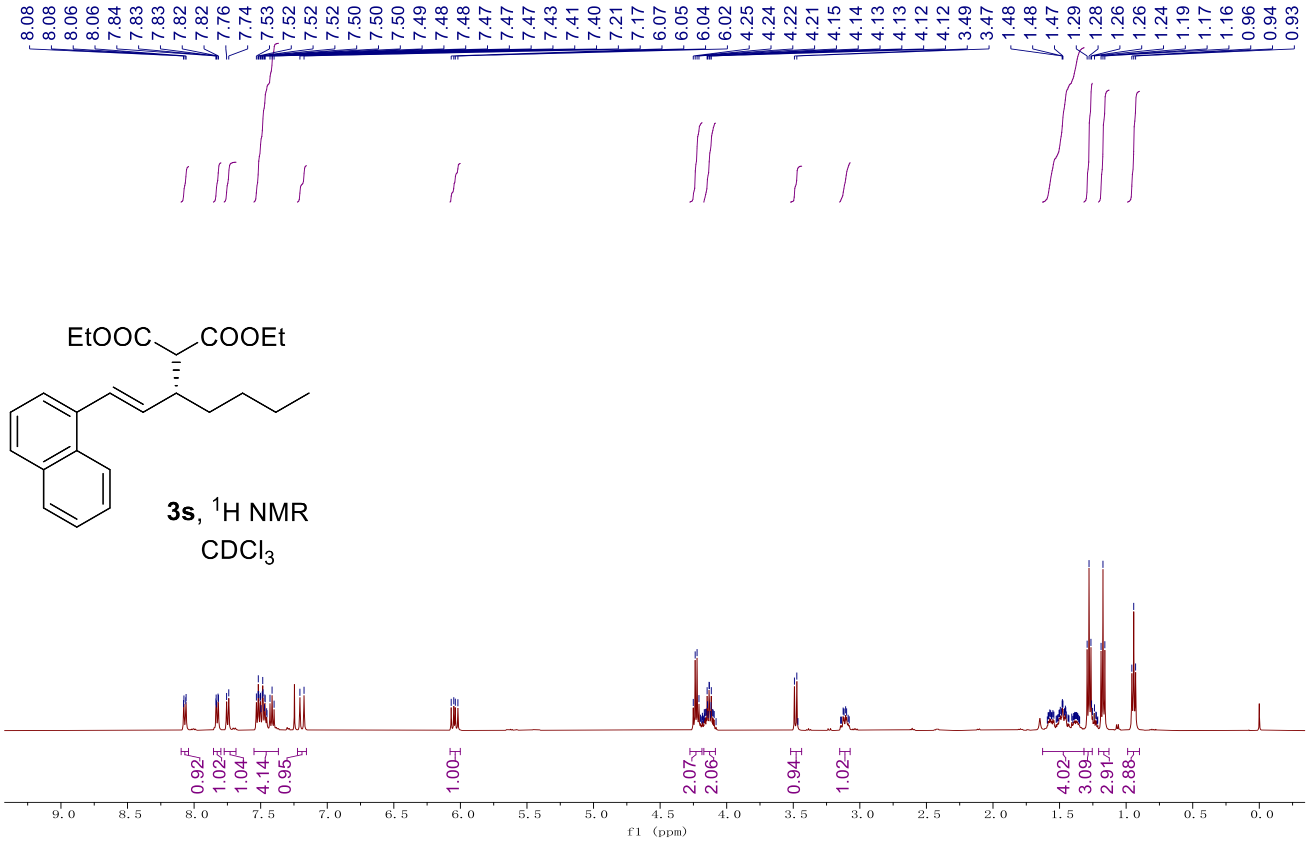

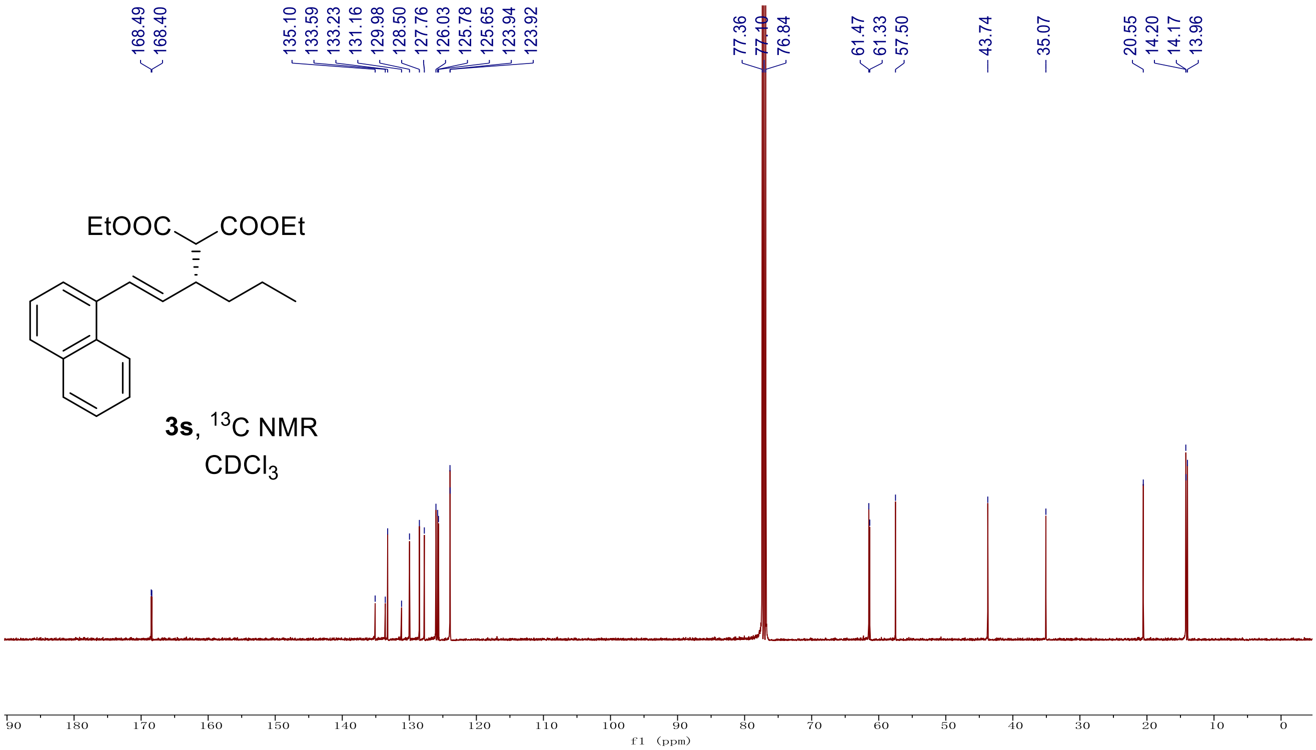

Supplementary Fig. 109. ^{1}H NMR and ^{13}C NMR spectra of compound **3s**.

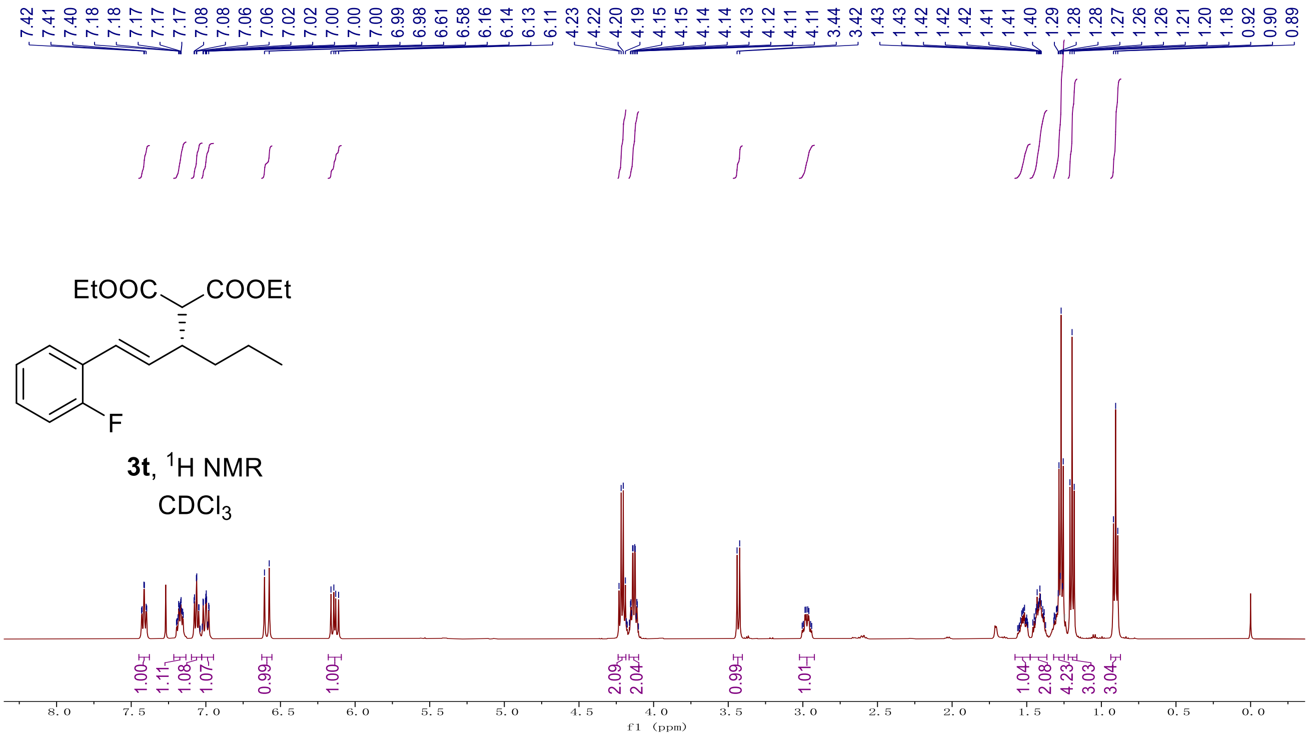
7.42 7.41 7.40 7.18 7.18 7.17 7.17 7.17 7.08 7.08 7.06 7.06 7.02 7.02 7.00 7.00 7.00 6.99 6.98 6.61 6.58 6.16 6.14 6.13 6.11 4.23 4.22 4.20 4.19 4.15 4.15 4.14 4.14 4.13 4.12 4.11 4.11 3.44 3.42 1.43 1.43 1.42 1.42 1.42 1.41 1.41 1.40 1.29 1.28 1.28 1.27 1.26 1.26 1.21 1.20 1.18 0.92 0.90 0.89
EtOOC
COOEt
F
3t, ¹H NMR
CDCl3
1.00 1.11 1.08 1.07 0.99 1.00 2.09 2.04 0.99 1.01 1.04 2.08 4.23 3.03 3.04
f1 (ppm)

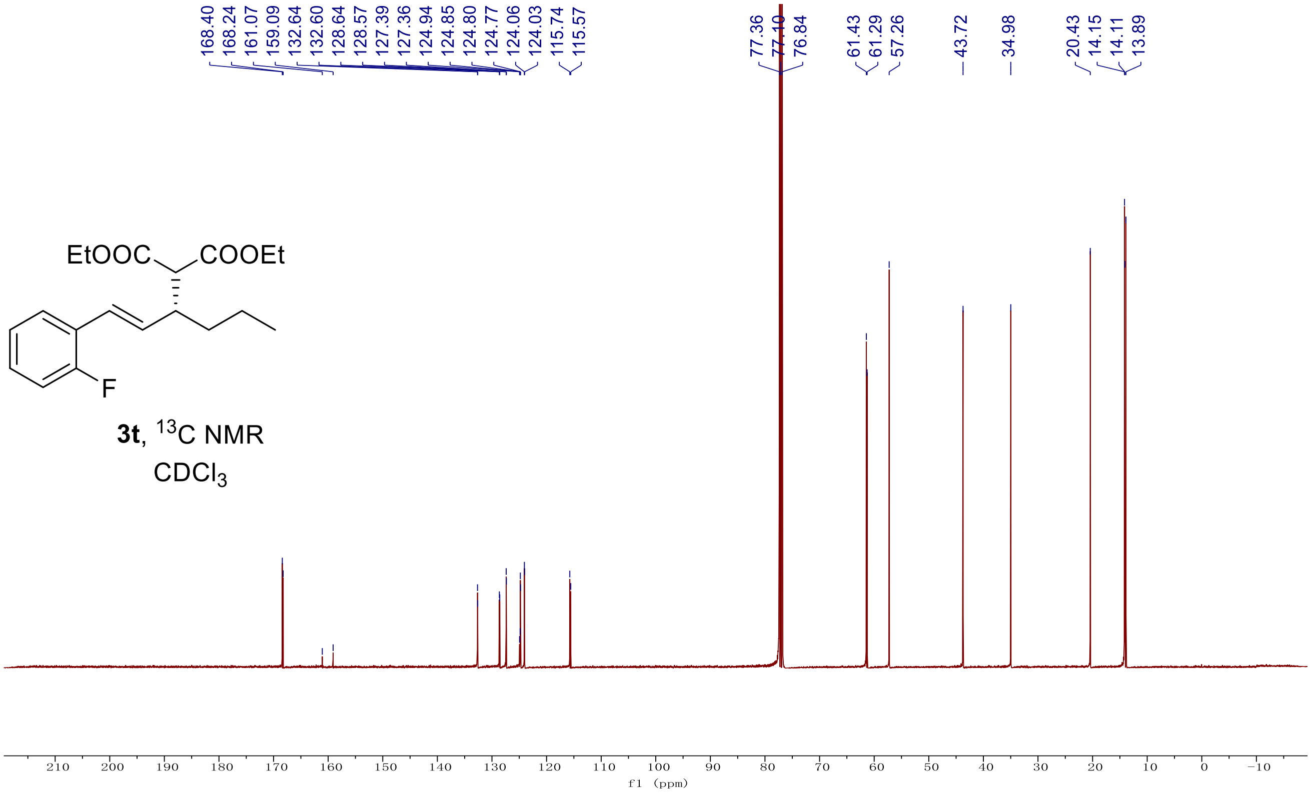
168.40 168.24 161.07 159.09 132.64 132.60 128.64 128.57 127.39 127.36 124.94 124.85 124.80 124.77 124.06 124.03 115.74 115.57
77.36 77.10 76.84
61.43 61.29 57.26
43.72
34.98
20.43 14.15 14.11 13.89
EtOOC
COOEt
F
3t, ¹³C NMR
CDCl3
f1 (ppm)

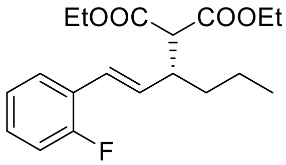

3t, ^{19}F NMR
$CDCl_3$

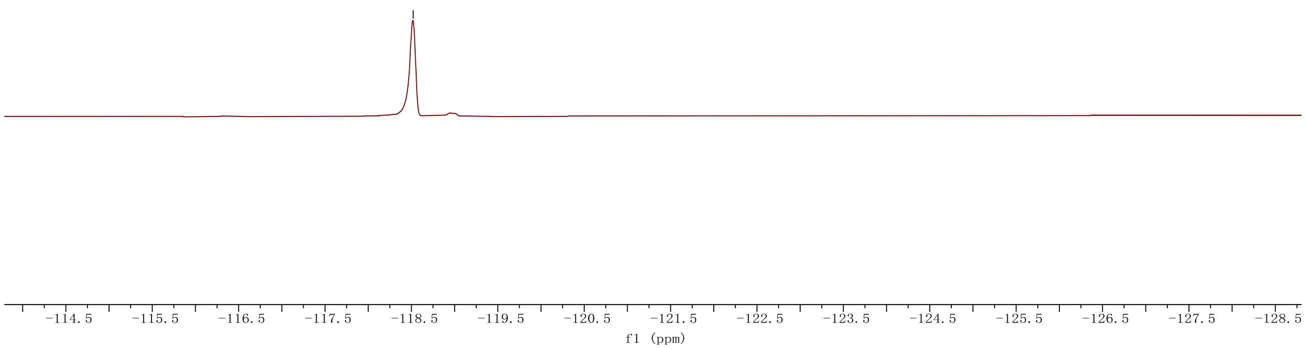

Supplementary Fig. 110. ^{1}H NMR, ^{13}C NMR and ^{19}F NMR spectra of compound **3t**.

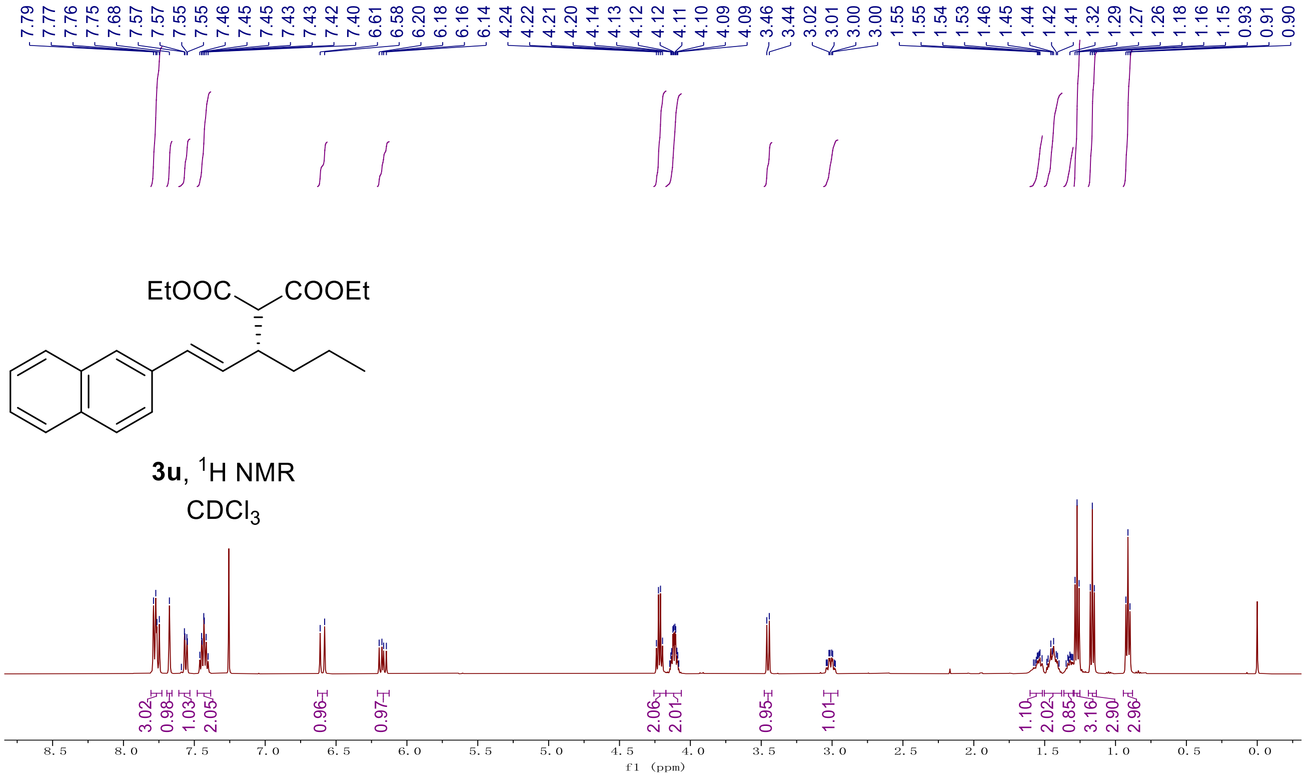

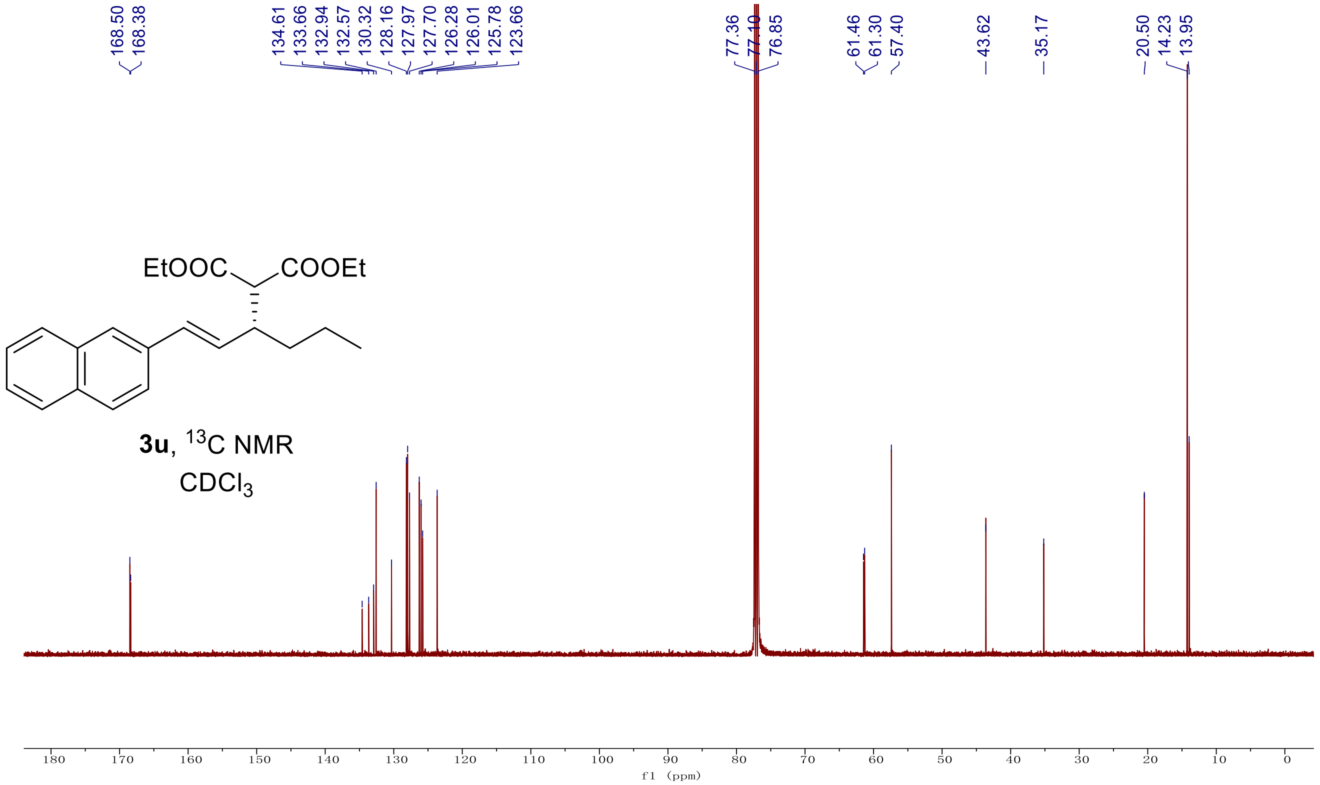

Supplementary Fig. 111. ^{1}H NMR and ^{13}C NMR spectra of compound **3u**.

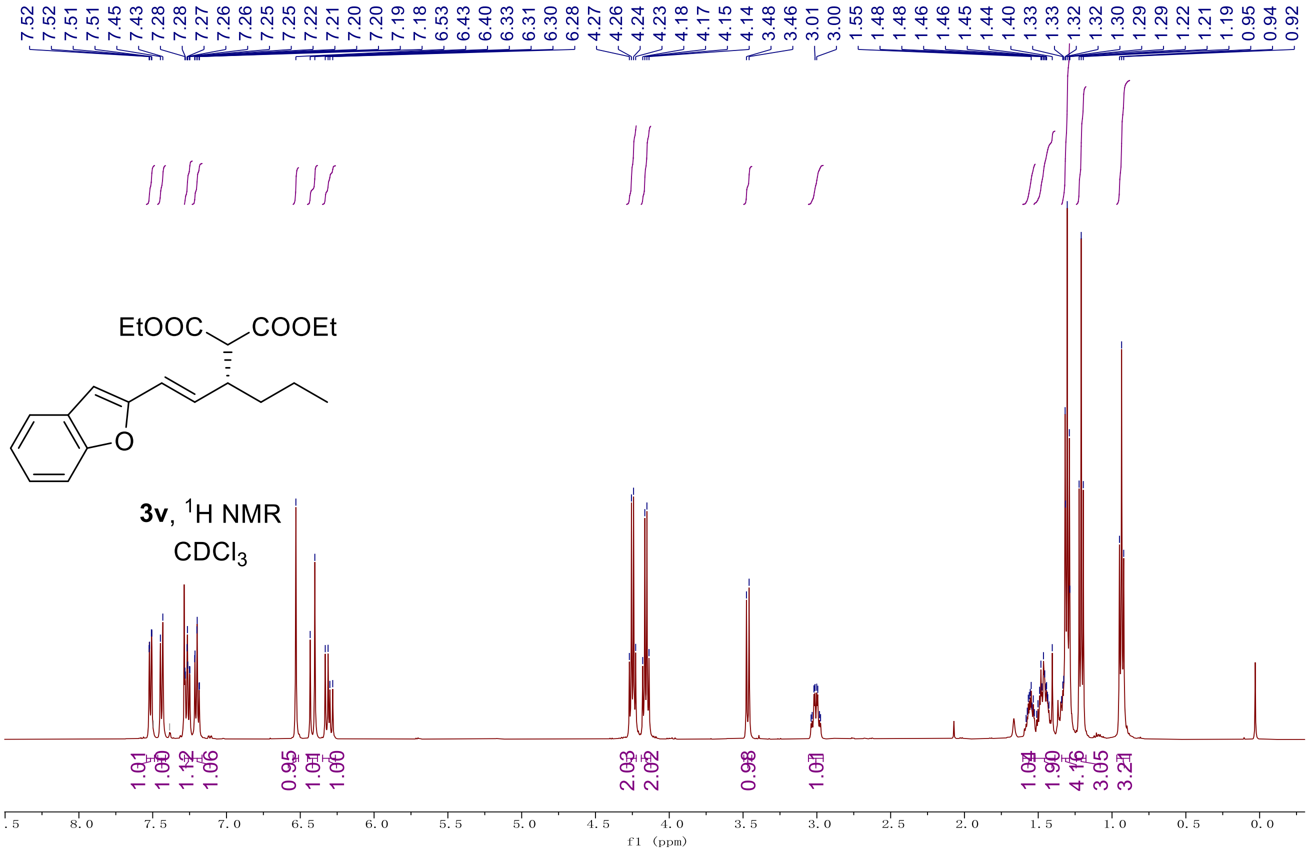

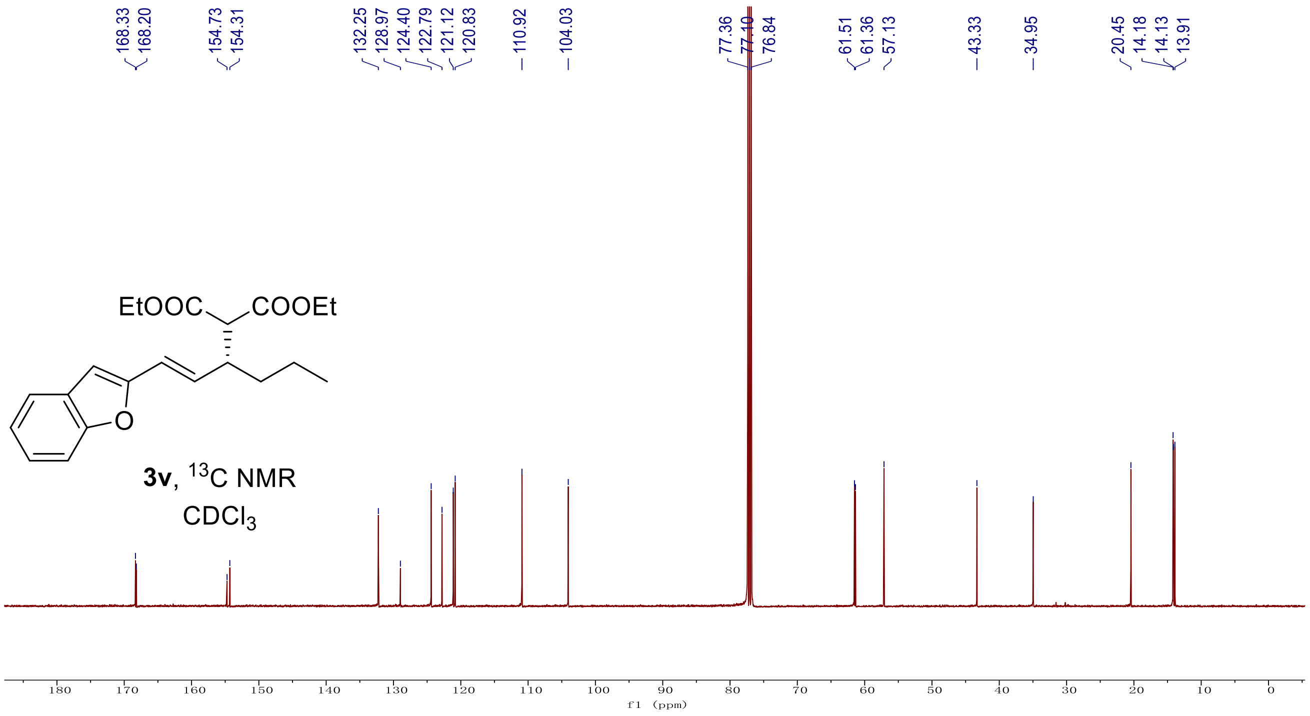

Supplementary Fig. 112. ^{1}H NMR and ^{13}C NMR spectra of compound **3v**.

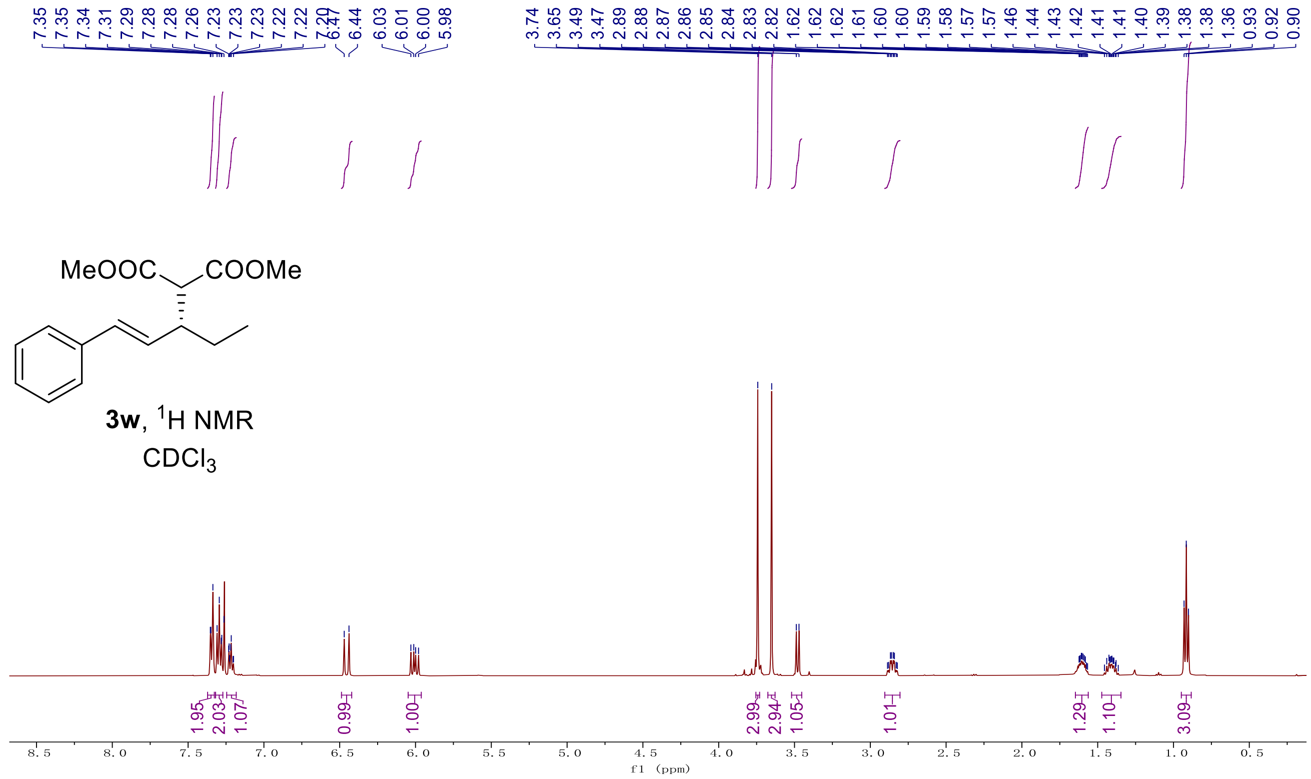

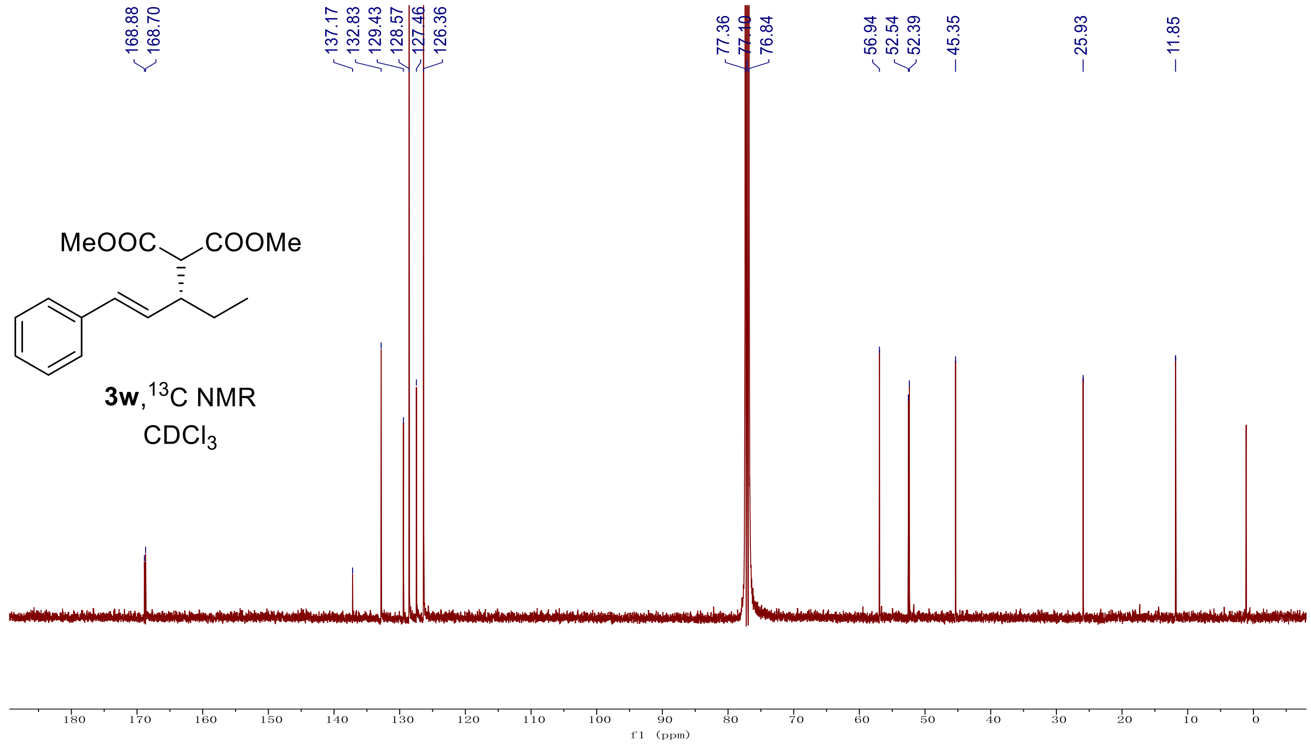

Supplementary Fig. 113. ^{1}H NMR and ^{13}C NMR spectra of compound **3w**.

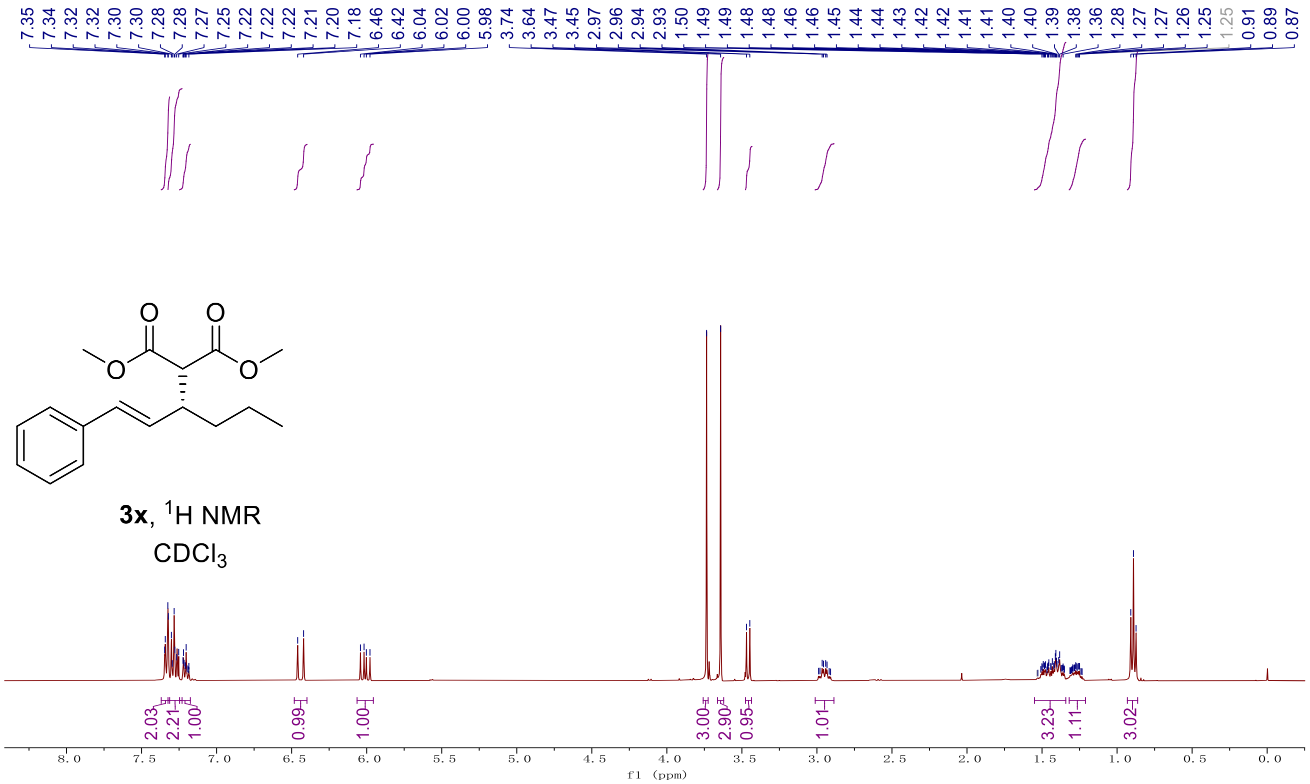

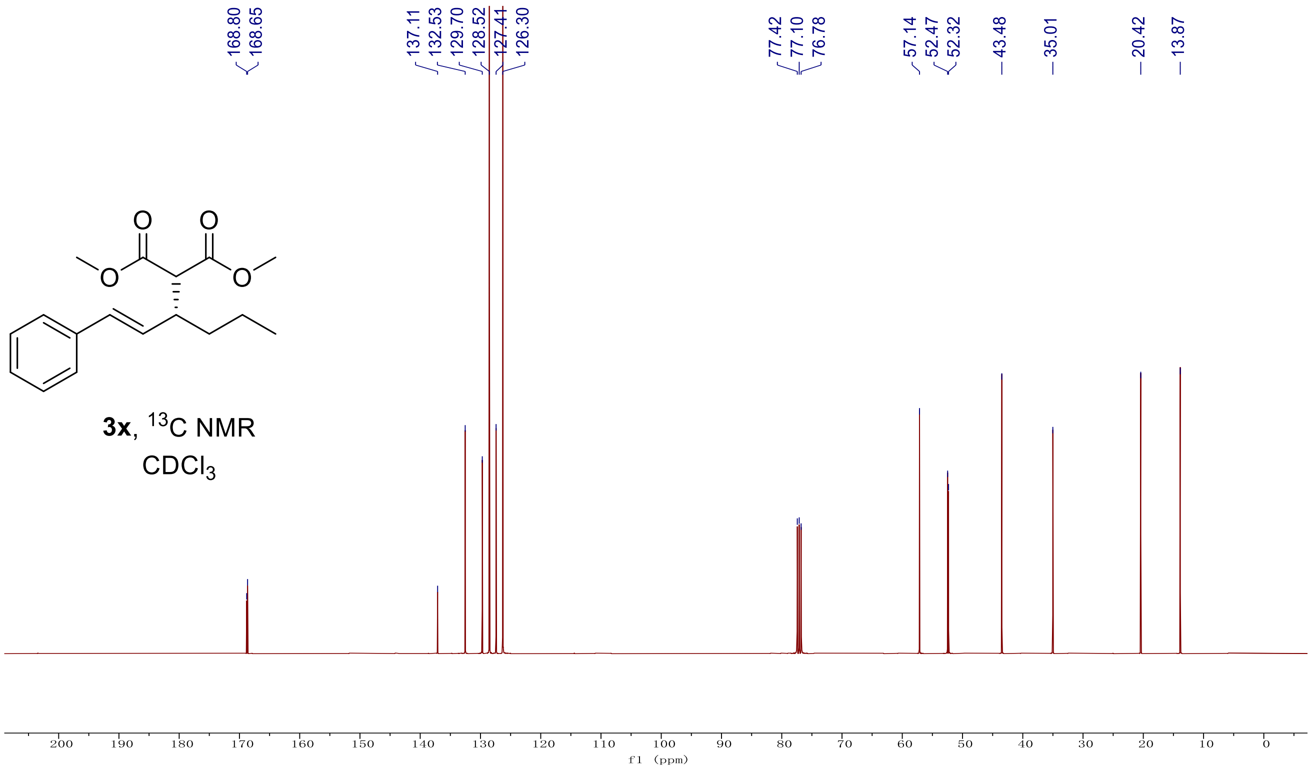

Supplementary Fig. 114. ^{1}H NMR and ^{13}C NMR spectra of compound **3x**.

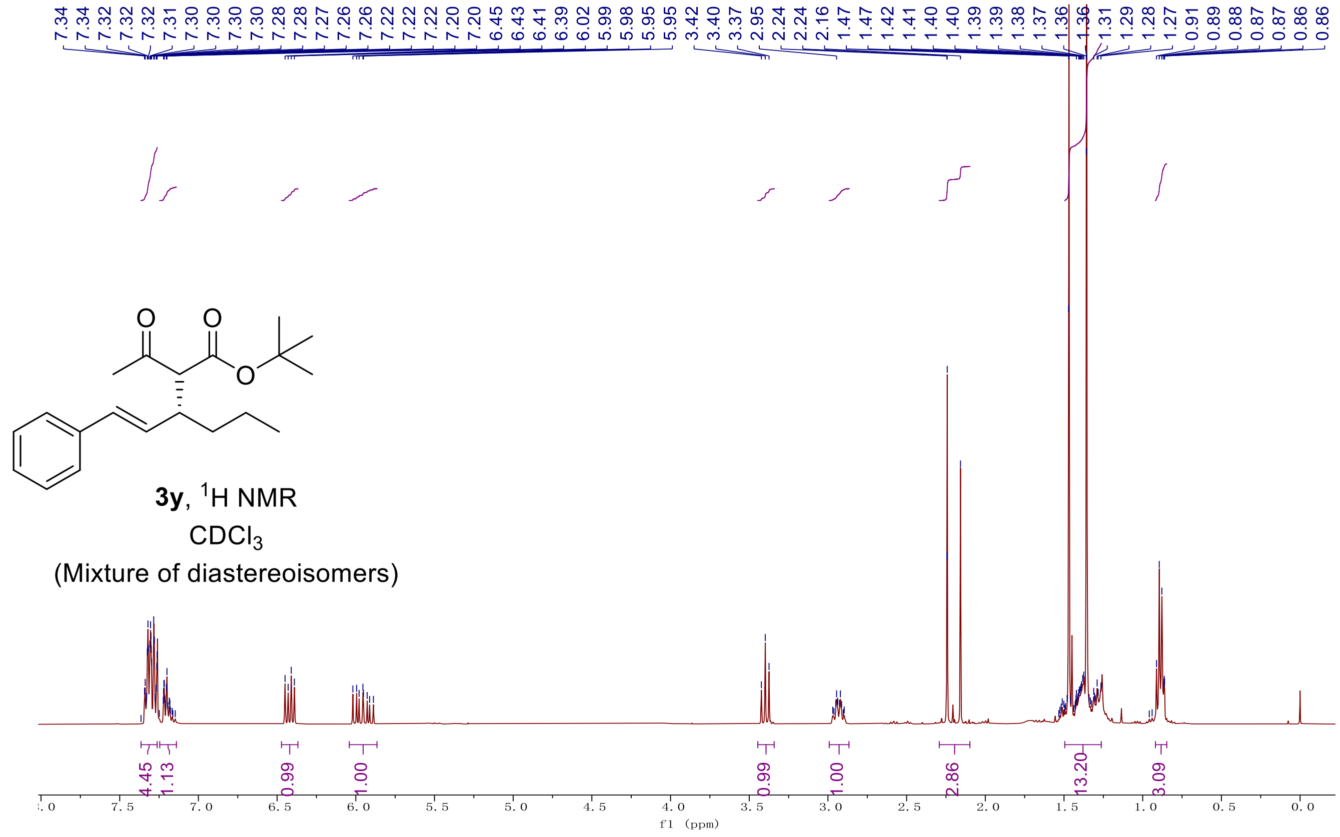

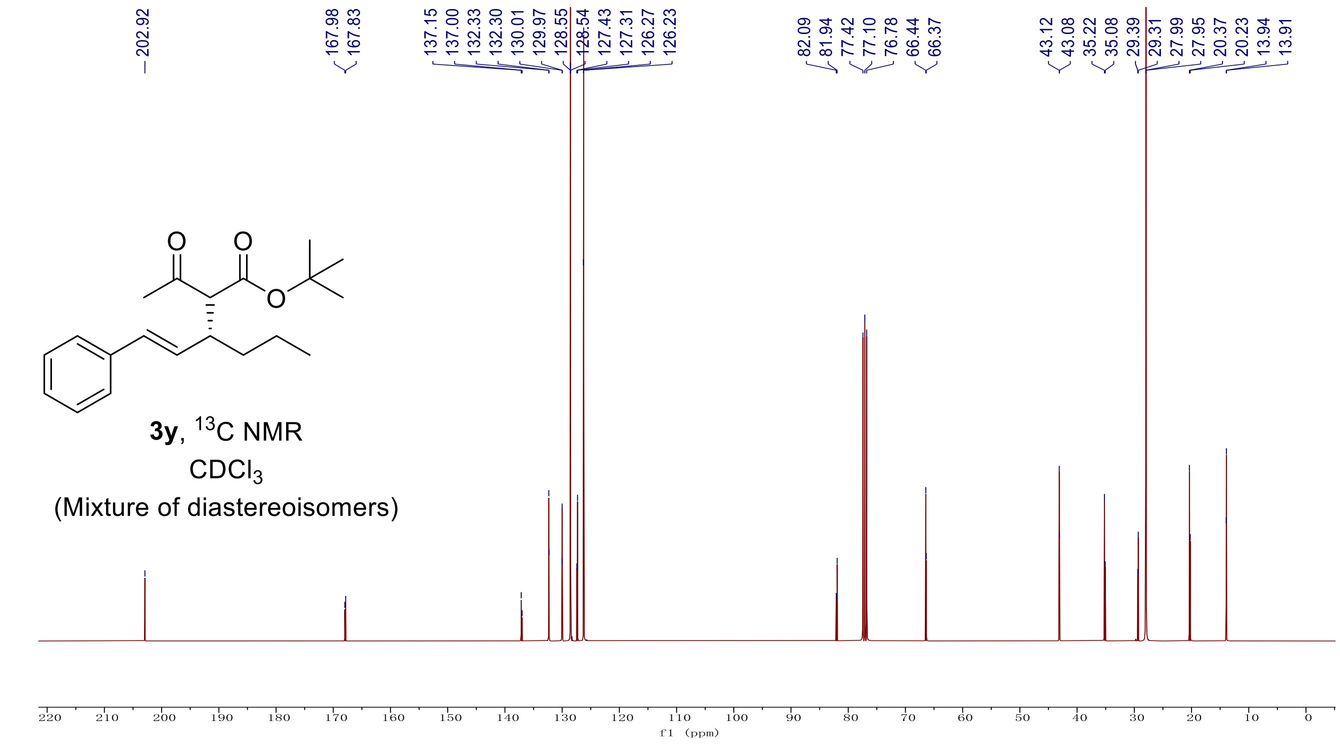

Supplementary Fig. 115. ^{1}H NMR and ^{13}C NMR spectra of compound **3y**.

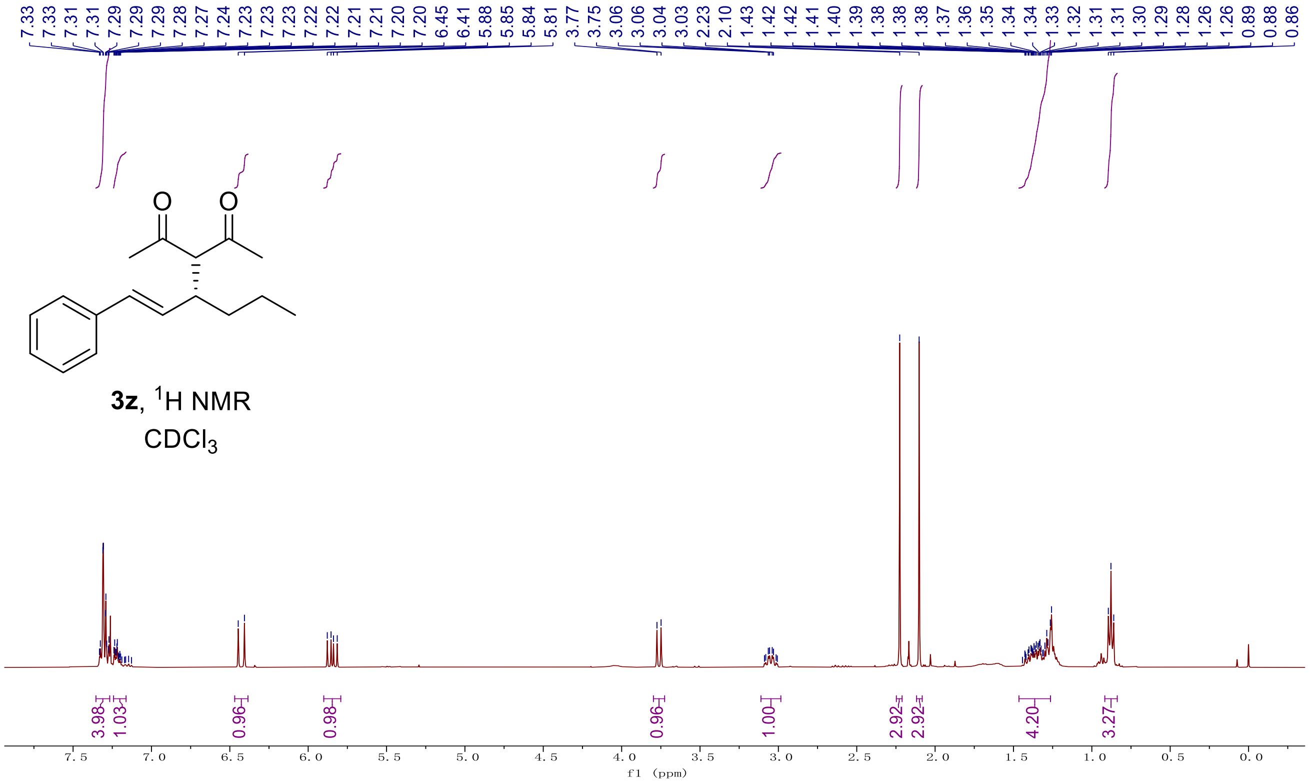

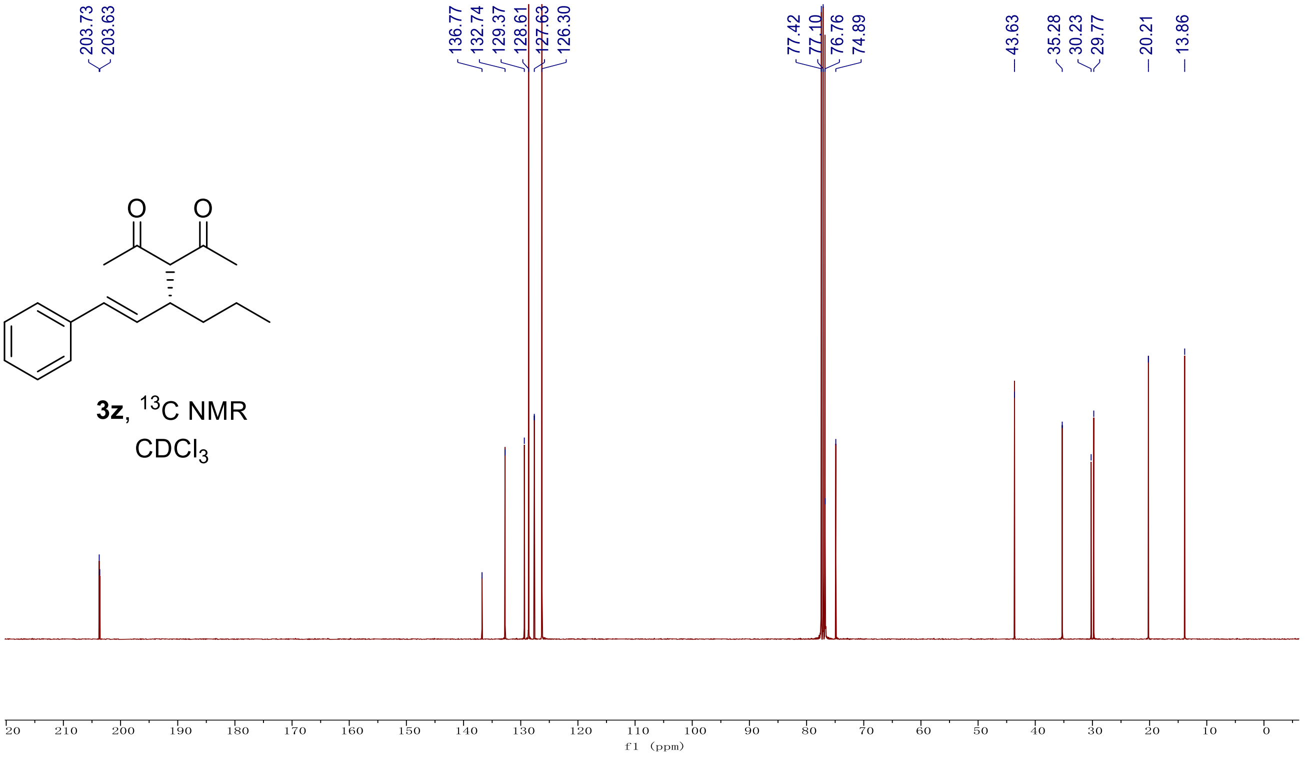

Supplementary Fig. 116. ^{1}H NMR and ^{13}C NMR spectra of compound **3z**.

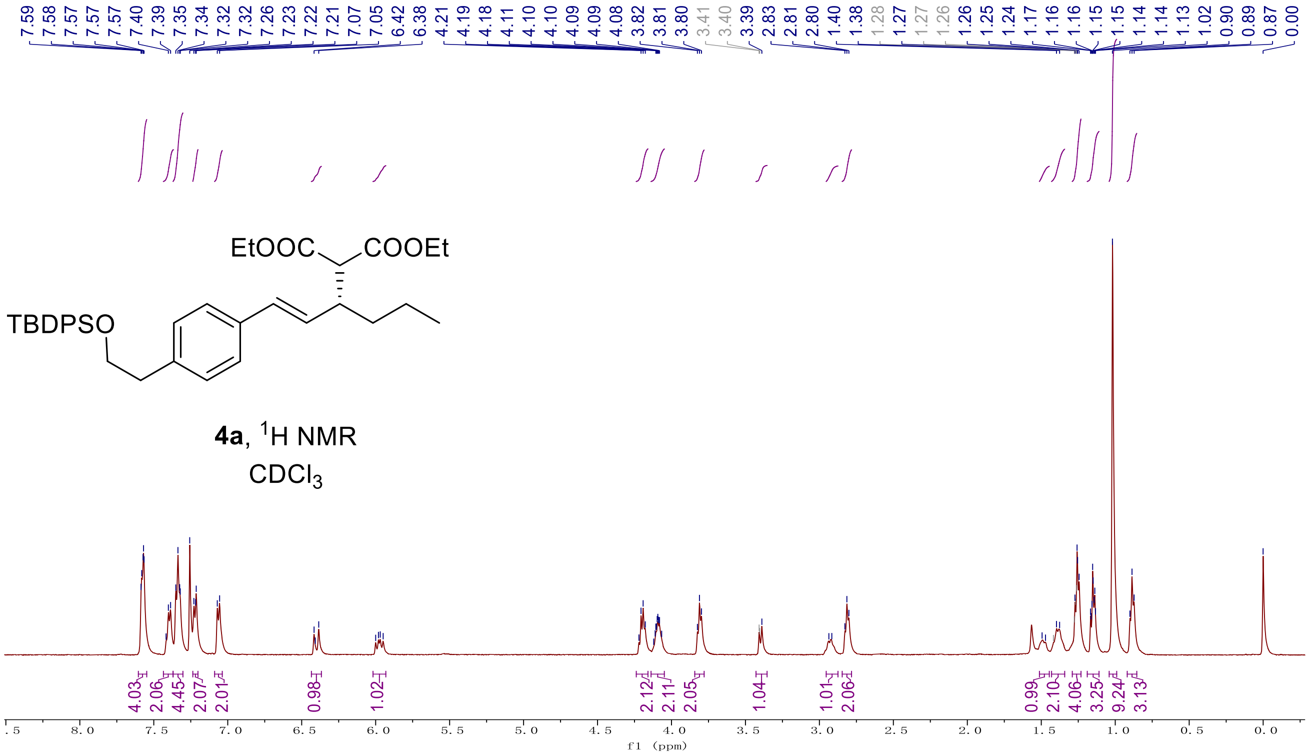

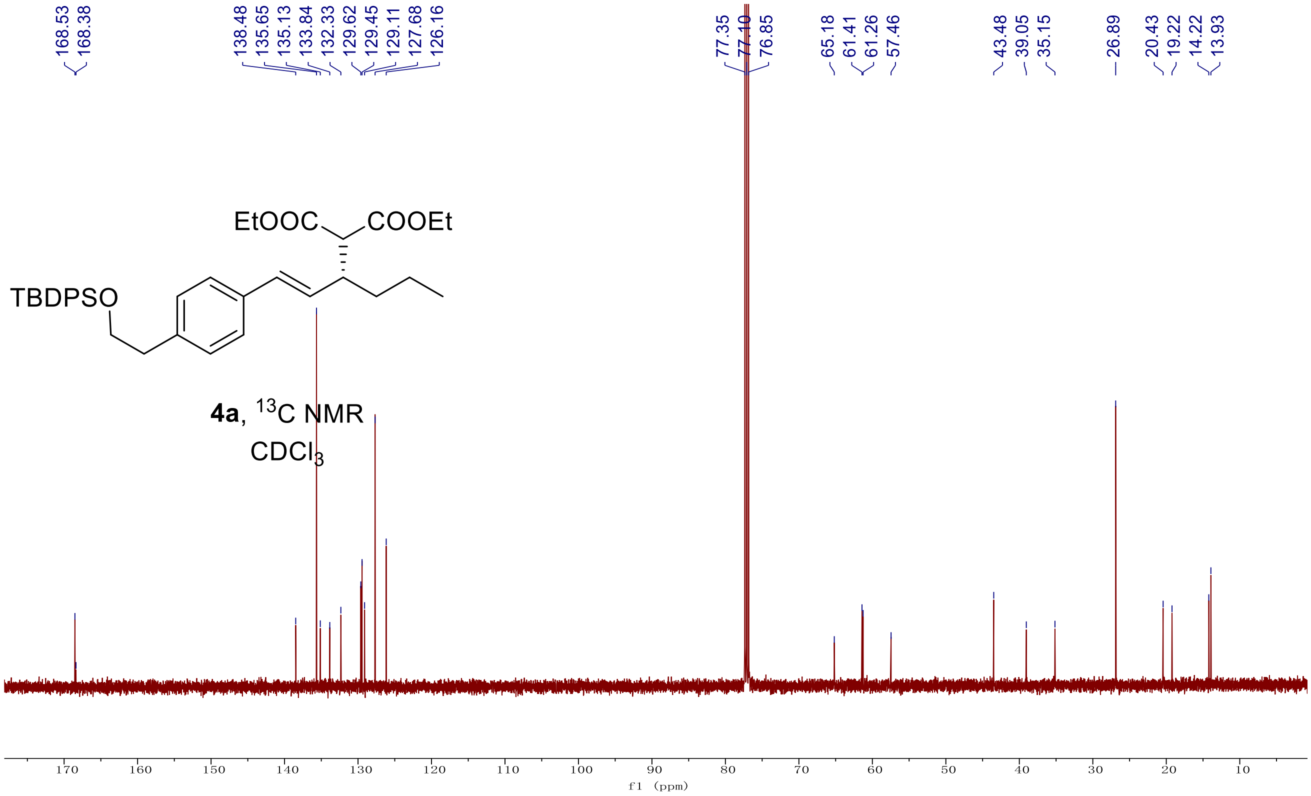

Supplementary Fig. 117. ^{1}H NMR and ^{13}C NMR spectra of compound **4a**.

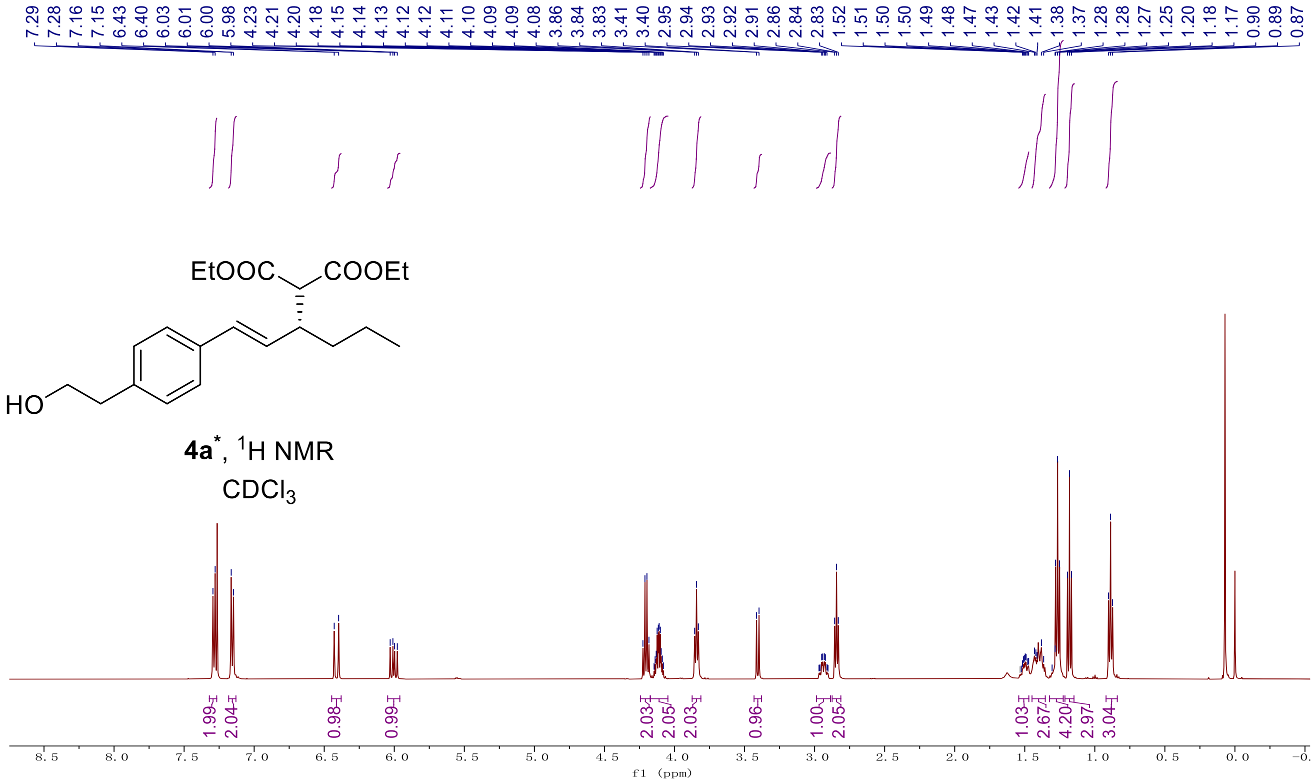

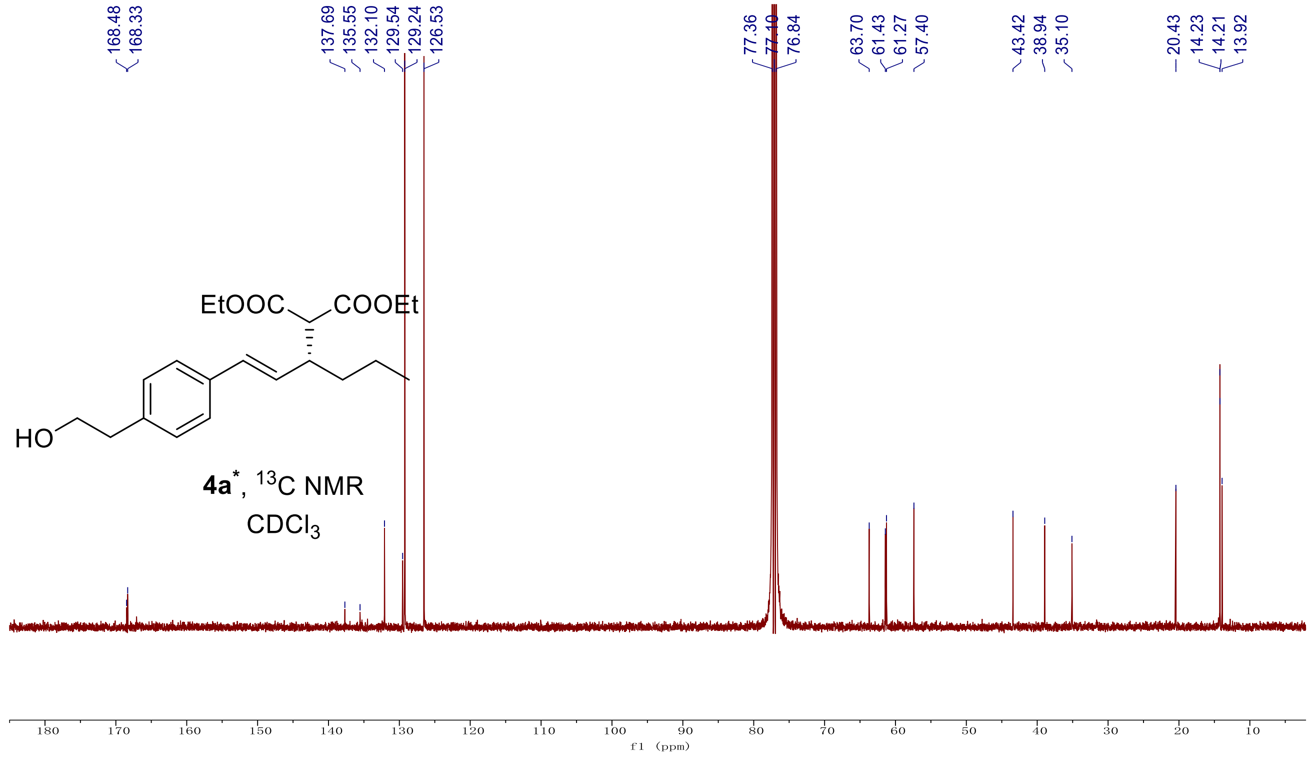

Supplementary Fig. 118. ^{1}H NMR and ^{13}C NMR spectra of compound **4a'**.

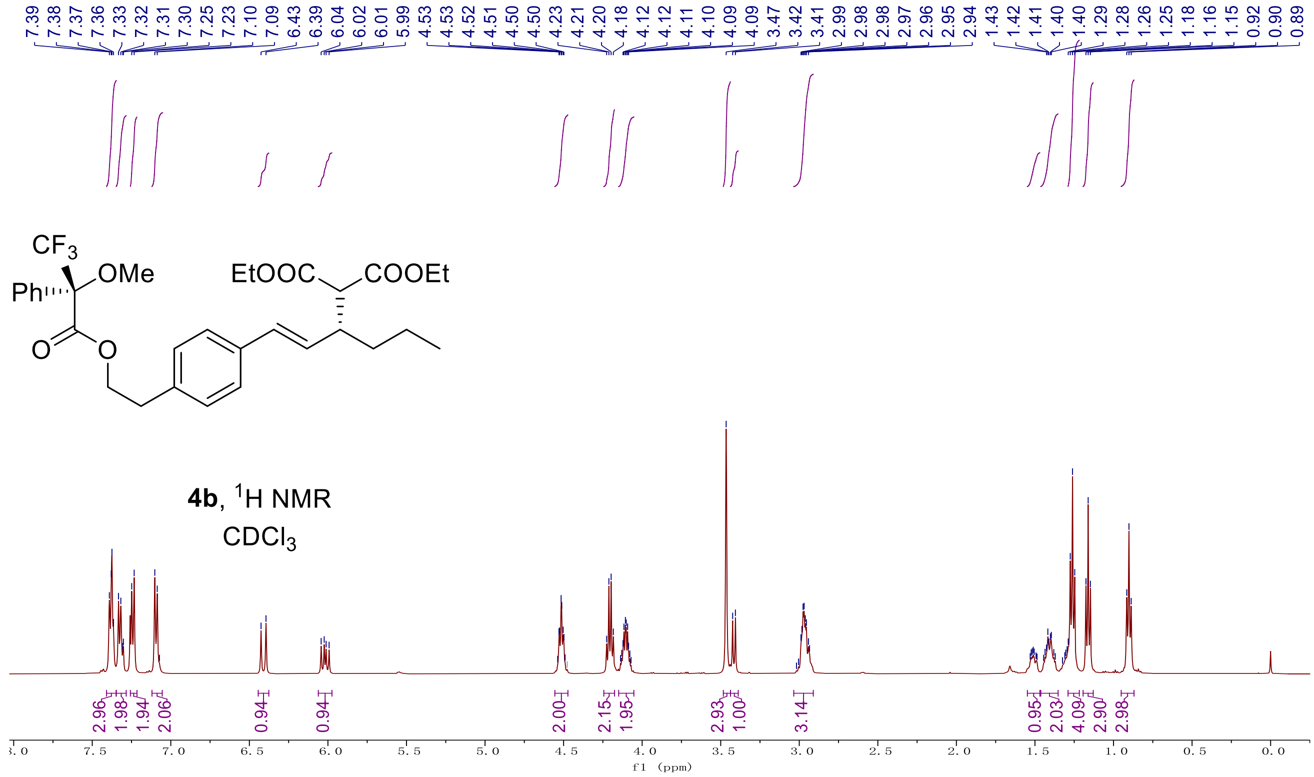
7.39 7.38 7.37 7.36 7.33 7.32 7.31 7.30 7.25 7.23 7.10 7.09 6.43 6.39 6.04 6.02 6.01 5.99 4.53 4.52 4.51 4.50 4.50 4.23 4.21 4.20 4.18 4.12 4.12 4.11 4.10 4.09 4.09 3.47 3.42 3.41 2.99 2.98 2.98 2.97 2.96 2.95 2.94 1.43 1.42 1.41 1.40 1.40 1.29 1.28 1.26 1.25 1.18 1.16 1.15 0.92 0.90 0.89
CF3
OMe
Ph
EtOOC
COOEt
O
O
4b, 1H NMR
CDCl3
2.96 1.98 1.94 2.06 0.94 0.94 2.00 2.15 1.95 2.93 1.00 3.14 0.95 2.03 4.09 2.90 2.98
f1 (ppm)

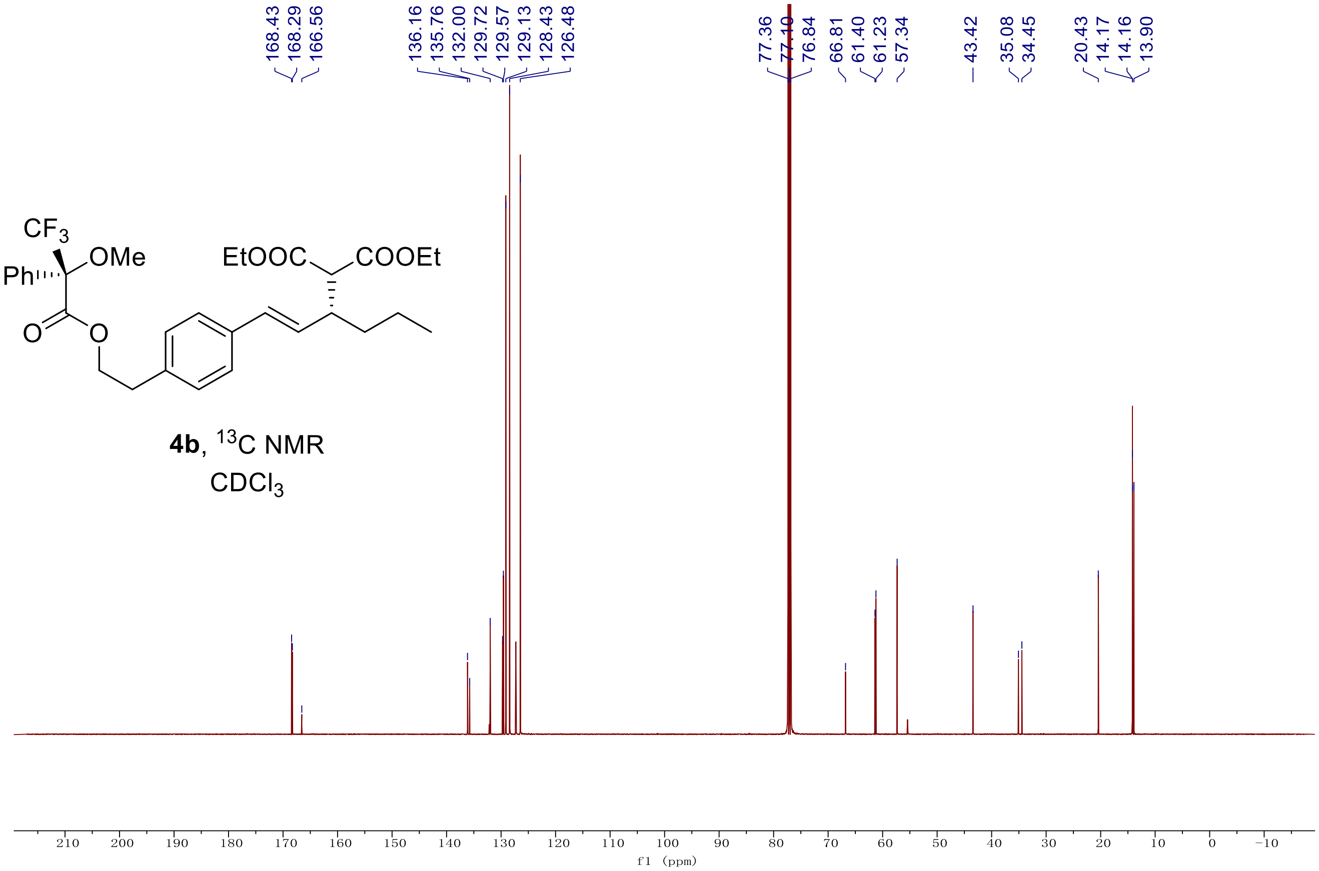
168.43 168.29 166.56 136.16 135.76 132.00 129.72 129.57 129.13 128.43 126.48 77.36 77.10 76.84 66.81 61.40 61.23 57.34 43.42 35.08 34.45 20.43 14.17 14.16 13.90
CF3
OMe
Ph
EtOOC
COOEt
O
O
4b, 13C NMR
CDCl3
f1 (ppm)

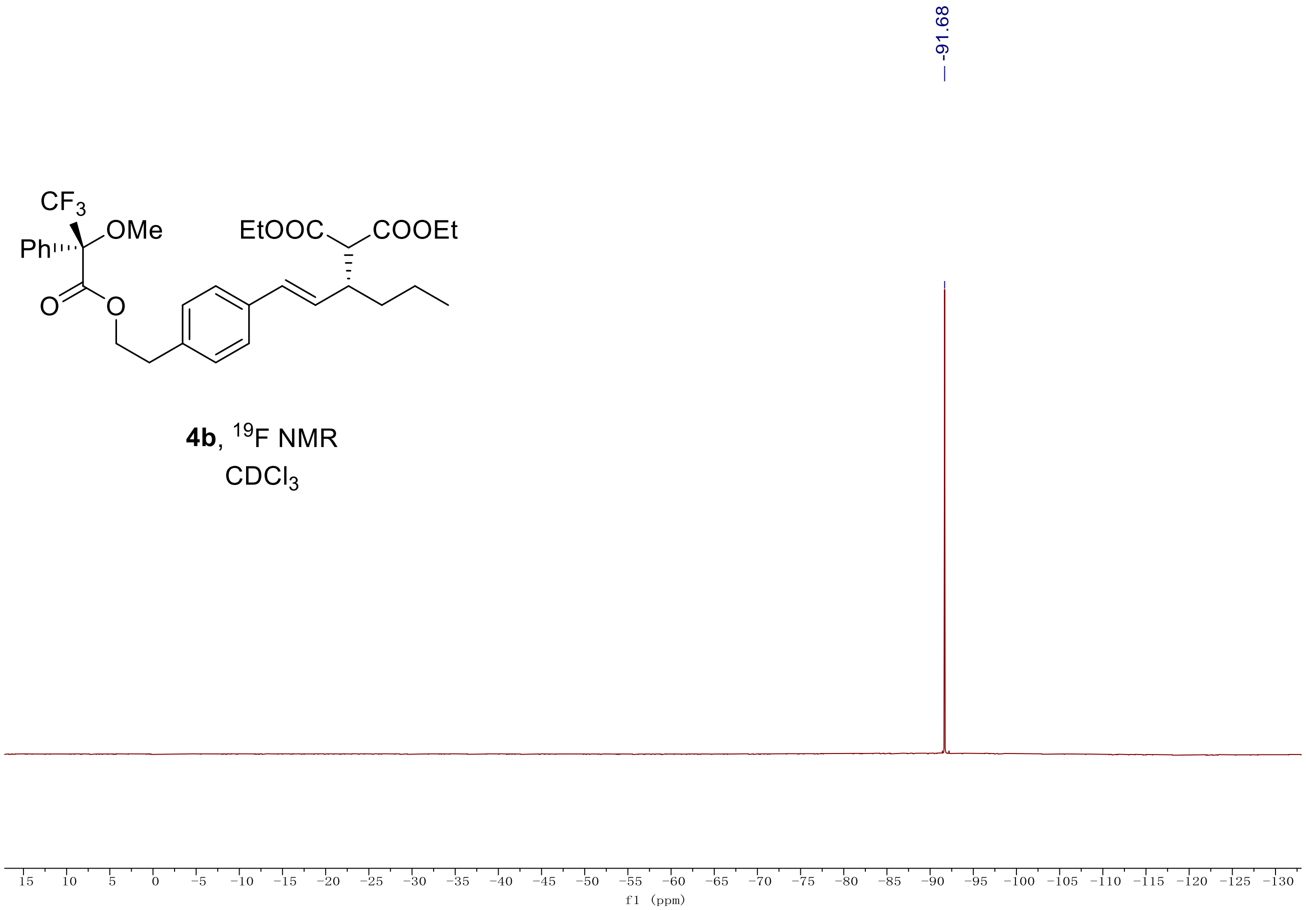

Supplementary Fig. 119. ^{1}H NMR,^{13}C NMR and ^{19}F NMR spectra of compound **4b**.

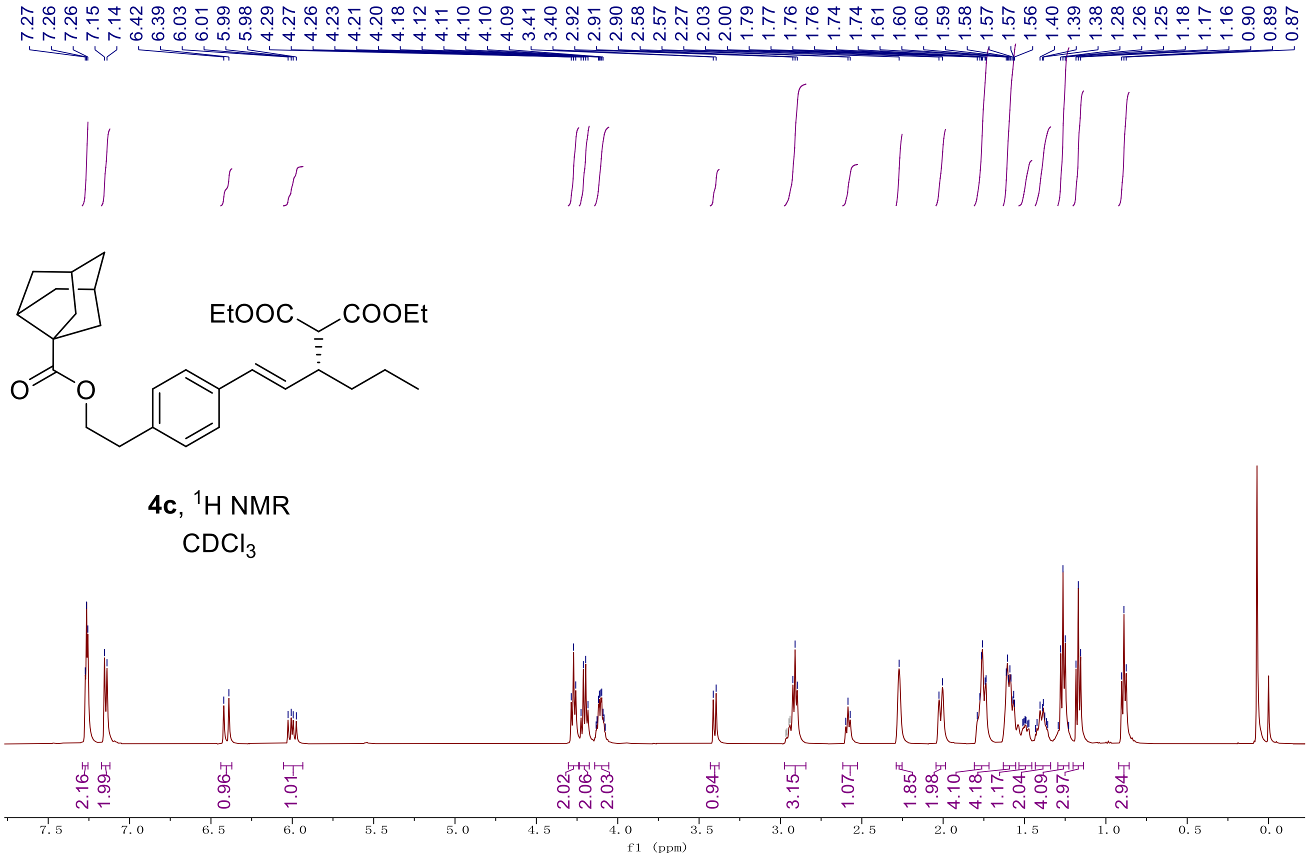

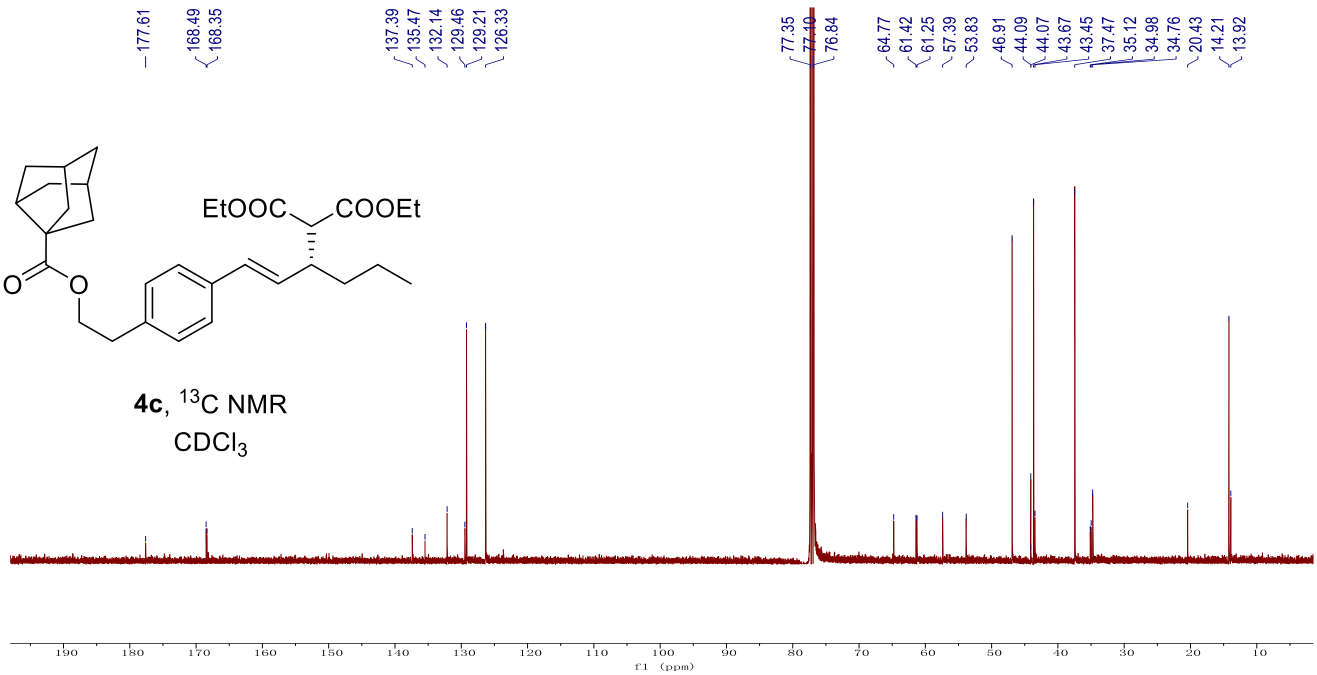

Supplementary Fig. 120. ^{1}H NMR and ^{13}C NMR spectra of compound **4c**.

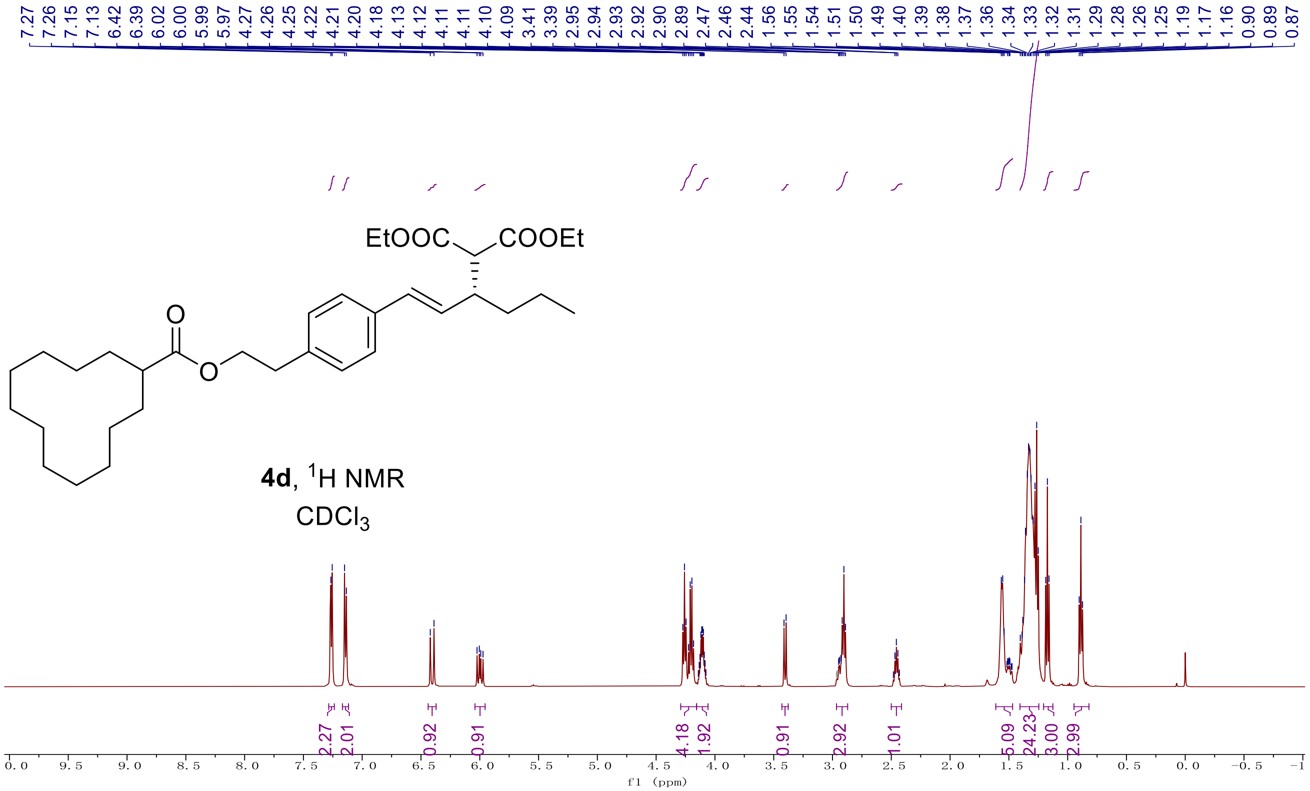

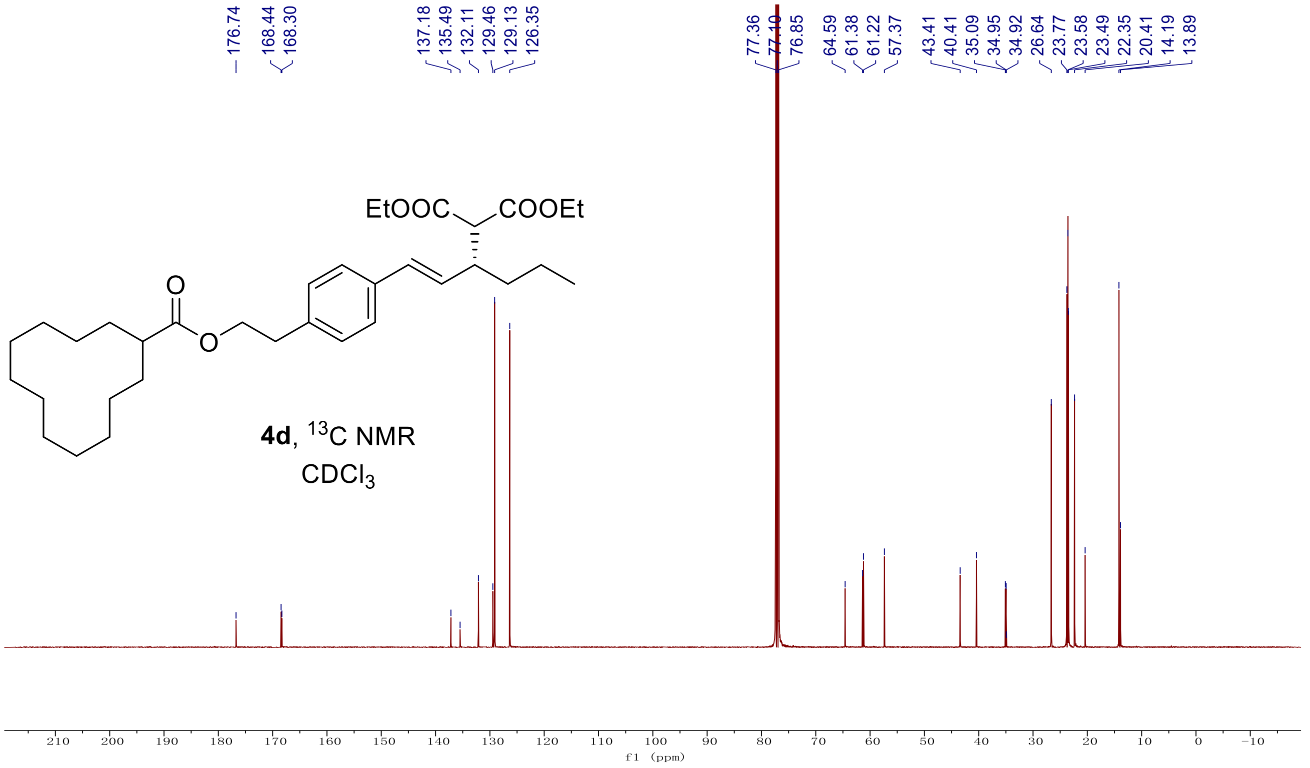

Supplementary Fig. 121. ^{1}H NMR and ^{13}C NMR spectra of compound **4d**.

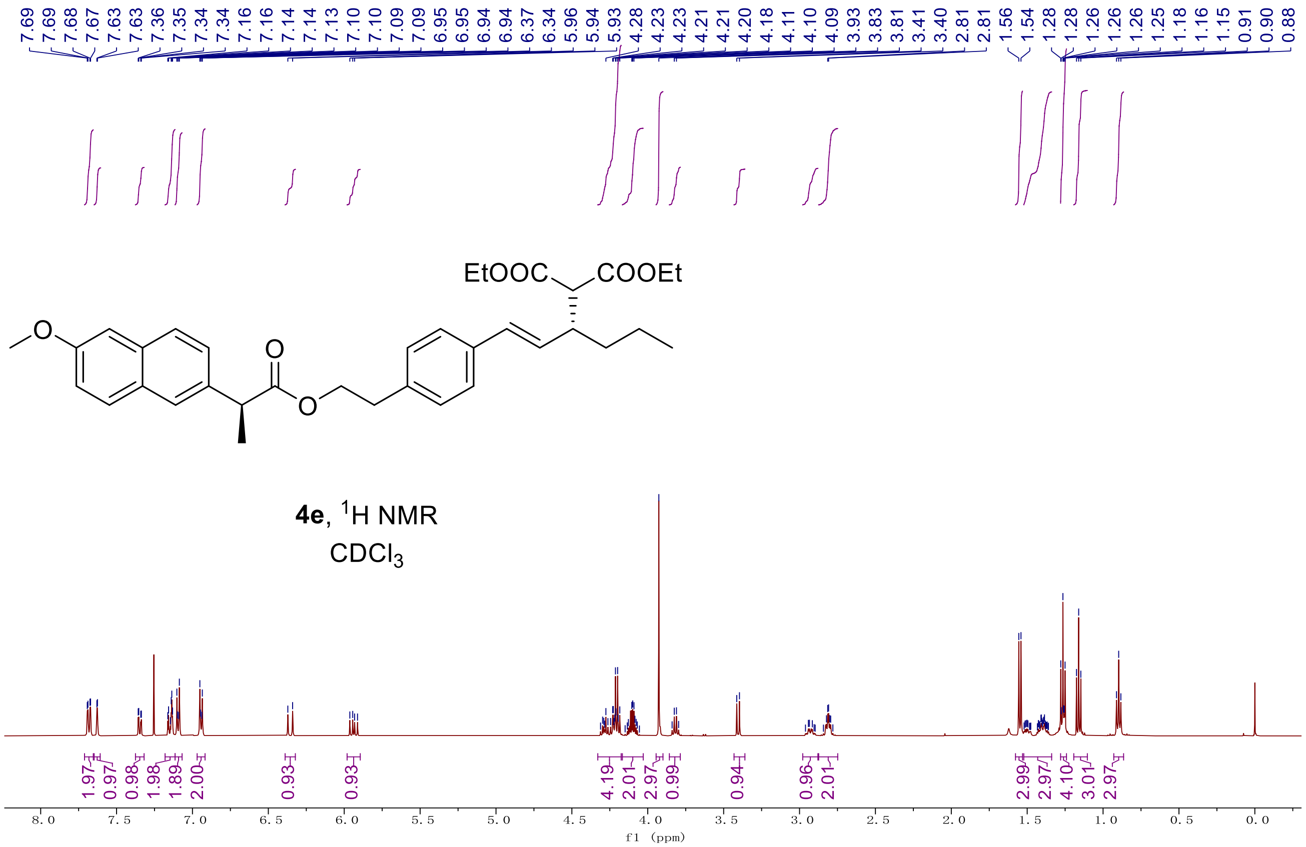

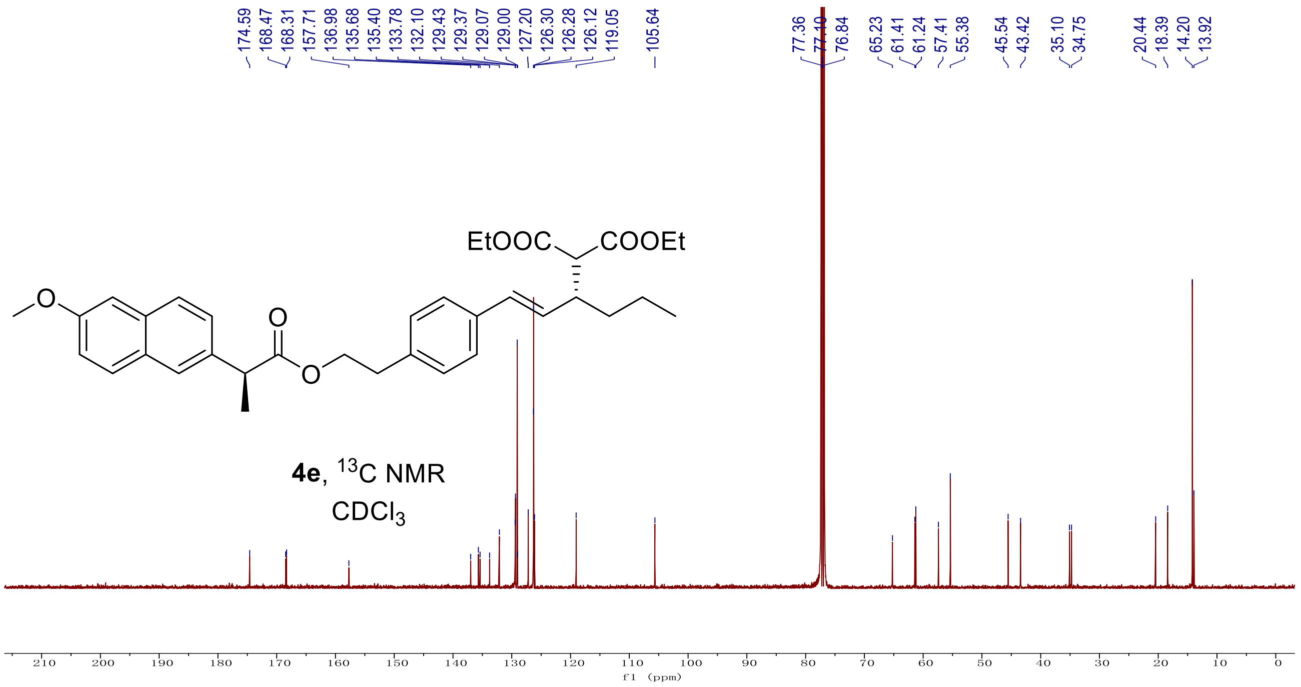

Supplementary Fig. 122. ^{1}H NMR and ^{13}C NMR spectra of compound **4e**.

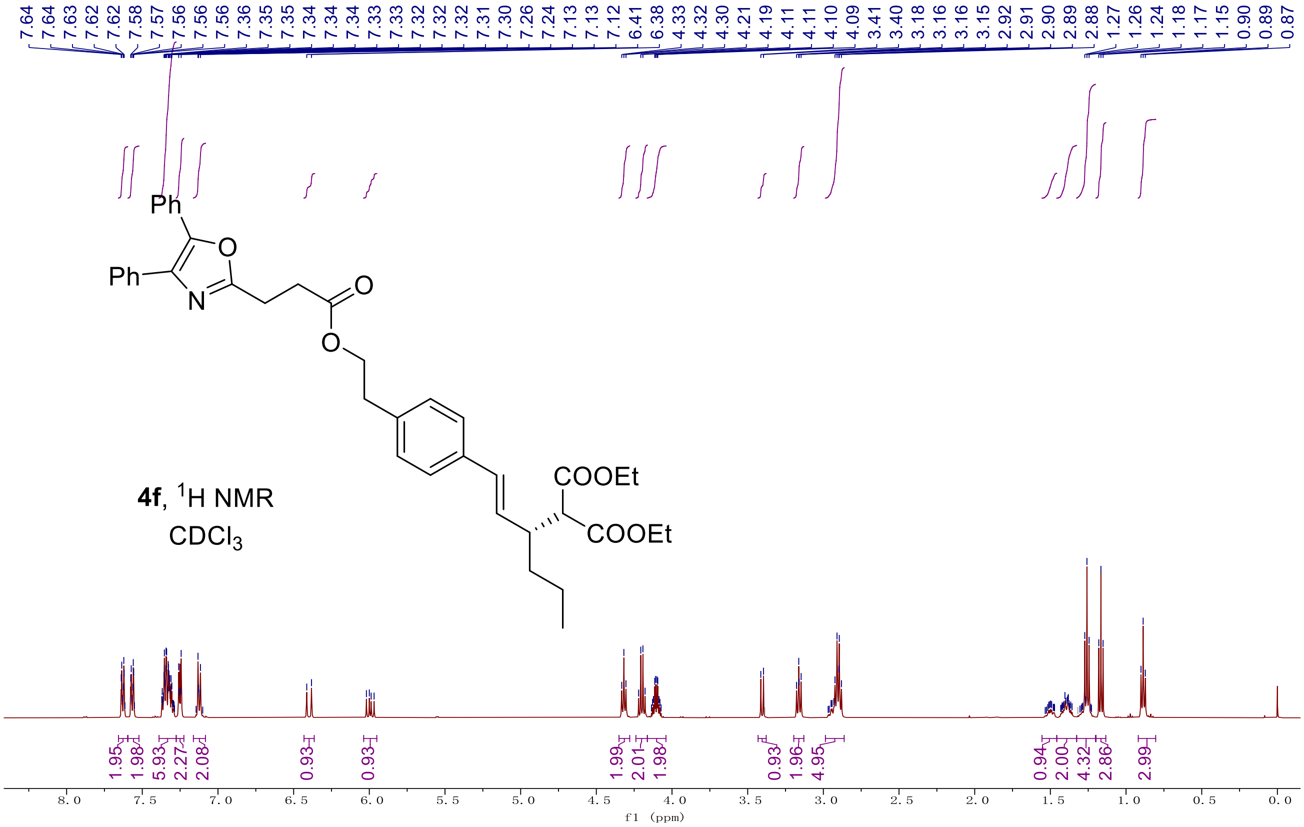

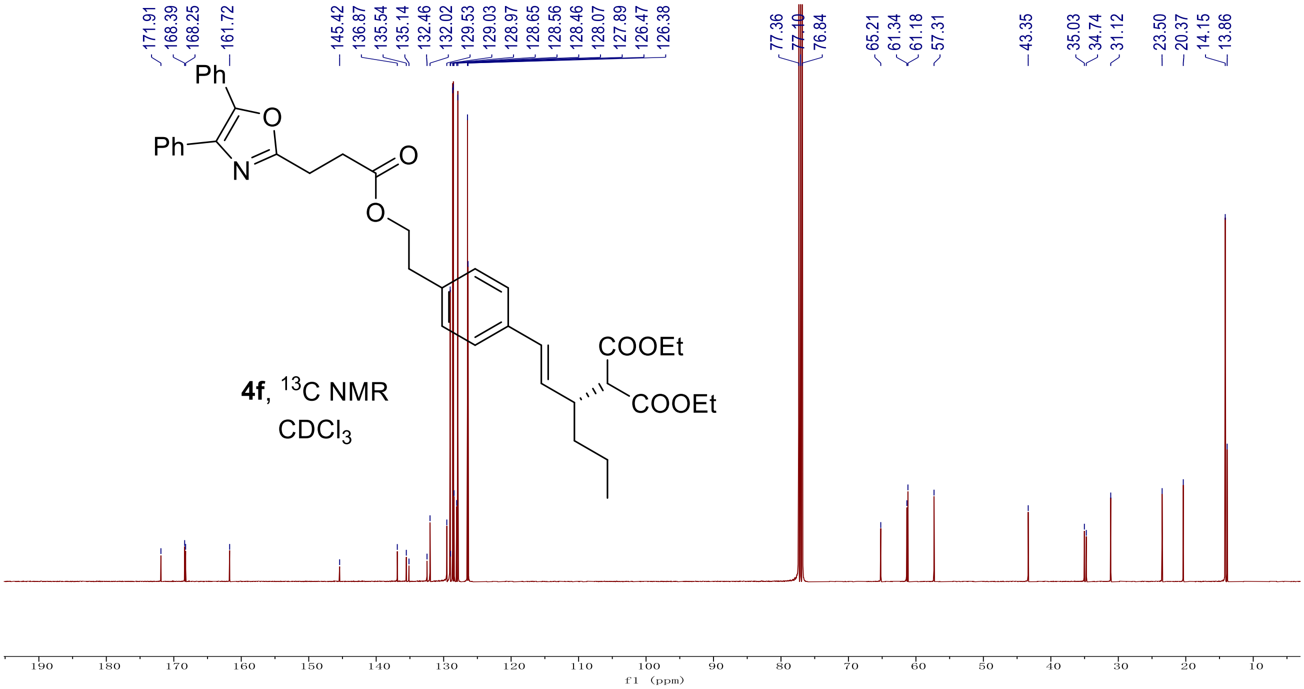

Supplementary Fig. 123. ^{1}H NMR and ^{13}C NMR spectra of compound **4f**.

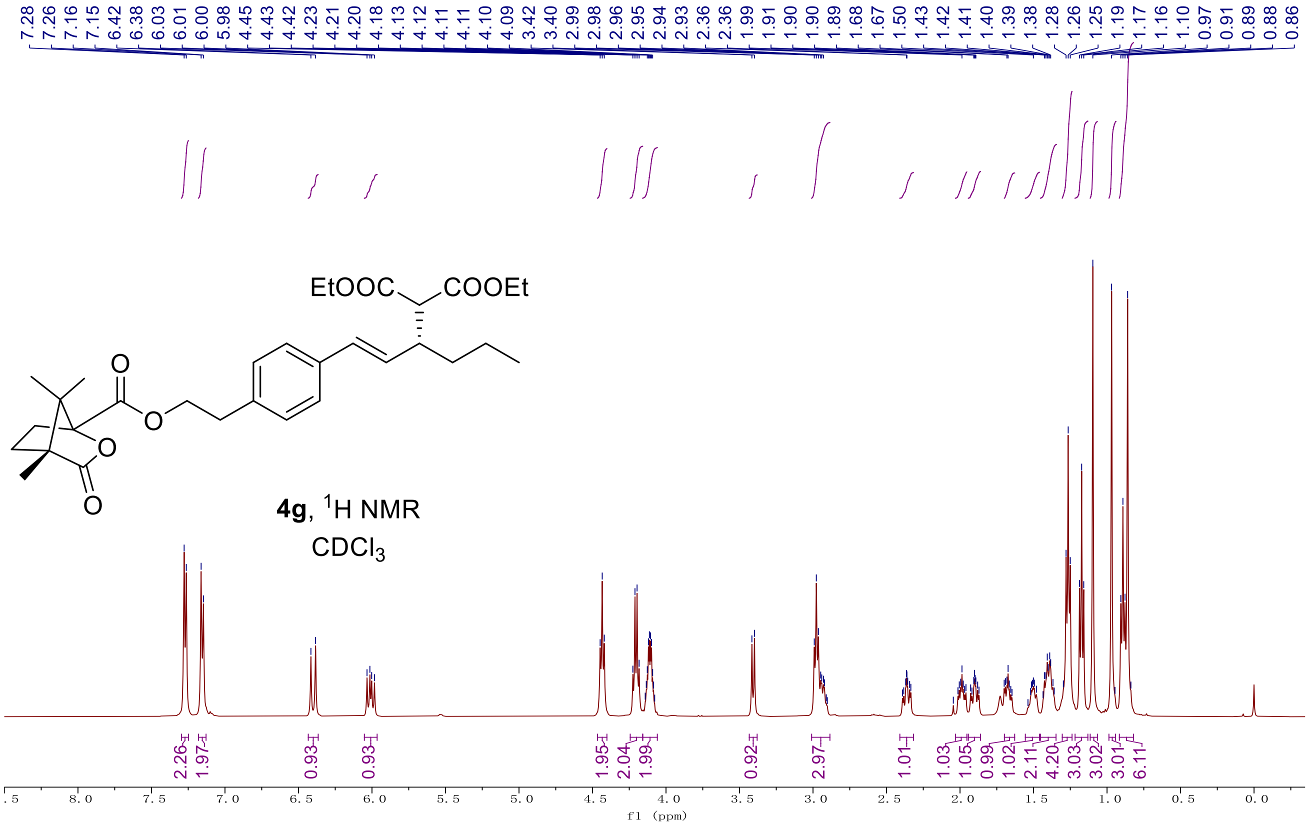

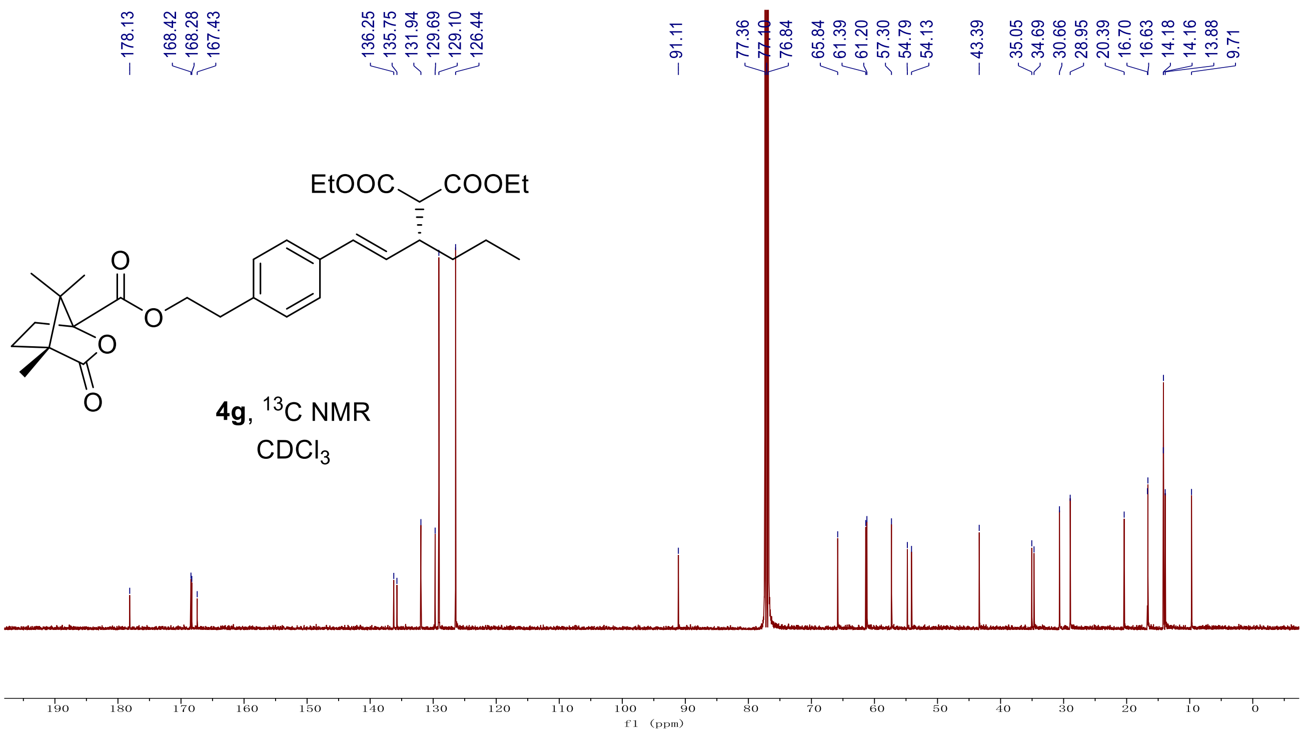

Supplementary Fig. 124. ^{1}H NMR and ^{13}C NMR spectra of compound **4g**.

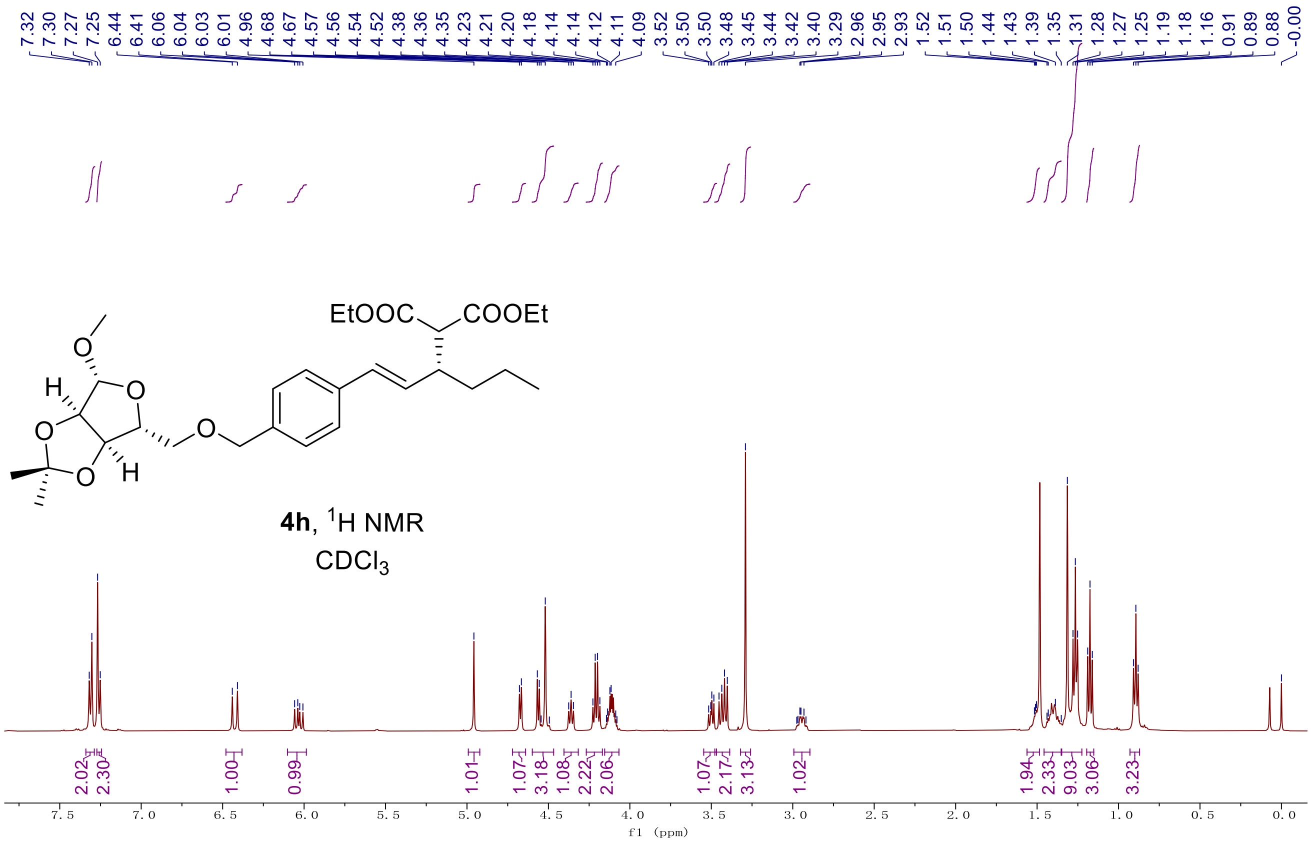

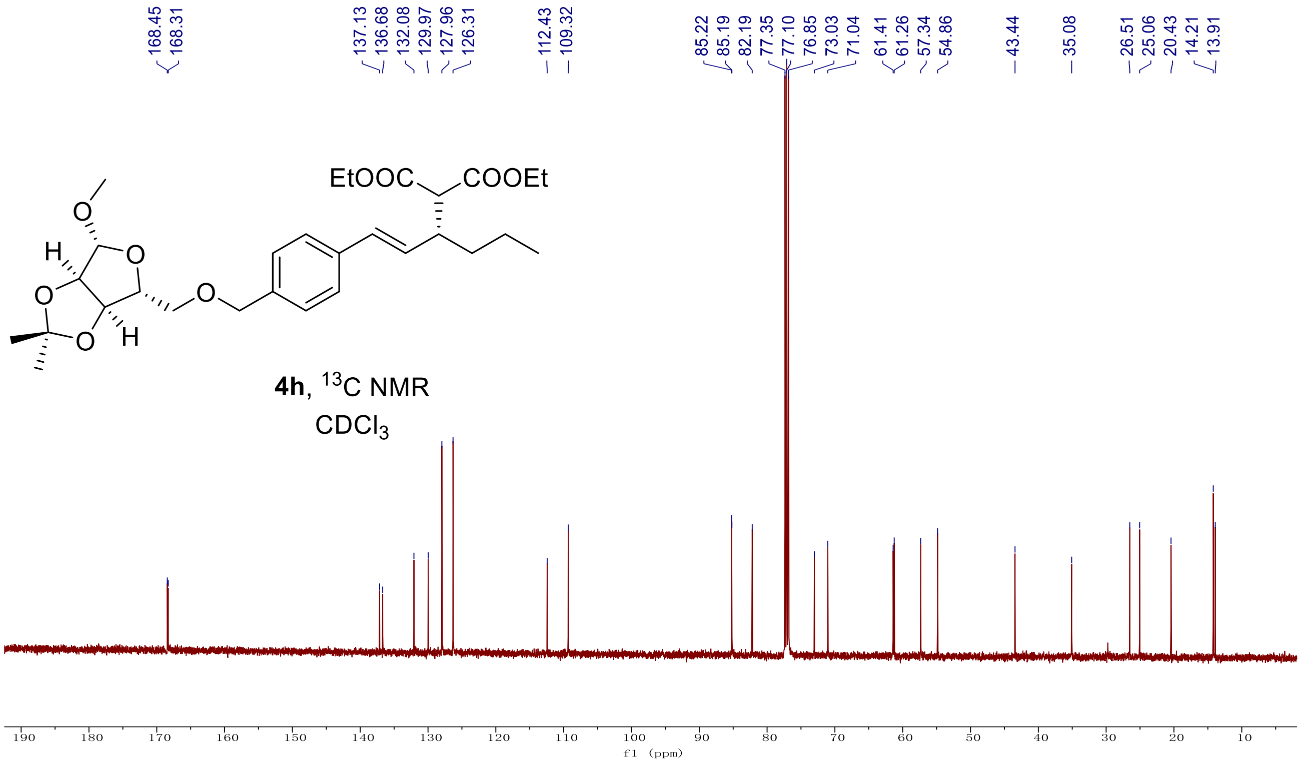

Supplementary Fig. 125. ^{1}H NMR and ^{13}C NMR spectra of compound **4h**.

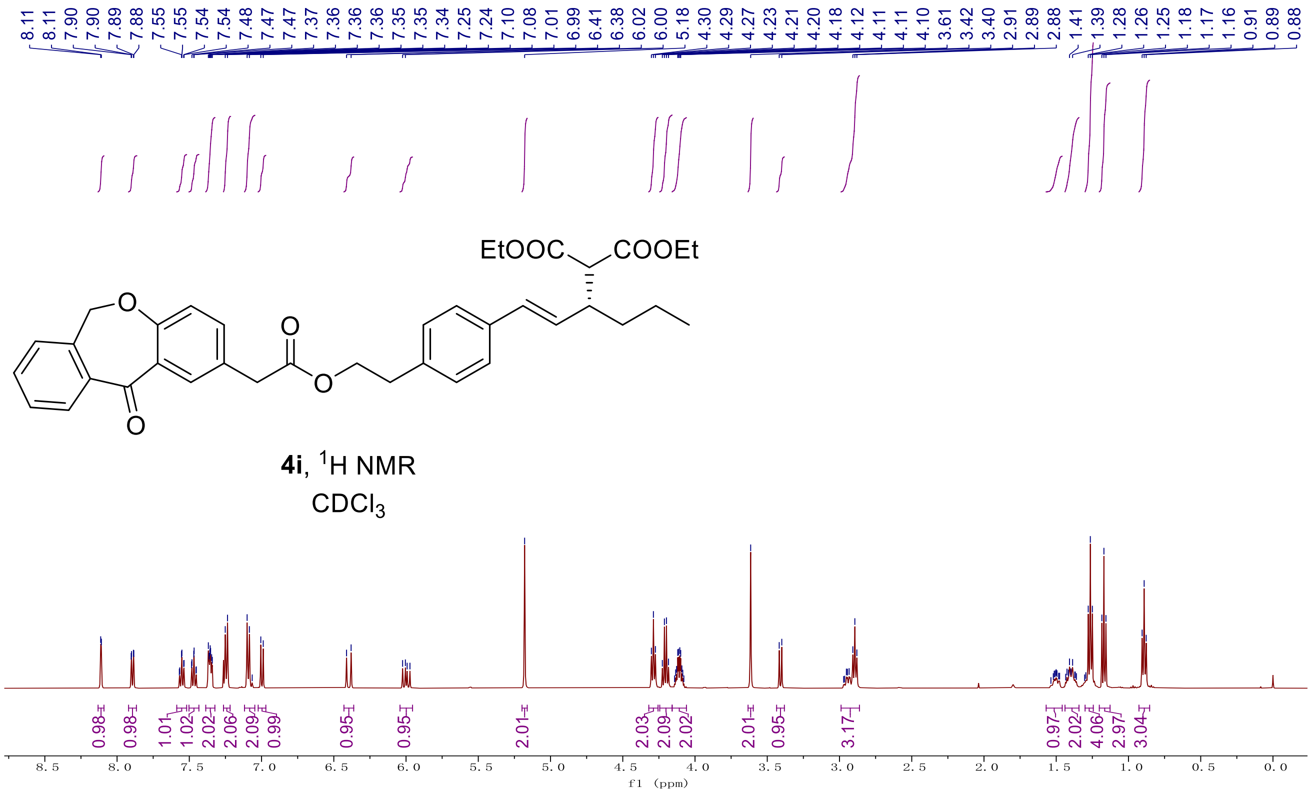

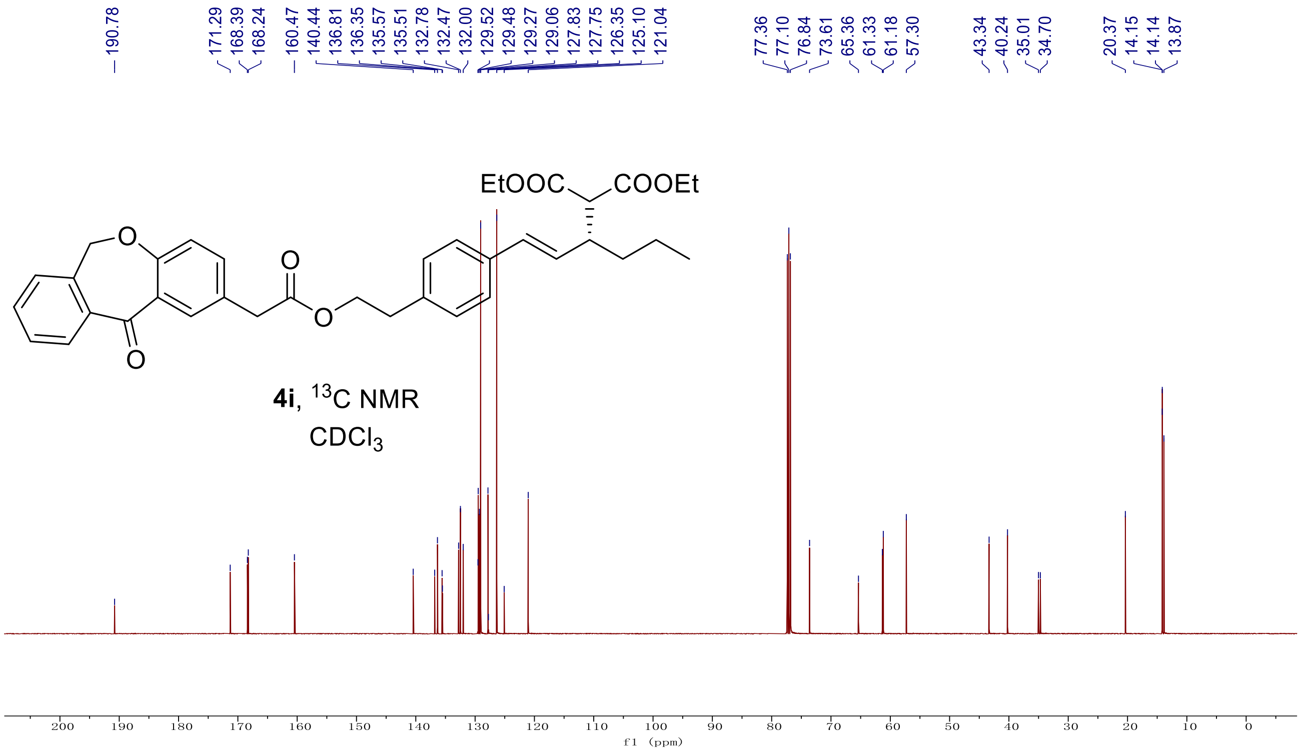

Supplementary Fig. 126. ^{1}H NMR and ^{13}C NMR spectra of compound **4i**.

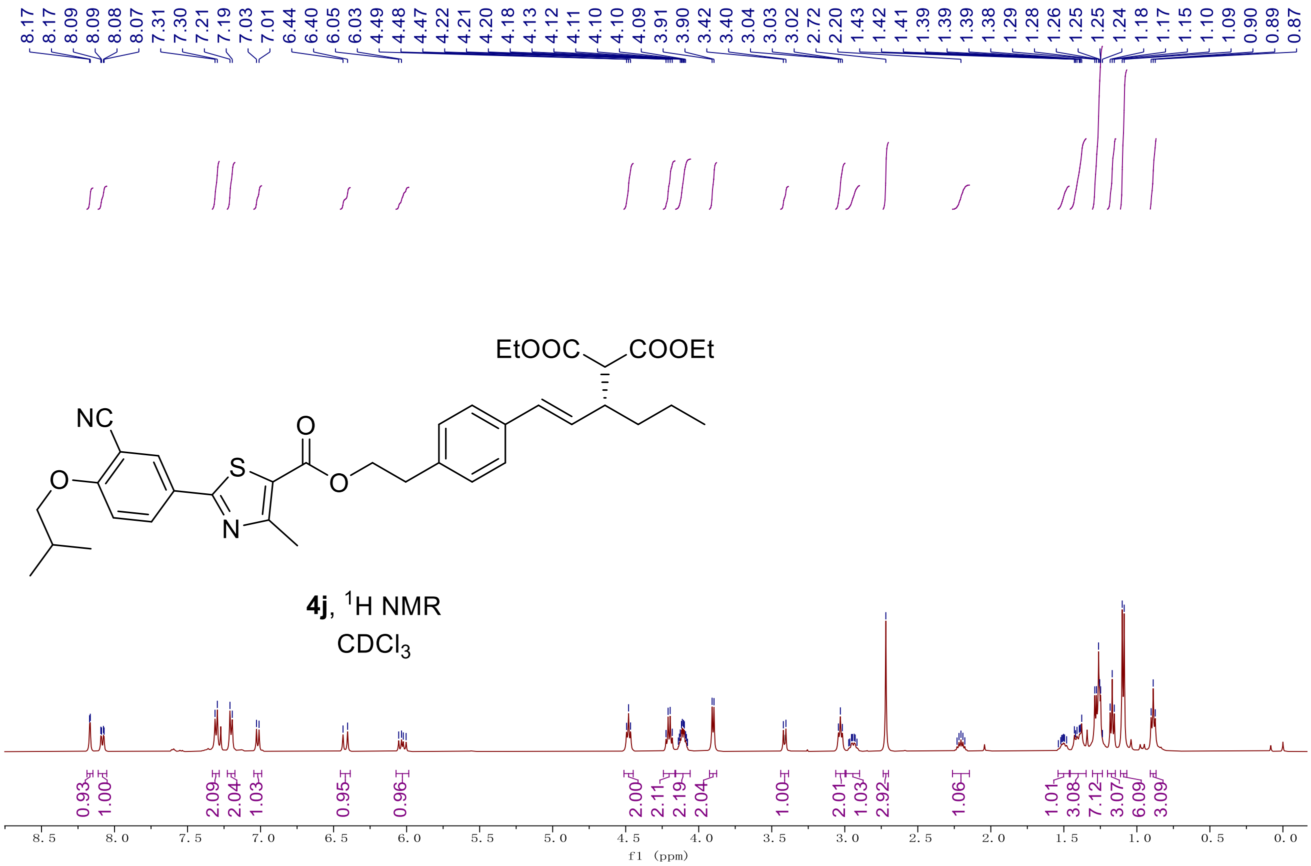

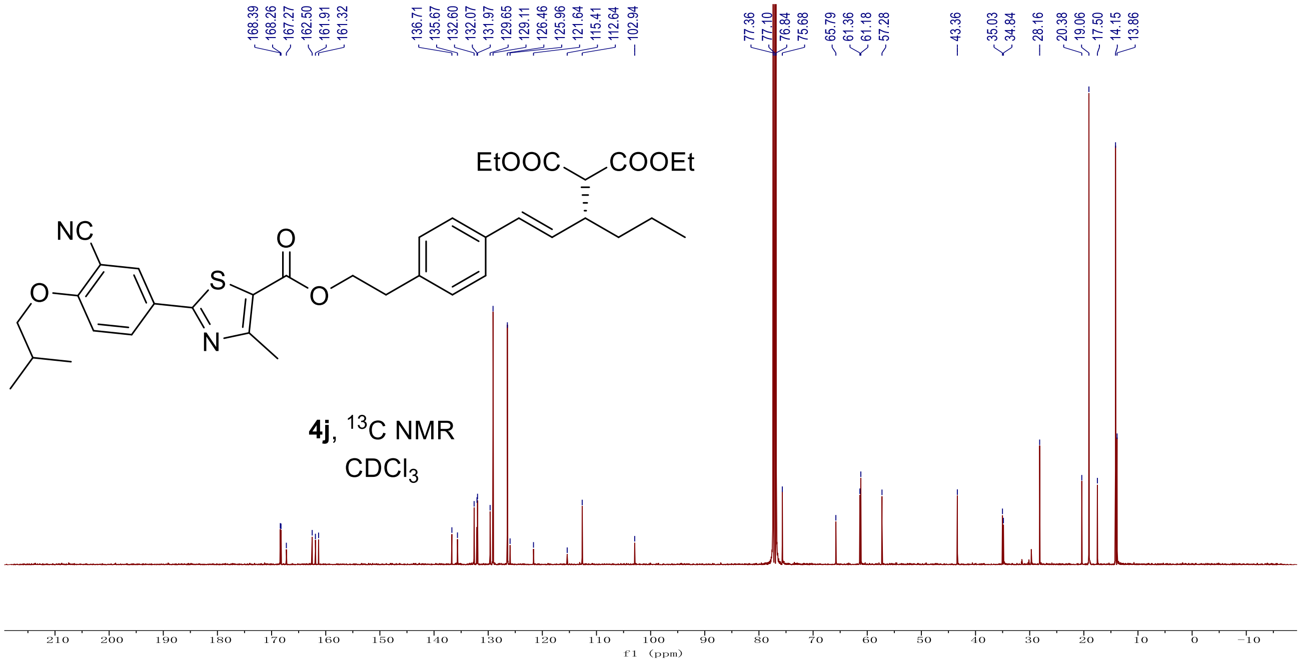

Supplementary Fig. 127. ^{1}H NMR and ^{13}C NMR spectra of compound **4j**.

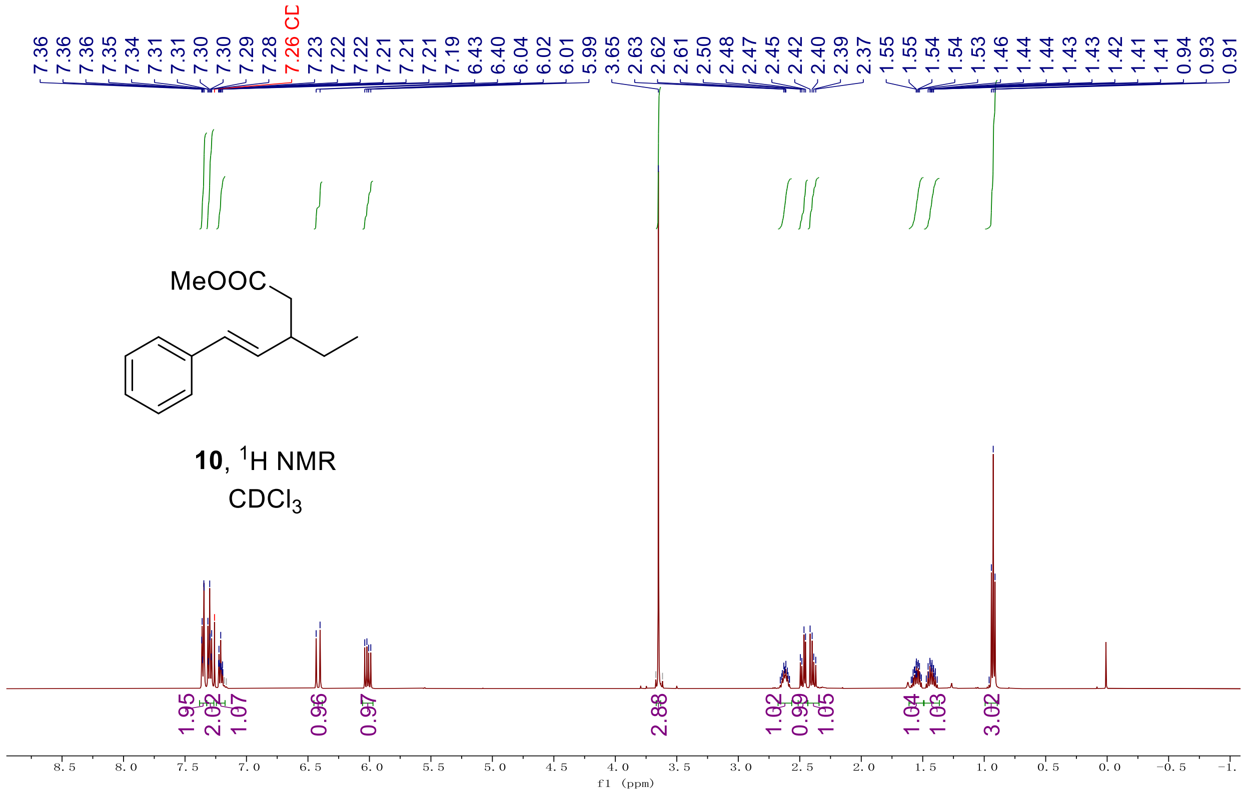

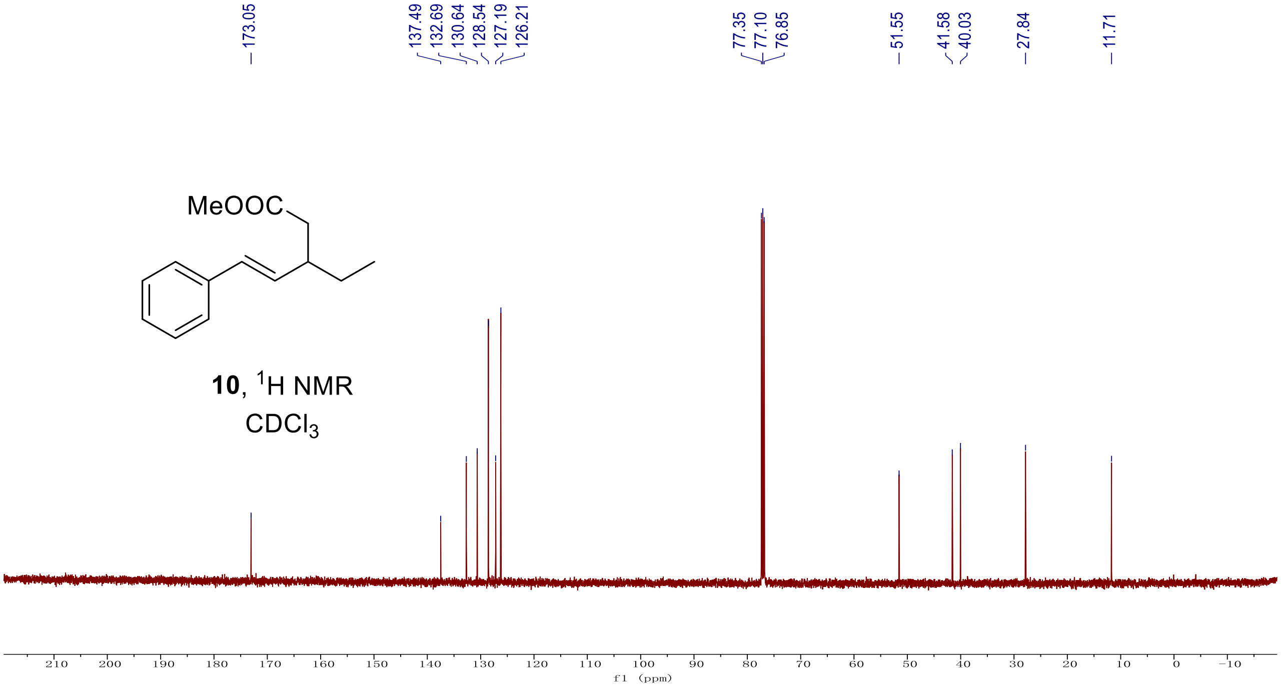

Supplementary Fig. 128. ^{1}H NMR and ^{13}C NMR spectra of compound **10**.

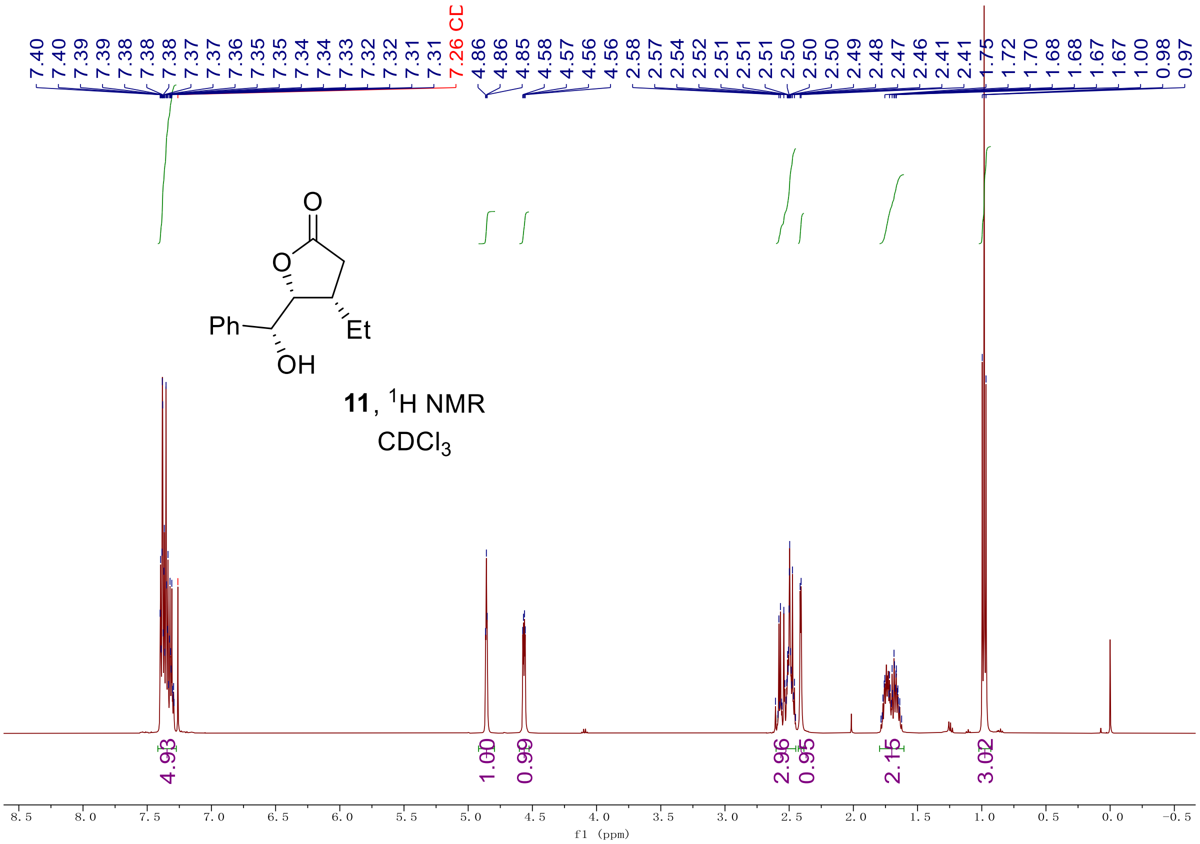

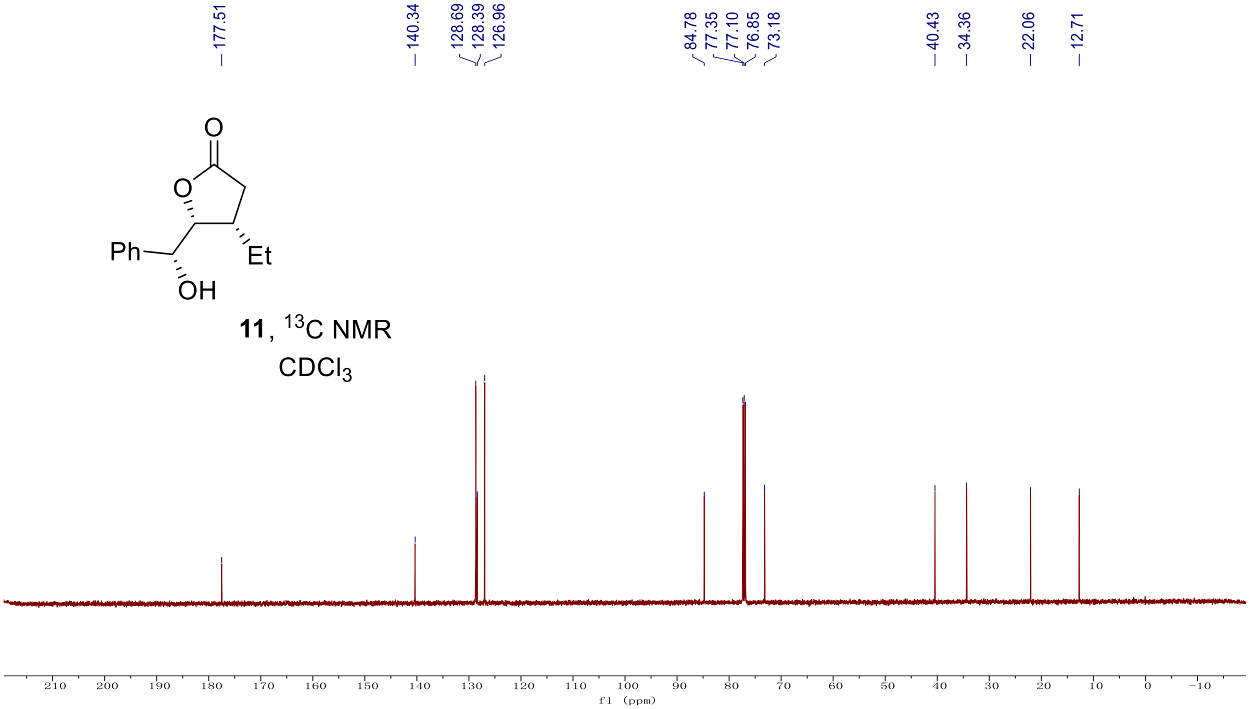

Supplementary Fig. 129. ^{1}H NMR and ^{13}C NMR spectra of compound **11**.

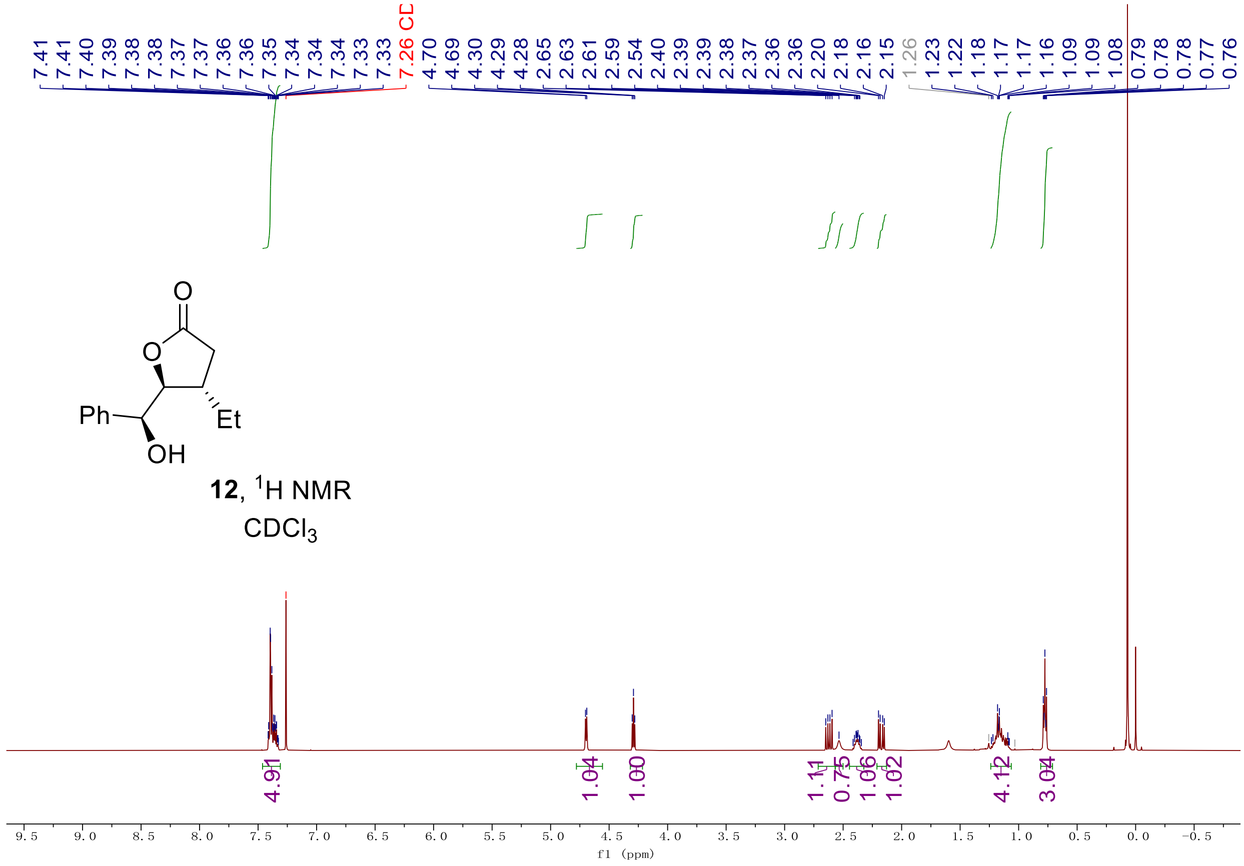

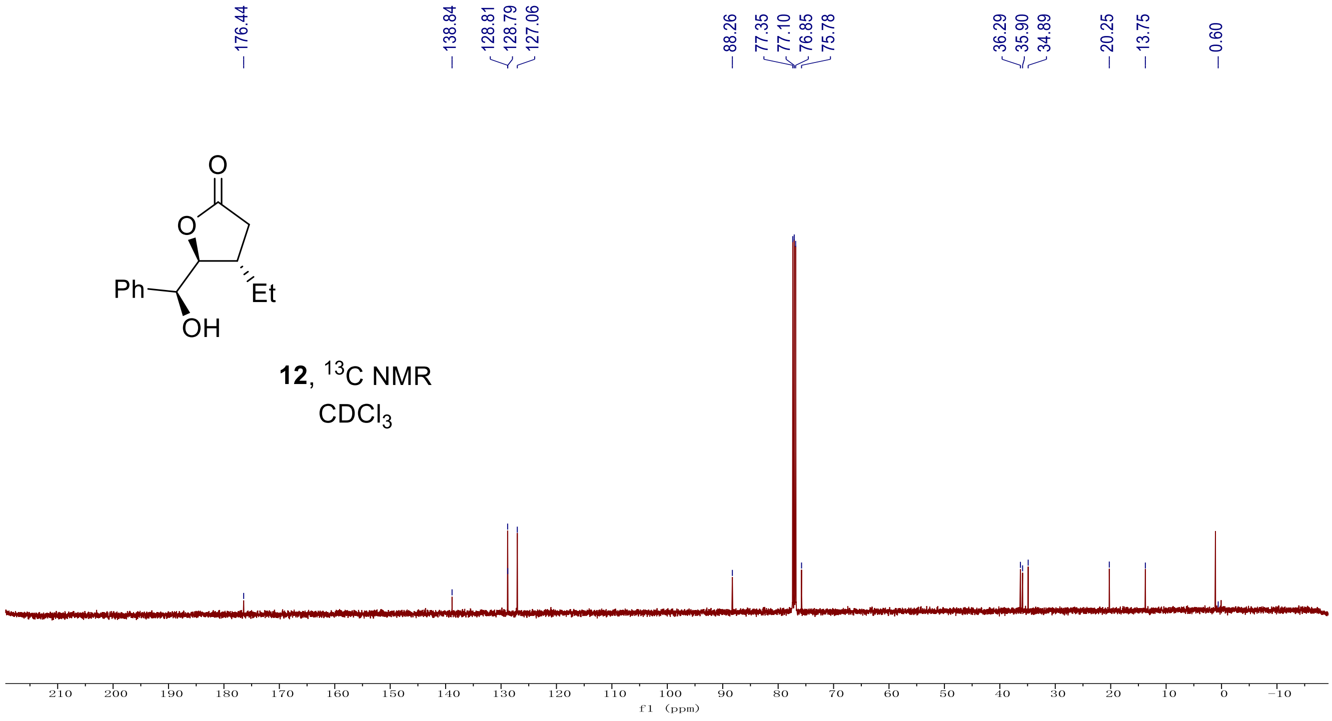

Supplementary Fig. 130. ^{1}H NMR and ^{13}C NMR spectra of compound **12**.

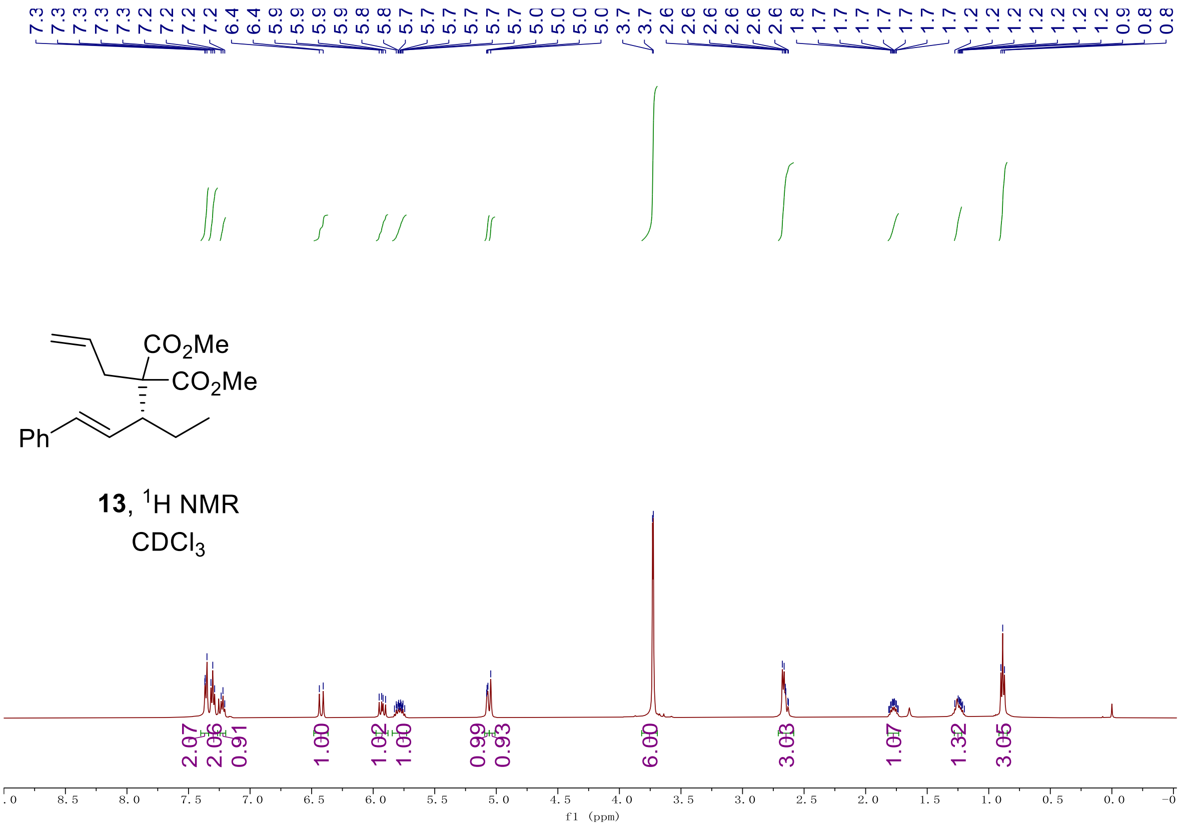

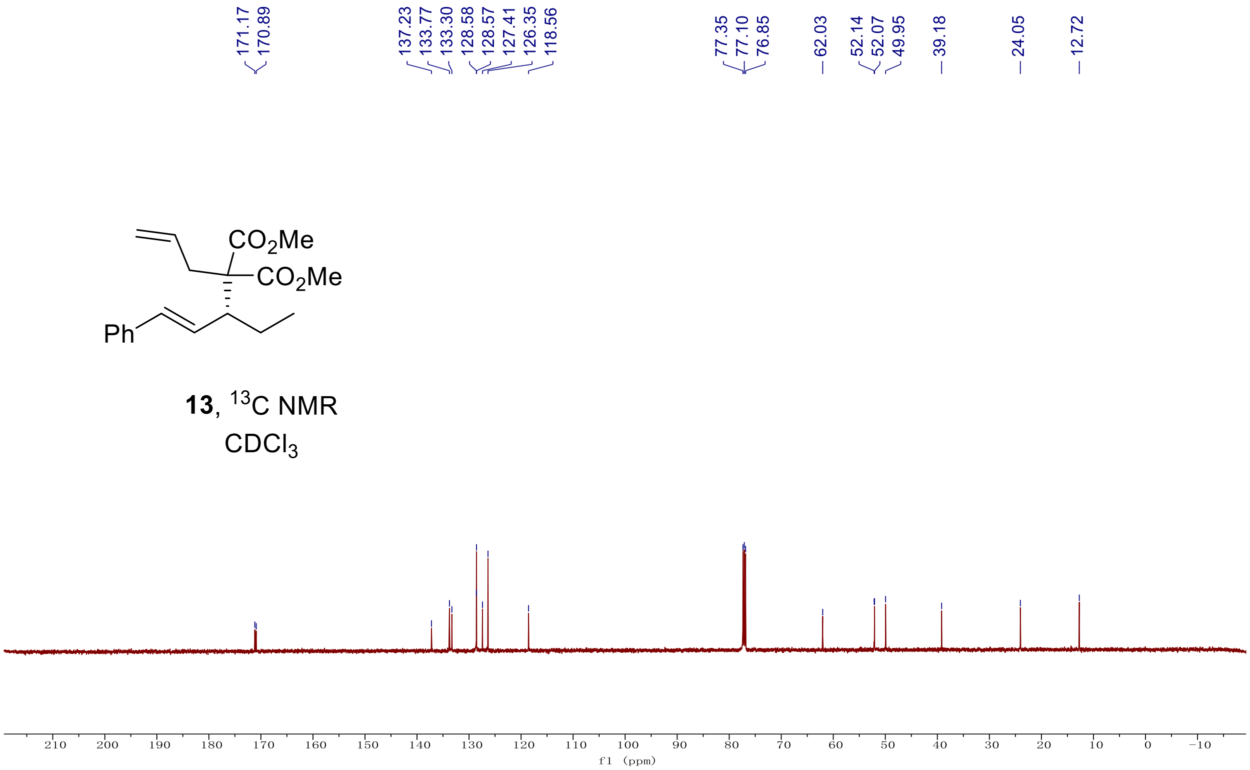

Supplementary Fig. 131. ^{1}H NMR and ^{13}C NMR spectra of compound **13**.

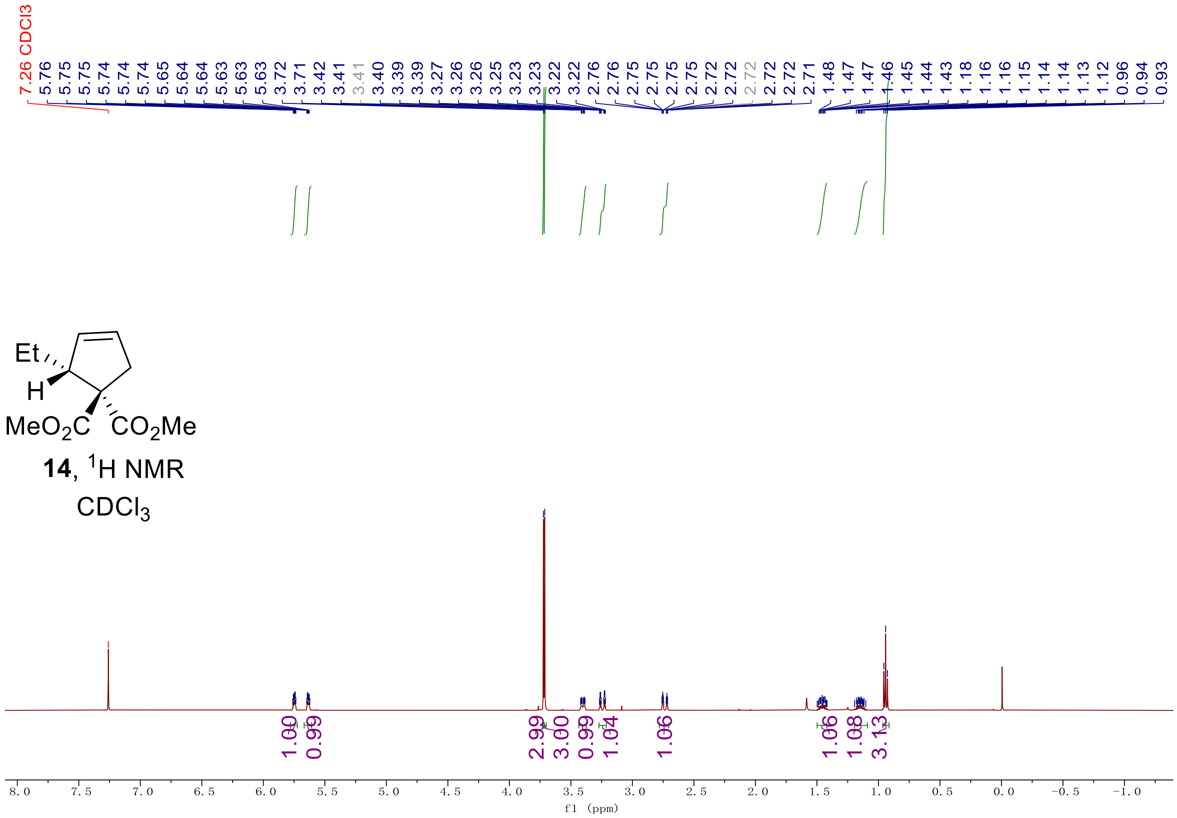

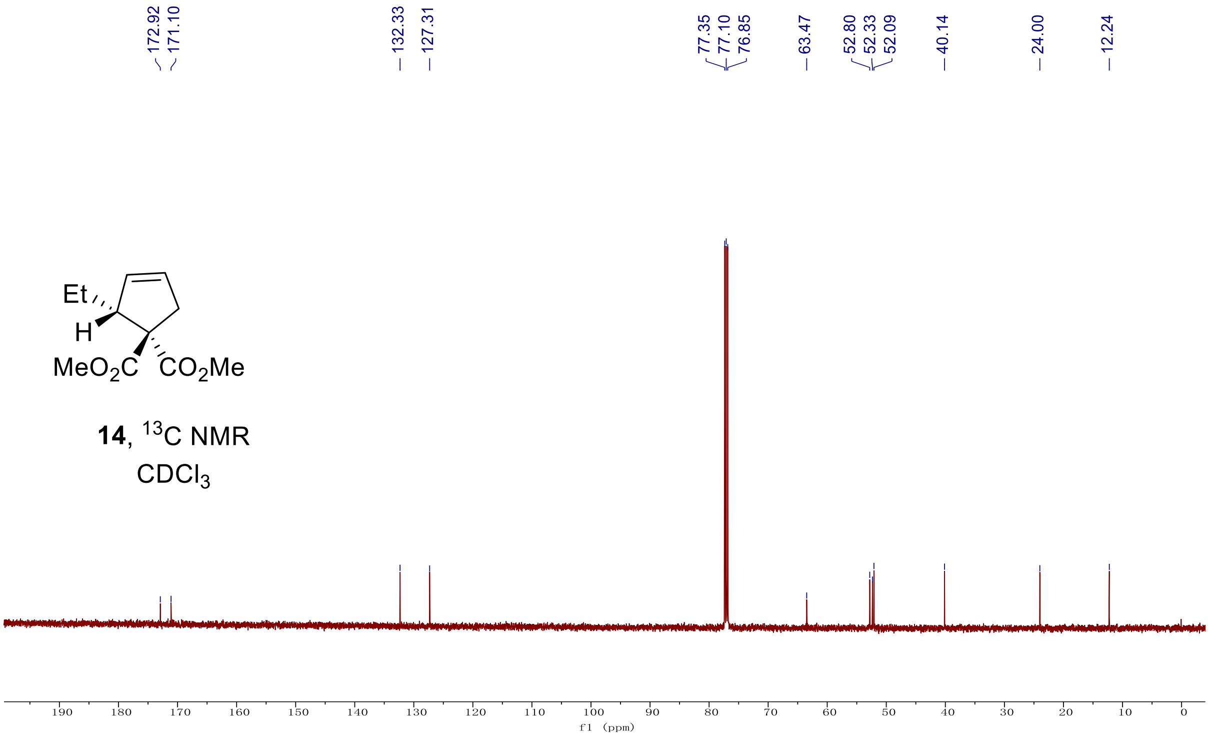

Supplementary Fig. 132. ^{1}H NMR and ^{13}C NMR spectra of compound **14**.

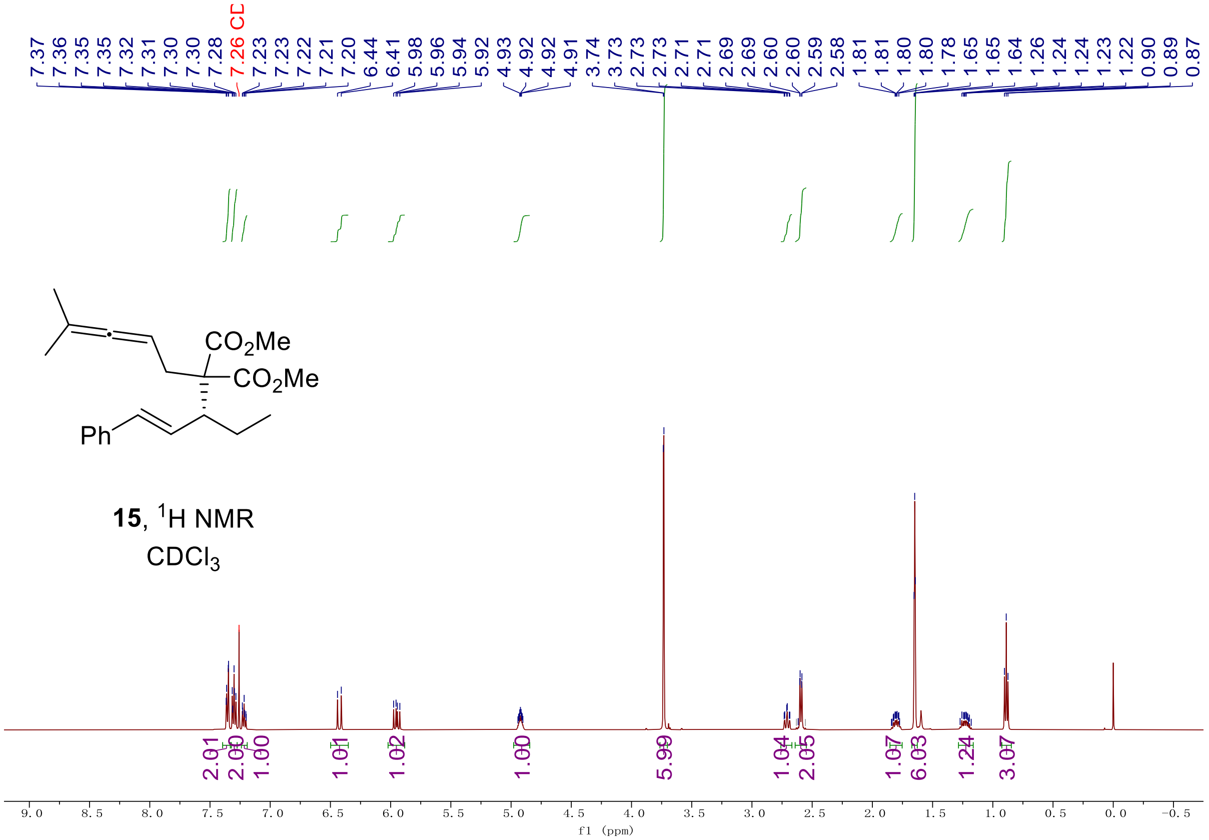

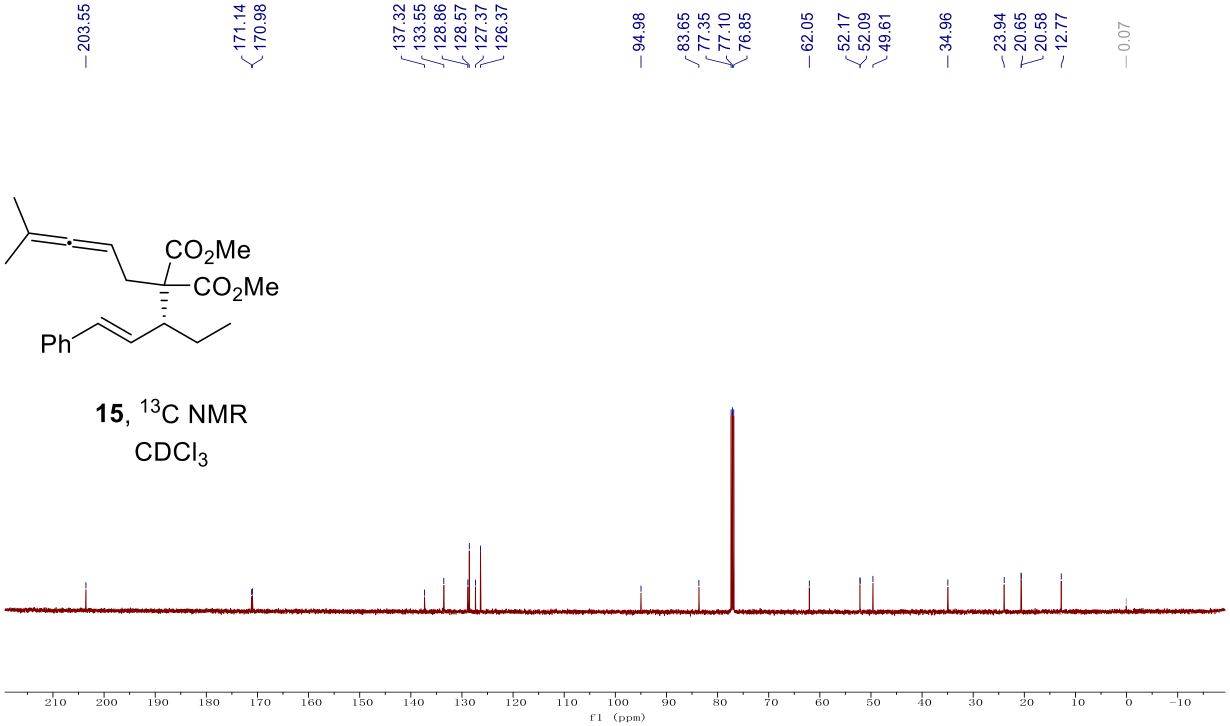

Supplementary Fig. 133. ^{1}H NMR and ^{13}C NMR spectra of compound **15**.

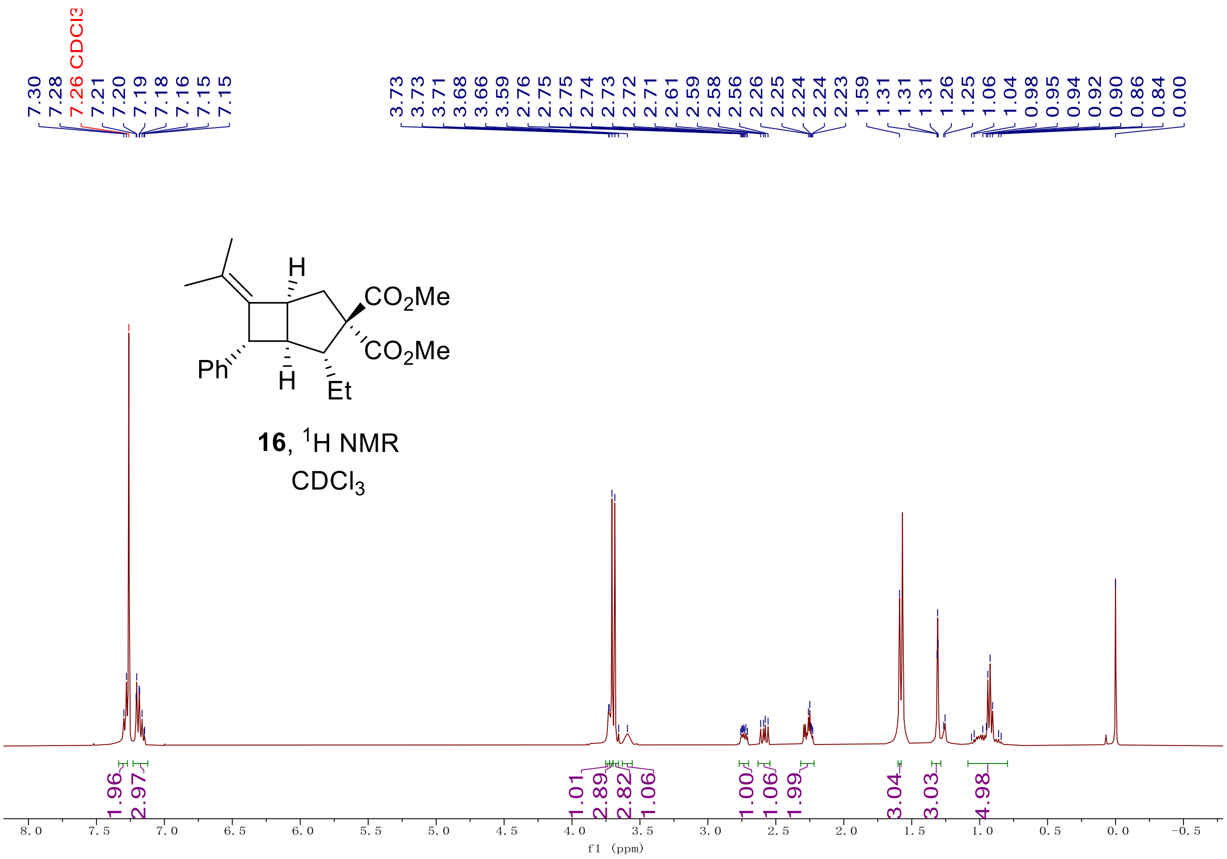

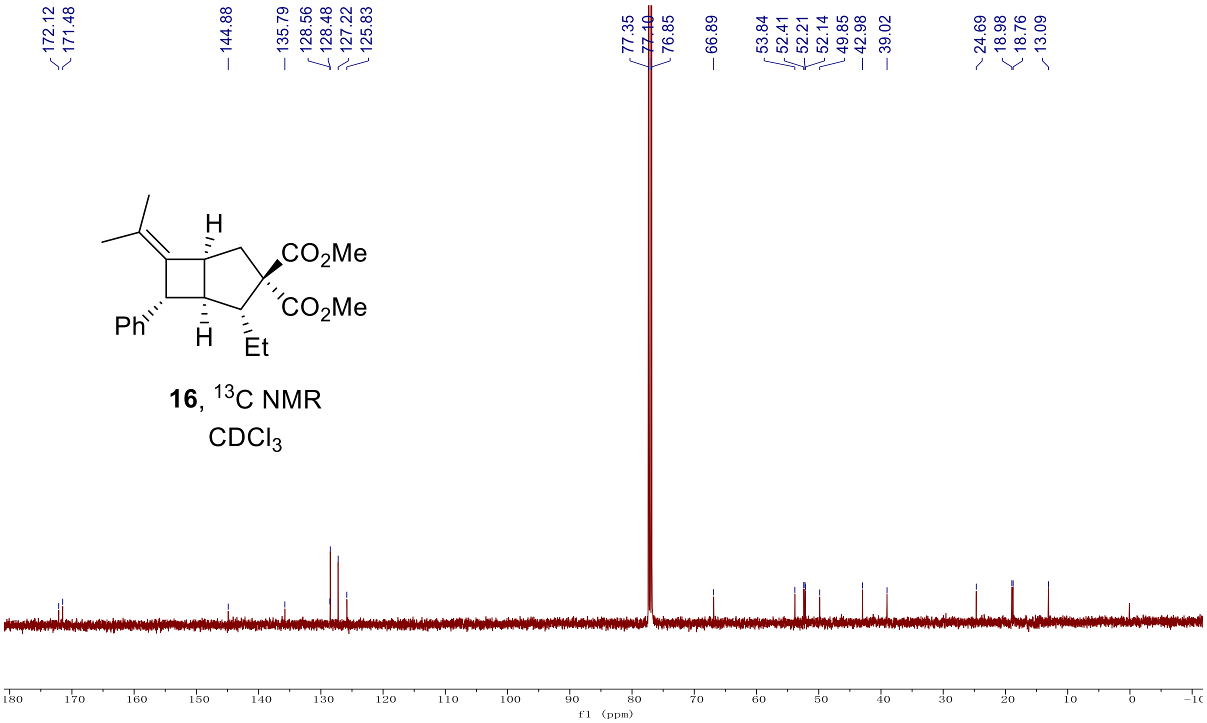

Supplementary Fig. 134. ^{1}H NMR and ^{13}C NMR spectra of compound **16**.

Supplementary References

[1] Li, M. B., Wang, Y., Tian, S.-K. *Angew. Chem. Int. Ed.* **51**, 2968−2971 (2012).
[2] Affo, W., Ohmiya, H., Fujioka, T., Ikeda, Y., Nakamura, T., Yorimitsu, H., Oshima, K., Imamura, Y., Mizuta, T., Miyoshi, K. *J. Am. Chem. Soc.* **128**, 8068−8077 (2006).
[3] Krasovskiy, A. L.; Haley, S.; Voigtritter, K.; Lipshutz, B. H. *Org. Lett.* **2014**, *16*, 4066−4069.
[4] Liu, X., Zhang, W., Wang, Y., Zhang, Z.-X., Jiao, L., Liu, Q. *J. Am. Chem. Soc.* **140**, 6873−6882 (2018).
[5] Sumida, Y., Hayashi, S., Hirano, K., Yorimitsu, H., Oshima, K. *Org. Lett.* **10**, 1629−1632 (2008).
[6] Wang, G.-Z., Shang, R., Fu, Y. *Org. Lett.* **20**, 888−891 (2018).
[7] Ji, D.-W., Hu, Y.-C., Zheng, H., Zhao, C.-Y., Chen, Q.-A., Dong, V. M. *Chem. Sci.* **10**, 6311–6315 (2019).
[8] Azemi, T., Kitamura, M., Narasaka, K. *Tetrahedron* **60**, 1339−1344 (2004).
[9] Jiménez-Aquino, A.; Flegeau, E. F.; Schneider, U.; Kobayashi, S. *Chem. Commun.*, **2011**, *47*, 9456−9458.
[10] Gan, Y., Hu, H., Liu, Y. *Org. Lett.* **22**, 4418−4423 (2020).
[11] Zhang, J.-J., Yang, J.-C., Guo, L.-N., Duan, X.-H. *Chem. Eur. J.* **23**, 10259−10263 (2017).
[12] Sharpless, K. B., Amberg, W., Bennani, Y. L., Crispino, G. A., Hartung, J., Jeong, K. S., Kwong, H. L., Morikawa, K., Wang, Z. M. *J. Org. Chem.* **57**, 2768−2771 (1992).
